# Genetic Insights Into Human‐Driven Hybridization, Cultural Shifts, and Ecological Consequences of Feral Pigs (*Sus scrofa*) in Hawai‘i

**DOI:** 10.1002/ece3.72822

**Published:** 2026-01-05

**Authors:** Anna M. Mangan, Timothy J. Smyser, Nicolai Barca, Steven C. Hess, Kealohanuiopuna M. Kinney, Darrin Phelps, Nathaniel H. Wehr, Dominic Wright, Antoinette J. Piaggio

**Affiliations:** ^1^ United States Department of Agriculture, Animal and Plant Health Inspection Service Wildlife Services, National Wildlife Research Center Fort Collins Colorado USA; ^2^ The Nature Conservancy Hawai‘i Honolulu Hawaii USA; ^3^ United States Department of Agriculture, Animal and Plant Health Inspection Service Wildlife Services, National Wildlife Research Center Hilo Hawaii USA; ^4^ Institute for Pacific Islands Forestry United States Forest Service Hilo Hawaii USA; ^5^ United States Department of Agriculture, Animal and Plant Health Inspection Service Wildlife Services Honolulu Hawaii USA; ^6^ Department of Natural Resources and Environmental Management University of Hawai‘i at Mānoa Honolulu Hawaii USA; ^7^ Department of Physics, Chemistry and Biology Linköping University Linköping Sweden

**Keywords:** ancestry, hybridization, Polynesian, SNP, *Sus scrofa*

## Abstract

Feral pigs ( *Sus scrofa* ) in Hawai‘i pose a persistent threat to native biodiversity, endemic species, and culturally important resources. Polynesian pigs, or *pua‘a,* were brought to the Hawaiian Islands with Polynesian settlement in the mid‐1200s and represent part of the cultural legacy of Hawai‘i. Since the introduction of European pigs in 1778 and onward, the ancestral composition of contemporary animals has been debated, and conservation efforts for island endemic species have been challenged by tension between ecological destruction caused by contemporary feral pigs and the cultural importance of this animal. To inform this complex issue, our objective was to evaluate the genetic ancestry of contemporary feral pig populations across Hawai‘i to elucidate genetic remnants of past introductions. We used a high‐resolution single nucleotide polymorphism (SNP) array, providing a survey of the entire genome, to characterize ancestry, including hybridization of Polynesian pigs with global Asian and European lineages. We assembled a comprehensive reference set—representing the *S. scrofa* wild–domestic species complex—from which we queried 608 Hawaiian feral pig samples to quantify ancestral composition. Our results demonstrate that contemporary Hawaiian feral pigs have admixed ancestry influenced by European Heritage breeds and animals of Asian origin—potentially descending from initial Polynesian introductions. Importantly, we establish that European domestic lineages represent the dominant ancestral influence among contemporary feral pigs in Hawai‘i, which challenges previous claims of genetic uniqueness of these populations within the broader *S. scrofa* wild–domestic species complex.

## Graphical abstract

Polynesian pigs were brought to the Hawaiian Islands with Polynesian settlement in the mid‐1200s and represent part of the cultural legacy of Hawai‘i. Yet, the introduction of European pigs since 1778 and onward has put into question the ancestral composition of contemporary animals, and conservation efforts have been challenged by tension between the ecological destruction caused by contemporary feral pigs and the cultural importance of this animal. Using a high‐resolution single nucleotide polymorphism array, we demonstrate the hybrid ancestry of contemporary feral pigs, characterized by a dominant ancestral influence from European domestic lineages. Our results challenge previous claims of genetic uniqueness of these populations and add to the body of literature focusing on this complex conservation topic.

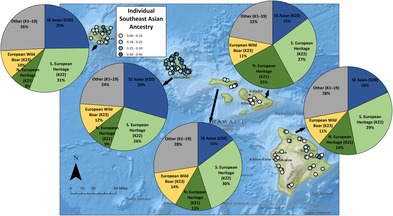

## 1 Introduction

Most of the Pacific Islands lacked terrestrial mammals prior to human arrival (Hess et al. 2019), but over the centuries, anthropogenic movement has accidentally or intentionally introduced many species to these remote island systems. Among the Hawaiian Islands, 19 mammals have been introduced including rodents (*Rodentia*), mongoose (*Urva auropunctata*), cattle ( *Bos taurus* ), and pigs ( *Sus scrofa* ) (Tomich 1986; Hess 2016). Archeological evidence demonstrates that pigs were first introduced to the Hawaiian archipelago with Polynesian settlement in the early to mid‐1200s (Wilmshurst et al. 2011). Polynesian pigs represented a distinct Pacific lineage of *S. scrofa* that appears to have originated in peninsular Southeast Asia and were carried throughout Oceania with Polynesian migration and settlement (Larson et al. 2007; Nogueira‐Filho et al. 2009; Hess et al. 2019; Horsburgh et al. 2022). Although to date, genotypes of Polynesian pigs have not been published, skeletal remains were documented at some of the earliest known Polynesian settlements on the islands of O‘ahu and Moloka‘i (Kirch 2021) and near a prehistoric habitation site on Kaua‘i (Burney et al. 2001).

Given their long history on the islands, pigs are represented in the cultural legacy and cosmogony of Hawaii's Indigenous people (Kirch and O'Day 2003). In particular, the hog child, *Kamapua‘a*—a mischievous and adventurous half‐man, half‐hog demigod—is associated with supernatural values such as the creation of landscape features and weather patterns (Dorton 1982; Hess et al. 2019). Humans are preceded by *Kamapua‘a* in the *Kumulipo*—the Hawaiian creation story that traces the origin of life from darkness to light, connecting gods, nature, and humans (Beckwith 2000). Polynesian pigs (*pua‘a* in Hawaiian) were traditionally used in widespread ritual slaughter during religious and ceremonial occasions and represented a prestigious possession and source of food (Giovas 2006). There are accounts of sacred pigs that were left to roam the region (Ellis 1827), but more frequently, *pua‘a* were raised in close association with villages and were dependent on anthropogenic food sources as the native ecosystems of the Hawaiian Islands were believed to provide limited foraging resources (Kirch and O'Day 2003; Wehr et al. 2018). *Nupepa* (Hawaiian Language newspapers) and other sources describing the ancient past, depict how pigs were classified in detail based on traits such as color, size, and sex, with at least 11 different names distinguishing various types of *pua‘a* (Malo 1903; Pukui and Elbert 1986).

The first introduction of European pigs to Hawai‘i coincided with Captain James Cook's voyage in 1778, with numerous introductions to the islands thereafter (Mayer and Brisbin 2008; Nogueira‐Filho et al. 2009). Pigs had been independently domesticated in Asia and Europe, and these domestic lineages reflect the deep phylogenetic divergence present among their wild progenitors that occurred approximately 1.6–0.8 m.y.a. (Groenen et al. 2012). Therefore, the subsequent introduction of European animals to Hawai‘i began setting the stage for the admixture of these long‐separated lineages. Historical records and voyage journals suggest that early European explorers transported pigs aboard tall ships for various reasons, such as food sources, to stock ports with provisions for future voyages, as gifts for local chiefs, and in some cases for purposes of providing new and improved breeds to Indigenous peoples (Cook 1842; Colnett 1968).

The association with people and the behavior of pigs present on Hawaiian Islands today has shifted away from the human‐dependence of the early Polynesian pigs to feral, self‐sustaining populations. Although the earliest accounts of feral pigs vary by island (Table 1), today feral populations are found on the Island of Hawai‘i, Maui, Moloka‘i, O‘ahu, Kaua‘i, and Niihau (privately owned and not included in this study). Importantly, pigs are no longer reliant on anthropogenic food sources for survival and the abundant foraging resources of introduced non‐native species, such as fruiting plants like strawberry guava ( *Psidium cattleianum* ) and non‐native earthworms (*Lumbricina* spp.), have supported the expansion of feral pig distributions and increases in abundance (Cuddihy and Stone 1990; Wehr et al. 2018; Hess et al. 2019; Peyton et al. 2024). There has also been a shift in the social importance of pigs, away from animal husbandry to a hunter–game relationship with an increase in recreational and subsistence hunting (Hess et al. 2019; Luat‐Hūʻeu et al. 2023). Pigs are the most popular wild game species across the Hawaiian Islands and feral pig harvest is relied on for both subsistence and celebrating major life events like weddings and funerals (Kramer 1971; Duffy 2010; Lohr et al. 2014).

**TABLE 1:** Earliest accounts found mentioning the presence of feral pigs across the Hawaiian Islands represented in this study. Unpublished results of a literature review of online books, travelogues, Hawaiian newspapers, and other documents available online or, when necessary, in printed books by N.B. searching for early accounts of feral pigs in Hawaii. Search terms: *pig, hog, puaa, boar, sow, pork, swine, animal, quadruped, wild, livestock, animal, beast, fur, meat,* and *hunt* yielded many early accounts of feral pigs across Hawaii. The earliest date and reference for each island are reported.

Island	Year and Location	Average Southeast Asian Ancestry (range)	References
O‘ahu	c.1837 Honolulu and Kailua	29.4% (14.0%–40.0%^a^)	Bishop and Thurston (1916)
Kaua‘i	1840 Koke‘e	24.8% (13.7%–34.2%^a^)	Wilkes (1849)
Hawai‘i	1851 Kalaeha	18.1% (9.0%–31.3%)	Maly and Maly (2005)
Moloka‘i	1873 Kalaupapa	14.7% (10.0%–19.9%)	Korn (1976)
Maui	1919 Kula Maui	14.7% (9.1%–19.0%)	Anonymous (1919)

^a^ Ancestry ranges shown for O‘ahu and Kaua‘i exclude data from one domestic Pietrain individual each which, if included, decreases the lower range value to < 0.5%.

These altered relationships play a role in the damage that modern feral pigs inflict. They are considered the single most detrimental invasive alien species in Hawai‘i (Staples and Cowie 2001) and their destructive effects on fragile, endemic ecosystems of the Hawaiian Islands have been well‐documented (e.g., Hess et al. 2019; Nogueira‐Filho et al. 2009; Wehr et al. 2018). The impacts of feral pigs include omnivory, rooting, trampling, and dissemination of invasive plant seeds (Diong 1982). Further, their creation of nutrient‐rich wallows supports invasive mosquitoes ( *Culex quinquefasciatus* ) that carry avian malaria and has contributed to the extinction of multiple endemic Hawaiian bird species (LaPointe 2006). Additionally, in the last decade, Rapid ‘Ōhiʻa Death (ROD) has killed millions of ʻōhiʻa lehua ( *Metrosideros polymorpha* ), a keystone tree species, jeopardizing forest health, watersheds, and Hawaiian cultural practices that rely on ʻōhiʻa (Leopold and Hess 2017; Wehr et al. 2018; Fortini et al. 2019; Perroy et al. 2021; Potter et al. 2023; Roy et al. 2024). While there are multiple environmental reservoirs for the fungal pathogens that cause ROD, increasing evidence implicates abundant populations of feral pigs as important mechanical vectors in transmission (Perroy et al. 2021). Feral pigs are also reservoirs for diseases such as brucellosis (La et al. 2024), trichinosis (Barrett‐Connor et al. 1976), bovine tuberculosis (Essey et al. 1983), and leptospirosis (Buchholz et al. 2016) and present risks for emerging foreign animal diseases such as African Swine Fever (Brown et al. 2023). Contemporary feral pigs also impose economic costs associated with extensive agricultural damage, livestock depredation (Shwiff et al. 2025), and the highest rate of vehicle strikes per distance driven in the United States (McKee et al. 2024).

As with many non‐native species that have cultural value (e.g., feral horses [*Equis ferus*] in the continental United States [Petersen et al. 2023] or black locust [ *Robinia pseudoacacia* ] in Hungary [Jarić et al. 2025]), there is a clear wildlife management conundrum with feral pigs in Hawai‘i. They present a case in which one culturally important species is threatening and damaging to many other culturally, economically, and biologically important natural resources. Furthermore, approximately 90% of terrestrial species on Hawai‘i are endemic, which makes the ecosystem a priority for conservation (Allison 2003). Given that invasive species are recognized as among the most critical threats to the persistence of endemic species and ecosystems (Bellard et al. 2016), the State of Hawai‘i has a dual mandate to preserve and protect traditional and customary practices, such as hunting (Hawai‘i Const. art. XII § VII), while simultaneously controlling the detrimental effects of this invasive species (Luat‐Hū'eu et al. 2021; DOFAW 2025a).

The origins and ancestry of contemporary Hawaiian feral pigs have been previously studied and have drawn mixed conclusions. Some researchers have suggested that ancient land use and animal husbandry practices minimized feralization (Luat‐Hūʻeu et al. 2023) or that the Polynesian breed was not prone to feralization until after European contact (Mueller‐Dombois et al. 1981; Diong 1982). Tomich (1986) proposed that the effect of introgression from European lineages has largely displaced ancestry from the Polynesian (Asian) pig, a suggestion that decouples the animals from Native Hawaiian cultural practices and makes eradication efforts less culturally sensitive. Others have identified a distinct genetic signal in contemporary animals associated with Asian lineage pigs and therefore suggest that they are worthy of conservation as a distinct genetic resource (Larson et al. 2005, 2007; McCann et al. 2014; Linderholm et al. 2016; Faria et al. 2019). Our current study used a high‐resolution, genome‐wide approach and a larger sample size than previously analyzed with the goal of characterizing the ancestry of contemporary feral pigs in Hawai‘i to assess hybridization of Polynesian pigs with global Asian and European lineages. If the hypothesis that contemporary Hawaiian feral pigs are a unique and isolated population, primarily of Polynesian descent, is supported, then we would expect to find high ancestry values associated with Asian lineages and distinct runs of homozygosity (ROH) resulting from a history of genetic drift and local adaptation. Alternatively, if contemporary populations demonstrate hybrid ancestry of primarily European breeds and limited ROH that have been disrupted by gene flow from distinct ancestral sources, then it would suggest that they are no longer an isolated Polynesian lineage.

## 2 Materials and Methods

### 2.1 *Sus scrofa* Reference Set Assembly

To investigate the genetic ancestry of Hawaiian feral pigs, we began by applying the methods of Smyser et al. (2020) in which the authors assembled a comprehensive *S. scrofa* reference set spanning the global wild–domestic species complex, including domestic breeds, wild boar, and sister taxa, from which they could query feral pigs of unknown origin. In this study, we began with the previously published reference set compiled in Smyser et al. (2020), including contributions from Burgos‐Paz et al. (2013), Goedbloed et al. (2013), Roberts and Lamberson (2015), Iacolina et al. (2016), Alexandri et al. (2017), and Yang et al. (2017). We supplemented this reference set with additional high‐density single nucleotide polymorphism (SNP) genotypes from India (Mehrotra et al. 2021) and China (Ai et al. 2013) as well as additional heritage pig genotypes (Smyser et al. 2024). We restricted our reference set to samples genotyped using Illumina BeadArray chemistry (Illumina PorcineSNP60 BeadChip [versions 1 and 2] or Genomic Profiler for Porcine HD microarrays [Ramos et al. 2009]). We followed the same quality control measures outlined in Smyser et al. (2020) to ensure that inferences made from additional genotypes were robust. Briefly, we estimated pairwise identity by descent (IBD) to exclude duplicate genotypes and remove samples from closely related dyads (IBD ≥ 0.70) and conducted iterative supervised runs with ADMIXTURE (version 1.3.0; Alexander et al. 2009) to confirm a strong association with the combined, genetically similar reference groups. Hereafter, we use the term “reference group” to refer to groups of reference samples organized by breed for domestic pigs or country of origin for native wild boar, “reference cluster” to represent genetically similar reference groups consolidated into genetic clusters, and “reference set” to denote the collection of all reference samples. Our final reference set was comprised of 2707 samples organized into 23 genetically cohesive reference clusters representing 2200 commercial or heritage breeds of domestic pig, 479 wild boar from populations sampled throughout their native range, and 28 samples from four sister taxa. Combining multiple SNP datasets produced across multiple Illumina BeadArrays yielded 27,467 shared autosomal SNP loci available for analysis.

Previous research suggests that Hawaiian feral pig populations have a mixture of mainland Asian (East Asian, and potentially Southeast Asian) and European origins (Larson et al. 2007; Linderholm et al. 2016; Faria et al. 2019). Therefore, we closely examined the reference clusters from Smyser et al. (2020) that were comprised of Meishan and related breeds and Asian wild boar populations and European Heritage breeds for subdivisions that could help clarify ancestral signals from these parts of the world. First, given the lingering controversy about the origins of Polynesian pigs (Larson et al. 2007; Yang et al. 2011; Faria et al. 2019; Horsburgh et al. 2022) and our interest in historical Polynesian introduction to the Hawaiian Islands, we were particularly interested in the contributions from Asian lineages in Smyser et al. (2020) and the newly incorporated Chinese breeds (Ai et al. 2013). As described in Smyser et al. (2020), we sought to combine genetically similar reference groups into reference clusters while maintaining the statistical power to identify associations of feral pigs to the reference cluster. Therefore, we partitioned these samples into subpopulations based on their spatial origin and an unsupervised ADMIXTURE clustering test. The unsupervised ADMIXTURE analysis allowed each reference sample to have a mixture of clusters. We evaluated clustering of these reference groups from *K*2–40 using the cross‐validation output from ADMIXTURE and visualization of the spatial clustering and breed cohesion in a neighbor‐joining tree (Huson and Bryant 2006) to identify the most informative value of *K*. Because we predicted that greater subdivision of these Asian breeds would be informative for Hawaiian pigs, we selected the highest clustering results feasible to delineate potentially meaningful reference groups (*K* = 5; Table 2 *K*_16–20_). In the absence of a validated Polynesian pig to include in our reference set, we categorized the domestic and wild boar samples from Southeast Asia (*K*_20_; i.e., southeast China and Thailand), which likely represent the ancestral origins for Polynesian voyagers who settled the Hawaiian Islands (Allen et al. 2001; Horsburgh et al. 2022), as the most likely reference for Polynesian pigs and hereafter refer to this reference group as the “Southeast Asian” cluster (*K*_20_). The four remaining subclusters represent samples from across eastern China (“Jinhua” [*K*_16_] and “Eastern China” [*K*_18_] clusters), western China (“Western China” [*K*_19_]), and representative breeds sampled across western, central, and northern China, South Korea, and Russia (“Other Asian” [*K*_17_]; Table S2). Second, given that Captain Cook initially departed from England, we hypothesized that there may be stronger signals of heritage breeds from northern Europe as opposed to southern Europe. To closely investigate the European origins of Hawaiian pigs, we incorporated the additional heritage pig genotypes (Smyser et al. 2024) into the European Heritage breeds cluster from Smyser et al. (2020) and delineated this group into Southern and Northern European regions of origin (i.e., “S. European Heritage” and “N. European Heritage” clusters [Table 2 *K*_22_ and *K*_21_, respectively]). We confirmed that these southern and northern regions were genetically distinct using an unsupervised ADMIXTURE clustering test (*K* = 2). After this thorough re‐evaluation of reference genotypes, our Hawaii‐specific reference set was organized into 23 genetically cohesive reference clusters (Table 2).

**TABLE 2:** Comprehensive *Sus scrofa* reference set for the description of genetic ancestry of Hawaiian feral pigs organized into 23 genetically cohesive reference clusters. Reference groups are reference samples organized by domestic breed, country of origin for native wild boar, and sister taxa. Sample sizes and references for the publicly available genotypes are also shown.

Reference Cluster	Sample Size	Reference Groups	Reference Type	References
K1	80	Berkshire	Domestic pig	Iacolina et al. (2016), Yang et al. (2017), Smyser et al. (2020)
K2	68	Hampshire	Domestic pig	Burgos‐Paz et al. (2013), Yang et al. (2017), Smyser et al. (2020)
K3	28	Sister Taxa ( *Sus verrucosus* , *Phacochoerus africanus* , *Sus celebensis* , *Babyrousa babyrussa* )	Sister Taxa	Yang et al. (2017)
K4	11	Ghurrah	Domestic pig	Mehrotra et al. (2021)
K5	92	Wild Boar Sardinia	Native wild boar	Iacolina et al. (2016)
K6	25	British Saddleback	Domestic pig	Goedbloed et al. (2013), Yang et al. (2017)
K7	66	Pietrain	Domestic pig	Goedbloed et al. (2013), Iacolina et al. (2016), Yang et al. (2017)
K8	241	Chester White and Other (Middle White, Breitov, Pulawska Spot, Bisaro, Mirgorod Swine, Poltava Swine, Prestice, Bunte Bentheimer, Livni, Urzhum, Angler Sattleschwein, Murom, Spotted Steppe, Kenya1, Kenya2, Canarian, Red White Belted, Pork Swine)	Domestic pig	Burgos‐Paz et al. (2013), Iacolina et al. (2016), Yang et al. (2017), Smyser et al. (2020)
K9	182	Duroc and Other (Hereford, Red Wattle)	Domestic pig	Burgos‐Paz et al. (2013), Goedbloed et al. (2013), Roberts and Lamberson (2015), Iacolina et al. (2016), Yang et al. (2017), Smyser et al. (2020)
K10	175	Landrace and Other (Welsh, Linderoth, British Lop, Pork Swine)	Domestic pig	Burgos‐Paz et al. (2013), Goedbloed et al. (2013), Yang et al. (2017), Smyser et al. (2020)
K11	14	Miniature Siberian	Domestic pig	Yang et al. (2017)
K12	41	Minzhu and Leanhua	Domestic pig	Yang et al. (2017), Smyser et al. (2020)
K13	19	Sutai and Lichahei	Domestic pig	Yang et al. (2017)
K14	191	Yorkshires and Large White	Domestic pig	Burgos‐Paz et al. (2013), Goedbloed et al. (2013), Iacolina et al. (2016), Yang et al. (2017), Smyser et al. (2020)
K15	47	Wild Boar Japan	Native wild boar	Smyser et al. (2020)
K16	29	Jinhua	Domestic pig	Ai et al. (2013), Yang et al. (2017)
K17^a^	269	Other Asian (Diqing Zang, Gansu Zang, Ganxiliangtouwu, Leping Spotted, Litang Zang, Mingguangxiaoer, Milin Zang, Neijiang, Rongchang, Shaziling, Tongcheng, Wild Boar (China), Wild Boar (Russia), Wild Boar (Korea), Gongbujiangda Zang, Tibetan)	Domestic pig (211); Native wild boar (58)	Ai et al. (2013), Yang et al. (2017)
K18	133	Eastern China (Meishan, Fengjing, Erhualian, Jiangquhai, Wannan Spotted)	Domestic pig	Ai et al. (2013), Burgos‐Paz et al. (2013), Yang et al. (2017), Smyser et al. (2020)
K19	56	Western China (Bamei, Guanling, Hetaodaer, Laiwuhei)	Domestic pig	Yang et al. (2017)
K20^a^	140	Southeast Asian (Bamaxiang, Congjiangxiang, Diannanxiaoer, Dongshan, Guangdongdahuabai, Jhom Thong Chiang Mai, Lantang, Leping Spotted, Luchuan, Om Koi Chiang Mai, Wild Boar (Thailand), Wuzhishan, Xiang)	Domestic pig (136); Native wild boar (4)	Yang et al. (2017)
K21	270	N. European Heritage (Red Wattle, Guinea Hog, Tamworth, Mulefoot, Gloucester Old Spot, Large Black, Ossabaw, Spotted, Choctaw, Leicoma, Monteiro, Moura, Piau, Cuino, Hairless, Red White Belted, Poland China)	Domestic pig	Burgos‐Paz et al. (2013), Goedbloed et al. (2013), Roberts and Lamberson (2015), Yang et al. (2017), Smyser et al. (2020), Smyser et al. (2024)
K22	252	S. European Heritage (Black Slavonian, Mora Romagnola, Casertana, Calabrese, Iberian, Mangalitsa, Turopolje, Mangalica, Cinta Senese, Nera Siciliana, Yucatan, Manchado de Jabugo, Sicilian)	Domestic pig	Burgos‐Paz et al. (2013), Iacolina et al. (2016), Yang et al. (2017), Smyser et al. (2020), Smyser et al. (2024)
K23	278	European Wild Boar (Greece, Netherlands, Spain, France, Italy, Russia, Croatia, Slovenia, Germany, Portugal, Poland, Tunisia, Bulgaria, Greece Samos, Luxembourg, Finland, Sweden)	Native wild boar	Burgos‐Paz et al. (2013), Goedbloed et al. (2013), Iacolina et al. (2016), Alexandri et al. (2017), Yang et al. (2017), Smyser et al. (2020)

^a^ We acknowledge that the SNP tool we use, although attempting to be a tool for application across all pigs, has some ascertainment to “favor” western animals. Accordingly, we have Asian wild boar grouped with Asian domestic pigs. Although this is a shortcoming of the analysis, it is reflective of the resolution of the tool that is currently available.

### 2.2 Sample Collection and Genotyping

**FIGURE 1:**
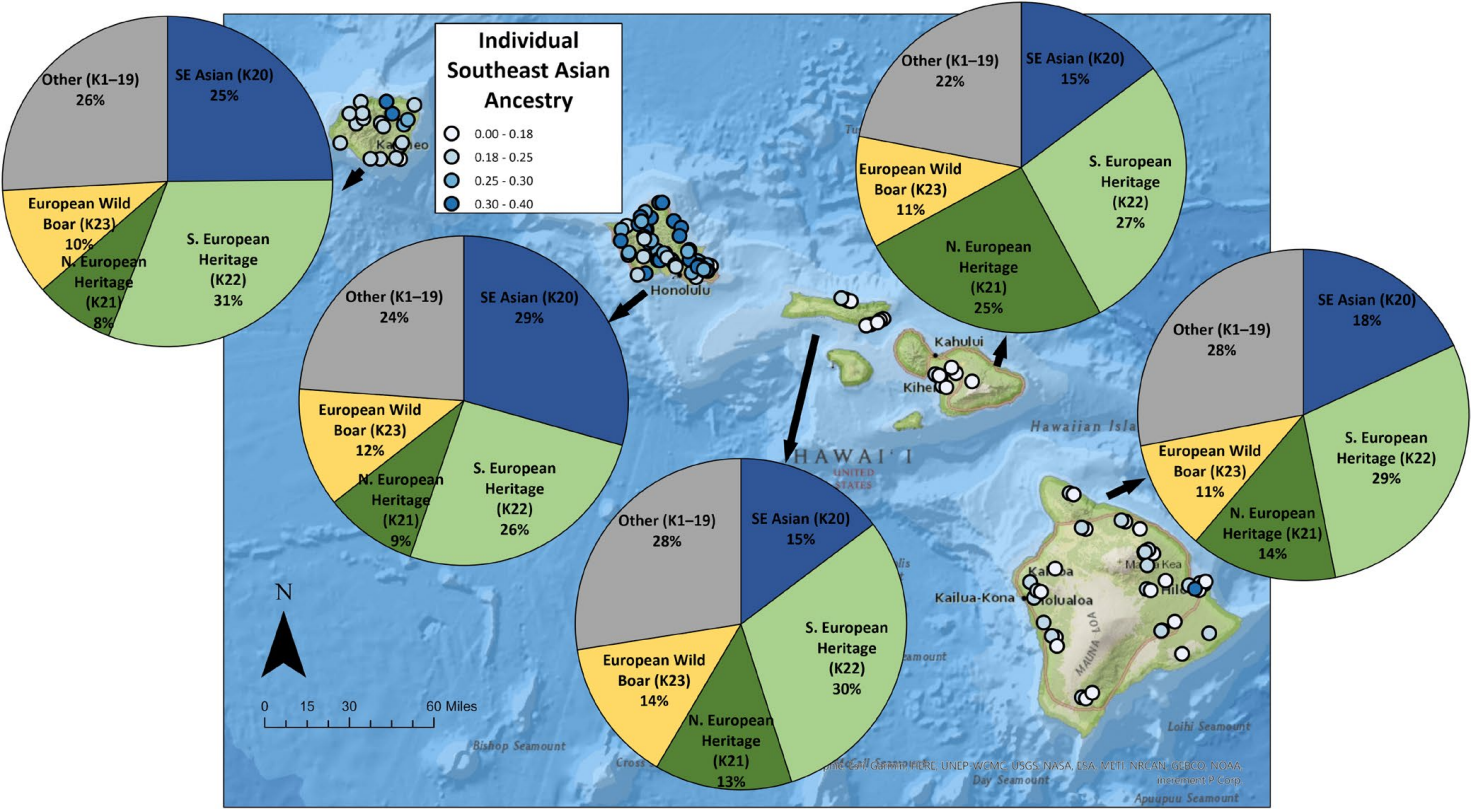
Genetic ancestry of 608 Hawaiian feral pigs. Tissues were collected from June 12, 2012 to February 25, 2022 across the five Hawaiian Islands with contemporary feral pig presence: Island of Hawai‘i (*n* = 111), Maui (*n* = 79), Moloka‘i (*n* = 24), O‘ahu (*n* = 321), and Kaua‘i (*n* = 73). The percent of Southeast Asian ancestry are depicted at the sampling locations for the 608 samples included in genetic analyses, with darker shades reflect higher contributions; coordinates were not available for 19 samples (Kauai = 12, Moloka‘i = 7), therefore ancestry data for those samples are not displayed as points on the map. The symbology gradient breaks are determined by quantiles, representing the same frequency of samples per class. Pie charts depict the average genetic ancestry values by island as calculated from iterative runs of ADMIXTURE in a supervised framework. Although average ancestry contributions are broadly the same across islands, the variation within islands is large. The largest contributions of Southeast Asian ancestry were found on O‘ahu and Kaua‘i followed by the Island of Hawai‘i, Maui, and Moloka‘i, in descending order.

We obtained 672 tissues (i.e., pinna, tongue, and hair) collected across five Hawaiian Islands from 12 June 2012 to 25 February 2022 (Figure 1). The majority of tissue samples were opportunistically collected from euthanized pigs that were removed during population control, damage mitigation, or disease surveillance efforts conducted by the United States Department of Agriculture (USDA), Animal and Plant Health Inspection Service (APHIS), Wildlife Services (WS), and National Feral Swine Damage Management Program (NFSDMP). Additional hunter‐harvested samples were provided by The Nature Conservancy and the USDA Forest Service Pacific Southwest Institute of Pacific Islands Forestry. Metadata, including general sampling locality and collection date, were recorded. Given that genetic samples were acquired during legally authorized control or harvest of feral pigs, sample collection was exempted from Institutional Animal Care and Use Committee review.

We extracted genomic DNA with a commercially available magnetic bead recovery kit (MagMax, Thermo Fisher Scientific, Waltham, MA, USA). We genotyped samples using the GeneSeek Genomic Profiler for Porcine HD, a commercially available genotyping array exclusively licensed to GeneSeek, a Neogen Corporation, with 62,128 biallelic, autosomal SNPs mapped to the Sscrofa11.1 genome assembly (Ramos et al. 2009; Warr et al. 2020). We acknowledge that this SNP array, although attempting to be a tool for application across all pigs, has some ascertainment bias to favor western animals (Ramos et al. 2009). Although this is a shortcoming of the analysis, it is reflective of the resolution of the tool that is currently available, and we believe it meets the needs of the current study as the ascertainment bias would contribute to lower resolution among Asian lineages as opposed to the difficulty differentiating Asian and European lineages. Finally, we conducted a series of quality control steps. We pruned the marker set to match that of our reference set described above, and we pruned the data to exclude individuals with more than 5% missing genotypes. Following these quality control steps, we retained 608 samples at 27,467 markers to analyze with our ancestry panel.

### 2.3 Quantification of Genetic Ancestry

We conducted iterative runs of ADMIXTURE in a supervised framework to query Hawaiian feral pigs relative to the 23 reference clusters. Specifically, we used R (version 4.1.1; R Core Team 2019) to organize input files, iteratively combine feral pig data with the reference set in PLINK (Purcell et al. 2007), pass files to ADMIXTURE, and compile output files. To infer genetic ancestry, we queried a single feral pig sample against the 23 reference clusters using 100 bootstrap iterations, resampling across loci, to obtain the relative contribution of reference clusters to the ancestry of each queried individual (*Q*‐matrix) and associated standard error (Libiger and Schork 2013). We queried a single feral pig sample per iteration to minimize the variation in reference cluster allele frequencies caused by ADMIXTURE's utilization of both the reference cluster and the sample(s) being analyzed when conducting supervised analyses (Alexander et al. 2009; Bansal and Libiger 2015). We calculated average ancestry estimates by island to identify patterns that may relate to Asian or European introduction histories.

Given our interest in directly comparing Asian versus European ancestry in the Hawaiian feral pig samples, we conducted an additional analysis using ADMIXTURE in an unsupervised framework. The unsupervised framework permitted us to partition Asian and European lineages (*K* = 2) while allowing reference samples to demonstrate some level of introgression between lineages (i.e., crossing Asian and European lines in the establishment of contemporary commercial breeds; Bosse et al. 2014). We iteratively queried feral pig samples against the reference dataset in this unsupervised ADMIXTURE analysis, which clarified individual patterns of admixture.

We also conducted a PCA to visualize the genetic ancestry of Hawaiian feral pigs relative to those in our reference set. As a dimension‐reduction analysis method unrestricted by a genetic model, PCA has been combined with other statistical approaches as an effective clustering method to identify spatial population structure (Patterson et al. 2006). Following the approach of Smyser et al. (2020), we used ADEGENET (Jombart 2008) to conduct a PCA of the 1149 reference samples composed of Asian breeds (*K*_16–20_) and European Heritage animals (*K*_21–22_). We subsequently projected the Hawaiian feral pig samples along the PC axes defined by the reference set by applying the linear combination of component loadings derived from the reference set to the allele composition of the Hawaiian feral pig genotypes (McVean 2009). By projecting the Hawaiian feral pig samples along the PCs defined by the reference set, we were able to visualize the relationships of genetic diversity without the Hawaiian feral pigs dictating the axes (McVean 2009).

### 2.4 Measures of Genetic Diversity

We assessed genotypes for the presence of long ROH using PLINK to further describe differences in samples and to evaluate if contemporary Hawaiian pigs plausibly originated from a small founder population descending directly from initial Polynesian introductions. Following the methods of Meyermans et al. (2020) and Barmentlo et al. (2024), we filtered for SNP call rates (≥ 95%) and individual call rates (≥ 90%). We restricted the characterization of ROHs to regions with a minimum density of 1 SNP per 80 kb, a maximum gap size of 600 kb, and excluded regions with > 1 heterozygous loci. We used the size of ROH segments to calculate the average fraction of ROH (fROH) of the total autosomal genetic material per sample (2,265,775 kb; NCBI Genome assembly Sscrofa11.1). fROH values were compared to reference clusters of Asian (*n* = 626) and European Heritage (*n* = 522) breeds. For simplicity, we used the 27,467‐marker set, described above, to calculate ROH for both Hawaiian and reference cluster samples. Given that Bosse et al. (2012) demonstrated that approximately 60,000 markers provide a more reliable characterization of ROH within the *S. scrofa* genome, we validated these results with a reduced number of samples for which we had a larger marker set (i.e., 62,128 SNPs, see Appendix S1). If contemporary Hawaiian pigs were of purely Polynesian ancestry, we would expect to find higher fROH values and longer ROH segments than the reference clusters, indicating inbreeding, bottlenecks, and founder effects.

## 3 Results

The average ancestry estimates of all Hawaiian feral pigs showed a clear signal of admixture with statistically significant associations to more than one reference cluster (i.e., assigned to reference clusters for which the standard error of proportional ancestry does not overlap 0; Table S1). Hawaiian samples were most strongly associated with two European Heritage reference clusters (*K*_22_ and *K*_21_; statistically significant association for 608/608 and 511/608 Hawaiian feral pigs, respectively). The combined average ancestry attributed to Southern European and Northern European domestic reference clusters was 39.6% (${\bar{Q}}_{22}$ = 27.4% and ${\bar{Q}}_{21}$ = 12.2%, respectively where ${\bar{Q}}_{i}$ represents the mean association for reference cluster *i* across all feral pigs). European Wild Boar contributed another 11.4% in ancestry composition (605/608), thus overall European ancestry accounts for 51.0% of the genetic signal. The average ancestry of Hawaiian pigs attributed to the Southeast Asian cluster (*K*_20_) was 24.3% (607/608). The remaining 19 reference clusters cumulatively contributed 24.8% to the average ancestry estimate, nine of which had almost no genetic association (${\bar{Q}}_{i}$ < 1%; Table S1). We corroborated the influence of higher European than Asian ancestry on the Hawaiian populations with an unsupervised ADMIXTURE analysis with *K* = 2 (${\bar{Q}}_{\text{European}}$ = 60.0%, ${\bar{Q}}_{\text{Asian}}$ = 40.0%). European ancestry values for individuals ranged from 50% to 77%, whereas values assigned to Asian ancestry ranged from 23% to 50% (European and Asian ancestry values for each individual sum to 1 in this analysis).

**FIGURE 2:**
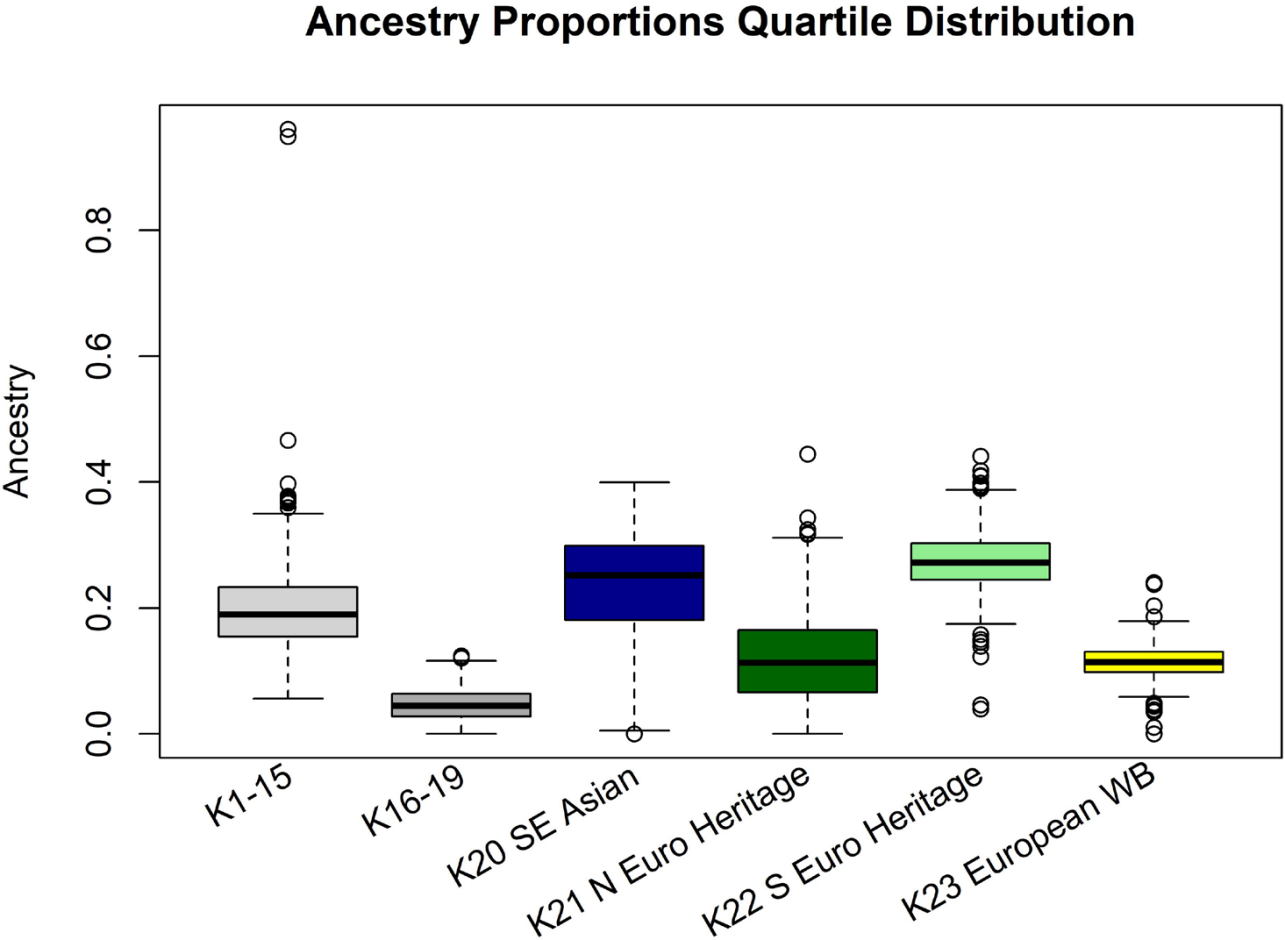
Genetic ancestry distribution across 23 reference clusters. Boxplots depict the quartiles of ancestry values assigned to 608 Hawaiian feral pig samples collected from June 12, 2012 to February 25, 2022 across the five Hawaiian Islands with contemporary feral pig presence: Island of Hawai‘i (*n* = 111), Maui (*n* = 79), Moloka‘i (*n* = 24), O‘ahu (*n* = 321), and Kaua‘i (*n* = 73). For simplicity, ancestry contributions are combined into single boxplots for reference clusters *K*_1–15_ (i.e., Berkshire, Hampshire, Sister Taxa, Ghurra, Wild Boar Sardinia, British Saddleback, Pietrain, Chester White and Other, Duroc and Other, Landrace and Other, Miniature Siberian, Minzhu and Lichahei, Yorkshire and Large White, and Wild Boar Japan) and *K*_16–19_ (Jinhua, Other Asian, Eastern China, and Western China). The remaining reference clusters (Southeast Asian [*K*_20_], Northern European Heritage [*K*_21_], Southern European Heritage [*K*_22_], and European Wild Boar [*K*_23_]) are depicted individually for clarity. Interestingly, the ancestry patterns of the Southeast Asian reference cluster (*K*_20_) did not demonstrate a significant divergence from the other reference clusters. Genetic association to the Southeast Asian cluster (*K*_20_) ranged from < 0.001 to 39.974% (25% quartile = 18.1%, 50% quartile = 25.2%, 75% quartile = 29.9%). Outliers are represented with black circles.

We found similar patterns of admixture at the individual level as well. Hawaiian feral pigs were highly admixed with 100% of samples demonstrating statistically significant associations to multiple reference clusters. Ancestry associations for only two samples were attributed to distinct genetic sources, with both having associations of > 93% to *K*_7_ (Pietrain breed; Table S1). Ancestry associations for all other animals were strongly admixed, with the maximum associations to a single cluster of < 45%. Genetic association to the Southeast Asian cluster (*K*_20_) ranged from 9.0% to 40.0% (excluding the two individuals with domestic Pietrain ancestry mentioned above; Table 1, Figure 1 and Figure 2), whereas genetic association to European clusters (*K*_21+22_) ranged from 18.2% to 59.6% (excluding the two Pietrain individuals; Table 1).

Although admixture of Asian and European ancestry was identified in almost all individuals, there were differences in ancestry proportions between islands. Feral pig samples from O‘ahu and Kaua‘i showed the highest associations to the Southeast Asian cluster (*K*_20_ averaging 29.3% and 24.8%, respectively), whereas Moloka‘i and Maui had the least (14.7% each). The Island of Hawai‘i had intermediate association to *K*_20_ (18.1%; Figure 1). The associations to European Heritage clusters (*K*_21_ and *K*_22_ combined) demonstrated an inverse pattern, with the highest average ancestry values found on Maui and decreasing in value on Moloka‘i, Island of Hawai‘i, Kaua‘i, and O‘ahu (52.4%, 43.8%, 43.1%, 38.8%, and 35.1%, respectively).

**FIGURE 3:**
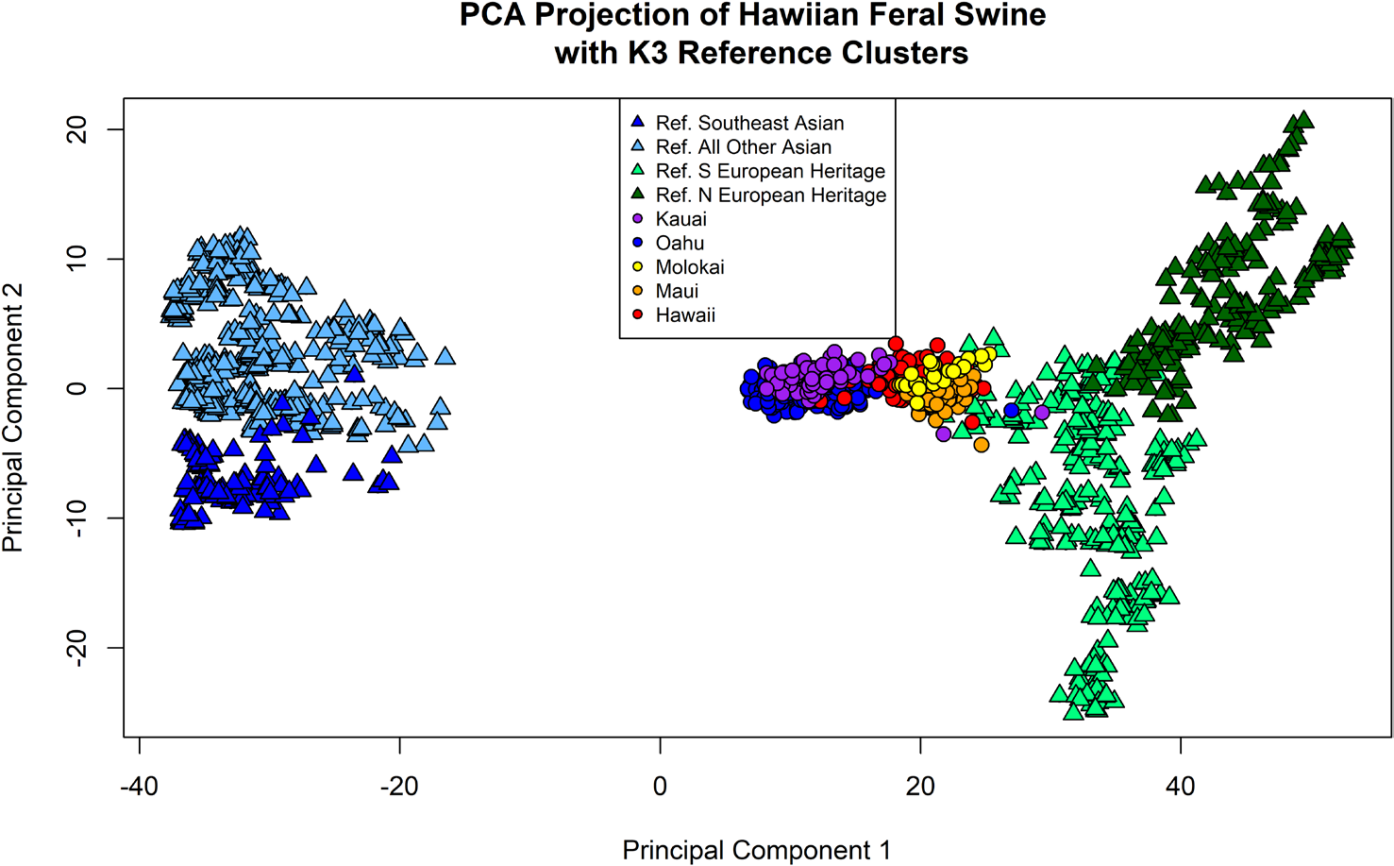
Principal components analysis (PCA) of Hawaiian feral pig and reference clusters. We defined the principal component (PC) axes by reference clusters of primary interest (e.g., Asian [*K*_16–20_] and European Heritage [*K*_21_ and *K*_22_]), represented by triangles. We then applied the linear combination of component loadings derived from the reference set to the allele composition of the Hawaiian feral pig samples to project feral pig samples along the PCs. Hawaiian feral pig samples are represented by circles with each island depicted in a different color. PC1 separates the Asian breeds from the European Heritage reference sets. Hawaiian feral pig samples are projected more closely in PCA space to the European reference samples.

We used principal components analysis (PCA) to visualize genetic ancestry of feral pigs relative to the reference set and found that principal component 1 (PC1) separated the reference samples into Asian breeds (*K*_16–20_) and European Heritage animals (*K*_21–22_; Figure 3). PC2 further partitioned the European reference samples into Northern and Southern origins, and the Southeast Asian reference samples from the rest of the Asian breeds. The projected Hawaiian feral pig samples clustered more closely in PCA space to the European Heritage reference samples. We observed some variation by island, with most samples from Kaua‘i and O‘ahu clustering nearest to the Asian lineage reference animals in PCA space.

We further tested the hypothesis that contemporary Hawaiian feral pigs are unique and isolated by searching for patterns of high ROH typically found in isolated island populations (e.g., distinct patterns observed in Japanese wild boar indicating a small or isolated population; Bosse et al. 2012). We evaluated the level of inbreeding of Hawaiian samples compared to reference clusters of Asian (*K*_16–20_) and European Heritage breeds (*K*_21–22_) and found the fROH value from Hawaiian samples (0.06) was between that of Asian (0.05) and European Heritage breeds (0.10). Additionally, the average number of long ROH segments (> 10 Mb) found in Hawaiian samples (11.9) was between that of the reference clusters as well (Asian = 9.7, European Heritage = 17.4). When we doubled the number of loci included in the ROH calculations with a subset of genotypes to validate the smaller marker set, we found similar patterns of mean fROH and long ROH when comparing Hawaiian samples to the reference clusters (Figure S1). Collectively, patterns of ROH demonstrate that feral pig populations in Hawai‘i have similar levels of genetic diversity as genetically managed domestic herds.

## 4 Discussion

Our results demonstrate that contemporary Hawaiian feral pigs are characterized by mixed ancestry that has been shaped largely by contributions from European Heritage breeds and, secondarily, Southeast Asian sources. Contemporary populations may have remnants of initial Polynesian introductions, given the associations to Southeast Asian reference clusters we observed; however, they do not have a genomically distinct signal that we would expect from historic Polynesian pigs. This hybridization has likely mitigated the loss of genetic diversity that would be expected among isolated, island populations from a single introduction. The restoration of genetic diversity through gene flow, as demonstrated by ROH comparable to that observed among genetically managed breeds, could have increased evolutionary potential and allowed contemporary populations to exploit the vastly different ecological landscape than their early Polynesian counterparts encountered (Nogueira‐Filho et al. 2009; Wehr et al. 2018; Hess et al. 2019). We describe the conservation dilemma posed by contemporary admixed populations, which can be highly detrimental to native ecosystems and endemic species, yet remain culturally important to the people of Hawai‘i.

While results from our unsupervised ADMIXTURE analysis, with clustering *K* = 2, showed a substantial proportion of genetic ancestry attributed to Asian origins (${\bar{Q}}_{\text{Asian}}$ = 40.0%) and our full ancestry panel showed significant contributions of Southeast Asian origin in particular (${\bar{Q}}_{20}$ = 24.3%), the association to European Heritage breeds remains the dominant ancestral influence (${\bar{Q}}_{\text{European}}$ = 60.0%; ${\bar{Q}}_{21+22}$ = 39.6% Table S1; Figure 1). Previous studies (i.e., Larson et al. 2007; Linderholm et al. 2016, and Faria et al. 2019) also identified admixed origins of Hawaiian pigs, including European, Asian, and potentially Polynesian ancestry. However, our study demonstrates that the greatest genetic contributions are from European breeds and genetic diversity is similar to that found among domestic breeds. While our work describes some Southeast Asian ancestry, and therefore possible Polynesian genomic elements remaining, in the absence of historical data sources or a Polynesian reference sample, our inference is limited to describing the ancestry of European Heritage pigs as the dominant contribution. Unpublished research by N.B., which analyzed early written accounts of feral pigs in Hawai‘i from the 1700s to 1900s, showed earlier documentation of feral populations correlated with higher average Southeast Asian ancestry (Table 1). This pattern suggests that the longer pigs were in captivity, the more selective breeding was done with introduced breeds in domestic herds before feral populations emerged. The greater representation of European versus Southeast Asian ancestry in our results calls into question previous claims that Hawaiian feral pigs represented a unique, isolated lineage of *S. scrofa* and suggests a history of introductions from various sources and likely multiple admixture events.

A lingering question from our ancestry results is the source of contributions from European wild boar (${\bar{Q}}_{23}$ = 11.4%; Table S1). We have been unable to identify historical accounts describing direct importation of European wild boar to the islands. It is possible that the domestic pigs Europeans introduced to the Pacific Islands possessed modest levels of wild boar ancestry as an outcome of the free‐range husbandry practices of the time (White 2011). Admixture of these European breeds may also explain why we failed to see greater ancestry contributions from northern Europe, where Captain Cook began his voyages, relative to southern European sources. Alternatively, European wild boar ancestry, despite presumed limited initial introductions, could be attributable to fitness advantages conveyed by unique behavioral and morphological characteristics of domestic pig–wild boar hybrids with selection functioning to amplify any historical influences from wild boar (Smyser et al. 2020; Barmentlo et al. 2024).

Presumably, hybrid populations that are dominated by European ancestry represent a different animal than the historic *pua‘a* brought to the islands by Polynesian voyagers. Some researchers have suggested that negative impacts of feral pigs in precolonial Hawai‘i would have been restricted by small populations, limited by food and protein sources (Barret and Stone 1983), or by small body size (Warshauer 1980). Although it is difficult to ascertain whether the expanded niche of invasive animals is attributable to ecological shifts within the islands or the expanded evolutionary potential of hybrid animals, Barmentlo et al. (2024) demonstrated that hybrid wild pigs possess the potential for rapid evolution with genomic recombination occurring at the gene level. Thus, contemporary feral pigs, and their impacts to Hawaiian ecosystems, are not comparable to what they were in the past. These feral animals are no longer commensal with humans, are self‐sustaining in the wild, maintain robust levels of genetic diversity and, concomitantly, adaptive potential. In this context, the admixed ancestry of feral pigs which we observed further threatens the few uninvaded regions of the Hawaiian Islands. Moreover, the hybridization of domestic and wild lineages may lead to gene combinations shaped by natural and artificial selection, which allows them to take advantage of niches in native ecosystems and inflict outsized damage (Fulgione et al. 2016; Barmentlo et al. 2024). The fitness of hybrid contemporary feral pigs in Hawai‘i, the threats to unique ecosystems, coupled with their importance in social systems, leads to a complex management landscape.

Hawaiian feral pigs embody complicated interactions between rare ecosystems, endemic species, and cultural significance—regardless of their ancestral origins. These hybrid feral animals are causing unique ecological and agricultural damage in addition to destroying culturally important resources. Yet, even amidst these considerable threats, there is cultural reverence of feral pigs to Hawaiian people (Luat‐Hū'eu et al. 2021). For example, Luat‐Hū'eu et al. (2021) concluded that Indigenous communities shifted toward hunting of feral pigs in the first half of the 19th century after decades of precolonial existence with pigs as a domestic resource complementing cultural practices. This shift toward hunting was in response to ecological changes associated with the introduction of invasive forage items that supported self‐sustaining wild populations, in addition to the privatization of community land and the collapse of local fisheries (Winter and McClatchey 2008; Luat‐Hū'eu et al. 2021). In the contemporary era, the relationship of Hawaiians with feral pigs represents new cultural traditions.

Managers are therefore faced with a challenging task, to balance cultural legacy and modern reliance on Hawaiian feral pigs by local and Indigenous communities, while also protecting and preserving natural ecosystems and resources threatened by their expansion and persistence. Current management generally relies on two main approaches: facilitating public hunting access (in addition to control and special hunts; Hawaiʻi Administrative Rules DOFAW 2025b) and employing exclusion fencing combined with direct removal via trapping or shooting (e.g., Hess 2016). Feral pigs impact many island stakeholders, not just hunters, including agricultural producers, motorists, tourists, private landowners, tribal members, etc., and therefore stakeholder involvement will need to be inclusive of all interests. Comanagement arrangements—in which power and responsibilities are shared between public resource users and government—advocate for incorporating the perspectives of local hunters into management plans to increase the effectiveness of management actions, reduce conflict, and support cultural practices (Berkes et al. 1991; Winter et al. 2020; Luat‐Hū'eu et al. 2021; Luat‐Hūʻeu et al. 2023). Prioritization of lands needed for conservation and for hunting is already underway for the entirety of Maui Nui with stakeholder engagement (Fortini et al. 2024). Such comanagement philosophies evolve over time, incorporate changing cultures and systems, and do not support the abundance of a single species at the expense of hundreds of others, which may help align feral pig management with cultural and ecological values (Winter and McClatchey 2008; Berkes 2017; Luat‐Hū'eu et al. 2021).

Our study addresses a knowledge gap in the genetic ancestry of feral pigs in Hawai‘i by applying high‐resolution genomic tools to a large sample size across five islands with contemporary feral pig presence. Our results corroborate the findings of previous studies, which have illuminated the hybrid ancestry of contemporary Hawaiian feral pigs, yet we quantified their ancestry as primarily of European origin and rejected the hypothesis that contemporary populations represent a unique, isolated lineage of primarily Southeast Asian origin. In the larger context of invasive species management, we echo the sentiment conveyed by previous researchers that the management of Hawaiian feral pigs will require a strong working relationship among stakeholders and wildlife managers.

## Author Contributions

**Anna M. Mangan:** conceptualization (equal), data curation (equal), formal analysis (equal), investigation (equal), visualization (lead), writing – original draft (lead), writing – review and editing (lead). **Timothy J. Smyser:** conceptualization (equal), data curation (equal), formal analysis (equal), funding acquisition (equal), investigation (equal), methodology (lead), supervision (lead), visualization (supporting), writing – original draft (equal), writing – review and editing (equal). **Nicolai Barca:** conceptualization (supporting), data curation (equal), validation (equal), writing – original draft (supporting), writing – review and editing (supporting). **Steven C. Hess:** conceptualization (supporting), data curation (equal), validation (equal), writing – original draft (supporting), writing – review and editing (supporting). **Kealohanuiopuna M. Kinney:** conceptualization (supporting), data curation (equal), validation (equal), writing – original draft (supporting), writing – review and editing (supporting). **Darrin Phelps:** conceptualization (equal), data curation (equal), validation (equal), writing – original draft (supporting), writing – review and editing (supporting). **Nathaniel H. Wehr:** conceptualization (equal), data curation (equal), validation (equal), writing – original draft (supporting), writing – review and editing (supporting). **Dominic Wright:** conceptualization (equal), data curation (equal), validation (equal), writing – original draft (supporting), writing – review and editing (supporting). **Antoinette J. Piaggio:** conceptualization (equal), data curation (equal), funding acquisition (lead), investigation (supporting), supervision (equal), writing – original draft (supporting), writing – review and editing (supporting).

## Conflicts of Interest

The authors declare no conflicts of interest.

## Supporting information


**Data S1:** ece372822‐sup‐0001‐supinfo.pdf.

## Data Availability

Individual genotype data with corresponding island‐level metadata and scripts are publicly at https://zenodo.org/records/15778474. Coordinates are not provided to protect landowner anonymity.
