# Supplementary material for: Genetic Insights Into Human‐Driven Hybridization, Cultural Shifts, and Ecological Consequences of Feral Pigs (Sus scrofa) in Hawai‘i
Source: Ecol Evol. 2026 Jan 5;16(1):e72822. doi: 10.1002/ece3.72822 (PMC12771612; doi:10.1002/ece3.72822)
Supplement: Supplementary file 1 — Data S1: ece372822‐sup‐0001‐supinfo.pdf. [file ECE3-16-e72822-s001.pdf]

Supplemental Information for:

Genetic insights into human-driven hybridization, cultural shifts, and ecological consequences of feral pigs (*Sus scrofa*) in Hawaiʻi

Anna M. Mangan, Timothy J. Smyser, Nicolai Barca, Steven C. Hess, Kealohanuiopuna M. Kinney, Darrin Phelps, Nathaniel H. Wehr, Dominic Wright, Antoinette J. Piaggio

Table of Contents:

**Supporting Information Text: Runs of homozygosity (ROH)**	Page 1
**Figure S1: Long Runs of Homozygosity**	Page 2
**Table S1: Ancestry *Q*-matrix**	Page 3
**Table S2: Asian Reference Cluster Delineation**	Page 34

Supporting Information Text

Runs of homozygosity (ROH): To validate the fraction of ROH (fROH) calculated with the reference set SNPs (*n* = 27,467) we calculated fROH with a subset of the reference genotypes which had a larger marker set (*n* = 62,128). We used the same parameters outlined in the manuscript (i.e., filtered for SNP call rates >95% and individual call rates >90% and restricted the characterization of ROHs to regions with a minimum density of 1 SNP/80 kb, a maximum gap size of 600 kb, and required complete homozygosity (excluding regions with >1 heterozygous loci)) but the Asian and European Heritage reference cluster breeds had fewer samples (*n* = 39 and 31, respectively). The average fROH value from Hawaiian samples (0.17) was between that of Asian (0.14) and European Heritage breeds (0.18). Similarly, the average number of long ROH segments in Hawaiian samples was not statistically different than the reference clusters (Figure S1) suggesting that Hawaiian samples are not characterized by distinct patterns of inbreeding, bottlenecks, and founder effects that would be expected if they were purely Polynesian pigs.

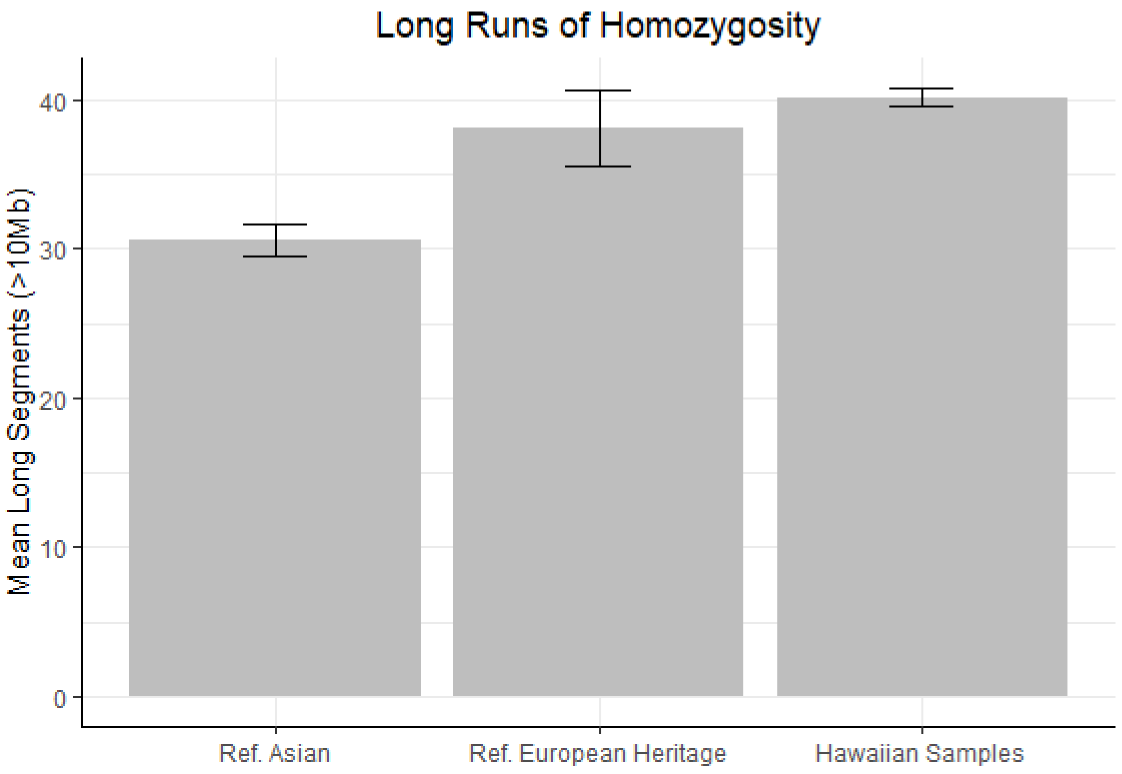

Fig. S1. Long Runs of Homozygosity. The mean number of long runs of homozygosity (ROH), defined as segments greater than 10Mb, for Reference Clusters 16 (Asian; 30.6) and 17 (European Heritage; 38.1) and Hawaiian (40.2) samples.

Table S1. Ancestry *Q*-matrix for feral swine samples to 23 Reference Clusters. The proportion of ancestry and uncertainty (standard error) as determined by 100 bootstrap iterations are presented for each individual (*n* = 608).

Individual ID	Island	Berkshire	Hampshire	Sister Taxa	Ghurrah	Wild Boar Sardinia	British Saddleback	Pietrain	Chester White and Other	Duroc and Other	Landrace and Other	Miniature Siberian	Minzhu and Leanhua	Sutai and Lichahei	Yorkshires and Large White	Wild Boar Japan	Jinhua	Other Asian	Eastern China	Western China	Southeast Asian	N. European Heritage	S. European Heritage	European Wild Boar
Average ($\bar{Q}_i$)	-	0.033	0.018	0.011	0.025	0.013	0.038	0.008	0.007	0.004	0.002	0.017	0.015	0.003	0.002	0.005	0.001	0.034	0.001	0.011	0.243	0.122	0.274	0.114
2019FS004	MOLOKA‘I	0.021 (0.021)	0.048 (0.024)	0.011 (0.007)	0.017 (0.017)	0.026 (0.016)	0.002 (0.02)	0.041 (0.031)	0 (0.014)	0 (0.011)	0 (0.001)	0 (0.008)	0.06 (0.032)	0 (0.018)	0.009 (0.018)	0 (0.003)	0 (0.003)	0.057 (0.03)	0 (0.003)	0 (0.014)	0.137 (0.025)	0.165 (0.066)	0.28 (0.043)	0.126 (0.031)
2020FS009	MOLOKA‘I	0.037 (0.024)	0.038 (0.022)	0.013 (0.007)	0.041 (0.019)	0 (0.009)	0 (0.007)	0.01 (0.022)	0.011 (0.026)	0 (0.015)	0 (0.003)	0 (0.009)	0.005 (0.022)	0.031 (0.029)	0 (0.012)	0 (0.003)	0 (0.004)	0.026 (0.03)	0 (0)	0.046 (0.035)	0.142 (0.026)	0.261 (0.065)	0.206 (0.04)	0.132 (0.021)
2020FS010	MOLOKA‘I	0.039 (0.026)	0.007 (0.016)	0.012 (0.007)	0.018 (0.017)	0.005 (0.011)	0 (0.018)	0 (0.002)	0 (0.006)	0 (0.014)	0.01 (0.024)	0 (0.002)	0 (0.016)	0.045 (0.035)	0.055 (0.028)	0 (0.002)	0 (0.001)	0.025 (0.026)	0 (0.003)	0.024 (0.025)	0.186 (0.027)	0.183 (0.068)	0.257 (0.046)	0.133 (0.027)
2020FS010_A	MOLOKA‘I	0.04 (0.026)	0.011 (0.017)	0.01 (0.007)	0.019 (0.018)	0.006 (0.012)	0 (0.018)	0 (0.002)	0 (0.007)	0 (0.016)	0.015 (0.026)	0 (0.001)	0 (0.019)	0.044 (0.035)	0.051 (0.028)	0 (0.002)	0 (0.001)	0.033 (0.028)	0 (0.002)	0.023 (0.027)	0.18 (0.028)	0.183 (0.069)	0.252 (0.047)	0.133 (0.028)
2020FS011	MOLOKA‘I	0.027 (0.022)	0.029 (0.02)	0.016 (0.007)	0.026 (0.014)	0.012 (0.012)	0.012 (0.022)	0 (0.007)	0 (0.006)	0.033 (0.021)	0 (0.002)	0.016 (0.017)	0 (0.008)	0 (0.01)	0.041 (0.025)	0.001 (0.005)	0 (0.005)	0 (0.008)	0 (0.002)	0.064 (0.022)	0.161 (0.026)	0.142 (0.055)	0.311 (0.041)	0.108 (0.022)
2020FS011_A	MOLOKA‘I	0.026 (0.022)	0.031 (0.021)	0.014 (0.006)	0.025 (0.014)	0.01 (0.012)	0.014 (0.022)	0 (0.007)	0 (0.003)	0.027 (0.02)	0 (0)	0.014 (0.016)	0 (0.007)	0 (0.006)	0.032 (0.024)	0 (0.004)	0 (0.005)	0 (0.011)	0 (0.001)	0.064 (0.023)	0.165 (0.027)	0.147 (0.057)	0.318 (0.041)	0.111 (0.022)
2020FS012	MOLOKA‘I	0.038 (0.025)	0.038 (0.023)	0.013 (0.007)	0.048 (0.02)	0.009 (0.012)	0 (0.011)	0.008 (0.023)	0 (0.026)	0.006 (0.018)	0 (0.009)	0 (0.007)	0.035 (0.027)	0.019 (0.028)	0.022 (0.019)	0 (0.003)	0 (0.004)	0.011 (0.023)	0 (0.001)	0.058 (0.033)	0.138 (0.026)	0.157 (0.066)	0.272 (0.043)	0.128 (0.026)
2020FS012_A	MOLOKA‘I	0.039 (0.025)	0.038 (0.023)	0.012 (0.007)	0.047 (0.02)	0.01 (0.012)	0 (0.012)	0.008 (0.023)	0 (0.027)	0.005 (0.018)	0 (0.01)	0 (0.007)	0.034 (0.027)	0.019 (0.027)	0.024 (0.019)	0 (0.002)	0 (0.004)	0.015 (0.025)	0 (0.001)	0.056 (0.034)	0.138 (0.026)	0.155 (0.066)	0.273 (0.043)	0.128 (0.026)
2020FS013_A	MOLOKA‘I	0 (0.01)	0.073 (0.023)	0.007 (0.005)	0 (0.009)	0.017 (0.014)	0 (0.012)	0.034 (0.026)	0.037 (0.037)	0 (0.009)	0 (0.02)	0 (0.008)	0 (0.012)	0.012 (0.024)	0 (0.014)	0 (0.001)	0 (0.007)	0.006 (0.025)	0 (0.002)	0.085 (0.035)	0.104 (0.026)	0.132 (0.059)	0.251 (0.053)	0.24 (0.032)
2020FS016_A	MOLOKA‘I	0.035 (0.024)	0.04 (0.023)	0.018 (0.007)	0.02 (0.016)	0.003 (0.013)	0.027 (0.026)	0 (0.013)	0 (0.003)	0 (0.003)	0 (0.003)	0 (0.005)	0.01 (0.022)	0 (0.003)	0.011 (0.017)	0 (0.002)	0 (0.001)	0.032 (0.027)	0 (0.008)	0.029 (0.028)	0.15 (0.025)	0.206 (0.062)	0.307 (0.041)	0.112 (0.025)
C1	KAUA‘I	0 (0.006)	0 (0.009)	0.017 (0.009)	0.058 (0.018)	0.032 (0.015)	0.01 (0.017)	0 (0.006)	0 (0.007)	0 (0)	0 (0.018)	0.028 (0.019)	0 (0.005)	0 (0)	0 (0.003)	0.002 (0.006)	0 (0.004)	0.084 (0.031)	0 (0)	0.005 (0.019)	0.294 (0.033)	0.075 (0.041)	0.308 (0.043)	0.086 (0.025)
C10	KAUA‘I	0 (0.008)	0 (0.007)	0.013 (0.007)	0.065 (0.019)	0.013 (0.012)	0.025 (0.02)	0 (0.001)	0 (0)	0 (0.002)	0 (0.004)	0.002 (0.013)	0 (0.011)	0 (0.013)	0 (0)	0.011 (0.009)	0 (0.004)	0.054 (0.026)	0 (0)	0 (0)	0.268 (0.03)	0.163 (0.044)	0.276 (0.038)	0.11 (0.026)
C11	KAUA‘I	0.011 (0.018)	0.007 (0.015)	0.015 (0.007)	0.052 (0.019)	0.011 (0.012)	0.064 (0.027)	0.006 (0.016)	0 (0.001)	0 (0)	0 (0.008)	0.002 (0.013)	0 (0.007)	0 (0)	0 (0)	0.009 (0.008)	0.003 (0.008)	0.032 (0.026)	0 (0.002)	0 (0.002)	0.29 (0.031)	0.118 (0.061)	0.28 (0.043)	0.099 (0.025)
C12	KAUA‘I	0.004 (0.018)	0 (0.012)	0.02 (0.008)	0.056 (0.021)	0.02 (0.013)	0.046 (0.025)	0 (0)	0 (0)	0 (0.003)	0 (0.008)	0 (0.011)	0 (0.015)	0 (0.013)	0 (0)	0.01 (0.009)	0 (0.003)	0.041 (0.028)	0 (0.001)	0 (0)	0.286 (0.034)	0.186 (0.051)	0.244 (0.041)	0.088 (0.03)

C13	KAUA'I	0.02 (0.02)	0 (0.002)	0.01 (0.007)	0.072 (0.019)	0.02 (0.013)	0.039 (0.027)	0 (0.004)	0 (0.018)	0 (0.001)	0 (0)	0.016 (0.017)	0 (0.006)	0 (0)	0 (0.004)	0.027 (0.009)	0 (0)	0.019 (0.021)	0 (0)	0 (0)	0.231 (0.026)	0.02 (0.035)	0.441 (0.037)	0.085 (0.023)
C14	KAUA'I	0.009 (0.015)	0 (0)	0 (0.004)	0.089 (0.016)	0.025 (0.016)	0.026 (0.022)	0.015 (0.017)	0 (0.005)	0 (0.003)	0 (0.003)	0 (0.01)	0.02 (0.023)	0 (0)	0 (0.005)	0.018 (0.008)	0 (0.005)	0.038 (0.026)	0 (0)	0 (0)	0.225 (0.028)	0.114 (0.044)	0.306 (0.038)	0.115 (0.022)
C15	KAUA'I	0.032 (0.024)	0 (0.005)	0.012 (0.008)	0.055 (0.019)	0.015 (0.013)	0.032 (0.022)	0.022 (0.021)	0 (0.008)	0 (0)	0 (0)	0.01 (0.015)	0.041 (0.025)	0 (0.001)	0 (0.007)	0.01 (0.008)	0 (0.003)	0.066 (0.026)	0 (0.004)	0 (0.004)	0.21 (0.027)	0.051 (0.048)	0.318 (0.036)	0.125 (0.021)
C16	KAUA'I	0 (0)	0 (0.005)	0.009 (0.007)	0.041 (0.017)	0.034 (0.017)	0 (0.012)	0 (0.008)	0 (0.006)	0 (0)	0 (0.007)	0.021 (0.016)	0.006 (0.015)	0 (0.016)	0 (0.008)	0.033 (0.008)	0 (0.002)	0.06 (0.025)	0 (0)	0 (0)	0.189 (0.026)	0.074 (0.041)	0.418 (0.047)	0.114 (0.022)
C2	KAUA'I	0 (0.011)	0 (0.006)	0.021 (0.008)	0.032 (0.02)	0.023 (0.014)	0.03 (0.024)	0 (0.006)	0 (0)	0 (0)	0 (0.001)	0.022 (0.019)	0.015 (0.02)	0 (0)	0 (0)	0.008 (0.008)	0 (0.005)	0.044 (0.023)	0 (0.002)	0 (0.001)	0.303 (0.03)	0.126 (0.054)	0.263 (0.045)	0.113 (0.024)
C3	KAUA'I	0 (0.013)	0.018 (0.014)	0.016 (0.008)	0.048 (0.021)	0 (0.009)	0.029 (0.023)	0 (0.008)	0 (0)	0 (0.004)	0 (0)	0.016 (0.016)	0.007 (0.019)	0 (0.009)	0 (0)	0.007 (0.007)	0.004 (0.009)	0.102 (0.028)	0 (0)	0 (0.001)	0.24 (0.026)	0 (0.031)	0.41 (0.037)	0.102 (0.026)
C4	KAUA'I	0.011 (0.018)	0.007 (0.015)	0.015 (0.007)	0.052 (0.019)	0.012 (0.013)	0.064 (0.027)	0.006 (0.016)	0 (0.001)	0 (0)	0 (0.008)	0.002 (0.013)	0 (0.007)	0 (0)	0 (0)	0.009 (0.008)	0.003 (0.008)	0.032 (0.025)	0 (0.001)	0 (0.002)	0.291 (0.031)	0.118 (0.061)	0.28 (0.042)	0.099 (0.025)
C6	KAUA'I	0 (0.008)	0 (0.004)	0.02 (0.009)	0.062 (0.022)	0.01 (0.012)	0.033 (0.022)	0.019 (0.019)	0 (0.031)	0 (0)	0 (0)	0.067 (0.028)	0 (0.008)	0 (0.001)	0 (0.004)	0.01 (0.008)	0 (0.002)	0.051 (0.024)	0 (0.009)	0 (0.006)	0.278 (0.028)	0.025 (0.039)	0.322 (0.037)	0.102 (0.024)
C7	KAUA'I	0 (0.016)	0.005 (0.012)	0.016 (0.007)	0.025 (0.018)	0.022 (0.013)	0.063 (0.029)	0.002 (0.014)	0 (0)	0 (0)	0 (0)	0.008 (0.016)	0 (0)	0 (0)	0 (0)	0 (0.003)	0 (0.003)	0.033 (0.02)	0 (0.004)	0 (0.004)	0.342 (0.028)	0.068 (0.048)	0.328 (0.036)	0.087 (0.025)
C8	KAUA'I	0 (0.009)	0 (0.005)	0 (0.004)	0.081 (0.019)	0.023 (0.015)	0.041 (0.027)	0.021 (0.02)	0 (0.005)	0 (0)	0 (0.008)	0.023 (0.018)	0.006 (0.018)	0 (0.006)	0 (0)	0.012 (0.008)	0 (0.001)	0.027 (0.023)	0 (0)	0 (0.004)	0.307 (0.03)	0.072 (0.05)	0.269 (0.047)	0.118 (0.021)
C9	KAUA'I	0 (0.005)	0 (0.01)	0.02 (0.008)	0.05 (0.02)	0.019 (0.013)	0.019 (0.017)	0.009 (0.018)	0 (0)	0 (0.003)	0 (0.014)	0.046 (0.021)	0 (0.006)	0 (0.009)	0 (0)	0 (0.001)	0 (0.004)	0.064 (0.025)	0 (0)	0 (0.008)	0.289 (0.026)	0.128 (0.047)	0.233 (0.041)	0.123 (0.022)
FP1	KAUA'I	0.009 (0.017)	0.019 (0.018)	0.011 (0.007)	0.05 (0.019)	0.028 (0.015)	0.05 (0.023)	0 (0.005)	0 (0.001)	0 (0)	0 (0.005)	0 (0.01)	0.006 (0.016)	0 (0.005)	0 (0)	0.012 (0.008)	0.003 (0.008)	0.088 (0.027)	0 (0.003)	0 (0.007)	0.208 (0.031)	0.043 (0.044)	0.352 (0.04)	0.121 (0.024)
FP2	KAUA'I	0.019 (0.02)	0 (0.008)	0.008 (0.007)	0.047 (0.019)	0.026 (0.014)	0.035 (0.021)	0.011 (0.017)	0 (0.007)	0 (0)	0 (0.007)	0 (0.012)	0.03 (0.022)	0 (0.015)	0 (0)	0.006 (0.006)	0 (0.003)	0.082 (0.028)	0 (0.002)	0 (0.01)	0.224 (0.031)	0.067 (0.048)	0.349 (0.041)	0.097 (0.021)
FP3	KAUA'I	0.014 (0.019)	0 (0.01)	0.02 (0.008)	0.021 (0.015)	0.003 (0.01)	0 (0.005)	0.002 (0.012)	0 (0)	0 (0.001)	0 (0.002)	0 (0.009)	0.047 (0.027)	0 (0)	0 (0)	0.019 (0.009)	0 (0.002)	0.022 (0.022)	0 (0)	0 (0.001)	0.291 (0.028)	0.104 (0.056)	0.318 (0.047)	0.139 (0.02)
FP4	KAUA'I	0.067 (0.021)	0.023 (0.022)	0 (0.002)	0.047 (0.016)	0 (0.012)	0.001 (0.02)	0.008 (0.018)	0 (0)	0.099 (0.039)	0 (0.001)	0 (0.012)	0 (0.007)	0.017 (0.031)	0 (0.001)	0.015 (0.009)	0.002 (0.006)	0.022 (0.02)	0 (0)	0 (0.004)	0.137 (0.027)	0.307 (0.065)	0.15 (0.047)	0.105 (0.025)
GE0000371	KAUA'I	0.02 (0.02)	0 (0.008)	0.016 (0.008)	0.055 (0.018)	0.017 (0.013)	0.022 (0.02)	0.007 (0.018)	0 (0.002)	0 (0.008)	0 (0.011)	0.023 (0.02)	0 (0.013)	0 (0.001)	0 (0)	0.009 (0.008)	0 (0.001)	0.062 (0.025)	0 (0)	0 (0)	0.254 (0.031)	0.159 (0.051)	0.257 (0.04)	0.098 (0.021)
GE0000372	O'AHU	0.033 (0.023)	0.018 (0.017)	0.002 (0.005)	0.045 (0.021)	0.025 (0.016)	0.034 (0.026)	0 (0.01)	0 (0.012)	0 (0)	0 (0.006)	0.015 (0.018)	0.034 (0.024)	0.049 (0.03)	0 (0.002)	0 (0.005)	0 (0.001)	0 (0.012)	0 (0.001)	0 (0.011)	0.328 (0.028)	0 (0.025)	0.34 (0.035)	0.076 (0.024)
GE0000374	O'AHU	0.012 (0.018)	0.041 (0.022)	0.02 (0.007)	0.008 (0.013)	0.037 (0.015)	0.031 (0.028)	0.008 (0.019)	0.11 (0.06)	0 (0)	0 (0.009)	0.041 (0.02)	0 (0.017)	0 (0.018)	0 (0.012)	0 (0.001)	0 (0.004)	0.021 (0.02)	0 (0.006)	0 (0.017)	0.379 (0.033)	0.024 (0.037)	0.157 (0.04)	0.111 (0.028)
GE0000375	O'AHU	0.014 (0.02)	0.015 (0.017)	0.012 (0.006)	0 (0.006)	0.001 (0.012)	0.047 (0.028)	0.009 (0.014)	0 (0.013)	0 (0.004)	0 (0.017)	0 (0.011)	0.067 (0.031)	0 (0.014)	0.023 (0.024)	0.019 (0.01)	0 (0.001)	0 (0.014)	0 (0.004)	0 (0.001)	0.293 (0.028)	0.086 (0.056)	0.269 (0.042)	0.144 (0.024)
GE0000376	O'AHU	0.014 (0.017)	0.001 (0.013)	0.021 (0.007)	0.016 (0.016)	0.013 (0.012)	0.057 (0.032)	0.011 (0.017)	0 (0.022)	0 (0)	0 (0.004)	0.064 (0.021)	0 (0.006)	0.005 (0.017)	0 (0.01)	0 (0.003)	0 (0)	0.023 (0.027)	0 (0)	0.022 (0.023)	0.356 (0.033)	0.026 (0.033)	0.219 (0.037)	0.154 (0.025)

GE0000377	O‘AHU	0.042 (0.023)	0.022 (0.018)	0.01 (0.005)	0.006 (0.011)	0.022 (0.014)	0.036 (0.022)	0.001 (0.012)	0 (0.016)	0 (0)	0 (0)	0.027 (0.019)	0 (0.006)	0 (0.004)	0.002 (0.014)	0.016 (0.008)	0 (0.002)	0 (0.009)	0.003 (0.01)	0.029 (0.022)	0.347 (0.026)	0.042 (0.04)	0.241 (0.032)	0.154 (0.025)
GE0000378	O‘AHU	0.031 (0.022)	0.032 (0.023)	0.019 (0.008)	0.028 (0.017)	0.011 (0.013)	0.066 (0.025)	0 (0)	0 (0.014)	0 (0.001)	0 (0.014)	0.007 (0.012)	0 (0.011)	0 (0.009)	0 (0)	0.003 (0.006)	0 (0.002)	0.027 (0.019)	0 (0.004)	0 (0)	0.309 (0.026)	0.133 (0.049)	0.244 (0.038)	0.09 (0.024)
GE0000379	O‘AHU	0.058 (0.021)	0.007 (0.011)	0.018 (0.007)	0.027 (0.015)	0 (0.01)	0.022 (0.018)	0 (0.003)	0 (0.026)	0 (0.004)	0 (0.011)	0 (0.011)	0.033 (0.024)	0.014 (0.023)	0 (0)	0 (0.005)	0 (0)	0 (0.01)	0 (0.003)	0 (0.005)	0.33 (0.027)	0.045 (0.038)	0.297 (0.038)	0.149 (0.025)
GE0000380	O‘AHU	0.038 (0.027)	0.035 (0.02)	0.021 (0.007)	0.002 (0.011)	0 (0.008)	0.011 (0.017)	0.005 (0.013)	0 (0.01)	0 (0.008)	0 (0.003)	0.021 (0.019)	0.068 (0.033)	0 (0.006)	0 (0)	0.002 (0.006)	0 (0.003)	0 (0.021)	0 (0.005)	0.025 (0.028)	0.272 (0.032)	0.1 (0.048)	0.239 (0.034)	0.161 (0.025)
GE0000381	O‘AHU	0.031 (0.019)	0 (0.01)	0.016 (0.006)	0.024 (0.016)	0.03 (0.015)	0.006 (0.012)	0 (0)	0 (0.015)	0 (0.002)	0 (0)	0.043 (0.018)	0 (0.014)	0 (0.011)	0 (0.002)	0.007 (0.007)	0 (0)	0.03 (0.025)	0 (0.004)	0.031 (0.023)	0.305 (0.027)	0.035 (0.033)	0.362 (0.036)	0.082 (0.026)
GE0000382	O‘AHU	0 (0.01)	0.007 (0.014)	0.01 (0.007)	0.015 (0.011)	0.033 (0.016)	0.086 (0.024)	0 (0)	0 (0.014)	0 (0)	0 (0.007)	0.041 (0.019)	0.025 (0.024)	0 (0.008)	0 (0.003)	0.001 (0.006)	0 (0.002)	0.037 (0.026)	0 (0.008)	0 (0.001)	0.272 (0.027)	0.132 (0.045)	0.241 (0.046)	0.099 (0.025)
GE0000383	O‘AHU	0.036 (0.026)	0.002 (0.011)	0.01 (0.007)	0.024 (0.014)	0.011 (0.013)	0.07 (0.024)	0 (0.005)	0 (0.01)	0 (0)	0 (0.004)	0 (0.008)	0.063 (0.03)	0.014 (0.024)	0 (0)	0 (0.005)	0 (0)	0.017 (0.022)	0 (0.002)	0 (0.007)	0.293 (0.027)	0.058 (0.053)	0.257 (0.046)	0.144 (0.023)
GE0000384	O‘AHU	0.055 (0.025)	0 (0.005)	0.011 (0.008)	0.054 (0.02)	0.009 (0.012)	0.02 (0.021)	0 (0.01)	0.021 (0.035)	0 (0.003)	0 (0.018)	0.019 (0.018)	0.007 (0.016)	0 (0.014)	0 (0.005)	0.006 (0.008)	0 (0.003)	0.008 (0.014)	0 (0.004)	0.006 (0.017)	0.322 (0.025)	0.088 (0.054)	0.28 (0.046)	0.094 (0.024)
GE0000386	O‘AHU	0 (0.012)	0.018 (0.015)	0.017 (0.007)	0.017 (0.014)	0.016 (0.014)	0.059 (0.022)	0.022 (0.016)	0 (0.027)	0 (0.001)	0 (0)	0.006 (0.012)	0.026 (0.023)	0 (0.012)	0 (0.01)	0.013 (0.008)	0 (0.004)	0.01 (0.021)	0 (0)	0.019 (0.02)	0.297 (0.027)	0.074 (0.048)	0.292 (0.04)	0.114 (0.023)
GE0000387	O‘AHU	0.06 (0.022)	0.013 (0.016)	0.009 (0.006)	0.031 (0.016)	0.019 (0.016)	0.014 (0.014)	0 (0.001)	0.001 (0.028)	0 (0)	0.001 (0.017)	0.014 (0.016)	0.023 (0.022)	0 (0.005)	0 (0.003)	0 (0.001)	0 (0.001)	0.05 (0.029)	0 (0)	0 (0.016)	0.306 (0.03)	0.059 (0.043)	0.286 (0.033)	0.114 (0.025)
GE0000388	O‘AHU	0.026 (0.023)	0.064 (0.019)	0 (0.004)	0.028 (0.018)	0.015 (0.014)	0.077 (0.033)	0 (0.011)	0 (0.024)	0 (0)	0 (0)	0.047 (0.023)	0.071 (0.032)	0 (0.011)	0 (0.011)	0 (0.004)	0 (0)	0.047 (0.033)	0 (0.001)	0 (0.021)	0.203 (0.032)	0.102 (0.052)	0.222 (0.042)	0.098 (0.028)
GE0000390	O‘AHU	0 (0.007)	0.054 (0.018)	0.006 (0.006)	0.034 (0.018)	0.018 (0.014)	0.015 (0.019)	0 (0.006)	0 (0.013)	0 (0)	0 (0.006)	0.047 (0.022)	0 (0.016)	0 (0.01)	0 (0.005)	0 (0.003)	0 (0.001)	0.073 (0.032)	0.003 (0.009)	0 (0.02)	0.283 (0.035)	0.16 (0.051)	0.215 (0.037)	0.09 (0.024)
GE0000391	O‘AHU	0.046 (0.024)	0.021 (0.018)	0.017 (0.006)	0.01 (0.013)	0 (0.002)	0.092 (0.026)	0.028 (0.019)	0.009 (0.032)	0 (0.001)	0 (0)	0.012 (0.016)	0.073 (0.029)	0 (0.003)	0 (0.009)	0 (0.003)	0 (0)	0.006 (0.018)	0 (0.003)	0.038 (0.028)	0.23 (0.03)	0 (0.028)	0.285 (0.039)	0.132 (0.023)
GE0000394	O‘AHU	0.02 (0.018)	0.053 (0.023)	0.019 (0.006)	0 (0.004)	0.023 (0.015)	0 (0.015)	0 (0.006)	0 (0)	0 (0.002)	0 (0.003)	0.069 (0.018)	0.017 (0.018)	0 (0)	0 (0.001)	0 (0.005)	0 (0.007)	0.027 (0.021)	0 (0)	0 (0.02)	0.308 (0.03)	0.055 (0.051)	0.259 (0.035)	0.149 (0.028)
GE0000398	KAUA‘I	0 (0.019)	0 (0.005)	0.023 (0.009)	0.053 (0.023)	0.028 (0.015)	0.064 (0.031)	0 (0.014)	0 (0)	0 (0)	0 (0)	0 (0.01)	0 (0.013)	0 (0.01)	0 (0)	0.02 (0.009)	0 (0.002)	0.056 (0.028)	0 (0.002)	0 (0.019)	0.282 (0.03)	0.043 (0.051)	0.307 (0.04)	0.124 (0.028)
GE0000399	KAUA‘I	0.013 (0.018)	0 (0.007)	0.025 (0.007)	0.069 (0.02)	0.019 (0.014)	0.029 (0.021)	0.017 (0.016)	0 (0.021)	0 (0)	0 (0.01)	0 (0.006)	0 (0.006)	0 (0.001)	0 (0.001)	0.003 (0.006)	0 (0.006)	0.047 (0.027)	0 (0.001)	0.006 (0.021)	0.325 (0.03)	0.021 (0.034)	0.318 (0.038)	0.107 (0.022)
GE0000400	KAUA‘I	0.004 (0.018)	0.014 (0.016)	0.021 (0.007)	0.059 (0.02)	0.046 (0.017)	0.012 (0.014)	0 (0.012)	0 (0.005)	0 (0)	0 (0)	0 (0.011)	0.028 (0.023)	0 (0.002)	0 (0)	0 (0.006)	0 (0.003)	0.043 (0.029)	0 (0.003)	0 (0)	0.264 (0.029)	0.088 (0.049)	0.338 (0.041)	0.082 (0.027)
GE0000401	KAUA‘I	0 (0.017)	0.019 (0.019)	0.027 (0.008)	0.028 (0.018)	0.008 (0.011)	0.02 (0.021)	0 (0.007)	0 (0.003)	0 (0.001)	0 (0.002)	0 (0.002)	0.02 (0.023)	0 (0.001)	0 (0)	0.02 (0.01)	0 (0.005)	0.064 (0.028)	0 (0)	0 (0)	0.246 (0.03)	0.123 (0.054)	0.297 (0.048)	0.129 (0.022)
GE0000406	O‘AHU	0.05 (0.023)	0.012 (0.015)	0.001 (0.005)	0.03 (0.018)	0 (0.009)	0.057 (0.028)	0 (0.006)	0 (0.006)	0 (0)	0 (0.006)	0.054 (0.017)	0 (0.004)	0 (0.008)	0 (0)	0 (0)	0 (0.002)	0.032 (0.026)	0 (0.008)	0.02 (0.021)	0.254 (0.028)	0.111 (0.047)	0.243 (0.037)	0.136 (0.022)
GE0000407	O‘AHU	0.035 (0.024)	0.054 (0.023)	0.004 (0.005)	0.046 (0.02)	0.01 (0.014)	0.049 (0.029)	0 (0.012)	0 (0.015)	0 (0)	0 (0.002)	0.034 (0.023)	0.015 (0.022)	0 (0.003)	0 (0.008)	0 (0.002)	0 (0.004)	0.018 (0.029)	0 (0.01)	0.046 (0.028)	0.267 (0.032)	0.079 (0.051)	0.223 (0.044)	0.12 (0.024)

GE0000408	OʻAHU	0.023 (0.024)	0.037 (0.02)	0.015 (0.006)	0.027 (0.014)	0.008 (0.011)	0.041 (0.032)	0 (0.004)	0 (0.02)	0 (0)	0 (0.001)	0.038 (0.021)	0.027 (0.021)	0 (0.014)	0 (0)	0 (0)	0 (0.002)	0.024 (0.025)	0 (0.007)	0 (0.008)	0.281 (0.033)	0.064 (0.044)	0.289 (0.041)	0.125 (0.025)
GE0000409	OʻAHU	0.042 (0.025)	0.001 (0.013)	0.013 (0.008)	0.048 (0.019)	0 (0.007)	0.008 (0.017)	0 (0.011)	0 (0.004)	0 (0)	0 (0.003)	0.01 (0.012)	0 (0.01)	0 (0)	0 (0.002)	0 (0.003)	0 (0.003)	0.009 (0.022)	0 (0.004)	0.075 (0.026)	0.25 (0.027)	0.138 (0.047)	0.268 (0.041)	0.138 (0.023)
GE0000410	OʻAHU	0 (0.017)	0.052 (0.017)	0.001 (0.004)	0.021 (0.015)	0.018 (0.015)	0.104 (0.034)	0 (0.005)	0 (0.016)	0 (0)	0 (0.005)	0.023 (0.02)	0.088 (0.03)	0 (0.006)	0 (0.002)	0 (0.004)	0 (0)	0.008 (0.022)	0 (0.001)	0 (0.016)	0.288 (0.03)	0.033 (0.035)	0.243 (0.038)	0.122 (0.025)
GE0002938	HAWAIʻI	0.002 (0.015)	0.029 (0.021)	0.016 (0.007)	0.01 (0.013)	0 (0.009)	0.013 (0.017)	0 (0.004)	0 (0.017)	0 (0.009)	0.012 (0.016)	0.006 (0.012)	0.007 (0.015)	0 (0.01)	0 (0.004)	0.012 (0.008)	0 (0.007)	0.044 (0.026)	0 (0.001)	0 (0.001)	0.222 (0.03)	0.153 (0.046)	0.345 (0.042)	0.13 (0.025)
GE0002939	HAWAIʻI	0 (0.007)	0 (0.01)	0.008 (0.006)	0.029 (0.017)	0.019 (0.016)	0 (0.002)	0.003 (0.015)	0 (0.005)	0 (0.009)	0 (0.005)	0.035 (0.016)	0.005 (0.018)	0.066 (0.032)	0 (0)	0.014 (0.008)	0 (0.004)	0.017 (0.021)	0 (0)	0 (0.011)	0.187 (0.026)	0.14 (0.043)	0.311 (0.035)	0.167 (0.022)
GE0002940	HAWAIʻI	0 (0.005)	0.016 (0.018)	0.022 (0.008)	0.032 (0.017)	0.012 (0.014)	0 (0.007)	0 (0.002)	0 (0.009)	0 (0.005)	0.042 (0.025)	0.022 (0.018)	0.001 (0.011)	0 (0.018)	0 (0.004)	0.005 (0.006)	0.011 (0.01)	0.004 (0.01)	0 (0.002)	0 (0.001)	0.213 (0.026)	0.142 (0.045)	0.346 (0.039)	0.131 (0.024)
GE0002941	HAWAIʻI	0 (0.003)	0.008 (0.016)	0.008 (0.007)	0.061 (0.019)	0.002 (0.01)	0.002 (0.013)	0 (0.014)	0 (0.019)	0 (0.012)	0.013 (0.018)	0.034 (0.02)	0 (0.005)	0 (0.008)	0 (0.002)	0 (0.005)	0 (0.006)	0.05 (0.027)	0 (0.005)	0 (0.006)	0.204 (0.029)	0.123 (0.051)	0.333 (0.043)	0.16 (0.025)
GE0002946	HAWAIʻI	0 (0.009)	0 (0.007)	0.019 (0.007)	0.011 (0.014)	0.007 (0.011)	0 (0.007)	0 (0.001)	0 (0.001)	0 (0.006)	0.044 (0.026)	0.046 (0.025)	0 (0.003)	0 (0.014)	0 (0.002)	0 (0.005)	0 (0.006)	0.09 (0.03)	0.016 (0.016)	0.001 (0.023)	0.167 (0.028)	0.151 (0.043)	0.289 (0.044)	0.157 (0.018)
GE0002947	HAWAIʻI	0 (0.013)	0.007 (0.019)	0.02 (0.008)	0.007 (0.012)	0.01 (0.013)	0 (0.007)	0 (0.001)	0 (0)	0 (0.009)	0.044 (0.025)	0 (0.014)	0 (0)	0 (0.003)	0 (0)	0 (0.005)	0 (0.006)	0.088 (0.031)	0.005 (0.011)	0.008 (0.025)	0.175 (0.028)	0.206 (0.049)	0.282 (0.049)	0.148 (0.022)
GE0002948	HAWAIʻI	0.017 (0.02)	0.037 (0.021)	0.02 (0.007)	0.005 (0.01)	0.012 (0.013)	0.03 (0.024)	0 (0.007)	0 (0.005)	0 (0.007)	0 (0.003)	0.008 (0.013)	0 (0.005)	0 (0.003)	0 (0.002)	0 (0.004)	0.002 (0.007)	0.087 (0.023)	0 (0.007)	0 (0.007)	0.192 (0.026)	0.121 (0.044)	0.307 (0.035)	0.163 (0.023)
GE0002950	HAWAIʻI	0.008 (0.017)	0 (0.001)	0.021 (0.007)	0.008 (0.015)	0.008 (0.013)	0.042 (0.025)	0 (0.007)	0 (0.009)	0 (0.005)	0 (0.002)	0.043 (0.019)	0 (0.009)	0 (0.016)	0 (0.004)	0.003 (0.005)	0.002 (0.007)	0.058 (0.027)	0 (0.007)	0 (0.012)	0.187 (0.023)	0.135 (0.046)	0.319 (0.039)	0.164 (0.023)
GE0003085	HAWAIʻI	0.004 (0.017)	0 (0.011)	0.013 (0.007)	0.013 (0.014)	0.026 (0.017)	0 (0.011)	0 (0.002)	0 (0.005)	0 (0.006)	0.012 (0.019)	0 (0.005)	0 (0.007)	0 (0.011)	0 (0.009)	0.004 (0.006)	0 (0.007)	0.045 (0.024)	0 (0.01)	0 (0.012)	0.247 (0.028)	0.144 (0.036)	0.355 (0.036)	0.137 (0.027)
GE0003086	HAWAIʻI	0 (0.006)	0.016 (0.017)	0.007 (0.006)	0.014 (0.016)	0.04 (0.017)	0 (0.003)	0 (0.002)	0 (0.004)	0 (0.006)	0 (0.012)	0.034 (0.018)	0 (0.013)	0 (0.003)	0 (0.003)	0 (0.001)	0 (0.004)	0.049 (0.024)	0 (0.002)	0 (0.007)	0.225 (0.028)	0.159 (0.044)	0.327 (0.038)	0.128 (0.027)
GE0003087	HAWAIʻI	0 (0.009)	0.023 (0.017)	0.011 (0.006)	0 (0.009)	0.023 (0.016)	0 (0.015)	0 (0.005)	0 (0)	0 (0.01)	0 (0.01)	0 (0.012)	0 (0)	0 (0.008)	0 (0.002)	0 (0.002)	0.002 (0.009)	0.053 (0.025)	0 (0.006)	0 (0.007)	0.22 (0.029)	0.162 (0.045)	0.333 (0.038)	0.173 (0.026)
GE0003088	HAWAIʻI	0 (0.018)	0.002 (0.013)	0.014 (0.007)	0.009 (0.013)	0.039 (0.017)	0 (0.012)	0 (0.01)	0 (0.011)	0 (0.008)	0.027 (0.022)	0.012 (0.017)	0 (0.004)	0 (0.016)	0 (0.006)	0 (0.003)	0.001 (0.008)	0.053 (0.026)	0 (0.01)	0.019 (0.024)	0.233 (0.03)	0.124 (0.044)	0.349 (0.035)	0.117 (0.025)
GE0003089	HAWAIʻI	0.019 (0.022)	0.032 (0.02)	0.022 (0.007)	0 (0.003)	0.003 (0.011)	0 (0.012)	0 (0)	0 (0)	0 (0.003)	0 (0.008)	0.03 (0.02)	0 (0.009)	0 (0.011)	0 (0)	0.017 (0.008)	0 (0.005)	0.089 (0.025)	0 (0.003)	0 (0.01)	0.175 (0.025)	0.148 (0.05)	0.278 (0.034)	0.186 (0.023)
GE0003090	HAWAIʻI	0 (0.016)	0.026 (0.018)	0.017 (0.007)	0.002 (0.009)	0 (0.011)	0.052 (0.032)	0.026 (0.02)	0 (0.006)	0 (0.002)	0.03 (0.027)	0 (0.004)	0 (0.01)	0.039 (0.026)	0 (0.01)	0.005 (0.006)	0.004 (0.007)	0 (0.012)	0 (0.001)	0.009 (0.02)	0.252 (0.029)	0.022 (0.039)	0.363 (0.033)	0.154 (0.024)
GE0003285	MAUI	0.025 (0.023)	0 (0.007)	0 (0.001)	0.065 (0.018)	0.003 (0.011)	0.024 (0.029)	0 (0.005)	0.025 (0.042)	0.038 (0.028)	0.003 (0.017)	0.027 (0.017)	0 (0.006)	0.053 (0.037)	0 (0)	0.004 (0.007)	0 (0.001)	0.049 (0.027)	0 (0.007)	0 (0.021)	0.12 (0.029)	0.272 (0.071)	0.216 (0.048)	0.076 (0.021)
GE0003296	MAUI	0.06 (0.026)	0 (0.011)	0.009 (0.006)	0.002 (0.01)	0.023 (0.017)	0.055 (0.027)	0 (0.001)	0 (0.003)	0.031 (0.023)	0 (0.003)	0.014 (0.016)	0.016 (0.02)	0 (0.016)	0 (0)	0.015 (0.008)	0 (0.001)	0 (0.024)	0 (0)	0.066 (0.027)	0.144 (0.028)	0.24 (0.051)	0.208 (0.044)	0.117 (0.026)
GE0003776	KAUAʻI	0 (0.003)	0 (0.01)	0.017 (0.008)	0.053 (0.019)	0.02 (0.014)	0.009 (0.015)	0 (0.008)	0 (0.004)	0 (0.002)	0 (0.004)	0.047 (0.021)	0.032 (0.023)	0 (0.005)	0 (0)	0.009 (0.008)	0 (0.002)	0.036 (0.026)	0 (0)	0 (0)	0.257 (0.032)	0.134 (0.041)	0.294 (0.038)	0.091 (0.024)

GE0003777	KAUA'I	0 (0.014)	0.005 (0.013)	0.015 (0.008)	0.057 (0.022)	0.03 (0.016)	0.029 (0.022)	0.011 (0.019)	0 (0.002)	0 (0.001)	0 (0.012)	0.031 (0.021)	0.007 (0.018)	0 (0)	0 (0)	0 (0.004)	0 (0.002)	0.056 (0.028)	0 (0.003)	0 (0.011)	0.267 (0.026)	0.075 (0.05)	0.328 (0.05)	0.09 (0.025)
GE0003778	KAUA'I	0 (0.012)	0.018 (0.014)	0.016 (0.008)	0.049 (0.021)	0 (0.009)	0.029 (0.023)	0 (0.008)	0 (0)	0 (0.004)	0 (0)	0.016 (0.016)	0.006 (0.019)	0 (0.009)	0 (0)	0.007 (0.007)	0.004 (0.009)	0.104 (0.028)	0 (0)	0 (0.001)	0.239 (0.027)	0 (0.031)	0.409 (0.037)	0.103 (0.026)
GE0003779	KAUA'I	0.032 (0.028)	0 (0.004)	0.011 (0.008)	0.04 (0.021)	0.01 (0.013)	0.083 (0.026)	0 (0.009)	0 (0)	0 (0)	0 (0)	0.047 (0.022)	0 (0.006)	0 (0.001)	0 (0)	0.005 (0.007)	0 (0.005)	0.085 (0.023)	0 (0.002)	0 (0.003)	0.239 (0.028)	0.083 (0.058)	0.266 (0.041)	0.1 (0.025)
GE0003781	KAUA'I	0.024 (0.026)	0 (0.006)	0.024 (0.008)	0.021 (0.015)	0.022 (0.015)	0.027 (0.022)	0.044 (0.022)	0 (0.001)	0 (0)	0 (0)	0.026 (0.019)	0.001 (0.016)	0 (0.003)	0 (0)	0.009 (0.009)	0 (0.003)	0.07 (0.03)	0 (0)	0 (0)	0.23 (0.034)	0.117 (0.053)	0.279 (0.041)	0.107 (0.022)
GE0003826	KAUA'I	0 (0.006)	0 (0.006)	0.024 (0.008)	0.042 (0.018)	0.033 (0.016)	0.07 (0.028)	0 (0.011)	0 (0.002)	0 (0)	0 (0)	0.011 (0.016)	0.005 (0.016)	0 (0.005)	0 (0.002)	0.018 (0.009)	0.002 (0.006)	0.051 (0.027)	0 (0)	0 (0.005)	0.214 (0.027)	0 (0.029)	0.392 (0.034)	0.139 (0.028)
GE0003827	KAUA'I	0 (0.009)	0 (0.006)	0.018 (0.008)	0.038 (0.018)	0.014 (0.011)	0.023 (0.019)	0 (0.011)	0 (0.005)	0 (0.008)	0 (0.001)	0 (0.006)	0 (0.005)	0 (0.004)	0 (0.004)	0.028 (0.009)	0 (0.001)	0.092 (0.024)	0 (0)	0 (0)	0.209 (0.025)	0.084 (0.04)	0.363 (0.037)	0.132 (0.025)
GE0003828	KAUA'I	0 (0.002)	0 (0.013)	0.018 (0.008)	0.012 (0.017)	0.021 (0.016)	0 (0.015)	0 (0.005)	0 (0.027)	0 (0.002)	0 (0.002)	0.031 (0.021)	0.035 (0.023)	0 (0.014)	0.018 (0.016)	0.018 (0.01)	0 (0)	0.061 (0.03)	0 (0.001)	0 (0)	0.186 (0.036)	0.072 (0.043)	0.399 (0.043)	0.128 (0.025)
GE0003960	MOLOKA'I	0 (0.002)	0.042 (0.023)	0.006 (0.006)	0.006 (0.014)	0.015 (0.013)	0.054 (0.031)	0.005 (0.018)	0.012 (0.031)	0 (0.01)	0 (0.003)	0 (0.003)	0.016 (0.022)	0.021 (0.027)	0 (0.006)	0.003 (0.006)	0 (0.001)	0.01 (0.022)	0 (0)	0.053 (0.032)	0.121 (0.026)	0.043 (0.051)	0.355 (0.045)	0.238 (0.026)
GE0003998	HAWAI'I	0.289 (0.032)	0.013 (0.019)	0 (0.002)	0.034 (0.018)	0.027 (0.015)	0.062 (0.032)	0 (0.009)	0 (0.013)	0 (0.002)	0.041 (0.034)	0 (0.007)	0 (0.005)	0 (0)	0 (0.003)	0 (0)	0 (0.002)	0 (0)	0 (0.001)	0.038 (0.024)	0.114 (0.028)	0.249 (0.05)	0.123 (0.043)	0.01 (0.017)
GE0003999	HAWAI'I	0.19 (0.026)	0.008 (0.016)	0.003 (0.005)	0.043 (0.018)	0.003 (0.009)	0.002 (0.016)	0 (0.009)	0 (0.002)	0 (0)	0 (0.013)	0.023 (0.017)	0.025 (0.023)	0 (0.006)	0 (0.002)	0 (0.003)	0 (0.002)	0 (0.002)	0 (0.001)	0.024 (0.02)	0.154 (0.026)	0.225 (0.045)	0.228 (0.036)	0.07 (0.025)
GE0004000	HAWAI'I	0.199 (0.023)	0.03 (0.019)	0 (0.002)	0.02 (0.014)	0 (0.003)	0.064 (0.026)	0 (0.011)	0 (0.01)	0 (0)	0 (0.011)	0 (0.004)	0 (0.014)	0 (0)	0 (0)	0 (0.004)	0.007 (0.008)	0 (0.013)	0 (0.008)	0.038 (0.024)	0.16 (0.024)	0.221 (0.049)	0.212 (0.035)	0.049 (0.023)
GE0004001	HAWAI'I	0.167 (0.029)	0.017 (0.016)	0 (0.004)	0.04 (0.018)	0.011 (0.012)	0 (0.003)	0 (0.002)	0 (0.014)	0 (0)	0 (0.014)	0.053 (0.02)	0.009 (0.018)	0 (0)	0 (0.006)	0 (0.004)	0 (0.006)	0 (0.008)	0 (0.002)	0.015 (0.017)	0.152 (0.027)	0.195 (0.044)	0.254 (0.033)	0.088 (0.025)
GE0004013	HAWAI'I	0.057 (0.03)	0.024 (0.02)	0.006 (0.006)	0.021 (0.021)	0 (0.005)	0.054 (0.032)	0 (0)	0 (0)	0 (0)	0 (0.009)	0 (0.002)	0 (0.007)	0 (0.001)	0 (0.002)	0.004 (0.007)	0.003 (0.008)	0.042 (0.025)	0 (0.008)	0 (0.001)	0.194 (0.031)	0.283 (0.058)	0.21 (0.044)	0.101 (0.027)
GE0004015	HAWAI'I	0.046 (0.029)	0.011 (0.017)	0 (0.004)	0.037 (0.022)	0.002 (0.011)	0.038 (0.027)	0 (0)	0 (0)	0 (0)	0 (0.015)	0 (0)	0 (0.003)	0 (0)	0 (0.004)	0.004 (0.007)	0.002 (0.008)	0.054 (0.023)	0 (0.005)	0 (0.006)	0.189 (0.032)	0.277 (0.056)	0.242 (0.041)	0.099 (0.025)
GE0004016	HAWAI'I	0.031 (0.026)	0.031 (0.02)	0.015 (0.008)	0.022 (0.02)	0 (0.002)	0.048 (0.032)	0 (0)	0 (0)	0 (0)	0 (0.007)	0 (0.001)	0.019 (0.021)	0 (0.002)	0 (0.009)	0.001 (0.006)	0.007 (0.009)	0.037 (0.024)	0 (0.003)	0 (0.012)	0.186 (0.029)	0.247 (0.05)	0.266 (0.038)	0.089 (0.023)
GE0004017	HAWAI'I	0.042 (0.031)	0.007 (0.016)	0.007 (0.007)	0.047 (0.023)	0 (0.009)	0.024 (0.025)	0 (0)	0 (0)	0 (0.002)	0 (0.011)	0 (0.003)	0 (0.011)	0 (0.001)	0 (0.005)	0 (0.004)	0.015 (0.012)	0.033 (0.023)	0 (0.006)	0 (0.004)	0.194 (0.036)	0.308 (0.055)	0.23 (0.045)	0.094 (0.029)
GE0004018	HAWAI'I	0.037 (0.031)	0.007 (0.016)	0.015 (0.008)	0.041 (0.023)	0 (0.002)	0.041 (0.03)	0 (0)	0 (0)	0 (0.012)	0 (0.007)	0 (0.003)	0.019 (0.023)	0 (0.004)	0 (0.006)	0 (0.005)	0.003 (0.007)	0.018 (0.02)	0 (0.005)	0 (0.017)	0.214 (0.034)	0.24 (0.063)	0.259 (0.044)	0.106 (0.025)
GE0004417	KAUA'I	0.003 (0.012)	0 (0.013)	0.019 (0.007)	0.014 (0.017)	0.019 (0.016)	0.02 (0.02)	0.016 (0.018)	0 (0.011)	0 (0)	0 (0.004)	0.027 (0.023)	0.018 (0.021)	0 (0.003)	0 (0)	0.017 (0.008)	0.005 (0.009)	0.076 (0.03)	0 (0.002)	0 (0.006)	0.217 (0.033)	0.125 (0.049)	0.358 (0.04)	0.066 (0.018)
GE0005125	MOLOKA'I	0 (0.008)	0.014 (0.018)	0 (0.005)	0.038 (0.02)	0.016 (0.013)	0.027 (0.026)	0.028 (0.028)	0 (0.035)	0 (0.003)	0 (0.012)	0 (0.004)	0 (0.015)	0.016 (0.023)	0.033 (0.025)	0 (0.002)	0 (0.001)	0.047 (0.031)	0 (0)	0.054 (0.034)	0.1 (0.027)	0.174 (0.067)	0.318 (0.048)	0.136 (0.026)
GE0005126	MOLOKA'I	0 (0.008)	0.014 (0.017)	0.011 (0.008)	0.048 (0.022)	0.057 (0.017)	0.025 (0.028)	0 (0.019)	0.057 (0.055)	0.01 (0.017)	0 (0)	0.013 (0.016)	0 (0.019)	0 (0.014)	0.046 (0.03)	0 (0.003)	0 (0.002)	0 (0.003)	0 (0.007)	0.092 (0.031)	0.146 (0.028)	0.119 (0.06)	0.245 (0.044)	0.116 (0.025)

GE0005127	MOLOKAʻI	0.012 (0.017)	0.013 (0.019)	0.009 (0.007)	0.031 (0.024)	0.011 (0.015)	0.006 (0.02)	0 (0.016)	0 (0.014)	0 (0.006)	0.014 (0.018)	0 (0.009)	0.06 (0.034)	0.005 (0.024)	0 (0.011)	0 (0.005)	0 (0.005)	0.061 (0.033)	0 (0)	0.016 (0.023)	0.117 (0.027)	0.249 (0.064)	0.282 (0.046)	0.113 (0.029)
HALE-4	MAUI	0.098 (0.023)	0.028 (0.019)	0.008 (0.006)	0.015 (0.015)	0 (0.009)	0.026 (0.022)	0 (0.001)	0 (0)	0.023 (0.022)	0 (0.006)	0.002 (0.014)	0 (0.01)	0 (0.002)	0 (0)	0.015 (0.007)	0.006 (0.007)	0.028 (0.022)	0 (0.006)	0 (0.006)	0.138 (0.028)	0.281 (0.052)	0.248 (0.037)	0.085 (0.02)
ID0014567	HAWAIʻI	0.065 (0.026)	0.032 (0.022)	0.003 (0.004)	0.013 (0.012)	0.038 (0.015)	0 (0.011)	0.041 (0.022)	0.07 (0.05)	0 (0)	0 (0.004)	0 (0.011)	0.035 (0.024)	0 (0.001)	0 (0.001)	0.013 (0.008)	0.005 (0.007)	0.02 (0.02)	0 (0.006)	0 (0.007)	0.227 (0.029)	0.054 (0.052)	0.323 (0.048)	0.062 (0.025)
ID0014568	HAWAIʻI	0.048 (0.027)	0.044 (0.02)	0 (0.003)	0.033 (0.016)	0.026 (0.017)	0.036 (0.025)	0.057 (0.024)	0.01 (0.033)	0 (0)	0 (0)	0.006 (0.015)	0.062 (0.029)	0 (0.003)	0 (0.007)	0.011 (0.007)	0 (0.002)	0 (0.019)	0 (0.004)	0.016 (0.017)	0.204 (0.032)	0 (0.033)	0.366 (0.039)	0.081 (0.023)
ID0014569	HAWAIʻI	0.081 (0.029)	0.02 (0.018)	0.005 (0.006)	0.032 (0.019)	0.033 (0.015)	0.045 (0.028)	0.006 (0.018)	0.051 (0.056)	0 (0)	0 (0.004)	0.026 (0.021)	0.067 (0.031)	0 (0.003)	0 (0.003)	0.006 (0.008)	0 (0.004)	0.032 (0.03)	0 (0.003)	0 (0.01)	0.157 (0.031)	0.055 (0.057)	0.323 (0.045)	0.061 (0.025)
ID0014571	OʻAHU	0 (0.014)	0.027 (0.017)	0.024 (0.006)	0 (0.009)	0 (0.006)	0.072 (0.032)	0.007 (0.015)	0 (0.023)	0 (0)	0.007 (0.019)	0 (0.011)	0.015 (0.023)	0 (0.018)	0 (0.005)	0.001 (0.005)	0 (0)	0 (0.016)	0 (0.005)	0.054 (0.027)	0.303 (0.024)	0.084 (0.051)	0.288 (0.04)	0.117 (0.021)
ID0014574	KAUAʻI	0 (0.013)	0.005 (0.012)	0.019 (0.008)	0.048 (0.018)	0.01 (0.013)	0.034 (0.024)	0 (0.004)	0 (0)	0 (0)	0 (0.008)	0.023 (0.019)	0.017 (0.021)	0 (0)	0 (0)	0 (0.005)	0 (0.006)	0.097 (0.028)	0 (0.001)	0 (0.015)	0.247 (0.028)	0.026 (0.038)	0.365 (0.045)	0.109 (0.024)
ID0014575	KAUAʻI	0 (0.007)	0 (0.002)	0.009 (0.008)	0.047 (0.02)	0.015 (0.013)	0.022 (0.02)	0 (0.002)	0 (0.015)	0 (0)	0 (0.002)	0.03 (0.021)	0.02 (0.022)	0 (0)	0 (0)	0 (0.003)	0 (0.005)	0.089 (0.032)	0 (0)	0 (0.009)	0.271 (0.033)	0.103 (0.049)	0.3 (0.044)	0.094 (0.023)
ID0014578	KAUAʻI	0 (0.014)	0 (0.001)	0.017 (0.007)	0.032 (0.019)	0.019 (0.014)	0.067 (0.026)	0 (0.002)	0 (0)	0 (0)	0 (0)	0.013 (0.017)	0 (0.004)	0 (0.004)	0 (0)	0.01 (0.007)	0 (0.003)	0.068 (0.026)	0 (0)	0 (0.013)	0.286 (0.029)	0.125 (0.047)	0.258 (0.04)	0.106 (0.025)
ID0014579	KAUAʻI	0 (0.008)	0 (0.003)	0.01 (0.007)	0.063 (0.019)	0.017 (0.013)	0.047 (0.028)	0 (0.015)	0 (0.01)	0 (0)	0.001 (0.02)	0.001 (0.015)	0 (0.005)	0 (0)	0 (0)	0 (0.006)	0.002 (0.009)	0.058 (0.025)	0 (0.002)	0 (0.009)	0.285 (0.029)	0.162 (0.049)	0.266 (0.048)	0.088 (0.026)
ID0014580	KAUAʻI	0.01 (0.019)	0.025 (0.018)	0.003 (0.005)	0.067 (0.019)	0.046 (0.016)	0.044 (0.024)	0.021 (0.024)	0 (0)	0 (0.002)	0 (0.006)	0 (0.007)	0.023 (0.023)	0 (0.001)	0 (0)	0.005 (0.007)	0 (0.003)	0.077 (0.033)	0 (0.001)	0 (0.01)	0.271 (0.032)	0.036 (0.048)	0.312 (0.044)	0.06 (0.025)
ID0014581	KAUAʻI	0 (0.012)	0 (0.01)	0.035 (0.008)	0.02 (0.017)	0.041 (0.015)	0.05 (0.026)	0 (0.012)	0 (0)	0 (0)	0 (0.001)	0.032 (0.022)	0.005 (0.02)	0 (0)	0 (0.001)	0 (0.005)	0 (0.005)	0.101 (0.032)	0 (0)	0 (0.019)	0.255 (0.033)	0.106 (0.047)	0.263 (0.046)	0.092 (0.021)
ID0015006	KAUAʻI	0 (0.007)	0 (0.006)	0.014 (0.007)	0.058 (0.02)	0.02 (0.014)	0.047 (0.024)	0 (0.002)	0 (0.022)	0 (0)	0 (0.005)	0.031 (0.022)	0.013 (0.022)	0 (0.01)	0 (0.01)	0.01 (0.008)	0 (0.003)	0.071 (0.025)	0 (0)	0 (0.002)	0.222 (0.029)	0.065 (0.046)	0.343 (0.041)	0.106 (0.021)
ID0015007	OʻAHU	0.004 (0.016)	0.005 (0.015)	0.001 (0.005)	0.039 (0.018)	0 (0.008)	0.018 (0.021)	0 (0)	0 (0)	0 (0.004)	0 (0.014)	0.009 (0.016)	0 (0.002)	0.032 (0.026)	0 (0)	0.012 (0.008)	0.008 (0.009)	0.017 (0.017)	0 (0.007)	0 (0.012)	0.296 (0.024)	0.207 (0.047)	0.203 (0.037)	0.148 (0.025)
ID0015009	OʻAHU	0.037 (0.028)	0 (0.012)	0.001 (0.006)	0.053 (0.022)	0.027 (0.015)	0.079 (0.029)	0.032 (0.028)	0 (0.019)	0 (0)	0 (0.004)	0 (0.011)	0 (0.011)	0.022 (0.024)	0 (0.003)	0 (0.002)	0 (0.002)	0 (0.012)	0 (0.004)	0.003 (0.016)	0.31 (0.029)	0.125 (0.057)	0.189 (0.045)	0.121 (0.024)
ID0015010	KAUAʻI	0.004 (0.015)	0.01 (0.015)	0.014 (0.008)	0.053 (0.02)	0.029 (0.016)	0.016 (0.02)	0 (0.001)	0 (0.003)	0 (0)	0 (0.006)	0.068 (0.023)	0.004 (0.017)	0 (0)	0 (0)	0 (0.004)	0 (0.002)	0.063 (0.031)	0 (0)	0 (0.021)	0.284 (0.034)	0.083 (0.042)	0.274 (0.036)	0.1 (0.022)
ID0015011	KAUAʻI	0 (0.005)	0 (0.01)	0.019 (0.008)	0.029 (0.018)	0.035 (0.017)	0.056 (0.024)	0 (0.004)	0 (0.018)	0 (0.002)	0 (0)	0.027 (0.02)	0.03 (0.025)	0 (0.014)	0 (0.003)	0.009 (0.009)	0 (0)	0.062 (0.026)	0 (0)	0 (0.013)	0.242 (0.026)	0.102 (0.05)	0.306 (0.047)	0.082 (0.022)
ID0015012	KAUAʻI	0.008 (0.016)	0 (0.008)	0.03 (0.008)	0.037 (0.019)	0.027 (0.014)	0.042 (0.025)	0 (0.009)	0 (0)	0 (0)	0 (0.003)	0.024 (0.021)	0.025 (0.024)	0 (0)	0 (0)	0 (0.001)	0 (0.002)	0.062 (0.027)	0 (0.005)	0 (0.008)	0.242 (0.024)	0.093 (0.049)	0.284 (0.041)	0.125 (0.022)
ID0016856	KAUAʻI	0 (0.009)	0 (0.001)	0 (0.005)	0.082 (0.018)	0.014 (0.015)	0 (0.001)	0.01 (0.014)	0 (0.018)	0 (0.002)	0 (0.004)	0.025 (0.021)	0 (0.002)	0 (0.008)	0 (0)	0.007 (0.008)	0 (0.001)	0.077 (0.027)	0 (0.001)	0 (0.005)	0.227 (0.031)	0.093 (0.041)	0.35 (0.037)	0.113 (0.026)
ID0016865	OʻAHU	0.013 (0.021)	0.041 (0.019)	0 (0.004)	0.051 (0.017)	0.028 (0.016)	0.016 (0.018)	0 (0.009)	0 (0.008)	0 (0.006)	0.012 (0.022)	0.005 (0.013)	0.037 (0.026)	0 (0.013)	0 (0.006)	0 (0.004)	0 (0)	0.086 (0.032)	0 (0.005)	0 (0.009)	0.224 (0.028)	0.09 (0.045)	0.289 (0.038)	0.106 (0.027)

ID0016866	O'AHU	0.035 (0.024)	0.023 (0.019)	0.013 (0.007)	0.029 (0.017)	0.024 (0.016)	0.027 (0.02)	0 (0.003)	0 (0.018)	0 (0)	0 (0.009)	0.008 (0.01)	0.015 (0.019)	0 (0.001)	0 (0.002)	0.001 (0.006)	0 (0)	0.046 (0.027)	0 (0)	0 (0.002)	0.336 (0.031)	0.045 (0.045)	0.3 (0.034)	0.099 (0.027)
ID0016876	O'AHU	0.006 (0.017)	0.003 (0.013)	0.022 (0.006)	0 (0.005)	0.033 (0.016)	0.033 (0.025)	0 (0)	0.057 (0.044)	0 (0)	0 (0.015)	0.009 (0.015)	0 (0.011)	0.05 (0.035)	0 (0.014)	0 (0.006)	0 (0.002)	0.017 (0.021)	0 (0.002)	0 (0.011)	0.335 (0.034)	0.157 (0.06)	0.216 (0.049)	0.061 (0.023)
ID0016906	O'AHU	0.01 (0.022)	0.052 (0.021)	0.022 (0.008)	0 (0.008)	0 (0.008)	0.035 (0.024)	0 (0)	0 (0)	0 (0.007)	0 (0)	0.02 (0.018)	0.043 (0.028)	0 (0.006)	0 (0)	0 (0.005)	0 (0.005)	0.011 (0.019)	0 (0.006)	0 (0.007)	0.294 (0.029)	0.119 (0.044)	0.268 (0.038)	0.127 (0.025)
ID0016907	O'AHU	0.028 (0.026)	0.025 (0.019)	0.027 (0.007)	0 (0.005)	0.004 (0.013)	0.056 (0.029)	0 (0.008)	0 (0.027)	0 (0.001)	0 (0.004)	0 (0.01)	0.041 (0.028)	0 (0)	0 (0.003)	0 (0.005)	0 (0)	0.031 (0.026)	0 (0)	0 (0.007)	0.281 (0.035)	0.057 (0.048)	0.302 (0.041)	0.147 (0.025)
ID0016908	O'AHU	0 (0.009)	0.04 (0.018)	0.016 (0.007)	0 (0.005)	0.023 (0.015)	0.073 (0.03)	0 (0.004)	0 (0.006)	0 (0.006)	0 (0)	0.01 (0.015)	0.08 (0.033)	0 (0.005)	0 (0.011)	0.01 (0.008)	0.009 (0.01)	0 (0.013)	0.005 (0.013)	0 (0.007)	0.283 (0.025)	0.078 (0.05)	0.278 (0.041)	0.093 (0.025)
ID0016911	O'AHU	0 (0.016)	0.048 (0.022)	0.006 (0.005)	0 (0.006)	0 (0.009)	0.068 (0.029)	0 (0.011)	0 (0.004)	0 (0)	0 (0.003)	0 (0.009)	0.019 (0.022)	0 (0.001)	0 (0)	0.003 (0.006)	0 (0.002)	0.06 (0.029)	0 (0.008)	0.006 (0.019)	0.267 (0.03)	0.169 (0.049)	0.219 (0.041)	0.135 (0.024)
ID0016912	O'AHU	0.015 (0.023)	0.043 (0.021)	0.017 (0.008)	0.006 (0.013)	0.008 (0.014)	0.075 (0.031)	0.018 (0.022)	0 (0.002)	0 (0)	0 (0.001)	0.001 (0.014)	0.025 (0.026)	0 (0.003)	0 (0.004)	0 (0.004)	0 (0.002)	0.043 (0.029)	0 (0)	0 (0.003)	0.292 (0.033)	0.023 (0.039)	0.29 (0.041)	0.144 (0.025)
ID0016915	O'AHU	0 (0)	0.026 (0.018)	0.015 (0.006)	0 (0.008)	0.025 (0.016)	0 (0.01)	0 (0.001)	0 (0.006)	0 (0.001)	0 (0.005)	0.029 (0.02)	0 (0.004)	0.01 (0.021)	0 (0.008)	0 (0.003)	0 (0.002)	0.067 (0.028)	0 (0)	0.002 (0.019)	0.271 (0.03)	0.232 (0.049)	0.205 (0.043)	0.116 (0.023)
ID0016922	O'AHU	0 (0.016)	0.011 (0.015)	0.007 (0.005)	0 (0.007)	0.014 (0.013)	0.041 (0.029)	0 (0.012)	0 (0.004)	0 (0.001)	0 (0.005)	0.007 (0.016)	0.069 (0.033)	0 (0)	0 (0.001)	0.002 (0.006)	0 (0.004)	0.049 (0.033)	0 (0.001)	0.001 (0.02)	0.266 (0.03)	0.097 (0.053)	0.274 (0.037)	0.163 (0.023)
ID0016925	O'AHU	0.009 (0.02)	0.021 (0.02)	0.03 (0.007)	0 (0.008)	0.019 (0.017)	0.004 (0.016)	0 (0.006)	0.016 (0.036)	0 (0.001)	0 (0.006)	0.028 (0.017)	0 (0.017)	0 (0.005)	0 (0.006)	0.011 (0.008)	0 (0)	0.044 (0.032)	0 (0.004)	0.023 (0.026)	0.258 (0.036)	0.064 (0.046)	0.338 (0.044)	0.136 (0.026)
ID0016929	O'AHU	0.018 (0.023)	0.023 (0.018)	0.01 (0.006)	0 (0.005)	0 (0.008)	0.078 (0.026)	0 (0)	0 (0.001)	0 (0)	0 (0)	0 (0.005)	0.095 (0.032)	0 (0.005)	0 (0)	0.009 (0.007)	0 (0)	0 (0.015)	0 (0.008)	0 (0.017)	0.277 (0.028)	0.071 (0.047)	0.265 (0.042)	0.154 (0.023)
ID0016930	O'AHU	0.002 (0.012)	0.025 (0.016)	0.024 (0.006)	0.007 (0.013)	0 (0.006)	0.028 (0.023)	0 (0.006)	0 (0.012)	0 (0.007)	0 (0)	0.016 (0.016)	0.071 (0.027)	0.04 (0.03)	0 (0.007)	0.005 (0.008)	0 (0.002)	0 (0.005)	0 (0.005)	0.006 (0.016)	0.312 (0.027)	0.07 (0.051)	0.268 (0.037)	0.126 (0.024)
ID0016931	O'AHU	0.032 (0.022)	0.06 (0.02)	0.006 (0.006)	0.025 (0.015)	0.008 (0.013)	0.089 (0.027)	0 (0.012)	0 (0.004)	0 (0)	0 (0.006)	0 (0.007)	0 (0.017)	0 (0)	0 (0.003)	0.002 (0.007)	0.002 (0.006)	0.051 (0.023)	0 (0.004)	0 (0.006)	0.273 (0.029)	0.136 (0.045)	0.202 (0.038)	0.113 (0.025)
ID0016934	O'AHU	0.053 (0.027)	0.055 (0.019)	0.005 (0.006)	0.011 (0.013)	0 (0.009)	0.085 (0.032)	0 (0.009)	0 (0.024)	0 (0)	0 (0.006)	0 (0.016)	0 (0.006)	0 (0)	0 (0)	0.006 (0.008)	0 (0.003)	0.069 (0.028)	0 (0.006)	0 (0.003)	0.231 (0.03)	0.153 (0.053)	0.199 (0.048)	0.134 (0.025)
ID0016936	O'AHU	0.021 (0.021)	0.011 (0.017)	0.011 (0.007)	0.019 (0.014)	0 (0.008)	0.047 (0.028)	0 (0.004)	0 (0.01)	0 (0)	0.011 (0.02)	0 (0.002)	0 (0.01)	0 (0.005)	0 (0.008)	0 (0.005)	0.003 (0.006)	0.015 (0.016)	0 (0.006)	0 (0.004)	0.394 (0.027)	0.118 (0.049)	0.222 (0.041)	0.128 (0.022)
ID0016937	O'AHU	0.068 (0.023)	0.034 (0.017)	0.02 (0.006)	0 (0.007)	0.018 (0.014)	0.028 (0.021)	0 (0.001)	0 (0.02)	0 (0.005)	0 (0.01)	0 (0.007)	0.014 (0.019)	0 (0.015)	0 (0)	0.004 (0.006)	0 (0)	0.018 (0.017)	0 (0.005)	0 (0.008)	0.338 (0.024)	0.107 (0.048)	0.252 (0.038)	0.098 (0.024)
ID0016939	O'AHU	0.032 (0.021)	0.006 (0.015)	0.015 (0.006)	0.006 (0.015)	0.007 (0.013)	0.003 (0.018)	0 (0.015)	0.068 (0.037)	0 (0)	0 (0.009)	0.015 (0.017)	0 (0.008)	0.013 (0.027)	0.001 (0.018)	0.003 (0.007)	0 (0.004)	0.062 (0.024)	0 (0.004)	0 (0.013)	0.332 (0.027)	0.037 (0.039)	0.276 (0.042)	0.123 (0.027)
ID0019825	O'AHU	0.033 (0.023)	0.008 (0.015)	0.014 (0.006)	0.02 (0.014)	0 (0.006)	0.076 (0.031)	0 (0.011)	0.013 (0.03)	0 (0.001)	0 (0.004)	0 (0.003)	0.019 (0.025)	0 (0.009)	0.009 (0.015)	0.002 (0.005)	0 (0.004)	0.006 (0.016)	0 (0.006)	0.013 (0.017)	0.357 (0.031)	0 (0.036)	0.251 (0.042)	0.179 (0.023)
ID0019826	O'AHU	0.061 (0.024)	0 (0.013)	0.014 (0.007)	0.004 (0.013)	0.011 (0.013)	0.003 (0.017)	0 (0.005)	0 (0.003)	0 (0)	0 (0.007)	0 (0.01)	0 (0.007)	0 (0.003)	0 (0.002)	0 (0.005)	0 (0.004)	0.058 (0.025)	0 (0.008)	0 (0.008)	0.351 (0.03)	0.084 (0.044)	0.272 (0.042)	0.142 (0.023)
ID0019884	O'AHU	0 (0.004)	0.012 (0.013)	0 (0.001)	0 (0.002)	0 (0.004)	0 (0.013)	0.933 (0.04)	0 (0)	0 (0)	0 (0.011)	0.003 (0.008)	0 (0.005)	0 (0)	0 (0.003)	0 (0.002)	0 (0.001)	0 (0)	0 (0.001)	0 (0.003)	0.005 (0.004)	0 (0)	0.046 (0.033)	0 (0.007)

ID0019885	O‘AHU	0.015 (0.02)	0.008 (0.018)	0.001 (0.005)	0.065 (0.017)	0 (0.003)	0.021 (0.023)	0 (0.011)	0.039 (0.033)	0 (0)	0 (0.01)	0.014 (0.015)	0.011 (0.021)	0 (0.01)	0 (0.012)	0.004 (0.007)	0 (0)	0.06 (0.028)	0 (0.009)	0 (0.012)	0.256 (0.026)	0.1 (0.046)	0.265 (0.039)	0.14 (0.023)
ID0019886	O‘AHU	0 (0.013)	0.02 (0.016)	0.014 (0.007)	0.023 (0.016)	0 (0.007)	0.095 (0.024)	0 (0.003)	0 (0.001)	0 (0)	0 (0)	0 (0.011)	0.024 (0.028)	0 (0.004)	0 (0.009)	0.019 (0.008)	0 (0.005)	0.068 (0.028)	0 (0.001)	0 (0.008)	0.275 (0.03)	0.019 (0.036)	0.287 (0.032)	0.155 (0.021)
ID0019891	O‘AHU	0.037 (0.024)	0.032 (0.021)	0 (0.004)	0.052 (0.019)	0 (0.009)	0.011 (0.02)	0 (0.01)	0 (0.002)	0 (0.001)	0 (0.005)	0.036 (0.021)	0.006 (0.021)	0 (0.001)	0 (0.001)	0.002 (0.006)	0 (0.004)	0.072 (0.027)	0 (0.003)	0 (0.013)	0.263 (0.029)	0.099 (0.049)	0.256 (0.032)	0.132 (0.028)
ID0019896	O‘AHU	0.034 (0.023)	0 (0.009)	0.007 (0.005)	0.041 (0.019)	0.01 (0.011)	0.037 (0.026)	0 (0.002)	0 (0.013)	0 (0)	0 (0)	0 (0.009)	0 (0.003)	0 (0.009)	0 (0.003)	0.002 (0.006)	0 (0.001)	0.043 (0.022)	0 (0.003)	0 (0.006)	0.3 (0.027)	0.038 (0.036)	0.353 (0.039)	0.136 (0.026)
ID0019899	KAUA‘I	0 (0.011)	0 (0.01)	0.022 (0.008)	0.032 (0.019)	0.018 (0.015)	0.033 (0.024)	0 (0)	0 (0)	0 (0)	0 (0)	0.037 (0.023)	0.008 (0.021)	0 (0)	0 (0)	0 (0.005)	0 (0.002)	0.059 (0.028)	0 (0.002)	0 (0.003)	0.296 (0.033)	0.121 (0.044)	0.264 (0.043)	0.111 (0.024)
ID0019900	KAUA‘I	0 (0.005)	0 (0.008)	0.013 (0.008)	0.066 (0.02)	0.038 (0.016)	0.007 (0.016)	0.001 (0.015)	0 (0.003)	0 (0)	0 (0.001)	0.04 (0.022)	0 (0.006)	0 (0)	0 (0)	0.003 (0.007)	0 (0.004)	0.046 (0.029)	0 (0.002)	0 (0.003)	0.267 (0.031)	0.162 (0.051)	0.28 (0.042)	0.078 (0.026)
ID0019901	KAUA‘I	0 (0.004)	0.015 (0.013)	0 (0)	0 (0.001)	0 (0.004)	0 (0.011)	0.945 (0.039)	0 (0.002)	0 (0.001)	0 (0.013)	0 (0.005)	0 (0.004)	0 (0)	0 (0.003)	0 (0.001)	0 (0.001)	0 (0)	0 (0)	0 (0.002)	0 (0.002)	0 (0)	0.039 (0.032)	0 (0.006)
ID0019910	O‘AHU	0 (0.011)	0.036 (0.019)	0.008 (0.006)	0.005 (0.01)	0 (0.008)	0.053 (0.027)	0 (0.005)	0 (0)	0 (0.002)	0 (0)	0.025 (0.02)	0.019 (0.022)	0 (0.012)	0 (0.003)	0.013 (0.007)	0 (0.004)	0.035 (0.022)	0 (0.002)	0 (0.005)	0.298 (0.027)	0.099 (0.052)	0.273 (0.042)	0.135 (0.024)
ID0019912	O‘AHU	0.043 (0.025)	0.016 (0.016)	0.003 (0.005)	0.051 (0.019)	0.018 (0.013)	0.059 (0.029)	0 (0.009)	0 (0.025)	0 (0)	0.025 (0.028)	0.04 (0.02)	0 (0.016)	0.005 (0.021)	0 (0.003)	0.004 (0.007)	0.008 (0.009)	0.049 (0.028)	0 (0.002)	0 (0.007)	0.304 (0.032)	0.081 (0.051)	0.189 (0.041)	0.107 (0.025)
ID0019913	O‘AHU	0.071 (0.021)	0.026 (0.018)	0.021 (0.006)	0.028 (0.017)	0.038 (0.017)	0.03 (0.021)	0 (0.005)	0 (0.014)	0 (0)	0 (0.003)	0.035 (0.021)	0 (0.009)	0 (0.008)	0 (0.005)	0 (0.004)	0.007 (0.009)	0.018 (0.018)	0 (0.002)	0 (0.016)	0.31 (0.028)	0.06 (0.043)	0.247 (0.04)	0.109 (0.028)
ID0019914	O‘AHU	0.045 (0.024)	0.048 (0.021)	0.005 (0.006)	0.05 (0.019)	0.012 (0.014)	0.054 (0.029)	0.007 (0.019)	0 (0.006)	0 (0)	0 (0)	0.007 (0.014)	0.046 (0.024)	0 (0.002)	0 (0.004)	0.007 (0.007)	0 (0.001)	0.005 (0.019)	0 (0.002)	0 (0.007)	0.322 (0.027)	0.006 (0.036)	0.262 (0.037)	0.125 (0.029)
ID0021903	KAUA‘I	0 (0.007)	0.005 (0.015)	0.02 (0.009)	0.042 (0.019)	0.011 (0.014)	0.019 (0.021)	0 (0.001)	0 (0.002)	0 (0)	0 (0)	0.002 (0.012)	0.01 (0.019)	0 (0.001)	0 (0.001)	0.013 (0.009)	0 (0.002)	0.077 (0.024)	0 (0.003)	0 (0)	0.237 (0.028)	0.045 (0.041)	0.39 (0.04)	0.127 (0.026)
ID0021904	KAUA‘I	0 (0.004)	0.011 (0.013)	0.015 (0.008)	0.063 (0.018)	0.038 (0.017)	0.061 (0.027)	0 (0.004)	0 (0.021)	0 (0)	0 (0)	0.043 (0.022)	0.045 (0.027)	0 (0.001)	0 (0.003)	0 (0.006)	0 (0)	0.069 (0.03)	0 (0.001)	0 (0.021)	0.254 (0.028)	0.048 (0.044)	0.252 (0.036)	0.102 (0.025)
ID0021906	KAUA‘I	0.002 (0.013)	0 (0.012)	0.014 (0.008)	0.061 (0.022)	0.039 (0.014)	0.023 (0.02)	0.03 (0.023)	0 (0.01)	0 (0)	0 (0.006)	0.018 (0.02)	0.011 (0.02)	0 (0.002)	0 (0)	0.005 (0.007)	0 (0.003)	0.08 (0.03)	0 (0)	0 (0.007)	0.265 (0.037)	0.073 (0.057)	0.309 (0.045)	0.07 (0.027)
ID0021907	KAUA‘I	0 (0.005)	0 (0.005)	0.018 (0.008)	0.058 (0.02)	0.037 (0.016)	0.018 (0.02)	0.012 (0.02)	0 (0.002)	0 (0)	0 (0.006)	0.008 (0.015)	0.036 (0.026)	0 (0)	0 (0)	0.004 (0.007)	0 (0.001)	0.052 (0.03)	0 (0)	0 (0.017)	0.262 (0.028)	0.075 (0.042)	0.302 (0.041)	0.118 (0.023)
ID0021911	KAUA‘I	0.017 (0.02)	0.015 (0.015)	0.006 (0.007)	0.04 (0.02)	0.017 (0.015)	0.019 (0.02)	0 (0.006)	0 (0)	0 (0)	0 (0)	0.03 (0.023)	0 (0.02)	0 (0)	0 (0)	0.002 (0.006)	0 (0.004)	0.09 (0.026)	0 (0.001)	0 (0)	0.252 (0.034)	0.112 (0.058)	0.298 (0.048)	0.103 (0.024)
ID0021913	KAUA‘I	0 (0.01)	0.004 (0.012)	0.018 (0.008)	0.035 (0.018)	0.004 (0.01)	0.039 (0.022)	0 (0.001)	0 (0)	0 (0)	0 (0.003)	0.04 (0.022)	0.014 (0.022)	0 (0.004)	0 (0)	0 (0.004)	0 (0.002)	0.097 (0.027)	0 (0.002)	0 (0.001)	0.213 (0.033)	0.129 (0.042)	0.282 (0.04)	0.124 (0.021)
ID0021942	O‘AHU	0 (0.009)	0 (0.01)	0.013 (0.007)	0.016 (0.015)	0.005 (0.01)	0.003 (0.015)	0 (0.011)	0.129 (0.049)	0 (0.001)	0 (0.009)	0.026 (0.02)	0.01 (0.021)	0.003 (0.017)	0 (0.015)	0 (0.003)	0 (0.003)	0.051 (0.029)	0 (0.007)	0.004 (0.016)	0.324 (0.033)	0.073 (0.039)	0.219 (0.035)	0.124 (0.024)
ID0021945	O‘AHU	0.023 (0.023)	0.046 (0.021)	0.004 (0.006)	0.023 (0.018)	0.002 (0.01)	0.067 (0.03)	0 (0.003)	0 (0)	0 (0.008)	0 (0)	0.021 (0.02)	0.019 (0.024)	0 (0.017)	0 (0.003)	0.01 (0.008)	0 (0.005)	0.047 (0.027)	0 (0.007)	0 (0.021)	0.272 (0.029)	0.069 (0.047)	0.292 (0.048)	0.106 (0.027)
ID0021949	O‘AHU	0.015 (0.017)	0.029 (0.017)	0.014 (0.007)	0.018 (0.015)	0.016 (0.015)	0.011 (0.013)	0 (0.004)	0 (0.004)	0 (0)	0 (0.002)	0.024 (0.018)	0 (0.01)	0 (0.015)	0 (0.001)	0 (0.003)	0.002 (0.007)	0.021 (0.017)	0 (0.004)	0.01 (0.017)	0.382 (0.027)	0.134 (0.043)	0.189 (0.035)	0.137 (0.025)

ID0021954	O‘AHU	0.001 (0.015)	0.021 (0.019)	0.003 (0.006)	0.065 (0.021)	0.004 (0.01)	0 (0.006)	0.001 (0.015)	0 (0)	0 (0)	0.011 (0.017)	0.045 (0.023)	0 (0.014)	0 (0.008)	0 (0)	0 (0.004)	0 (0.002)	0.059 (0.026)	0 (0.002)	0 (0.006)	0.3 (0.029)	0.094 (0.045)	0.276 (0.042)	0.118 (0.022)
ID0021956	O‘AHU	0.043 (0.021)	0 (0.007)	0.009 (0.006)	0.026 (0.014)	0.009 (0.012)	0.045 (0.024)	0 (0)	0 (0.029)	0 (0.003)	0 (0.009)	0.013 (0.016)	0.012 (0.016)	0 (0.006)	0 (0.008)	0.015 (0.007)	0 (0.005)	0.018 (0.02)	0 (0.007)	0 (0.005)	0.32 (0.028)	0.145 (0.051)	0.246 (0.039)	0.099 (0.025)
ID0021957	O‘AHU	0.026 (0.018)	0 (0.013)	0.017 (0.006)	0.012 (0.014)	0.037 (0.017)	0.059 (0.026)	0 (0.015)	0.072 (0.044)	0 (0.001)	0 (0.006)	0 (0.009)	0 (0.011)	0 (0.01)	0 (0.007)	0 (0.003)	0 (0.001)	0.029 (0.023)	0 (0)	0 (0)	0.359 (0.027)	0.112 (0.045)	0.178 (0.042)	0.099 (0.027)
ID0023113	O‘AHU	0 (0.013)	0.016 (0.02)	0.006 (0.006)	0 (0.004)	0.032 (0.015)	0.077 (0.038)	0 (0.003)	0 (0.009)	0 (0.001)	0 (0.002)	0.016 (0.019)	0 (0.014)	0 (0.003)	0 (0.005)	0 (0.007)	0 (0.002)	0.063 (0.025)	0 (0)	0 (0.008)	0.312 (0.032)	0.092 (0.062)	0.239 (0.049)	0.145 (0.027)
ID0023114	O‘AHU	0.016 (0.022)	0.008 (0.019)	0.01 (0.006)	0 (0.007)	0.023 (0.016)	0.071 (0.033)	0 (0.01)	0 (0.018)	0 (0)	0 (0.007)	0.023 (0.019)	0 (0.012)	0 (0)	0 (0)	0 (0.004)	0 (0)	0.034 (0.022)	0 (0.004)	0 (0.017)	0.286 (0.03)	0.142 (0.052)	0.257 (0.041)	0.131 (0.031)
ID0023115	O‘AHU	0.005 (0.016)	0.014 (0.016)	0.009 (0.005)	0.01 (0.011)	0.022 (0.013)	0.018 (0.017)	0 (0.008)	0.027 (0.034)	0 (0.002)	0 (0.007)	0.067 (0.019)	0 (0.005)	0.015 (0.021)	0 (0.007)	0 (0)	0 (0.005)	0.003 (0.023)	0 (0.001)	0.051 (0.029)	0.323 (0.027)	0.112 (0.044)	0.195 (0.041)	0.129 (0.024)
ID0023116	O‘AHU	0.047 (0.023)	0.003 (0.012)	0.02 (0.007)	0.015 (0.015)	0.013 (0.014)	0 (0.006)	0 (0.005)	0 (0.005)	0 (0)	0 (0)	0.022 (0.018)	0.003 (0.013)	0 (0.007)	0 (0)	0.014 (0.009)	0 (0.002)	0.012 (0.016)	0 (0.005)	0 (0.01)	0.363 (0.027)	0.104 (0.045)	0.275 (0.041)	0.111 (0.027)
ID0023555	HAWAI‘I	0.1 (0.029)	0.009 (0.013)	0 (0.004)	0.038 (0.017)	0 (0.006)	0.076 (0.036)	0 (0.007)	0 (0)	0 (0.001)	0 (0)	0.045 (0.022)	0.041 (0.023)	0 (0.011)	0.015 (0.02)	0.02 (0.007)	0.002 (0.007)	0.001 (0.017)	0 (0.002)	0.013 (0.021)	0.139 (0.027)	0.167 (0.058)	0.244 (0.04)	0.091 (0.022)
ID0023556	O‘AHU	0 (0.017)	0.015 (0.02)	0.006 (0.005)	0 (0.01)	0.023 (0.015)	0.064 (0.032)	0.014 (0.017)	0 (0.024)	0 (0.005)	0 (0.004)	0.007 (0.015)	0.04 (0.029)	0 (0.01)	0.011 (0.017)	0 (0.005)	0 (0.002)	0.022 (0.024)	0 (0.001)	0 (0.017)	0.318 (0.035)	0.076 (0.052)	0.256 (0.044)	0.148 (0.029)
ID0023557	O‘AHU	0.038 (0.028)	0 (0.007)	0.007 (0.006)	0.012 (0.015)	0.034 (0.015)	0.04 (0.033)	0 (0.003)	0 (0.015)	0 (0.004)	0 (0)	0.007 (0.016)	0.039 (0.028)	0 (0.002)	0 (0.003)	0 (0.004)	0 (0)	0.031 (0.027)	0 (0)	0 (0.014)	0.29 (0.034)	0.108 (0.062)	0.26 (0.048)	0.134 (0.028)
ID0023558	O‘AHU	0.024 (0.02)	0.012 (0.018)	0.017 (0.007)	0.013 (0.017)	0.009 (0.013)	0 (0.014)	0 (0.008)	0 (0.003)	0 (0)	0 (0)	0 (0.012)	0 (0.012)	0 (0.006)	0 (0.004)	0 (0.004)	0 (0.001)	0.044 (0.027)	0 (0.01)	0 (0.006)	0.325 (0.026)	0.133 (0.051)	0.295 (0.041)	0.128 (0.028)
ID0023559	O‘AHU	0.047 (0.026)	0.028 (0.019)	0.025 (0.007)	0.012 (0.013)	0 (0.005)	0.1 (0.033)	0.027 (0.019)	0.004 (0.036)	0 (0.004)	0 (0)	0.049 (0.02)	0.043 (0.034)	0 (0.017)	0 (0.012)	0 (0.005)	0 (0.006)	0 (0.007)	0 (0.004)	0.035 (0.023)	0.287 (0.033)	0 (0.027)	0.203 (0.038)	0.138 (0.023)
ID0023561	O‘AHU	0.051 (0.025)	0.034 (0.021)	0.021 (0.006)	0.005 (0.012)	0 (0.006)	0.059 (0.026)	0 (0.009)	0 (0.019)	0 (0.002)	0 (0)	0.007 (0.012)	0 (0.012)	0 (0.015)	0 (0.01)	0 (0.001)	0 (0.004)	0.033 (0.021)	0 (0.004)	0 (0.013)	0.357 (0.03)	0.023 (0.035)	0.268 (0.036)	0.141 (0.023)
ID0023562	O‘AHU	0.017 (0.019)	0.008 (0.016)	0.013 (0.006)	0.011 (0.014)	0.016 (0.015)	0.042 (0.023)	0 (0.011)	0.049 (0.037)	0 (0.003)	0 (0.003)	0.004 (0.013)	0.06 (0.028)	0 (0.008)	0 (0.003)	0 (0.004)	0 (0)	0 (0.01)	0 (0.001)	0 (0.009)	0.38 (0.031)	0 (0.025)	0.288 (0.035)	0.111 (0.024)
ID0023563	O‘AHU	0 (0.014)	0.033 (0.02)	0.007 (0.005)	0.004 (0.011)	0 (0.007)	0.011 (0.022)	0 (0.006)	0 (0.006)	0 (0)	0 (0.004)	0.007 (0.016)	0 (0.011)	0 (0.016)	0 (0.002)	0.01 (0.007)	0 (0)	0.055 (0.028)	0 (0.008)	0 (0.001)	0.326 (0.03)	0.142 (0.059)	0.314 (0.04)	0.09 (0.024)
ID0023564	O‘AHU	0.019 (0.022)	0.02 (0.017)	0.013 (0.007)	0.011 (0.016)	0.015 (0.012)	0.033 (0.024)	0 (0.003)	0 (0)	0 (0)	0 (0)	0 (0.007)	0.031 (0.028)	0 (0.005)	0 (0)	0.006 (0.007)	0 (0.002)	0.038 (0.027)	0 (0.001)	0 (0.003)	0.266 (0.026)	0.093 (0.046)	0.348 (0.039)	0.106 (0.023)
ID0023565	O‘AHU	0.033 (0.026)	0.016 (0.018)	0.001 (0.004)	0.034 (0.019)	0 (0)	0.02 (0.025)	0 (0.004)	0 (0.009)	0 (0)	0 (0.006)	0.014 (0.019)	0.016 (0.024)	0 (0.001)	0 (0)	0.001 (0.006)	0 (0)	0.057 (0.024)	0 (0.006)	0 (0.002)	0.282 (0.032)	0.109 (0.057)	0.301 (0.043)	0.117 (0.023)
ID0023567	O‘AHU	0.01 (0.017)	0.027 (0.02)	0.011 (0.007)	0.022 (0.019)	0.007 (0.01)	0.016 (0.021)	0 (0.004)	0 (0)	0 (0)	0 (0)	0 (0.011)	0 (0.017)	0 (0.005)	0 (0)	0 (0.006)	0 (0.002)	0.044 (0.025)	0 (0.007)	0 (0.003)	0.32 (0.027)	0.122 (0.053)	0.31 (0.04)	0.111 (0.022)
ID0023570	O‘AHU	0.01 (0.019)	0.016 (0.018)	0.01 (0.006)	0 (0.006)	0.02 (0.016)	0.091 (0.034)	0 (0.018)	0 (0.021)	0.016 (0.021)	0 (0.001)	0 (0.01)	0 (0.015)	0 (0.007)	0 (0.005)	0 (0.005)	0 (0.001)	0.09 (0.03)	0 (0.002)	0 (0.007)	0.267 (0.03)	0.104 (0.063)	0.242 (0.046)	0.134 (0.028)
ID0023573	O‘AHU	0 (0.009)	0.017 (0.018)	0.019 (0.007)	0.002 (0.01)	0 (0.01)	0.077 (0.033)	0 (0.011)	0 (0.016)	0 (0.008)	0 (0.002)	0 (0.013)	0 (0.015)	0 (0)	0 (0.008)	0 (0.005)	0 (0)	0.074 (0.033)	0 (0)	0.016 (0.028)	0.288 (0.036)	0.062 (0.047)	0.291 (0.045)	0.154 (0.025)

ID0023574	OʻAHU	0 (0.008)	0.02 (0.018)	0.016 (0.007)	0.007 (0.013)	0.014 (0.014)	0.062 (0.034)	0.014 (0.018)	0.001 (0.034)	0 (0.003)	0 (0.001)	0.022 (0.02)	0.002 (0.017)	0 (0.003)	0 (0.003)	0 (0.005)	0 (0.001)	0.057 (0.029)	0 (0)	0 (0.018)	0.31 (0.038)	0.079 (0.054)	0.239 (0.044)	0.157 (0.029)
ID0023575	OʻAHU	0.018 (0.02)	0.024 (0.02)	0.005 (0.006)	0.05 (0.02)	0 (0.008)	0 (0.01)	0 (0.006)	0 (0)	0 (0)	0 (0)	0 (0.01)	0.011 (0.024)	0 (0.011)	0 (0)	0.001 (0.005)	0 (0.005)	0.043 (0.027)	0.001 (0.011)	0 (0.01)	0.292 (0.026)	0.133 (0.046)	0.297 (0.04)	0.125 (0.021)
ID0023578	KAUAʻI	0.022 (0.022)	0.023 (0.016)	0.022 (0.007)	0.03 (0.017)	0.02 (0.014)	0.055 (0.025)	0.036 (0.021)	0 (0.006)	0 (0)	0 (0.001)	0.02 (0.021)	0.009 (0.019)	0 (0)	0 (0)	0 (0.005)	0 (0.003)	0.054 (0.027)	0 (0)	0 (0)	0.331 (0.034)	0.013 (0.046)	0.27 (0.042)	0.093 (0.023)
ID0023579	KAUAʻI	0 (0.005)	0.007 (0.013)	0.016 (0.008)	0.061 (0.021)	0.031 (0.017)	0.066 (0.028)	0 (0.01)	0 (0)	0 (0)	0 (0)	0.02 (0.018)	0.032 (0.022)	0 (0)	0 (0)	0 (0.004)	0 (0)	0.065 (0.035)	0 (0)	0 (0.018)	0.251 (0.032)	0.043 (0.043)	0.313 (0.041)	0.095 (0.026)
ID0023580	KAUAʻI	0.013 (0.016)	0 (0.006)	0.014 (0.007)	0.069 (0.017)	0.023 (0.017)	0 (0.006)	0.015 (0.014)	0.002 (0.026)	0 (0.004)	0 (0.002)	0.009 (0.014)	0 (0.01)	0 (0.003)	0 (0)	0.009 (0.008)	0 (0.002)	0.054 (0.024)	0 (0)	0 (0.01)	0.221 (0.027)	0.098 (0.039)	0.362 (0.036)	0.111 (0.023)
ID0023581	KAUAʻI	0.007 (0.019)	0 (0.006)	0.016 (0.008)	0.044 (0.019)	0.041 (0.015)	0.07 (0.031)	0 (0)	0 (0)	0 (0)	0 (0.006)	0.008 (0.014)	0 (0.01)	0 (0)	0 (0)	0 (0.004)	0 (0.003)	0.08 (0.028)	0 (0.002)	0 (0.006)	0.282 (0.033)	0.021 (0.033)	0.288 (0.04)	0.14 (0.024)
ID0023582	KAUAʻI	0.001 (0.015)	0.006 (0.013)	0.023 (0.007)	0.03 (0.02)	0.032 (0.014)	0.053 (0.024)	0.015 (0.016)	0 (0.011)	0 (0)	0 (0.008)	0 (0.006)	0.018 (0.018)	0 (0.001)	0 (0.001)	0 (0.004)	0.001 (0.007)	0.123 (0.027)	0 (0.005)	0 (0.005)	0.206 (0.029)	0.052 (0.045)	0.325 (0.041)	0.116 (0.023)
ID0023583	KAUAʻI	0.035 (0.022)	0 (0.01)	0.009 (0.008)	0.062 (0.024)	0.01 (0.013)	0.028 (0.022)	0.014 (0.02)	0 (0.004)	0 (0)	0 (0)	0.03 (0.02)	0.012 (0.02)	0 (0)	0 (0.004)	0 (0.005)	0 (0.001)	0.077 (0.031)	0 (0)	0.043 (0.031)	0.214 (0.032)	0.02 (0.042)	0.324 (0.04)	0.121 (0.021)
ID0023584	KAUAʻI	0 (0.011)	0.011 (0.014)	0.014 (0.008)	0.077 (0.018)	0.003 (0.008)	0.025 (0.02)	0 (0.011)	0 (0.006)	0 (0)	0 (0)	0.034 (0.022)	0.016 (0.02)	0 (0)	0 (0.001)	0 (0.003)	0 (0.002)	0.054 (0.026)	0 (0)	0 (0)	0.259 (0.027)	0.092 (0.049)	0.311 (0.042)	0.105 (0.024)
ID0023585	KAUAʻI	0.011 (0.017)	0.016 (0.015)	0.01 (0.008)	0.06 (0.02)	0.007 (0.011)	0.016 (0.015)	0 (0.005)	0 (0.015)	0 (0)	0 (0.003)	0.041 (0.021)	0.031 (0.024)	0 (0)	0 (0.002)	0 (0.002)	0 (0.002)	0.075 (0.027)	0 (0)	0 (0)	0.271 (0.028)	0.026 (0.039)	0.33 (0.041)	0.108 (0.023)
ID0023586	KAUAʻI	0 (0.012)	0.021 (0.017)	0.016 (0.007)	0.041 (0.021)	0.037 (0.015)	0.02 (0.016)	0.026 (0.019)	0 (0)	0 (0)	0 (0)	0.022 (0.02)	0.002 (0.018)	0 (0)	0 (0)	0 (0.005)	0 (0.004)	0.103 (0.026)	0 (0.002)	0 (0)	0.226 (0.032)	0.088 (0.047)	0.303 (0.039)	0.095 (0.027)
ID0023590	OʻAHU	0.022 (0.022)	0.006 (0.014)	0.014 (0.006)	0.011 (0.012)	0.034 (0.015)	0.014 (0.018)	0.023 (0.026)	0.086 (0.052)	0 (0)	0 (0.006)	0.035 (0.017)	0.011 (0.023)	0 (0.015)	0 (0)	0.004 (0.007)	0 (0.005)	0.014 (0.022)	0 (0.006)	0.029 (0.021)	0.307 (0.028)	0.048 (0.047)	0.25 (0.048)	0.093 (0.025)
ID0023591	OʻAHU	0.036 (0.02)	0.065 (0.024)	0.013 (0.006)	0 (0.009)	0 (0.003)	0 (0.011)	0 (0.002)	0 (0.005)	0 (0)	0 (0.002)	0.016 (0.014)	0 (0.009)	0 (0.004)	0 (0)	0.006 (0.006)	0 (0.002)	0.029 (0.02)	0 (0)	0 (0.01)	0.34 (0.028)	0.051 (0.042)	0.286 (0.035)	0.159 (0.022)
ID0023592	OʻAHU	0 (0.003)	0.02 (0.016)	0.01 (0.008)	0.049 (0.021)	0 (0.006)	0.034 (0.025)	0 (0.008)	0.081 (0.051)	0 (0)	0 (0.004)	0.003 (0.013)	0 (0.007)	0.029 (0.031)	0 (0.015)	0.013 (0.008)	0 (0.003)	0.026 (0.031)	0 (0.004)	0.031 (0.028)	0.339 (0.032)	0 (0.033)	0.246 (0.041)	0.119 (0.022)
ID0023593	OʻAHU	0 (0.014)	0.025 (0.017)	0.022 (0.006)	0.023 (0.016)	0.01 (0.013)	0.056 (0.025)	0.031 (0.022)	0 (0.015)	0 (0.004)	0 (0)	0 (0.006)	0 (0.015)	0 (0.019)	0 (0.004)	0.01 (0.008)	0 (0.004)	0.028 (0.018)	0 (0.005)	0 (0.007)	0.355 (0.025)	0.077 (0.041)	0.234 (0.04)	0.132 (0.023)
ID0023596	OʻAHU	0.014 (0.02)	0.003 (0.014)	0.018 (0.007)	0 (0.007)	0 (0.008)	0.07 (0.029)	0 (0.003)	0 (0.011)	0 (0)	0 (0.013)	0 (0.009)	0.03 (0.023)	0 (0.002)	0 (0.014)	0.014 (0.008)	0 (0.002)	0.005 (0.015)	0 (0.007)	0 (0.011)	0.353 (0.025)	0.122 (0.051)	0.259 (0.039)	0.111 (0.024)
ID0023597	OʻAHU	0.045 (0.025)	0.015 (0.019)	0.005 (0.005)	0.003 (0.011)	0.001 (0.01)	0.053 (0.031)	0 (0.009)	0 (0.006)	0 (0.014)	0.031 (0.026)	0.006 (0.016)	0 (0.016)	0 (0.013)	0 (0)	0.001 (0.005)	0 (0.005)	0.114 (0.027)	0 (0.011)	0 (0.002)	0.196 (0.031)	0.213 (0.049)	0.21 (0.04)	0.108 (0.023)
ID0024820	HAWAIʻI	0 (0.005)	0.036 (0.021)	0.008 (0.005)	0.005 (0.011)	0.022 (0.013)	0.067 (0.029)	0 (0.005)	0 (0.021)	0 (0)	0 (0)	0.013 (0.018)	0 (0.01)	0.029 (0.03)	0 (0.006)	0.012 (0.008)	0 (0.006)	0.044 (0.032)	0 (0.007)	0.064 (0.032)	0.132 (0.026)	0.131 (0.054)	0.322 (0.039)	0.114 (0.023)
ID0024821	HAWAIʻI	0 (0.012)	0.034 (0.017)	0.008 (0.006)	0.036 (0.017)	0.024 (0.013)	0.075 (0.026)	0 (0.005)	0 (0.006)	0 (0.003)	0 (0.003)	0 (0.01)	0 (0.01)	0 (0.007)	0 (0.001)	0 (0.005)	0.007 (0.008)	0.109 (0.023)	0 (0.003)	0 (0.015)	0.151 (0.025)	0.084 (0.041)	0.331 (0.028)	0.14 (0.02)
ID0024822	HAWAIʻI	0.013 (0.016)	0.03 (0.019)	0.018 (0.006)	0 (0.007)	0.009 (0.011)	0.057 (0.025)	0 (0)	0 (0.004)	0 (0.004)	0 (0.004)	0.02 (0.019)	0 (0.008)	0 (0.015)	0 (0.002)	0.012 (0.008)	0 (0.001)	0.052 (0.037)	0 (0)	0.06 (0.035)	0.193 (0.029)	0.087 (0.055)	0.309 (0.038)	0.139 (0.024)

ID0024824	O‘AHU	0.01 (0.017)	0.03 (0.019)	0.006 (0.005)	0.008 (0.015)	0.001 (0.008)	0 (0.016)	0 (0)	0 (0)	0 (0.001)	0 (0)	0.033 (0.022)	0 (0.008)	0 (0.002)	0 (0.001)	0.012 (0.008)	0 (0.002)	0.026 (0.028)	0 (0.005)	0.019 (0.024)	0.305 (0.033)	0.171 (0.055)	0.279 (0.04)	0.1 (0.025)
ID0024825	O‘AHU	0.018 (0.02)	0.005 (0.015)	0.004 (0.005)	0.047 (0.018)	0.021 (0.012)	0.027 (0.024)	0 (0.008)	0 (0.018)	0 (0)	0.035 (0.022)	0.031 (0.019)	0 (0.008)	0 (0)	0 (0.004)	0 (0.006)	0 (0.001)	0.044 (0.022)	0 (0.003)	0 (0.002)	0.29 (0.031)	0.093 (0.046)	0.28 (0.042)	0.105 (0.023)
ID0024826	O‘AHU	0.033 (0.023)	0.023 (0.016)	0.01 (0.007)	0.016 (0.017)	0.011 (0.011)	0.057 (0.029)	0 (0)	0 (0)	0 (0)	0 (0)	0.008 (0.014)	0.043 (0.027)	0 (0.012)	0 (0.002)	0.017 (0.009)	0 (0.001)	0.017 (0.026)	0 (0)	0 (0.013)	0.248 (0.031)	0.053 (0.046)	0.36 (0.038)	0.105 (0.027)
ID0024827	O‘AHU	0.032 (0.023)	0.006 (0.015)	0.004 (0.006)	0.041 (0.017)	0.025 (0.013)	0.036 (0.026)	0 (0.005)	0 (0.007)	0 (0)	0.002 (0.018)	0.042 (0.021)	0 (0.015)	0.014 (0.023)	0 (0.004)	0 (0.002)	0 (0.002)	0.054 (0.023)	0 (0.001)	0 (0)	0.258 (0.03)	0.118 (0.057)	0.261 (0.042)	0.107 (0.023)
ID0024846	O‘AHU	0.041 (0.026)	0.023 (0.019)	0.012 (0.007)	0.009 (0.012)	0.01 (0.011)	0.079 (0.029)	0.006 (0.013)	0.024 (0.033)	0 (0)	0 (0.002)	0.005 (0.012)	0.029 (0.021)	0 (0.014)	0 (0.009)	0 (0.001)	0 (0.002)	0.035 (0.028)	0 (0.003)	0 (0.007)	0.355 (0.03)	0.031 (0.036)	0.215 (0.036)	0.126 (0.024)
ID0024848	O‘AHU	0.023 (0.025)	0.021 (0.017)	0.013 (0.007)	0.026 (0.017)	0 (0.009)	0.036 (0.023)	0.005 (0.017)	0 (0.001)	0 (0.001)	0 (0.002)	0.004 (0.013)	0.043 (0.026)	0 (0.01)	0 (0.002)	0.001 (0.006)	0 (0.001)	0.009 (0.016)	0 (0.004)	0.005 (0.019)	0.322 (0.029)	0.035 (0.044)	0.305 (0.041)	0.152 (0.024)
ID0024849	O‘AHU	0.054 (0.029)	0.01 (0.013)	0.018 (0.007)	0.005 (0.009)	0.014 (0.013)	0 (0.01)	0 (0.003)	0 (0.005)	0 (0)	0.008 (0.02)	0.084 (0.025)	0 (0.004)	0.022 (0.022)	0 (0.002)	0.003 (0.006)	0 (0.001)	0.003 (0.015)	0 (0.004)	0.014 (0.017)	0.34 (0.031)	0.021 (0.037)	0.292 (0.04)	0.112 (0.026)
ID0024850	O‘AHU	0.027 (0.022)	0 (0.008)	0.001 (0.005)	0.031 (0.02)	0.002 (0.008)	0.047 (0.029)	0 (0.007)	0 (0.017)	0 (0)	0 (0.009)	0.015 (0.019)	0.007 (0.02)	0 (0.006)	0 (0.001)	0.001 (0.006)	0 (0)	0.032 (0.022)	0 (0.006)	0 (0.006)	0.284 (0.026)	0.135 (0.048)	0.306 (0.039)	0.112 (0.026)
ID0024857	O‘AHU	0.002 (0.015)	0 (0.008)	0.016 (0.006)	0 (0.003)	0.026 (0.014)	0 (0.006)	0 (0.009)	0 (0.016)	0 (0.006)	0.008 (0.016)	0.012 (0.016)	0 (0.016)	0 (0.01)	0 (0.002)	0.003 (0.006)	0.012 (0.01)	0.041 (0.025)	0 (0.009)	0 (0.013)	0.334 (0.03)	0.227 (0.049)	0.232 (0.04)	0.085 (0.026)
ID0024858	O‘AHU	0.042 (0.023)	0.022 (0.016)	0.009 (0.007)	0.021 (0.013)	0.007 (0.012)	0.002 (0.014)	0.031 (0.023)	0.054 (0.048)	0 (0.001)	0 (0.004)	0 (0.008)	0 (0.02)	0 (0)	0 (0.007)	0.005 (0.006)	0 (0.007)	0 (0.016)	0 (0.005)	0.029 (0.02)	0.382 (0.029)	0.023 (0.041)	0.256 (0.037)	0.118 (0.023)
ID0024859	O‘AHU	0 (0.013)	0.024 (0.018)	0.011 (0.007)	0.031 (0.018)	0.009 (0.01)	0.03 (0.02)	0 (0.003)	0 (0.021)	0 (0.001)	0 (0.005)	0 (0.01)	0.039 (0.026)	0 (0.015)	0.006 (0.014)	0.003 (0.005)	0 (0.004)	0.039 (0.025)	0 (0.002)	0 (0.008)	0.307 (0.029)	0.186 (0.05)	0.223 (0.041)	0.092 (0.022)
ID0024869	KAUA‘I	0.035 (0.022)	0.014 (0.015)	0.016 (0.007)	0.044 (0.02)	0.028 (0.015)	0.052 (0.024)	0.025 (0.02)	0 (0.007)	0 (0)	0 (0.002)	0 (0.006)	0.028 (0.023)	0 (0.006)	0 (0.001)	0.001 (0.006)	0 (0.003)	0.094 (0.03)	0 (0.001)	0 (0)	0.216 (0.029)	0.072 (0.049)	0.289 (0.039)	0.085 (0.022)
ID0024870	KAUA‘I	0.043 (0.028)	0 (0.011)	0.016 (0.008)	0.066 (0.023)	0.033 (0.017)	0.039 (0.027)	0.044 (0.027)	0.036 (0.042)	0 (0)	0 (0.015)	0 (0.006)	0 (0.007)	0 (0.001)	0 (0.012)	0.005 (0.008)	0 (0.006)	0.094 (0.032)	0 (0.001)	0 (0)	0.216 (0.034)	0.005 (0.04)	0.319 (0.051)	0.084 (0.028)
ID0024871	KAUA‘I	0.014 (0.021)	0 (0.007)	0.028 (0.008)	0.052 (0.016)	0.008 (0.01)	0.028 (0.021)	0 (0.01)	0 (0.006)	0 (0.001)	0 (0)	0 (0.007)	0.024 (0.021)	0 (0.009)	0 (0)	0.012 (0.009)	0 (0.003)	0.07 (0.027)	0 (0)	0 (0)	0.245 (0.03)	0.084 (0.045)	0.328 (0.044)	0.107 (0.021)
ID0024872	KAUA‘I	0 (0.011)	0.018 (0.015)	0.02 (0.008)	0.044 (0.018)	0.026 (0.014)	0.027 (0.019)	0 (0.005)	0 (0.007)	0 (0)	0 (0)	0.068 (0.02)	0.005 (0.02)	0 (0)	0 (0.001)	0 (0.002)	0.004 (0.007)	0.088 (0.027)	0 (0)	0 (0.004)	0.25 (0.031)	0.017 (0.035)	0.32 (0.036)	0.112 (0.027)
ID0024873	KAUA‘I	0.003 (0.015)	0 (0.005)	0.022 (0.009)	0.069 (0.017)	0.014 (0.013)	0.038 (0.023)	0.013 (0.014)	0 (0.009)	0 (0.002)	0 (0)	0.003 (0.014)	0.039 (0.025)	0 (0.004)	0 (0)	0.013 (0.008)	0 (0.003)	0.048 (0.025)	0 (0)	0 (0.004)	0.239 (0.027)	0.01 (0.029)	0.395 (0.04)	0.094 (0.024)
ID0024874	KAUA‘I	0 (0.012)	0.002 (0.014)	0.029 (0.008)	0.058 (0.021)	0.017 (0.013)	0.048 (0.028)	0.031 (0.023)	0 (0.007)	0 (0)	0 (0)	0 (0)	0.027 (0.026)	0 (0)	0 (0.001)	0.007 (0.007)	0 (0.005)	0.051 (0.029)	0 (0.008)	0 (0.005)	0.241 (0.032)	0 (0.015)	0.339 (0.039)	0.149 (0.022)
ID0024875	KAUA‘I	0 (0.004)	0 (0.007)	0.016 (0.007)	0.074 (0.017)	0.017 (0.014)	0.054 (0.023)	0 (0.011)	0 (0.018)	0 (0.001)	0 (0)	0 (0.008)	0.078 (0.024)	0 (0.006)	0 (0.003)	0.008 (0.009)	0 (0.002)	0.022 (0.025)	0 (0.002)	0 (0)	0.238 (0.026)	0.05 (0.037)	0.316 (0.039)	0.126 (0.023)
ID0024876	KAUA‘I	0 (0.001)	0.012 (0.014)	0.012 (0.007)	0.067 (0.023)	0.022 (0.016)	0.033 (0.025)	0.009 (0.018)	0 (0.026)	0 (0.001)	0 (0)	0.029 (0.021)	0.066 (0.026)	0 (0)	0 (0.003)	0.003 (0.007)	0 (0)	0.004 (0.014)	0 (0)	0 (0.01)	0.278 (0.028)	0.04 (0.044)	0.285 (0.04)	0.139 (0.023)
ID0024877	KAUA‘I	0 (0.005)	0.005 (0.013)	0.014 (0.008)	0.059 (0.023)	0.026 (0.017)	0.065 (0.024)	0 (0.009)	0 (0)	0 (0.001)	0 (0)	0.019 (0.019)	0.045 (0.025)	0 (0)	0 (0)	0 (0.005)	0 (0.001)	0.046 (0.026)	0 (0)	0 (0.022)	0.244 (0.03)	0.017 (0.035)	0.339 (0.039)	0.121 (0.022)

ID0024879	KAUA'I	0 (0.009)	0 (0.01)	0.01 (0.007)	0.044 (0.016)	0.018 (0.016)	0.039 (0.026)	0.016 (0.02)	0 (0.006)	0 (0)	0 (0)	0.01 (0.017)	0.035 (0.026)	0 (0.006)	0 (0.006)	0.009 (0.008)	0 (0.004)	0.02 (0.019)	0 (0.001)	0 (0.014)	0.271 (0.025)	0.08 (0.051)	0.323 (0.044)	0.126 (0.024)
ID0024880	KAUA'I	0 (0.012)	0 (0.008)	0.022 (0.01)	0.052 (0.021)	0.017 (0.013)	0.006 (0.015)	0 (0.008)	0 (0)	0 (0)	0 (0)	0.01 (0.016)	0 (0.008)	0 (0.002)	0 (0.001)	0.021 (0.008)	0 (0.005)	0.076 (0.027)	0 (0)	0 (0)	0.226 (0.03)	0.144 (0.043)	0.31 (0.039)	0.116 (0.023)
ID0024884	O'AHU	0 (0.014)	0.028 (0.021)	0.02 (0.007)	0.021 (0.017)	0.021 (0.017)	0.005 (0.014)	0.024 (0.022)	0.097 (0.054)	0 (0)	0 (0.016)	0.028 (0.021)	0 (0.014)	0.005 (0.018)	0 (0)	0.002 (0.006)	0 (0.001)	0.017 (0.021)	0.01 (0.013)	0 (0.01)	0.346 (0.033)	0.068 (0.043)	0.222 (0.036)	0.086 (0.026)
ID0024885	O'AHU	0.026 (0.023)	0.031 (0.022)	0.02 (0.007)	0.014 (0.014)	0.004 (0.013)	0.019 (0.018)	0.035 (0.025)	0 (0.028)	0 (0)	0 (0.008)	0.03 (0.021)	0.001 (0.015)	0 (0.003)	0 (0.002)	0 (0.003)	0 (0.001)	0 (0.006)	0 (0.004)	0 (0.011)	0.352 (0.028)	0.114 (0.057)	0.226 (0.046)	0.128 (0.028)
ID0024892	MAUI	0.005 (0.018)	0 (0.002)	0.008 (0.006)	0.04 (0.02)	0.012 (0.014)	0.021 (0.02)	0 (0.011)	0 (0.011)	0.026 (0.02)	0 (0.007)	0.049 (0.022)	0.024 (0.024)	0 (0.002)	0 (0)	0 (0.004)	0 (0)	0 (0.014)	0 (0)	0.022 (0.021)	0.143 (0.027)	0.247 (0.054)	0.301 (0.041)	0.101 (0.024)
ID0024893	O'AHU	0.058 (0.025)	0.03 (0.017)	0.021 (0.007)	0 (0.003)	0.013 (0.014)	0.037 (0.023)	0 (0.001)	0 (0.004)	0 (0.003)	0 (0.003)	0.035 (0.019)	0.014 (0.023)	0 (0.012)	0 (0.001)	0.015 (0.01)	0 (0.001)	0.012 (0.016)	0 (0.002)	0 (0.009)	0.314 (0.029)	0 (0.023)	0.317 (0.036)	0.135 (0.025)
ID0024894	O'AHU	0.06 (0.024)	0 (0.009)	0.005 (0.006)	0.066 (0.018)	0.016 (0.013)	0.05 (0.023)	0 (0.011)	0.018 (0.032)	0 (0)	0 (0.01)	0.05 (0.022)	0.018 (0.021)	0 (0.001)	0 (0.003)	0 (0.005)	0 (0.001)	0.039 (0.022)	0 (0)	0 (0.008)	0.27 (0.03)	0.077 (0.046)	0.226 (0.037)	0.104 (0.023)
ID0024896	O'AHU	0.048 (0.022)	0.03 (0.02)	0.011 (0.006)	0.027 (0.018)	0.014 (0.015)	0.007 (0.017)	0.011 (0.016)	0.021 (0.042)	0 (0)	0 (0.003)	0.028 (0.017)	0.058 (0.029)	0 (0.008)	0 (0)	0.002 (0.006)	0 (0.001)	0.022 (0.025)	0 (0.001)	0 (0)	0.283 (0.027)	0.09 (0.052)	0.235 (0.04)	0.113 (0.025)
ID0024897	O'AHU	0.021 (0.018)	0.03 (0.022)	0.014 (0.007)	0.028 (0.016)	0.022 (0.016)	0 (0.006)	0.003 (0.013)	0.035 (0.038)	0 (0.001)	0 (0.002)	0.015 (0.017)	0 (0.009)	0 (0.015)	0 (0.005)	0.006 (0.008)	0 (0)	0.052 (0.023)	0 (0.002)	0 (0.007)	0.326 (0.031)	0.106 (0.049)	0.248 (0.038)	0.094 (0.032)
ID0024898	O'AHU	0.054 (0.03)	0 (0.002)	0.016 (0.008)	0.054 (0.02)	0 (0.008)	0.043 (0.027)	0.001 (0.014)	0.012 (0.033)	0 (0)	0 (0.003)	0.05 (0.023)	0.037 (0.028)	0 (0.022)	0 (0.004)	0.01 (0.009)	0 (0.001)	0.023 (0.027)	0 (0)	0 (0)	0.242 (0.031)	0.062 (0.055)	0.281 (0.05)	0.116 (0.024)
ID0024899	O'AHU	0.052 (0.024)	0.021 (0.02)	0.024 (0.007)	0.011 (0.015)	0.016 (0.015)	0.041 (0.029)	0.04 (0.026)	0.023 (0.039)	0 (0)	0 (0.005)	0.025 (0.019)	0 (0.002)	0 (0.008)	0.001 (0.011)	0 (0.005)	0 (0.007)	0.005 (0.021)	0 (0.005)	0.016 (0.022)	0.385 (0.028)	0 (0.028)	0.216 (0.035)	0.124 (0.026)
ID0024902	O'AHU	0 (0.016)	0.029 (0.018)	0 (0.005)	0.049 (0.017)	0.025 (0.016)	0.08 (0.029)	0 (0.007)	0.033 (0.052)	0 (0.003)	0 (0.002)	0.007 (0.015)	0.05 (0.028)	0.014 (0.022)	0 (0)	0 (0.003)	0 (0.004)	0 (0.014)	0 (0.009)	0 (0.014)	0.346 (0.029)	0.094 (0.059)	0.208 (0.047)	0.065 (0.025)
ID0024904	O'AHU	0 (0.02)	0.014 (0.017)	0.012 (0.007)	0.016 (0.016)	0.013 (0.014)	0.044 (0.026)	0 (0.005)	0 (0.008)	0 (0.004)	0 (0)	0.049 (0.022)	0 (0.016)	0 (0.013)	0 (0.004)	0.007 (0.008)	0 (0.001)	0.084 (0.026)	0 (0.003)	0 (0.006)	0.227 (0.033)	0.112 (0.061)	0.297 (0.044)	0.124 (0.027)
ID0024905	O'AHU	0.045 (0.029)	0.021 (0.016)	0.01 (0.007)	0.021 (0.016)	0.028 (0.018)	0.005 (0.018)	0 (0.002)	0 (0.013)	0 (0)	0 (0.006)	0.046 (0.021)	0 (0.016)	0 (0)	0 (0.002)	0 (0.006)	0 (0.002)	0.093 (0.03)	0 (0.005)	0 (0.018)	0.223 (0.033)	0.143 (0.051)	0.252 (0.041)	0.112 (0.03)
ID0024906	O'AHU	0.025 (0.025)	0.025 (0.019)	0.012 (0.007)	0.022 (0.017)	0.021 (0.014)	0.034 (0.023)	0 (0.003)	0 (0.013)	0 (0.001)	0 (0.002)	0.018 (0.017)	0.016 (0.022)	0 (0.013)	0 (0.002)	0.008 (0.007)	0.003 (0.006)	0.071 (0.033)	0 (0.002)	0 (0.016)	0.247 (0.035)	0.107 (0.052)	0.276 (0.037)	0.116 (0.024)
ID0024907	O'AHU	0.033 (0.026)	0.01 (0.015)	0.013 (0.007)	0.031 (0.018)	0.036 (0.016)	0.01 (0.015)	0 (0.002)	0 (0.02)	0 (0.002)	0 (0)	0.01 (0.015)	0.005 (0.016)	0.012 (0.026)	0 (0.002)	0.006 (0.008)	0 (0)	0.027 (0.02)	0 (0.002)	0 (0.006)	0.309 (0.03)	0.106 (0.049)	0.26 (0.032)	0.131 (0.028)
ID0024908	O'AHU	0.001 (0.02)	0.048 (0.021)	0.014 (0.006)	0 (0.007)	0.011 (0.012)	0.013 (0.02)	0 (0.003)	0 (0.017)	0 (0.01)	0 (0)	0.005 (0.014)	0.057 (0.031)	0.007 (0.02)	0 (0.001)	0.01 (0.008)	0 (0.003)	0.064 (0.035)	0 (0.002)	0 (0.014)	0.22 (0.032)	0.176 (0.059)	0.264 (0.041)	0.111 (0.027)
ID0024909	O'AHU	0.003 (0.022)	0.018 (0.017)	0.011 (0.007)	0.02 (0.017)	0.019 (0.014)	0.028 (0.022)	0 (0.001)	0 (0.011)	0 (0.003)	0 (0.009)	0.031 (0.02)	0 (0.02)	0 (0.006)	0 (0.002)	0 (0.006)	0 (0.003)	0.078 (0.029)	0 (0.002)	0 (0.004)	0.254 (0.031)	0.187 (0.061)	0.242 (0.038)	0.109 (0.029)
ID0024910	O'AHU	0 (0.013)	0.035 (0.019)	0.003 (0.005)	0.02 (0.017)	0.01 (0.013)	0.027 (0.023)	0 (0.003)	0 (0.014)	0 (0)	0 (0.002)	0.044 (0.022)	0.048 (0.033)	0 (0.006)	0 (0.005)	0.005 (0.007)	0 (0.003)	0.062 (0.032)	0 (0.002)	0 (0.013)	0.232 (0.033)	0.147 (0.054)	0.251 (0.044)	0.116 (0.025)
ID0024915	O'AHU	0.03 (0.02)	0.005 (0.014)	0.011 (0.006)	0.002 (0.009)	0.034 (0.015)	0.032 (0.024)	0.021 (0.018)	0.017 (0.038)	0 (0)	0 (0.011)	0.001 (0.011)	0.022 (0.02)	0 (0.015)	0.02 (0.017)	0 (0.002)	0 (0.003)	0.038 (0.023)	0 (0.002)	0 (0.02)	0.372 (0.026)	0.064 (0.041)	0.229 (0.038)	0.1 (0.027)

ID0030524	O'AHU	0.058 (0.026)	0.037 (0.022)	0.016 (0.008)	0.039 (0.017)	0.003 (0.012)	0.049 (0.028)	0 (0.001)	0 (0.02)	0 (0.003)	0 (0)	0.036 (0.02)	0.041 (0.03)	0 (0.003)	0 (0.005)	0 (0.006)	0 (0.003)	0 (0.015)	0.007 (0.011)	0.01 (0.019)	0.28 (0.028)	0.042 (0.05)	0.276 (0.046)	0.106 (0.025)
ID0030525	O'AHU	0.009 (0.019)	0.056 (0.019)	0.014 (0.007)	0.013 (0.015)	0.019 (0.013)	0.05 (0.027)	0 (0.004)	0 (0.007)	0 (0)	0 (0.002)	0.029 (0.02)	0.052 (0.027)	0.019 (0.026)	0 (0.011)	0.013 (0.009)	0 (0.001)	0.006 (0.018)	0 (0.006)	0 (0.013)	0.325 (0.028)	0.016 (0.04)	0.284 (0.041)	0.096 (0.026)
ID0030526	O'AHU	0 (0.007)	0.005 (0.013)	0.025 (0.006)	0 (0.009)	0.013 (0.014)	0.032 (0.021)	0 (0.003)	0 (0.003)	0 (0)	0 (0.001)	0.05 (0.023)	0.051 (0.03)	0 (0.016)	0 (0.002)	0 (0.005)	0 (0)	0 (0.018)	0 (0.002)	0.017 (0.021)	0.353 (0.029)	0.09 (0.051)	0.274 (0.042)	0.092 (0.023)
ID0030527	O'AHU	0.046 (0.026)	0.01 (0.013)	0.005 (0.006)	0.009 (0.009)	0 (0.008)	0.015 (0.017)	0 (0.005)	0.017 (0.033)	0 (0)	0 (0.011)	0.035 (0.019)	0.02 (0.02)	0 (0.009)	0 (0.003)	0.001 (0.006)	0 (0.003)	0.045 (0.028)	0 (0.001)	0.007 (0.021)	0.316 (0.029)	0.074 (0.046)	0.284 (0.042)	0.116 (0.021)
ID0030528	O'AHU	0.045 (0.022)	0.009 (0.014)	0.02 (0.008)	0.022 (0.018)	0.012 (0.014)	0 (0.009)	0.034 (0.025)	0 (0.03)	0 (0)	0.004 (0.016)	0.043 (0.021)	0 (0.005)	0.013 (0.024)	0 (0.002)	0.004 (0.007)	0 (0.007)	0.036 (0.022)	0 (0.003)	0 (0.006)	0.346 (0.03)	0.015 (0.031)	0.269 (0.038)	0.13 (0.028)
ID0030529	O'AHU	0.037 (0.02)	0.02 (0.018)	0.012 (0.006)	0.028 (0.015)	0.02 (0.016)	0.019 (0.017)	0 (0.006)	0.038 (0.047)	0 (0.002)	0 (0.014)	0.016 (0.016)	0.036 (0.024)	0.003 (0.019)	0 (0.006)	0.012 (0.007)	0 (0.001)	0.002 (0.017)	0 (0.002)	0 (0.004)	0.308 (0.027)	0.052 (0.046)	0.26 (0.037)	0.137 (0.026)
ID0030530	O'AHU	0.087 (0.021)	0.013 (0.018)	0.02 (0.006)	0.009 (0.013)	0.005 (0.012)	0 (0.009)	0 (0.003)	0 (0.023)	0 (0.001)	0 (0.005)	0.003 (0.014)	0 (0.01)	0 (0.012)	0 (0.002)	0 (0.006)	0 (0.001)	0.013 (0.016)	0 (0)	0 (0.015)	0.384 (0.029)	0.08 (0.046)	0.254 (0.037)	0.131 (0.03)
ID0030531	O'AHU	0.033 (0.02)	0 (0.009)	0.014 (0.007)	0.031 (0.016)	0.029 (0.016)	0 (0.006)	0 (0.007)	0 (0.002)	0 (0)	0 (0.01)	0.028 (0.018)	0.015 (0.021)	0 (0.013)	0 (0)	0.013 (0.01)	0 (0.003)	0.005 (0.011)	0 (0.002)	0 (0.009)	0.325 (0.023)	0.172 (0.044)	0.216 (0.039)	0.119 (0.027)
ID0030532	O'AHU	0 (0.009)	0.033 (0.02)	0.023 (0.007)	0.014 (0.012)	0.031 (0.016)	0.025 (0.025)	0.001 (0.013)	0.088 (0.06)	0 (0)	0 (0.007)	0.03 (0.018)	0 (0.013)	0 (0.005)	0.044 (0.026)	0 (0.005)	0 (0.005)	0.036 (0.021)	0 (0.003)	0 (0.017)	0.333 (0.026)	0.027 (0.043)	0.216 (0.039)	0.098 (0.023)
ID0030537	O'AHU	0.012 (0.017)	0.057 (0.02)	0.016 (0.007)	0.026 (0.02)	0.014 (0.014)	0.046 (0.026)	0.015 (0.019)	0 (0.009)	0 (0)	0 (0)	0 (0.011)	0.052 (0.027)	0 (0)	0 (0.002)	0.004 (0.006)	0 (0.001)	0.023 (0.027)	0 (0.001)	0 (0.001)	0.291 (0.032)	0 (0.021)	0.321 (0.037)	0.124 (0.023)
ID0030540	O'AHU	0.017 (0.021)	0.055 (0.021)	0.013 (0.007)	0.029 (0.019)	0.017 (0.015)	0.041 (0.025)	0 (0.009)	0.027 (0.042)	0 (0.001)	0 (0.008)	0.016 (0.018)	0 (0.018)	0 (0.008)	0 (0.005)	0 (0.005)	0 (0.002)	0.041 (0.031)	0 (0.005)	0.018 (0.025)	0.275 (0.032)	0.087 (0.054)	0.259 (0.043)	0.105 (0.024)
ID0030541	O'AHU	0.01 (0.023)	0.043 (0.02)	0.009 (0.006)	0.031 (0.016)	0.015 (0.014)	0.022 (0.021)	0 (0)	0 (0.02)	0 (0.004)	0 (0)	0.027 (0.019)	0.049 (0.03)	0.014 (0.026)	0 (0.006)	0 (0.006)	0 (0.002)	0.031 (0.025)	0 (0.002)	0 (0.015)	0.265 (0.031)	0.057 (0.045)	0.307 (0.042)	0.121 (0.025)
ID0030542	O'AHU	0 (0.018)	0.057 (0.021)	0.023 (0.007)	0 (0.008)	0.009 (0.014)	0.06 (0.034)	0 (0.012)	0 (0.011)	0 (0.002)	0 (0)	0.004 (0.015)	0 (0.013)	0.013 (0.021)	0 (0.003)	0 (0.004)	0.001 (0.005)	0.05 (0.027)	0 (0.004)	0 (0.023)	0.276 (0.034)	0.145 (0.055)	0.242 (0.045)	0.121 (0.025)
ID0030543	O'AHU	0 (0.011)	0.032 (0.023)	0.019 (0.007)	0.002 (0.01)	0.022 (0.016)	0.049 (0.032)	0 (0.005)	0 (0.012)	0 (0.001)	0 (0.008)	0 (0.011)	0.033 (0.03)	0.003 (0.024)	0 (0.009)	0.006 (0.008)	0 (0.001)	0.028 (0.025)	0.003 (0.011)	0 (0.005)	0.274 (0.031)	0.165 (0.061)	0.261 (0.043)	0.105 (0.027)
ID0030545	O'AHU	0.004 (0.018)	0.025 (0.017)	0.011 (0.007)	0.049 (0.016)	0.017 (0.015)	0.077 (0.032)	0 (0.012)	0.001 (0.016)	0 (0.001)	0 (0.012)	0.021 (0.019)	0.024 (0.021)	0 (0.016)	0 (0.005)	0 (0.004)	0 (0.001)	0.014 (0.019)	0 (0.004)	0 (0.003)	0.313 (0.029)	0.085 (0.049)	0.259 (0.038)	0.101 (0.024)
ID0030546	O'AHU	0.017 (0.018)	0.051 (0.021)	0 (0.004)	0.042 (0.019)	0.006 (0.011)	0.072 (0.027)	0 (0)	0 (0.011)	0 (0)	0 (0)	0 (0.007)	0.063 (0.029)	0 (0.017)	0.016 (0.016)	0 (0.005)	0 (0.001)	0.029 (0.027)	0 (0.002)	0 (0.005)	0.296 (0.03)	0 (0.015)	0.278 (0.036)	0.129 (0.025)
ID0030547	O'AHU	0.012 (0.019)	0.037 (0.019)	0.008 (0.007)	0.035 (0.017)	0.008 (0.013)	0 (0.002)	0 (0.008)	0.129 (0.059)	0 (0)	0 (0.003)	0 (0.01)	0.009 (0.02)	0.035 (0.031)	0.019 (0.028)	0.01 (0.009)	0 (0.001)	0.033 (0.022)	0 (0.008)	0 (0.01)	0.298 (0.032)	0 (0.012)	0.241 (0.045)	0.124 (0.026)
ID0030548	O'AHU	0.004 (0.016)	0.027 (0.019)	0.011 (0.007)	0.036 (0.018)	0.003 (0.01)	0.057 (0.028)	0 (0)	0 (0.004)	0 (0)	0 (0.001)	0 (0.009)	0.064 (0.031)	0 (0.019)	0 (0.003)	0.005 (0.007)	0 (0.001)	0.053 (0.034)	0 (0.001)	0 (0.014)	0.251 (0.029)	0.032 (0.047)	0.329 (0.037)	0.127 (0.022)
ID0030549	O'AHU	0.122 (0.025)	0.007 (0.013)	0.006 (0.006)	0.027 (0.019)	0.012 (0.011)	0.037 (0.024)	0.036 (0.025)	0 (0.009)	0 (0.002)	0 (0.004)	0 (0.002)	0.067 (0.03)	0 (0.005)	0 (0.001)	0.007 (0.006)	0 (0.002)	0.002 (0.015)	0 (0.003)	0 (0.003)	0.245 (0.026)	0.111 (0.053)	0.226 (0.036)	0.095 (0.024)
ID0030550	O'AHU	0.001 (0.015)	0.015 (0.016)	0.018 (0.007)	0.026 (0.016)	0.011 (0.013)	0 (0.012)	0.015 (0.021)	0.038 (0.052)	0 (0.001)	0 (0.002)	0.003 (0.014)	0 (0.01)	0.006 (0.025)	0.052 (0.034)	0 (0.006)	0 (0.001)	0.05 (0.031)	0 (0.006)	0.032 (0.028)	0.311 (0.032)	0.05 (0.047)	0.215 (0.042)	0.157 (0.026)

ID0030551	O‘AHU	0.003 (0.014)	0.033 (0.022)	0.013 (0.006)	0.019 (0.014)	0.013 (0.012)	0.037 (0.024)	0 (0.012)	0 (0.007)	0 (0)	0 (0.006)	0.009 (0.016)	0 (0.011)	0 (0.007)	0 (0.009)	0.008 (0.007)	0.008 (0.009)	0.088 (0.025)	0 (0.004)	0 (0)	0.282 (0.027)	0.081 (0.049)	0.286 (0.036)	0.12 (0.023)
ID0030552	O‘AHU	0.074 (0.021)	0.046 (0.02)	0 (0.006)	0.047 (0.018)	0.013 (0.014)	0.02 (0.02)	0 (0.001)	0 (0.022)	0 (0.005)	0 (0.003)	0.009 (0.013)	0.007 (0.018)	0 (0.01)	0 (0.006)	0.016 (0.009)	0 (0)	0.007 (0.014)	0 (0)	0 (0.007)	0.309 (0.027)	0.041 (0.037)	0.299 (0.031)	0.113 (0.027)
ID0030554	O‘AHU	0.051 (0.022)	0.015 (0.016)	0.001 (0.005)	0.02 (0.014)	0.009 (0.011)	0.073 (0.028)	0 (0.01)	0 (0.003)	0.005 (0.013)	0 (0.008)	0.018 (0.017)	0.055 (0.029)	0 (0.013)	0 (0.009)	0 (0.001)	0 (0.005)	0.015 (0.02)	0 (0.003)	0 (0.007)	0.254 (0.03)	0.032 (0.035)	0.343 (0.038)	0.109 (0.021)
ID0030555	O‘AHU	0 (0.011)	0.018 (0.017)	0.018 (0.008)	0.029 (0.015)	0.021 (0.013)	0.048 (0.024)	0 (0)	0 (0.017)	0 (0.001)	0 (0.002)	0.02 (0.018)	0.014 (0.021)	0 (0.011)	0 (0.012)	0.007 (0.007)	0 (0.003)	0.05 (0.027)	0.01 (0.014)	0 (0.006)	0.277 (0.027)	0.11 (0.05)	0.293 (0.04)	0.084 (0.023)
ID0030556	O‘AHU	0.023 (0.023)	0.031 (0.02)	0.012 (0.007)	0.011 (0.012)	0.027 (0.014)	0.019 (0.023)	0 (0)	0 (0)	0 (0.004)	0 (0.002)	0 (0.006)	0 (0.006)	0 (0.017)	0 (0)	0.006 (0.006)	0.004 (0.008)	0 (0.018)	0 (0.002)	0.085 (0.031)	0.33 (0.027)	0.059 (0.044)	0.264 (0.044)	0.13 (0.024)
ID0030557	O‘AHU	0.06 (0.022)	0 (0.011)	0.024 (0.007)	0.032 (0.018)	0.016 (0.012)	0 (0.006)	0 (0.001)	0.01 (0.027)	0 (0.002)	0.006 (0.018)	0.037 (0.022)	0 (0.004)	0 (0.018)	0 (0.002)	0 (0.005)	0 (0)	0.014 (0.015)	0 (0.004)	0 (0.01)	0.343 (0.028)	0.198 (0.051)	0.175 (0.042)	0.086 (0.025)
ID0030558	O‘AHU	0.014 (0.019)	0.035 (0.021)	0.014 (0.006)	0.023 (0.015)	0.014 (0.013)	0.028 (0.023)	0 (0.004)	0 (0.009)	0 (0)	0 (0.008)	0 (0.007)	0.036 (0.02)	0 (0.003)	0 (0)	0.015 (0.009)	0 (0.004)	0.007 (0.016)	0 (0.001)	0 (0.01)	0.317 (0.026)	0.068 (0.044)	0.312 (0.035)	0.115 (0.022)
ID0030559	O‘AHU	0.031 (0.022)	0.055 (0.018)	0.019 (0.008)	0.027 (0.02)	0.015 (0.014)	0.053 (0.029)	0.031 (0.022)	0 (0)	0 (0.001)	0 (0.004)	0.002 (0.012)	0.033 (0.028)	0 (0.002)	0 (0.001)	0.005 (0.006)	0 (0)	0.041 (0.029)	0 (0.002)	0 (0.005)	0.258 (0.029)	0 (0.023)	0.282 (0.039)	0.148 (0.022)
ID0030560	O‘AHU	0.003 (0.019)	0.059 (0.02)	0.012 (0.007)	0.023 (0.02)	0 (0.006)	0.057 (0.027)	0.021 (0.019)	0 (0.006)	0 (0)	0 (0.001)	0.015 (0.015)	0.045 (0.027)	0 (0.01)	0 (0.002)	0.007 (0.007)	0 (0)	0.049 (0.028)	0 (0)	0 (0.01)	0.221 (0.031)	0.027 (0.04)	0.331 (0.038)	0.131 (0.021)
ID0030561	O‘AHU	0 (0.009)	0.04 (0.021)	0.012 (0.007)	0.036 (0.018)	0.003 (0.008)	0.055 (0.03)	0.009 (0.016)	0.002 (0.032)	0 (0)	0 (0.015)	0 (0.008)	0 (0.001)	0.005 (0.019)	0.026 (0.02)	0 (0.004)	0 (0.003)	0.017 (0.021)	0 (0.003)	0.023 (0.02)	0.349 (0.029)	0.071 (0.046)	0.239 (0.035)	0.113 (0.022)
ID0030563	O‘AHU	0.044 (0.025)	0 (0.01)	0.007 (0.005)	0.008 (0.012)	0.002 (0.011)	0.085 (0.027)	0.001 (0.011)	0 (0.004)	0 (0.006)	0 (0.003)	0.011 (0.012)	0 (0.019)	0.017 (0.025)	0 (0.002)	0 (0.002)	0 (0.001)	0 (0.016)	0 (0.003)	0.069 (0.03)	0.269 (0.03)	0.118 (0.052)	0.264 (0.039)	0.105 (0.024)
ID0030564	O‘AHU	0.062 (0.024)	0.002 (0.012)	0.012 (0.006)	0.014 (0.012)	0.021 (0.014)	0 (0.004)	0 (0.004)	0.018 (0.036)	0 (0)	0.031 (0.023)	0.011 (0.014)	0 (0.003)	0 (0.005)	0 (0.002)	0 (0.005)	0 (0.006)	0.038 (0.025)	0 (0.001)	0 (0.006)	0.34 (0.03)	0.102 (0.05)	0.252 (0.038)	0.098 (0.027)
ID0030565	O‘AHU	0.005 (0.014)	0.052 (0.021)	0.008 (0.007)	0.028 (0.017)	0.012 (0.013)	0 (0.012)	0 (0.007)	0.022 (0.036)	0 (0.002)	0 (0)	0.022 (0.017)	0.025 (0.023)	0 (0.004)	0 (0.004)	0 (0.002)	0 (0.003)	0.007 (0.019)	0 (0.006)	0.013 (0.016)	0.341 (0.029)	0.106 (0.05)	0.245 (0.039)	0.114 (0.025)
ID0030566	O‘AHU	0 (0.015)	0.024 (0.018)	0.025 (0.007)	0.011 (0.011)	0.025 (0.017)	0.08 (0.022)	0 (0)	0 (0.01)	0 (0)	0 (0.008)	0.017 (0.018)	0.048 (0.028)	0 (0.013)	0 (0)	0 (0.003)	0 (0.002)	0.028 (0.024)	0.01 (0.015)	0.003 (0.017)	0.272 (0.028)	0.139 (0.052)	0.176 (0.036)	0.141 (0.027)
ID0030569	O‘AHU	0.094 (0.031)	0.021 (0.019)	0.01 (0.006)	0.038 (0.021)	0 (0.004)	0.056 (0.028)	0 (0.008)	0 (0.005)	0 (0.006)	0 (0.008)	0.016 (0.019)	0.016 (0.021)	0 (0.016)	0 (0)	0.002 (0.006)	0.001 (0.007)	0.06 (0.032)	0 (0.005)	0.012 (0.022)	0.234 (0.032)	0.114 (0.057)	0.226 (0.042)	0.101 (0.022)
ID0030576	O‘AHU	0.006 (0.014)	0.032 (0.019)	0.011 (0.007)	0.021 (0.015)	0.01 (0.013)	0.061 (0.026)	0 (0.001)	0.083 (0.046)	0 (0)	0 (0.008)	0.027 (0.02)	0.032 (0.025)	0 (0.007)	0 (0.009)	0 (0.005)	0 (0)	0 (0.009)	0 (0)	0 (0.004)	0.351 (0.033)	0.018 (0.043)	0.267 (0.038)	0.081 (0.029)
ID0030577	O‘AHU	0.013 (0.02)	0.028 (0.022)	0.024 (0.006)	0 (0.006)	0.031 (0.016)	0.067 (0.028)	0.004 (0.015)	0 (0.001)	0 (0.002)	0 (0.003)	0 (0.008)	0.015 (0.021)	0.006 (0.028)	0 (0.001)	0.015 (0.009)	0 (0.003)	0.028 (0.023)	0 (0.006)	0 (0.007)	0.314 (0.024)	0.132 (0.053)	0.191 (0.043)	0.132 (0.028)
ID0030620	O‘AHU	0.095 (0.024)	0.022 (0.018)	0.016 (0.006)	0.019 (0.016)	0 (0.007)	0.045 (0.028)	0 (0.004)	0 (0.007)	0 (0.009)	0 (0.003)	0.003 (0.014)	0 (0.016)	0.026 (0.023)	0 (0)	0.014 (0.007)	0 (0.005)	0.032 (0.022)	0 (0.002)	0 (0.007)	0.256 (0.024)	0.084 (0.052)	0.263 (0.042)	0.125 (0.024)
ID0030621	O‘AHU	0.132 (0.03)	0.006 (0.015)	0.002 (0.004)	0.037 (0.018)	0 (0)	0.017 (0.02)	0 (0.002)	0 (0.001)	0 (0.004)	0 (0.005)	0.023 (0.021)	0 (0.016)	0 (0.011)	0 (0)	0.005 (0.007)	0 (0.003)	0.063 (0.026)	0 (0)	0 (0.003)	0.235 (0.032)	0.099 (0.054)	0.257 (0.042)	0.123 (0.024)
ID0034106	O‘AHU	0 (0.008)	0.009 (0.016)	0.022 (0.006)	0.011 (0.014)	0.013 (0.012)	0.03 (0.022)	0.004 (0.017)	0.058 (0.05)	0 (0)	0 (0.009)	0.024 (0.019)	0.028 (0.025)	0 (0.006)	0.024 (0.021)	0.007 (0.007)	0 (0.002)	0.022 (0.022)	0 (0.003)	0.003 (0.02)	0.344 (0.032)	0 (0.016)	0.278 (0.037)	0.123 (0.021)

ID0034108	O‘AHU	0.033 (0.024)	0.004 (0.013)	0.008 (0.007)	0.049 (0.015)	0.017 (0.013)	0.039 (0.025)	0 (0)	0 (0.003)	0 (0.002)	0 (0.017)	0.039 (0.018)	0 (0)	0 (0.009)	0 (0.001)	0.013 (0.008)	0 (0)	0 (0.005)	0 (0)	0 (0)	0.288 (0.023)	0.147 (0.042)	0.254 (0.034)	0.109 (0.027)
ID0034117	O‘AHU	0.015 (0.021)	0 (0.008)	0.013 (0.008)	0.039 (0.017)	0.015 (0.012)	0 (0)	0 (0.008)	0 (0.011)	0 (0.005)	0.006 (0.018)	0 (0.013)	0 (0.009)	0 (0.014)	0 (0)	0 (0.003)	0 (0.002)	0 (0.014)	0 (0.003)	0.017 (0.019)	0.371 (0.027)	0.194 (0.042)	0.243 (0.035)	0.086 (0.024)
ID0034118	O‘AHU	0.001 (0.017)	0.013 (0.018)	0.016 (0.006)	0 (0.009)	0.014 (0.016)	0.082 (0.031)	0 (0)	0 (0.018)	0 (0.003)	0 (0)	0 (0.013)	0.057 (0.032)	0 (0.005)	0 (0.007)	0.005 (0.007)	0 (0.003)	0.011 (0.02)	0.006 (0.014)	0 (0.019)	0.279 (0.032)	0.123 (0.064)	0.272 (0.048)	0.121 (0.027)
ID0034119	O‘AHU	0.029 (0.023)	0.022 (0.017)	0.018 (0.007)	0.012 (0.015)	0.019 (0.013)	0.034 (0.022)	0 (0.002)	0 (0.009)	0 (0.008)	0 (0.011)	0.013 (0.017)	0 (0.007)	0 (0.002)	0 (0)	0 (0.004)	0 (0.002)	0.032 (0.023)	0 (0)	0 (0.007)	0.28 (0.029)	0.184 (0.049)	0.22 (0.037)	0.137 (0.021)
ID0034120	O‘AHU	0.026 (0.021)	0.071 (0.018)	0.009 (0.007)	0.028 (0.02)	0 (0.003)	0.072 (0.029)	0.022 (0.018)	0 (0.004)	0 (0.004)	0 (0.001)	0.025 (0.018)	0.04 (0.027)	0 (0.009)	0 (0.008)	0.002 (0.006)	0 (0.001)	0.051 (0.028)	0 (0.001)	0 (0.002)	0.246 (0.032)	0 (0.03)	0.276 (0.034)	0.13 (0.018)
ID0034121	O‘AHU	0.033 (0.028)	0.016 (0.017)	0.002 (0.005)	0.019 (0.017)	0.023 (0.014)	0.039 (0.028)	0 (0)	0 (0)	0 (0)	0 (0.016)	0.002 (0.016)	0 (0.012)	0 (0)	0 (0)	0 (0.002)	0 (0.003)	0.066 (0.022)	0 (0.009)	0 (0.002)	0.251 (0.031)	0.194 (0.054)	0.258 (0.046)	0.097 (0.022)
ID0034130	O‘AHU	0.019 (0.022)	0.024 (0.02)	0.022 (0.007)	0 (0.002)	0.026 (0.018)	0.014 (0.017)	0 (0.003)	0 (0.005)	0 (0.001)	0 (0.011)	0 (0.012)	0 (0.009)	0 (0.001)	0 (0)	0.006 (0.006)	0 (0.007)	0.067 (0.034)	0.009 (0.014)	0 (0.013)	0.261 (0.035)	0.24 (0.046)	0.21 (0.038)	0.101 (0.027)
ID0034132	O‘AHU	0.004 (0.014)	0.027 (0.018)	0.016 (0.007)	0.022 (0.017)	0.016 (0.014)	0.007 (0.016)	0 (0.011)	0 (0)	0 (0.002)	0.032 (0.024)	0.062 (0.018)	0.013 (0.018)	0 (0.013)	0 (0.002)	0 (0.005)	0.003 (0.007)	0.037 (0.021)	0 (0.003)	0 (0.011)	0.31 (0.031)	0.122 (0.047)	0.217 (0.042)	0.113 (0.021)
ID0034133	O‘AHU	0.047 (0.024)	0.028 (0.018)	0.004 (0.006)	0.02 (0.015)	0.009 (0.013)	0 (0.016)	0.015 (0.019)	0 (0.003)	0 (0)	0.037 (0.028)	0.036 (0.019)	0 (0.011)	0 (0.005)	0 (0.004)	0.009 (0.008)	0 (0.005)	0.044 (0.025)	0 (0)	0 (0.013)	0.33 (0.031)	0.03 (0.046)	0.266 (0.041)	0.123 (0.024)
ID0034134	HAWAI‘I	0.134 (0.025)	0.006 (0.016)	0.007 (0.006)	0.007 (0.011)	0 (0.003)	0.058 (0.032)	0 (0.004)	0 (0.004)	0 (0)	0 (0.001)	0 (0.009)	0 (0.014)	0 (0)	0 (0.004)	0.01 (0.007)	0 (0.004)	0.032 (0.022)	0.01 (0.012)	0.003 (0.017)	0.195 (0.024)	0.135 (0.06)	0.293 (0.039)	0.112 (0.023)
ID0034135	HAWAI‘I	0.131 (0.027)	0.018 (0.021)	0.003 (0.004)	0.001 (0.009)	0.02 (0.013)	0.113 (0.034)	0 (0.001)	0 (0.011)	0 (0.001)	0 (0)	0 (0.003)	0.025 (0.027)	0 (0.015)	0.025 (0.021)	0.01 (0.007)	0 (0.004)	0.019 (0.016)	0 (0.006)	0 (0.014)	0.205 (0.026)	0.05 (0.052)	0.292 (0.041)	0.087 (0.023)
ID0034136	HAWAI‘I	0.108 (0.027)	0 (0.012)	0.007 (0.006)	0.009 (0.011)	0.011 (0.012)	0.086 (0.034)	0 (0.008)	0 (0.009)	0 (0.002)	0 (0)	0 (0.011)	0.031 (0.024)	0 (0.001)	0 (0.008)	0.002 (0.004)	0 (0.003)	0 (0.013)	0 (0.003)	0.017 (0.019)	0.227 (0.025)	0.117 (0.059)	0.27 (0.039)	0.114 (0.022)
ID0034137	HAWAI‘I	0.079 (0.028)	0.054 (0.023)	0.004 (0.005)	0.057 (0.018)	0 (0.01)	0.084 (0.026)	0 (0.007)	0.006 (0.028)	0 (0)	0 (0.001)	0 (0.003)	0.009 (0.014)	0 (0.017)	0 (0.008)	0.014 (0.007)	0.004 (0.006)	0 (0.009)	0.008 (0.008)	0 (0.012)	0.137 (0.018)	0.184 (0.051)	0.281 (0.041)	0.078 (0.019)
ID0034138	HAWAI‘I	0.099 (0.034)	0.041 (0.022)	0 (0.004)	0.025 (0.016)	0 (0.004)	0.004 (0.02)	0 (0.012)	0.121 (0.063)	0 (0.015)	0 (0.002)	0 (0.007)	0 (0.007)	0 (0.019)	0 (0.011)	0.008 (0.006)	0 (0.004)	0.036 (0.021)	0 (0.008)	0 (0.002)	0.181 (0.021)	0.164 (0.063)	0.242 (0.047)	0.079 (0.022)
ID0034139	O‘AHU	0.029 (0.027)	0.044 (0.019)	0.014 (0.007)	0 (0.008)	0.008 (0.011)	0.019 (0.02)	0 (0.001)	0 (0)	0 (0.002)	0 (0.001)	0.015 (0.016)	0 (0.001)	0 (0)	0 (0)	0 (0.005)	0 (0.003)	0.013 (0.021)	0 (0)	0.047 (0.027)	0.284 (0.027)	0.143 (0.043)	0.254 (0.033)	0.13 (0.02)
ID0034140	O‘AHU	0.091 (0.03)	0.019 (0.02)	0.011 (0.006)	0.007 (0.014)	0 (0.006)	0.119 (0.031)	0.012 (0.014)	0 (0)	0 (0.003)	0 (0.004)	0.009 (0.017)	0 (0.01)	0 (0.008)	0 (0)	0.007 (0.008)	0 (0.003)	0.066 (0.026)	0 (0)	0 (0.005)	0.264 (0.032)	0.094 (0.056)	0.227 (0.043)	0.074 (0.021)
ID0034142	O‘AHU	0.025 (0.019)	0.024 (0.019)	0.019 (0.006)	0 (0.003)	0 (0.01)	0.006 (0.012)	0.021 (0.023)	0.024 (0.028)	0 (0)	0 (0.011)	0 (0.005)	0.026 (0.025)	0.055 (0.029)	0 (0.009)	0.013 (0.009)	0 (0.002)	0.046 (0.025)	0 (0.002)	0 (0.014)	0.275 (0.027)	0.004 (0.031)	0.308 (0.041)	0.153 (0.023)
ID0034143	O‘AHU	0.079 (0.027)	0 (0.011)	0.004 (0.005)	0.017 (0.014)	0 (0.004)	0.036 (0.021)	0 (0)	0 (0.011)	0 (0.004)	0 (0.001)	0.036 (0.023)	0 (0.013)	0 (0.004)	0 (0.007)	0.007 (0.008)	0 (0.001)	0.06 (0.028)	0.004 (0.012)	0 (0.015)	0.27 (0.031)	0.066 (0.043)	0.291 (0.04)	0.13 (0.024)
ID0034144	O‘AHU	0.007 (0.015)	0.005 (0.013)	0.024 (0.007)	0.012 (0.013)	0.017 (0.014)	0.008 (0.019)	0.017 (0.024)	0 (0.018)	0 (0.001)	0 (0)	0.027 (0.02)	0.019 (0.02)	0 (0.012)	0 (0)	0 (0.003)	0 (0.003)	0.01 (0.023)	0.021 (0.017)	0.02 (0.031)	0.338 (0.027)	0.126 (0.044)	0.276 (0.038)	0.073 (0.023)
ID0034145	O‘AHU	0.059 (0.026)	0.029 (0.02)	0.002 (0.005)	0.02 (0.014)	0.009 (0.015)	0.056 (0.027)	0.014 (0.018)	0 (0.022)	0 (0.009)	0 (0.003)	0.036 (0.023)	0.059 (0.034)	0 (0.007)	0 (0.001)	0 (0.005)	0 (0.005)	0.021 (0.023)	0.006 (0.017)	0 (0.017)	0.295 (0.03)	0.03 (0.035)	0.241 (0.04)	0.123 (0.03)

ID0034146	HAWAIʻI	0.127 (0.03)	0.039 (0.025)	0.011 (0.006)	0.005 (0.013)	0.001 (0.009)	0.009 (0.017)	0.038 (0.024)	0.032 (0.038)	0 (0.014)	0 (0.016)	0.045 (0.02)	0.046 (0.029)	0 (0.016)	0 (0)	0.007 (0.007)	0.004 (0.007)	0 (0.014)	0 (0.001)	0.018 (0.02)	0.105 (0.025)	0.125 (0.056)	0.314 (0.042)	0.075 (0.024)
ID0034147	HAWAIʻI	0.094 (0.031)	0.038 (0.021)	0.011 (0.006)	0 (0.006)	0.008 (0.012)	0.055 (0.026)	0 (0)	0 (0.029)	0 (0.001)	0 (0.005)	0.024 (0.018)	0.002 (0.018)	0 (0.015)	0.023 (0.021)	0 (0.005)	0 (0.004)	0 (0.002)	0 (0.001)	0.036 (0.022)	0.189 (0.022)	0.167 (0.06)	0.241 (0.04)	0.111 (0.022)
ID0034148	HAWAIʻI	0.086 (0.027)	0.001 (0.012)	0.017 (0.006)	0 (0.006)	0.003 (0.01)	0.042 (0.027)	0 (0.001)	0 (0.007)	0 (0.002)	0 (0)	0 (0.01)	0 (0.013)	0 (0.003)	0.022 (0.021)	0.016 (0.007)	0.005 (0.008)	0.001 (0.012)	0.009 (0.011)	0.014 (0.018)	0.214 (0.022)	0.12 (0.056)	0.325 (0.043)	0.125 (0.023)
ID0034149	HAWAIʻI	0.149 (0.031)	0.072 (0.022)	0 (0.003)	0.012 (0.012)	0 (0.009)	0.09 (0.028)	0.009 (0.018)	0.066 (0.047)	0 (0)	0 (0)	0 (0.008)	0 (0.002)	0 (0.006)	0 (0.005)	0 (0.004)	0 (0)	0.031 (0.024)	0.014 (0.014)	0 (0.013)	0.183 (0.024)	0 (0.027)	0.269 (0.036)	0.105 (0.023)
ID0034150	HAWAIʻI	0.085 (0.037)	0.059 (0.022)	0.005 (0.005)	0 (0.004)	0 (0.003)	0.079 (0.03)	0.006 (0.019)	0.076 (0.057)	0.004 (0.016)	0 (0)	0.02 (0.017)	0.041 (0.027)	0 (0.011)	0 (0.008)	0 (0.004)	0 (0.002)	0 (0.009)	0.006 (0.014)	0.06 (0.033)	0.146 (0.026)	0.082 (0.067)	0.231 (0.041)	0.1 (0.025)
ID0034151	HAWAIʻI	0.056 (0.031)	0.019 (0.018)	0.011 (0.007)	0.007 (0.011)	0 (0.01)	0.065 (0.027)	0 (0.006)	0.106 (0.059)	0 (0.008)	0 (0)	0.028 (0.018)	0.036 (0.023)	0 (0.008)	0 (0.009)	0.019 (0.008)	0.006 (0.007)	0 (0.003)	0 (0.009)	0 (0.006)	0.18 (0.026)	0.111 (0.063)	0.276 (0.045)	0.079 (0.024)
ID0034152	HAWAIʻI	0.074 (0.028)	0.036 (0.022)	0.01 (0.007)	0.025 (0.018)	0.002 (0.01)	0.074 (0.027)	0.035 (0.027)	0.017 (0.039)	0 (0.011)	0 (0.008)	0.008 (0.013)	0.015 (0.018)	0.006 (0.018)	0 (0.008)	0.017 (0.008)	0 (0.003)	0 (0.005)	0 (0.003)	0.009 (0.016)	0.16 (0.023)	0.13 (0.057)	0.296 (0.045)	0.086 (0.025)
ID0034153	HAWAIʻI	0.137 (0.03)	0.022 (0.018)	0.005 (0.005)	0 (0.009)	0.006 (0.011)	0.041 (0.025)	0 (0.007)	0 (0.042)	0 (0.004)	0 (0)	0 (0.007)	0.002 (0.014)	0 (0.008)	0.039 (0.022)	0.001 (0.005)	0 (0.004)	0.01 (0.018)	0.006 (0.01)	0.023 (0.019)	0.189 (0.025)	0.143 (0.058)	0.291 (0.037)	0.084 (0.02)
ID0034154	HAWAIʻI	0.159 (0.031)	0.025 (0.023)	0 (0.003)	0.004 (0.009)	0 (0.009)	0.034 (0.028)	0.012 (0.019)	0.052 (0.038)	0 (0.013)	0 (0.008)	0.013 (0.016)	0.047 (0.025)	0.013 (0.023)	0 (0.004)	0.01 (0.008)	0 (0.003)	0 (0.004)	0 (0)	0.001 (0.018)	0.139 (0.025)	0.135 (0.059)	0.247 (0.053)	0.108 (0.022)
ID0034155	HAWAIʻI	0.131 (0.031)	0.039 (0.024)	0.006 (0.006)	0.023 (0.017)	0 (0.005)	0.063 (0.027)	0 (0.013)	0.009 (0.026)	0 (0.01)	0 (0.013)	0.022 (0.017)	0.044 (0.028)	0 (0.017)	0 (0.006)	0.011 (0.008)	0 (0.006)	0 (0)	0 (0.005)	0 (0.012)	0.117 (0.028)	0.172 (0.059)	0.259 (0.041)	0.102 (0.023)
ID0034156	HAWAIʻI	0.105 (0.031)	0.047 (0.021)	0 (0.003)	0.014 (0.013)	0.037 (0.013)	0.044 (0.026)	0.003 (0.014)	0.025 (0.041)	0 (0.002)	0 (0.007)	0.02 (0.016)	0.039 (0.03)	0 (0.01)	0 (0.005)	0.011 (0.008)	0 (0.001)	0 (0.011)	0.004 (0.009)	0.031 (0.027)	0.163 (0.025)	0.087 (0.055)	0.279 (0.044)	0.09 (0.022)
ID0034160	OʻAHU	0.038 (0.025)	0.035 (0.017)	0.003 (0.005)	0 (0.007)	0 (0.006)	0.095 (0.034)	0.015 (0.019)	0 (0.007)	0 (0.003)	0 (0)	0.022 (0.019)	0.035 (0.025)	0.018 (0.023)	0 (0.001)	0.005 (0.007)	0 (0.001)	0.041 (0.026)	0 (0.003)	0 (0.004)	0.291 (0.035)	0 (0.018)	0.267 (0.037)	0.135 (0.023)
ID0034161	OʻAHU	0.016 (0.02)	0.022 (0.016)	0.008 (0.005)	0.002 (0.012)	0 (0.01)	0.082 (0.031)	0.007 (0.014)	0 (0.01)	0 (0)	0 (0)	0.032 (0.02)	0.039 (0.025)	0.002 (0.016)	0 (0.004)	0.005 (0.007)	0 (0.001)	0.049 (0.027)	0 (0.002)	0.003 (0.017)	0.298 (0.036)	0 (0.006)	0.315 (0.034)	0.12 (0.019)
ID0034162	OʻAHU	0.038 (0.026)	0.01 (0.015)	0.004 (0.006)	0.033 (0.022)	0 (0.008)	0.055 (0.026)	0 (0)	0 (0.01)	0 (0)	0 (0.008)	0.023 (0.02)	0.029 (0.026)	0.013 (0.019)	0 (0.001)	0 (0.005)	0 (0.001)	0.038 (0.026)	0 (0.009)	0.016 (0.025)	0.32 (0.033)	0.022 (0.034)	0.305 (0.042)	0.094 (0.027)
ID0034164	OʻAHU	0.024 (0.02)	0.044 (0.021)	0.018 (0.006)	0 (0.005)	0.007 (0.012)	0 (0.006)	0 (0.003)	0 (0.014)	0 (0)	0 (0.003)	0 (0.013)	0.048 (0.03)	0 (0.011)	0 (0.004)	0 (0.005)	0 (0.001)	0.013 (0.02)	0 (0.004)	0 (0.001)	0.335 (0.029)	0.083 (0.051)	0.279 (0.039)	0.15 (0.024)
ID0034165	OʻAHU	0 (0.011)	0.027 (0.017)	0.007 (0.006)	0.018 (0.016)	0.009 (0.012)	0.028 (0.02)	0 (0)	0 (0.01)	0 (0.001)	0 (0)	0.042 (0.023)	0.074 (0.03)	0 (0.009)	0 (0.006)	0.004 (0.006)	0 (0.001)	0.023 (0.026)	0 (0.005)	0.008 (0.025)	0.234 (0.034)	0.134 (0.048)	0.263 (0.037)	0.129 (0.028)
ID0034166	OʻAHU	0.02 (0.024)	0.016 (0.016)	0.003 (0.006)	0.032 (0.019)	0.009 (0.011)	0.063 (0.03)	0 (0.002)	0 (0.013)	0 (0)	0 (0)	0.041 (0.021)	0.046 (0.029)	0 (0.01)	0 (0.002)	0.005 (0.007)	0 (0.001)	0.032 (0.031)	0 (0.004)	0.007 (0.026)	0.246 (0.03)	0.108 (0.051)	0.241 (0.041)	0.13 (0.027)
ID0034167	OʻAHU	0.031 (0.027)	0.021 (0.018)	0.02 (0.008)	0.005 (0.011)	0 (0.004)	0.054 (0.031)	0 (0.006)	0 (0.001)	0 (0)	0 (0.004)	0 (0.011)	0 (0.008)	0 (0.01)	0 (0.001)	0.005 (0.006)	0 (0.004)	0.086 (0.029)	0 (0.01)	0 (0.02)	0.27 (0.028)	0.131 (0.061)	0.273 (0.045)	0.104 (0.021)
ID0034168	OʻAHU	0 (0.015)	0.025 (0.02)	0.008 (0.007)	0.04 (0.018)	0.009 (0.012)	0.01 (0.017)	0 (0.012)	0.05 (0.038)	0 (0)	0 (0.003)	0 (0.011)	0.04 (0.024)	0 (0.005)	0 (0.005)	0 (0.003)	0 (0.003)	0.017 (0.024)	0 (0.005)	0 (0.008)	0.337 (0.032)	0.114 (0.042)	0.237 (0.041)	0.113 (0.022)
ID0034169	OʻAHU	0.032 (0.025)	0.012 (0.018)	0.018 (0.007)	0.03 (0.021)	0.008 (0.013)	0.003 (0.017)	0 (0.001)	0 (0.015)	0 (0.011)	0 (0.008)	0 (0.011)	0 (0.009)	0 (0.007)	0 (0)	0.006 (0.007)	0.009 (0.01)	0.021 (0.023)	0.015 (0.013)	0 (0.021)	0.285 (0.03)	0.245 (0.051)	0.226 (0.038)	0.088 (0.025)

ID0034173	O'AHU	0 (0.013)	0.015 (0.018)	0.005 (0.006)	0.038 (0.019)	0 (0.007)	0.023 (0.026)	0 (0.002)	0 (0)	0 (0)	0 (0.004)	0.019 (0.02)	0 (0.011)	0 (0)	0 (0)	0 (0.003)	0 (0.003)	0.037 (0.029)	0 (0.004)	0.019 (0.027)	0.248 (0.03)	0.246 (0.058)	0.204 (0.043)	0.145 (0.022)
ID0034174	O'AHU	0.012 (0.021)	0 (0.014)	0.009 (0.007)	0.013 (0.015)	0.017 (0.014)	0.001 (0.013)	0 (0)	0 (0.006)	0 (0.005)	0 (0.002)	0.039 (0.023)	0.078 (0.037)	0 (0.008)	0 (0.003)	0 (0.004)	0 (0.003)	0.02 (0.026)	0 (0.012)	0 (0.001)	0.235 (0.037)	0.203 (0.058)	0.258 (0.042)	0.114 (0.028)
ID0034178	O'AHU	0 (0.013)	0.006 (0.015)	0.015 (0.007)	0.044 (0.021)	0.011 (0.013)	0.057 (0.029)	0 (0.009)	0 (0.025)	0 (0.007)	0 (0.003)	0 (0.013)	0 (0.012)	0 (0.006)	0 (0)	0.009 (0.007)	0.006 (0.008)	0 (0.014)	0 (0.002)	0 (0.001)	0.337 (0.026)	0.202 (0.056)	0.233 (0.041)	0.08 (0.025)
ID0034180	O'AHU	0.075 (0.026)	0.003 (0.01)	0.009 (0.006)	0.023 (0.015)	0.018 (0.015)	0.052 (0.027)	0 (0.003)	0 (0.007)	0 (0)	0 (0.008)	0.031 (0.019)	0.021 (0.021)	0 (0.015)	0.013 (0.018)	0.002 (0.006)	0 (0)	0.019 (0.021)	0 (0)	0 (0.009)	0.28 (0.026)	0.001 (0.026)	0.317 (0.041)	0.137 (0.023)
ID0034181	O'AHU	0.068 (0.026)	0 (0.009)	0 (0.003)	0.026 (0.017)	0.021 (0.014)	0.018 (0.021)	0.012 (0.022)	0.112 (0.055)	0 (0.003)	0 (0.011)	0.034 (0.025)	0.013 (0.024)	0.025 (0.024)	0 (0.006)	0 (0.005)	0 (0)	0.042 (0.026)	0 (0.008)	0 (0.02)	0.26 (0.035)	0 (0.036)	0.245 (0.039)	0.122 (0.024)
ID0034182	O'AHU	0.069 (0.027)	0 (0.011)	0.001 (0.005)	0.04 (0.018)	0 (0.007)	0.111 (0.027)	0 (0)	0 (0.006)	0.03 (0.023)	0.035 (0.027)	0 (0.006)	0.054 (0.03)	0 (0.013)	0 (0.003)	0 (0.005)	0 (0.003)	0.03 (0.026)	0 (0)	0.011 (0.019)	0.235 (0.032)	0.11 (0.054)	0.179 (0.045)	0.096 (0.022)
ID0034183	O'AHU	0.026 (0.02)	0.006 (0.015)	0.004 (0.005)	0.009 (0.013)	0.016 (0.014)	0.003 (0.018)	0.025 (0.02)	0.007 (0.036)	0 (0)	0 (0.003)	0.036 (0.02)	0.044 (0.03)	0 (0.001)	0 (0.005)	0 (0.004)	0.001 (0.009)	0 (0.018)	0 (0.009)	0.03 (0.025)	0.309 (0.026)	0.121 (0.055)	0.238 (0.038)	0.124 (0.026)
ID0034197	O'AHU	0.058 (0.025)	0 (0.006)	0.013 (0.007)	0.024 (0.015)	0.001 (0.011)	0.05 (0.029)	0.008 (0.016)	0 (0.018)	0 (0)	0 (0.005)	0.019 (0.017)	0.012 (0.019)	0 (0.02)	0 (0.006)	0 (0.001)	0 (0.001)	0.053 (0.025)	0 (0.002)	0 (0.005)	0.292 (0.029)	0.047 (0.045)	0.285 (0.049)	0.139 (0.021)
ID0037096	O'AHU	0.027 (0.022)	0.012 (0.016)	0.002 (0.006)	0.045 (0.019)	0 (0.007)	0.084 (0.029)	0 (0.006)	0.015 (0.034)	0 (0.009)	0 (0.013)	0 (0.007)	0.065 (0.027)	0 (0.007)	0 (0.002)	0.007 (0.007)	0 (0.001)	0.007 (0.022)	0 (0)	0.038 (0.026)	0.227 (0.026)	0.124 (0.045)	0.24 (0.039)	0.107 (0.021)
ID0037097	O'AHU	0.076 (0.024)	0.009 (0.016)	0.001 (0.005)	0.036 (0.017)	0 (0.009)	0.049 (0.028)	0 (0.007)	0 (0.032)	0 (0.002)	0 (0.013)	0 (0.008)	0.019 (0.025)	0.013 (0.023)	0.023 (0.019)	0.001 (0.006)	0 (0.004)	0.056 (0.029)	0 (0.011)	0 (0.006)	0.283 (0.029)	0.093 (0.049)	0.219 (0.041)	0.122 (0.019)
ID0037098	O'AHU	0.039 (0.028)	0.005 (0.015)	0.001 (0.005)	0.04 (0.021)	0.005 (0.011)	0.069 (0.027)	0 (0.003)	0 (0.012)	0 (0.004)	0 (0.014)	0 (0.009)	0.012 (0.025)	0.013 (0.022)	0 (0.005)	0.004 (0.006)	0 (0.002)	0.047 (0.025)	0 (0.011)	0 (0.017)	0.301 (0.029)	0.099 (0.052)	0.251 (0.045)	0.115 (0.024)
ID0037099	O'AHU	0.01 (0.019)	0.027 (0.02)	0.027 (0.007)	0 (0.005)	0.032 (0.015)	0.069 (0.028)	0 (0.004)	0 (0.015)	0 (0.001)	0 (0.002)	0 (0.012)	0.03 (0.027)	0 (0.007)	0 (0)	0 (0.002)	0 (0.004)	0.028 (0.029)	0 (0.011)	0.021 (0.026)	0.303 (0.029)	0.092 (0.05)	0.27 (0.039)	0.093 (0.025)
ID0037100	HAWAI'I	0.114 (0.03)	0.051 (0.021)	0.007 (0.005)	0.025 (0.013)	0.02 (0.014)	0.088 (0.026)	0.003 (0.015)	0 (0.024)	0 (0)	0 (0.009)	0.027 (0.017)	0.025 (0.023)	0 (0.017)	0 (0)	0.008 (0.008)	0 (0.001)	0.057 (0.025)	0.005 (0.011)	0 (0.007)	0.09 (0.024)	0.114 (0.05)	0.273 (0.042)	0.093 (0.029)
ID0037102	HAWAI'I	0.108 (0.032)	0.032 (0.02)	0.004 (0.005)	0 (0.005)	0 (0.007)	0.068 (0.028)	0.013 (0.019)	0.042 (0.05)	0 (0.001)	0 (0)	0.034 (0.019)	0.06 (0.026)	0 (0.019)	0 (0.011)	0.014 (0.008)	0 (0)	0 (0.008)	0 (0.003)	0 (0.014)	0.144 (0.029)	0.09 (0.058)	0.306 (0.051)	0.085 (0.023)
ID0037105	O'AHU	0 (0.007)	0.022 (0.019)	0.002 (0.006)	0.066 (0.017)	0.019 (0.014)	0.03 (0.024)	0.017 (0.017)	0 (0.001)	0 (0.006)	0 (0.018)	0 (0.012)	0 (0.004)	0.015 (0.023)	0 (0.005)	0 (0.004)	0.002 (0.007)	0.029 (0.022)	0 (0.005)	0 (0.011)	0.342 (0.028)	0.122 (0.052)	0.247 (0.035)	0.085 (0.023)
ID0037106	HAWAI'I	0.133 (0.026)	0.034 (0.019)	0.003 (0.005)	0.044 (0.019)	0 (0.004)	0.001 (0.015)	0 (0.011)	0 (0.004)	0 (0.006)	0.053 (0.031)	0.012 (0.016)	0 (0)	0 (0.005)	0 (0)	0.003 (0.005)	0 (0.002)	0 (0.017)	0 (0.003)	0.057 (0.025)	0.12 (0.021)	0.151 (0.049)	0.265 (0.041)	0.124 (0.019)
ID0037107	HAWAI'I	0.152 (0.03)	0.03 (0.021)	0.012 (0.006)	0 (0.006)	0.005 (0.01)	0.106 (0.027)	0.021 (0.02)	0 (0.02)	0 (0.002)	0 (0.004)	0 (0.007)	0.008 (0.019)	0 (0.007)	0 (0.007)	0.015 (0.008)	0 (0.004)	0 (0.008)	0 (0.002)	0.048 (0.029)	0.144 (0.027)	0.083 (0.059)	0.268 (0.044)	0.108 (0.024)
ID0037108	HAWAI'I	0.142 (0.029)	0.026 (0.022)	0.008 (0.006)	0.012 (0.014)	0 (0.003)	0.051 (0.027)	0 (0.007)	0.005 (0.025)	0.005 (0.018)	0 (0.003)	0.031 (0.019)	0.034 (0.023)	0 (0.009)	0 (0.001)	0.017 (0.008)	0 (0.003)	0 (0.012)	0.005 (0.009)	0 (0.014)	0.145 (0.023)	0.163 (0.064)	0.243 (0.042)	0.113 (0.024)
ID0037109	HAWAI'I	0.073 (0.029)	0.039 (0.021)	0 (0.001)	0.042 (0.017)	0 (0.007)	0.019 (0.02)	0 (0.015)	0.052 (0.042)	0 (0.013)	0 (0.01)	0 (0.005)	0 (0.011)	0 (0.004)	0 (0.014)	0 (0.004)	0 (0)	0.015 (0.018)	0 (0.002)	0.004 (0.016)	0.209 (0.028)	0.185 (0.058)	0.285 (0.035)	0.077 (0.022)
ID0037110	HAWAI'I	0.119 (0.029)	0.042 (0.019)	0.005 (0.005)	0.002 (0.01)	0 (0.005)	0.064 (0.029)	0 (0.002)	0 (0.031)	0 (0)	0 (0.006)	0.023 (0.017)	0.019 (0.021)	0 (0.015)	0 (0.003)	0.003 (0.006)	0 (0.002)	0.023 (0.022)	0 (0.008)	0.041 (0.026)	0.143 (0.025)	0.107 (0.051)	0.299 (0.043)	0.108 (0.022)

ID0037113	O‘AHU	0 (0.01)	0.005 (0.012)	0.007 (0.006)	0.043 (0.016)	0.003 (0.011)	0.064 (0.027)	0 (0.005)	0 (0.002)	0 (0)	0 (0.008)	0.015 (0.017)	0 (0.014)	0 (0.017)	0 (0.002)	0 (0.006)	0 (0.006)	0.047 (0.03)	0 (0.002)	0.034 (0.024)	0.306 (0.028)	0.099 (0.048)	0.278 (0.038)	0.099 (0.019)
ID0037115	MOLOKA‘I	0 (0.017)	0.05 (0.022)	0.008 (0.007)	0.024 (0.016)	0.024 (0.016)	0.07 (0.036)	0.024 (0.024)	0.066 (0.059)	0 (0.011)	0 (0)	0.007 (0.013)	0.039 (0.028)	0 (0.001)	0 (0.008)	0 (0)	0 (0.002)	0.025 (0.024)	0 (0.002)	0.014 (0.021)	0.14 (0.026)	0.039 (0.047)	0.268 (0.052)	0.204 (0.029)
ID0037116	MOLOKA‘I	0.067 (0.028)	0.009 (0.015)	0.004 (0.005)	0.017 (0.016)	0.029 (0.018)	0.023 (0.023)	0 (0.013)	0.017 (0.041)	0 (0.002)	0 (0)	0.01 (0.016)	0.033 (0.025)	0 (0.016)	0 (0.006)	0 (0.001)	0 (0.004)	0.028 (0.021)	0 (0.006)	0 (0.009)	0.137 (0.025)	0.132 (0.052)	0.376 (0.042)	0.118 (0.031)
ID0037117	MOLOKA‘I	0.061 (0.028)	0.04 (0.022)	0.004 (0.005)	0.027 (0.019)	0.014 (0.014)	0.019 (0.021)	0.01 (0.019)	0 (0.031)	0 (0)	0 (0)	0.006 (0.013)	0.028 (0.025)	0.014 (0.025)	0.02 (0.021)	0 (0)	0 (0.002)	0.024 (0.021)	0 (0.002)	0 (0.012)	0.144 (0.026)	0.07 (0.051)	0.388 (0.043)	0.133 (0.029)
ID0037118	MOLOKA‘I	0.044 (0.028)	0.022 (0.019)	0.004 (0.005)	0.018 (0.017)	0.019 (0.015)	0.055 (0.03)	0 (0.005)	0 (0.004)	0 (0.013)	0 (0.001)	0 (0.002)	0.009 (0.024)	0 (0.009)	0 (0.007)	0 (0)	0.008 (0.01)	0.031 (0.02)	0 (0.001)	0 (0.015)	0.199 (0.027)	0.139 (0.055)	0.307 (0.046)	0.145 (0.031)
ID0037119	MOLOKA‘I	0.057 (0.031)	0.029 (0.023)	0.011 (0.007)	0.002 (0.013)	0.032 (0.017)	0.021 (0.023)	0 (0.003)	0 (0.02)	0 (0.009)	0 (0.002)	0.004 (0.012)	0.028 (0.026)	0 (0.004)	0.004 (0.015)	0 (0.002)	0 (0.003)	0.026 (0.027)	0 (0)	0.044 (0.033)	0.155 (0.029)	0.121 (0.057)	0.332 (0.044)	0.134 (0.03)
ID0037120	MOLOKA‘I	0.049 (0.028)	0.047 (0.023)	0.01 (0.007)	0.027 (0.019)	0.035 (0.019)	0.041 (0.029)	0.013 (0.018)	0 (0.013)	0 (0.005)	0 (0.003)	0 (0.011)	0.022 (0.025)	0 (0.009)	0 (0.011)	0 (0.003)	0 (0.003)	0.059 (0.028)	0 (0.004)	0 (0.015)	0.135 (0.028)	0.059 (0.046)	0.378 (0.044)	0.127 (0.028)
ID0037121	MOLOKA‘I	0.032 (0.026)	0.04 (0.02)	0.009 (0.006)	0.021 (0.017)	0.015 (0.014)	0.032 (0.028)	0 (0.007)	0 (0.018)	0 (0.003)	0 (0)	0.006 (0.016)	0.004 (0.019)	0 (0)	0.014 (0.018)	0 (0)	0 (0.003)	0.05 (0.027)	0 (0.002)	0.027 (0.026)	0.171 (0.026)	0.084 (0.051)	0.366 (0.042)	0.131 (0.026)
ID0037122	MOLOKA‘I	0.105 (0.028)	0.031 (0.02)	0.009 (0.006)	0.017 (0.012)	0.025 (0.016)	0.069 (0.027)	0 (0.002)	0 (0.021)	0 (0.006)	0 (0)	0.001 (0.011)	0.004 (0.02)	0 (0.006)	0 (0.004)	0 (0.001)	0 (0.005)	0.042 (0.022)	0 (0.002)	0 (0.013)	0.162 (0.023)	0.08 (0.049)	0.29 (0.045)	0.164 (0.03)
ID0037123	MOLOKA‘I	0.046 (0.026)	0.065 (0.023)	0.009 (0.007)	0.019 (0.016)	0.025 (0.018)	0.001 (0.015)	0.005 (0.014)	0.02 (0.038)	0 (0.002)	0 (0.009)	0.047 (0.023)	0.024 (0.026)	0 (0.003)	0 (0.009)	0 (0)	0 (0.003)	0.067 (0.029)	0 (0.006)	0 (0.004)	0.111 (0.034)	0.115 (0.06)	0.292 (0.045)	0.152 (0.03)
ID0037124	MOLOKA‘I	0.057 (0.024)	0 (0.014)	0.012 (0.007)	0.03 (0.02)	0.027 (0.017)	0.047 (0.03)	0.029 (0.024)	0 (0.023)	0 (0.006)	0 (0)	0.008 (0.016)	0 (0.017)	0 (0.001)	0.021 (0.022)	0 (0)	0 (0.005)	0.021 (0.023)	0 (0.007)	0.023 (0.022)	0.191 (0.03)	0.067 (0.053)	0.356 (0.048)	0.11 (0.026)
ID0037126	HAWAI‘I	0.1 (0.028)	0.054 (0.018)	0.007 (0.007)	0.043 (0.018)	0.012 (0.014)	0.02 (0.021)	0 (0.014)	0 (0.019)	0 (0)	0 (0)	0.036 (0.019)	0.026 (0.024)	0 (0.018)	0 (0.003)	0.014 (0.007)	0 (0.005)	0.024 (0.024)	0.013 (0.014)	0 (0.015)	0.12 (0.021)	0.125 (0.059)	0.296 (0.043)	0.111 (0.02)
ID0037127	HAWAI‘I	0.066 (0.032)	0.029 (0.021)	0 (0.003)	0.027 (0.015)	0.03 (0.016)	0.014 (0.021)	0 (0.003)	0 (0.018)	0 (0)	0 (0.002)	0.03 (0.02)	0.021 (0.02)	0 (0.011)	0 (0.007)	0 (0.003)	0.006 (0.007)	0.001 (0.014)	0.014 (0.012)	0 (0.005)	0.197 (0.025)	0.155 (0.052)	0.298 (0.04)	0.112 (0.026)
ID0037128	O‘AHU	0.012 (0.023)	0.042 (0.019)	0.018 (0.007)	0 (0.008)	0 (0.006)	0.03 (0.025)	0 (0)	0 (0.002)	0 (0.006)	0 (0)	0 (0.013)	0 (0.009)	0 (0.004)	0 (0)	0.001 (0.006)	0 (0.006)	0.022 (0.018)	0 (0.001)	0 (0.013)	0.361 (0.029)	0.09 (0.049)	0.269 (0.04)	0.155 (0.024)
ID0037129	O‘AHU	0.04 (0.027)	0.047 (0.02)	0.023 (0.007)	0 (0.01)	0.006 (0.013)	0.081 (0.027)	0 (0.002)	0 (0)	0 (0.001)	0 (0)	0.028 (0.022)	0.019 (0.02)	0 (0)	0 (0)	0.003 (0.006)	0 (0.004)	0.016 (0.019)	0 (0.005)	0 (0.006)	0.313 (0.03)	0.054 (0.052)	0.252 (0.044)	0.118 (0.024)
ID0037130	O‘AHU	0.031 (0.028)	0.052 (0.02)	0.013 (0.007)	0.009 (0.013)	0.019 (0.013)	0.052 (0.03)	0 (0)	0 (0)	0 (0.01)	0 (0)	0.014 (0.014)	0.042 (0.03)	0 (0.009)	0 (0)	0.006 (0.007)	0 (0.005)	0.046 (0.026)	0 (0.006)	0 (0.013)	0.242 (0.027)	0.078 (0.053)	0.264 (0.044)	0.133 (0.026)
ID0037133	O‘AHU	0.031 (0.029)	0.055 (0.018)	0.013 (0.006)	0.009 (0.014)	0.002 (0.008)	0.051 (0.03)	0 (0)	0 (0)	0 (0.004)	0 (0)	0 (0.01)	0.065 (0.031)	0 (0.001)	0 (0)	0.008 (0.007)	0.001 (0.008)	0.013 (0.022)	0 (0.003)	0 (0.009)	0.265 (0.028)	0.112 (0.053)	0.267 (0.044)	0.108 (0.027)
ID0037134	O‘AHU	0 (0.015)	0.06 (0.019)	0.013 (0.006)	0.013 (0.014)	0.015 (0.013)	0.077 (0.029)	0 (0.008)	0 (0.019)	0 (0)	0 (0)	0.028 (0.017)	0.024 (0.021)	0 (0.004)	0 (0.002)	0 (0.003)	0.006 (0.01)	0.022 (0.023)	0 (0.002)	0.016 (0.024)	0.296 (0.031)	0.029 (0.041)	0.309 (0.037)	0.092 (0.025)
ID0037150	O‘AHU	0.032 (0.027)	0.035 (0.018)	0 (0.004)	0.034 (0.017)	0 (0.006)	0.084 (0.03)	0.011 (0.017)	0 (0.008)	0 (0.009)	0 (0.004)	0 (0.009)	0 (0.016)	0 (0.01)	0 (0.012)	0.006 (0.006)	0 (0.004)	0.086 (0.029)	0 (0.004)	0.003 (0.021)	0.25 (0.025)	0.128 (0.046)	0.252 (0.038)	0.08 (0.019)
ID0037155	HAWAI‘I	0.134 (0.028)	0.043 (0.02)	0 (0.002)	0.061 (0.014)	0 (0.003)	0.071 (0.03)	0 (0.008)	0 (0.015)	0 (0.001)	0 (0.002)	0.021 (0.018)	0 (0.006)	0 (0.01)	0 (0.009)	0.005 (0.006)	0 (0.005)	0.001 (0.013)	0 (0.002)	0.017 (0.019)	0.198 (0.024)	0.131 (0.048)	0.247 (0.041)	0.07 (0.022)

ID0037156	HAWAIʻI	0.061 (0.031)	0.056 (0.023)	0.005 (0.005)	0.01 (0.012)	0.012 (0.014)	0.043 (0.026)	0 (0.006)	0 (0.021)	0 (0)	0 (0.003)	0.019 (0.017)	0.006 (0.015)	0 (0)	0 (0)	0.001 (0.005)	0.001 (0.007)	0.01 (0.019)	0.025 (0.015)	0 (0.017)	0.185 (0.024)	0.191 (0.056)	0.255 (0.05)	0.119 (0.027)
ID0037157	HAWAIʻI	0.066 (0.024)	0.048 (0.025)	0.003 (0.005)	0.047 (0.017)	0.004 (0.01)	0.073 (0.029)	0 (0.009)	0 (0.011)	0 (0.006)	0 (0.013)	0.004 (0.01)	0 (0.015)	0 (0.01)	0.009 (0.017)	0 (0.003)	0 (0.002)	0 (0.015)	0 (0.004)	0.03 (0.023)	0.156 (0.026)	0.212 (0.045)	0.249 (0.04)	0.099 (0.022)
ID0037158	OʻAHU	0.016 (0.017)	0.044 (0.019)	0.012 (0.007)	0.023 (0.017)	0.008 (0.01)	0.038 (0.026)	0 (0.001)	0 (0.001)	0 (0.002)	0 (0.001)	0 (0.012)	0.056 (0.026)	0 (0.01)	0 (0.003)	0.006 (0.007)	0 (0.004)	0.02 (0.02)	0 (0.007)	0 (0.013)	0.305 (0.028)	0.104 (0.05)	0.237 (0.039)	0.131 (0.02)
ID0037159	OʻAHU	0 (0.01)	0.014 (0.018)	0.015 (0.008)	0.033 (0.019)	0 (0.008)	0.022 (0.022)	0 (0.002)	0 (0.016)	0 (0.001)	0 (0.005)	0.025 (0.02)	0 (0.015)	0 (0.001)	0 (0)	0.003 (0.007)	0 (0)	0.041 (0.024)	0 (0.012)	0 (0.002)	0.31 (0.033)	0.158 (0.046)	0.247 (0.036)	0.133 (0.023)
ID0037160	OʻAHU	0.051 (0.026)	0.009 (0.017)	0.013 (0.006)	0 (0.004)	0 (0.009)	0.064 (0.026)	0 (0.008)	0 (0.021)	0 (0)	0 (0.008)	0 (0.007)	0 (0.017)	0 (0.012)	0 (0.006)	0.006 (0.007)	0.002 (0.007)	0.069 (0.028)	0 (0.008)	0 (0.008)	0.305 (0.03)	0.097 (0.046)	0.28 (0.039)	0.106 (0.023)
ID0037161	OʻAHU	0.02 (0.026)	0.015 (0.018)	0.013 (0.007)	0.013 (0.013)	0 (0.004)	0.051 (0.03)	0 (0.004)	0 (0.018)	0 (0)	0 (0.01)	0.031 (0.02)	0.021 (0.024)	0 (0.007)	0 (0)	0.012 (0.009)	0 (0)	0.048 (0.028)	0 (0.002)	0 (0.005)	0.311 (0.03)	0.061 (0.047)	0.297 (0.044)	0.106 (0.023)
ID0037162	OʻAHU	0.06 (0.026)	0.018 (0.018)	0.003 (0.005)	0.024 (0.014)	0.004 (0.012)	0.071 (0.028)	0 (0.005)	0.017 (0.029)	0 (0.005)	0 (0.004)	0.027 (0.02)	0.009 (0.021)	0.009 (0.021)	0 (0.009)	0.007 (0.007)	0 (0.002)	0.018 (0.023)	0 (0.005)	0.022 (0.025)	0.279 (0.032)	0.028 (0.035)	0.27 (0.043)	0.135 (0.025)
ID0037163	OʻAHU	0 (0.015)	0.045 (0.022)	0.007 (0.007)	0.041 (0.016)	0 (0.008)	0.056 (0.029)	0 (0.007)	0.016 (0.023)	0 (0.002)	0 (0.012)	0.015 (0.016)	0 (0.003)	0.025 (0.026)	0 (0.011)	0.009 (0.008)	0 (0.002)	0 (0.019)	0 (0.001)	0.023 (0.022)	0.346 (0.027)	0.009 (0.03)	0.292 (0.038)	0.118 (0.024)
ID0037164	OʻAHU	0.057 (0.027)	0.009 (0.013)	0.014 (0.006)	0 (0.006)	0.002 (0.01)	0.037 (0.025)	0 (0.011)	0 (0)	0 (0.004)	0 (0)	0.01 (0.015)	0 (0.016)	0.002 (0.018)	0 (0)	0.003 (0.006)	0.002 (0.007)	0.056 (0.023)	0 (0.003)	0 (0.008)	0.276 (0.029)	0.083 (0.051)	0.329 (0.04)	0.12 (0.021)
ID0037165	OʻAHU	0.043 (0.026)	0.025 (0.017)	0.022 (0.006)	0 (0.006)	0.035 (0.016)	0.007 (0.016)	0 (0.008)	0 (0.009)	0 (0.002)	0 (0.006)	0.009 (0.014)	0 (0.018)	0.015 (0.024)	0 (0)	0 (0.002)	0 (0.003)	0.02 (0.018)	0 (0.002)	0.011 (0.019)	0.384 (0.031)	0.031 (0.04)	0.268 (0.039)	0.13 (0.024)
ID0037166	OʻAHU	0.008 (0.018)	0.004 (0.013)	0.013 (0.006)	0.005 (0.011)	0.02 (0.015)	0.045 (0.026)	0 (0)	0 (0.022)	0 (0.001)	0 (0.006)	0.044 (0.021)	0 (0.01)	0 (0.012)	0 (0.003)	0.002 (0.007)	0 (0.001)	0.005 (0.014)	0 (0)	0.024 (0.023)	0.314 (0.024)	0.153 (0.055)	0.245 (0.04)	0.118 (0.023)
ID0037167	OʻAHU	0.014 (0.018)	0.032 (0.019)	0.018 (0.006)	0.012 (0.012)	0.008 (0.013)	0 (0.01)	0 (0.004)	0.037 (0.031)	0 (0.001)	0 (0.006)	0 (0.013)	0 (0.015)	0 (0.009)	0 (0)	0.004 (0.006)	0.003 (0.007)	0.085 (0.027)	0 (0.006)	0 (0.005)	0.301 (0.025)	0.06 (0.042)	0.341 (0.033)	0.084 (0.021)
ID0037168	OʻAHU	0.046 (0.022)	0.025 (0.018)	0.021 (0.006)	0.016 (0.015)	0.021 (0.016)	0.014 (0.019)	0 (0.015)	0.012 (0.029)	0 (0)	0 (0)	0.039 (0.02)	0.024 (0.024)	0 (0.01)	0.026 (0.019)	0.005 (0.008)	0 (0.004)	0 (0.016)	0 (0.011)	0.045 (0.029)	0.328 (0.03)	0 (0.033)	0.26 (0.032)	0.12 (0.024)
ID0037169	OʻAHU	0.03 (0.025)	0.041 (0.02)	0.022 (0.007)	0.02 (0.015)	0 (0.007)	0 (0.01)	0.002 (0.017)	0 (0.014)	0 (0.001)	0 (0.002)	0.026 (0.019)	0 (0.003)	0 (0.006)	0 (0.001)	0.005 (0.007)	0 (0)	0.033 (0.024)	0 (0.003)	0 (0.009)	0.269 (0.031)	0.157 (0.053)	0.281 (0.038)	0.114 (0.023)
ID0037173	OʻAHU	0.019 (0.02)	0.013 (0.016)	0.012 (0.006)	0.005 (0.01)	0 (0.006)	0.025 (0.02)	0 (0.005)	0 (0.003)	0 (0.001)	0 (0.006)	0.006 (0.015)	0.009 (0.022)	0 (0.003)	0 (0.002)	0 (0.003)	0 (0.001)	0.073 (0.026)	0 (0.002)	0 (0.015)	0.289 (0.028)	0.172 (0.047)	0.239 (0.043)	0.139 (0.019)
ID0038031	OʻAHU	0.033 (0.022)	0.046 (0.022)	0.017 (0.005)	0 (0.003)	0.018 (0.012)	0 (0.009)	0 (0.003)	0 (0)	0 (0.001)	0 (0.003)	0 (0.004)	0.015 (0.02)	0 (0)	0 (0.001)	0 (0.003)	0.013 (0.011)	0.02 (0.018)	0 (0.007)	0.006 (0.019)	0.379 (0.025)	0.08 (0.043)	0.234 (0.034)	0.139 (0.023)
ID0038034	OʻAHU	0.042 (0.023)	0.05 (0.019)	0.016 (0.006)	0 (0.003)	0.003 (0.01)	0.068 (0.027)	0 (0.011)	0 (0.002)	0 (0.005)	0 (0.011)	0.003 (0.01)	0 (0.018)	0 (0.01)	0 (0.001)	0.008 (0.007)	0 (0.004)	0.015 (0.015)	0 (0.001)	0 (0.007)	0.356 (0.025)	0.021 (0.035)	0.272 (0.037)	0.145 (0.021)
ID0038045	OʻAHU	0.046 (0.024)	0.008 (0.013)	0.018 (0.007)	0.023 (0.015)	0.02 (0.015)	0.046 (0.026)	0 (0.015)	0 (0)	0 (0)	0 (0.003)	0 (0.008)	0.04 (0.025)	0 (0.005)	0 (0.017)	0 (0.005)	0 (0.003)	0.003 (0.017)	0 (0.003)	0 (0.012)	0.336 (0.033)	0.043 (0.041)	0.293 (0.037)	0.123 (0.027)
ID0038060	HAWAIʻI	0.1 (0.024)	0.038 (0.019)	0.008 (0.006)	0 (0.002)	0 (0.007)	0.072 (0.027)	0 (0.003)	0 (0.011)	0 (0.007)	0 (0.001)	0 (0.002)	0.031 (0.018)	0 (0.005)	0 (0.008)	0 (0.003)	0.003 (0.006)	0 (0.006)	0 (0.002)	0 (0.013)	0.205 (0.021)	0.168 (0.059)	0.292 (0.044)	0.084 (0.021)
ID0038061	HAWAIʻI	0.064 (0.03)	0.038 (0.02)	0 (0.004)	0.051 (0.019)	0.028 (0.018)	0.067 (0.029)	0.016 (0.021)	0 (0.018)	0 (0.001)	0 (0.015)	0.004 (0.011)	0 (0.004)	0 (0.008)	0 (0.01)	0.006 (0.006)	0.011 (0.008)	0.003 (0.018)	0 (0.006)	0.006 (0.016)	0.153 (0.027)	0.254 (0.056)	0.237 (0.042)	0.062 (0.022)

ID0038070	HAWAIʻI	0.074 (0.025)	0.026 (0.022)	0.011 (0.006)	0.047 (0.017)	0 (0.002)	0.075 (0.034)	0.011 (0.016)	0.028 (0.042)	0 (0.001)	0 (0.009)	0 (0.006)	0.001 (0.012)	0 (0.003)	0 (0.009)	0.009 (0.007)	0.002 (0.005)	0.009 (0.013)	0 (0.005)	0.006 (0.015)	0.163 (0.022)	0.202 (0.058)	0.259 (0.04)	0.078 (0.019)
ID0038071	HAWAIʻI	0.109 (0.028)	0.035 (0.023)	0.002 (0.005)	0.034 (0.017)	0 (0.006)	0.036 (0.03)	0 (0.011)	0.036 (0.042)	0 (0.003)	0.004 (0.02)	0.01 (0.012)	0 (0.002)	0 (0.002)	0.012 (0.017)	0.003 (0.005)	0.007 (0.008)	0 (0.002)	0 (0.006)	0 (0.005)	0.187 (0.021)	0.231 (0.065)	0.203 (0.037)	0.09 (0.021)
ID0038080	OʻAHU	0.042 (0.025)	0.004 (0.012)	0.018 (0.007)	0.021 (0.016)	0.029 (0.017)	0.029 (0.023)	0 (0)	0 (0.025)	0 (0.001)	0.035 (0.032)	0.048 (0.022)	0 (0.005)	0 (0.002)	0 (0.011)	0 (0.001)	0 (0.002)	0.037 (0.026)	0 (0.003)	0 (0.018)	0.33 (0.029)	0.079 (0.05)	0.244 (0.039)	0.084 (0.024)
ID0038493	HAWAIʻI	0.117 (0.029)	0.026 (0.019)	0.005 (0.005)	0.015 (0.014)	0 (0.008)	0.065 (0.03)	0 (0.004)	0.047 (0.036)	0 (0.004)	0 (0)	0 (0.009)	0.028 (0.025)	0 (0.015)	0 (0.01)	0.013 (0.009)	0 (0.001)	0 (0)	0 (0.004)	0.027 (0.026)	0.151 (0.026)	0.064 (0.051)	0.335 (0.05)	0.108 (0.021)
ID0038498	OʻAHU	0.013 (0.014)	0 (0.007)	0.007 (0.007)	0.048 (0.016)	0.014 (0.013)	0 (0.007)	0 (0.01)	0 (0.014)	0 (0.009)	0 (0.008)	0.032 (0.019)	0.012 (0.018)	0 (0.014)	0 (0.001)	0.002 (0.005)	0 (0.001)	0.012 (0.02)	0 (0)	0.029 (0.023)	0.352 (0.027)	0.13 (0.042)	0.264 (0.034)	0.086 (0.024)
ID0038499	OʻAHU	0.047 (0.025)	0.011 (0.015)	0.01 (0.007)	0.027 (0.018)	0.017 (0.015)	0.021 (0.023)	0.015 (0.017)	0 (0.013)	0 (0)	0 (0.001)	0.027 (0.019)	0.002 (0.016)	0 (0.006)	0.01 (0.015)	0 (0.004)	0 (0)	0.035 (0.027)	0 (0.003)	0.016 (0.026)	0.281 (0.032)	0.111 (0.054)	0.259 (0.036)	0.11 (0.025)
ID0038564	OʻAHU	0.004 (0.02)	0.036 (0.022)	0.007 (0.005)	0.001 (0.012)	0.024 (0.015)	0.057 (0.028)	0 (0.007)	0 (0)	0 (0.003)	0 (0)	0.014 (0.015)	0.045 (0.033)	0 (0.01)	0 (0)	0 (0.002)	0 (0)	0.066 (0.035)	0 (0.007)	0.007 (0.023)	0.237 (0.031)	0.105 (0.05)	0.266 (0.044)	0.132 (0.026)
ID0038590	OʻAHU	0 (0.008)	0.037 (0.019)	0.017 (0.007)	0.013 (0.013)	0.032 (0.017)	0.064 (0.028)	0.03 (0.02)	0 (0.018)	0 (0.006)	0 (0.009)	0.003 (0.014)	0 (0.014)	0.004 (0.023)	0 (0.005)	0.014 (0.008)	0 (0.002)	0 (0.008)	0 (0.005)	0.012 (0.018)	0.356 (0.026)	0.055 (0.04)	0.274 (0.034)	0.088 (0.026)
ID0041704	MAUI	0.035 (0.027)	0 (0.005)	0.002 (0.004)	0.042 (0.017)	0.019 (0.015)	0.093 (0.036)	0 (0)	0 (0.022)	0.02 (0.022)	0 (0.001)	0 (0.006)	0 (0.014)	0.01 (0.028)	0 (0.001)	0.004 (0.007)	0 (0.002)	0 (0.012)	0 (0.001)	0.028 (0.02)	0.19 (0.029)	0.217 (0.062)	0.228 (0.044)	0.111 (0.021)
ID0041705	OʻAHU	0.014 (0.019)	0.048 (0.019)	0.009 (0.006)	0.004 (0.013)	0.025 (0.013)	0.069 (0.031)	0.031 (0.024)	0 (0.037)	0 (0.008)	0.005 (0.017)	0.014 (0.014)	0 (0.016)	0 (0.013)	0.039 (0.028)	0.002 (0.005)	0.01 (0.008)	0.026 (0.022)	0.006 (0.01)	0.03 (0.028)	0.236 (0.026)	0.147 (0.061)	0.213 (0.036)	0.072 (0.022)
ID0041706	OʻAHU	0.074 (0.026)	0.013 (0.015)	0.004 (0.005)	0.037 (0.019)	0.001 (0.009)	0.102 (0.028)	0 (0.007)	0 (0.001)	0 (0.002)	0 (0.014)	0.02 (0.018)	0 (0.01)	0 (0.002)	0 (0.001)	0.001 (0.006)	0 (0.005)	0.056 (0.03)	0 (0.008)	0 (0.012)	0.267 (0.031)	0.088 (0.048)	0.257 (0.041)	0.082 (0.025)
ID0041709	MAUI	0.102 (0.028)	0.004 (0.012)	0.007 (0.006)	0.034 (0.019)	0.002 (0.01)	0.046 (0.027)	0 (0.004)	0 (0.012)	0.039 (0.023)	0 (0.006)	0 (0.011)	0 (0.01)	0 (0.014)	0 (0.001)	0.01 (0.008)	0 (0.004)	0.037 (0.026)	0 (0.006)	0.016 (0.021)	0.138 (0.026)	0.18 (0.059)	0.266 (0.037)	0.121 (0.023)
ID0041711	OʻAHU	0.037 (0.024)	0 (0.012)	0.009 (0.007)	0.021 (0.017)	0.005 (0.008)	0.087 (0.03)	0.007 (0.016)	0 (0.001)	0 (0)	0 (0.003)	0 (0.012)	0 (0.009)	0 (0.012)	0 (0.007)	0 (0)	0 (0.003)	0.008 (0.023)	0 (0.006)	0.051 (0.029)	0.289 (0.026)	0.148 (0.056)	0.229 (0.041)	0.109 (0.022)
ID0041715	OʻAHU	0 (0.016)	0.028 (0.019)	0.006 (0.006)	0.02 (0.017)	0 (0.005)	0 (0.005)	0 (0.008)	0 (0.019)	0 (0.003)	0 (0)	0.012 (0.019)	0.061 (0.035)	0 (0.008)	0 (0.003)	0 (0.002)	0 (0.005)	0.038 (0.031)	0.015 (0.019)	0 (0.012)	0.284 (0.034)	0.172 (0.051)	0.268 (0.038)	0.095 (0.025)
ID0041722	OʻAHU	0.03 (0.022)	0.014 (0.015)	0.013 (0.006)	0.02 (0.017)	0.004 (0.011)	0.048 (0.026)	0.003 (0.016)	0.071 (0.045)	0 (0)	0 (0.015)	0 (0.01)	0.008 (0.018)	0 (0.015)	0 (0.004)	0.003 (0.006)	0 (0.004)	0.02 (0.015)	0 (0.002)	0 (0.01)	0.4 (0.025)	0 (0.018)	0.292 (0.041)	0.075 (0.02)
ID0041723	OʻAHU	0.029 (0.022)	0.003 (0.011)	0.012 (0.006)	0 (0.01)	0 (0.004)	0.035 (0.03)	0.001 (0.015)	0 (0.016)	0 (0)	0 (0.007)	0 (0.004)	0.052 (0.026)	0 (0.001)	0.012 (0.015)	0.011 (0.008)	0 (0.001)	0.042 (0.031)	0 (0.003)	0 (0.008)	0.277 (0.027)	0.119 (0.055)	0.312 (0.039)	0.094 (0.02)
ID0041726	OʻAHU	0.017 (0.018)	0.016 (0.015)	0.017 (0.008)	0.017 (0.012)	0 (0.006)	0.074 (0.026)	0 (0.009)	0.049 (0.037)	0 (0)	0 (0.011)	0.03 (0.019)	0 (0.016)	0 (0)	0 (0.005)	0 (0.004)	0 (0.001)	0.053 (0.026)	0 (0.006)	0 (0.014)	0.333 (0.029)	0 (0.012)	0.262 (0.037)	0.131 (0.02)
ID0041729	OʻAHU	0.005 (0.018)	0.029 (0.02)	0.027 (0.007)	0.016 (0.013)	0 (0.005)	0.022 (0.02)	0 (0.003)	0 (0.016)	0 (0.004)	0 (0)	0 (0.007)	0.033 (0.03)	0 (0.009)	0.014 (0.017)	0 (0.005)	0 (0.002)	0 (0.02)	0 (0.005)	0.048 (0.028)	0.354 (0.033)	0 (0.004)	0.322 (0.037)	0.13 (0.023)
ID0041732	MAUI	0.073 (0.025)	0.016 (0.016)	0.013 (0.007)	0.025 (0.017)	0.009 (0.014)	0.042 (0.025)	0 (0.004)	0 (0.015)	0.028 (0.023)	0 (0.008)	0.015 (0.015)	0.016 (0.018)	0 (0.022)	0 (0)	0.004 (0.006)	0 (0)	0.001 (0.014)	0 (0.001)	0.019 (0.019)	0.18 (0.027)	0.19 (0.058)	0.289 (0.041)	0.078 (0.021)
ID0041733	MAUI	0.046 (0.025)	0 (0.003)	0.006 (0.006)	0.027 (0.018)	0.013 (0.013)	0.023 (0.024)	0 (0.002)	0 (0.005)	0.042 (0.024)	0 (0.002)	0 (0.007)	0.012 (0.02)	0 (0.003)	0 (0)	0.005 (0.006)	0 (0.002)	0.022 (0.025)	0 (0.003)	0.043 (0.024)	0.138 (0.021)	0.231 (0.059)	0.302 (0.04)	0.089 (0.021)

ID0041739	O‘AHU	0 (0.003)	0.021 (0.018)	0.008 (0.006)	0.018 (0.015)	0.004 (0.009)	0.051 (0.032)	0.016 (0.022)	0 (0.027)	0 (0.003)	0 (0.006)	0 (0.012)	0 (0.022)	0.004 (0.02)	0.014 (0.014)	0 (0.006)	0 (0.002)	0.051 (0.03)	0.003 (0.01)	0.01 (0.018)	0.344 (0.036)	0.097 (0.053)	0.247 (0.043)	0.113 (0.024)
ID0041740	O‘AHU	0.031 (0.026)	0.018 (0.018)	0.008 (0.006)	0.03 (0.018)	0 (0.009)	0.066 (0.03)	0 (0.011)	0 (0.003)	0 (0.003)	0 (0)	0.017 (0.018)	0 (0.004)	0 (0.008)	0 (0.007)	0 (0.002)	0 (0.006)	0.035 (0.024)	0 (0.002)	0.023 (0.028)	0.239 (0.029)	0.069 (0.052)	0.324 (0.038)	0.138 (0.024)
ID0041741	O‘AHU	0.004 (0.02)	0.021 (0.02)	0.013 (0.008)	0 (0.005)	0.006 (0.012)	0.036 (0.027)	0.058 (0.026)	0 (0.001)	0 (0.004)	0 (0.008)	0 (0.006)	0.001 (0.015)	0 (0.017)	0 (0.001)	0.004 (0.006)	0 (0.004)	0.048 (0.025)	0 (0.005)	0 (0.018)	0.277 (0.03)	0.13 (0.058)	0.284 (0.04)	0.118 (0.027)
ID0041742	O‘AHU	0.045 (0.025)	0 (0.009)	0 (0.004)	0.021 (0.016)	0 (0.008)	0.068 (0.026)	0.046 (0.025)	0 (0.005)	0 (0.005)	0 (0.004)	0.031 (0.023)	0.003 (0.02)	0 (0.017)	0 (0)	0.002 (0.005)	0 (0.001)	0 (0.007)	0 (0.007)	0.05 (0.029)	0.266 (0.032)	0.08 (0.048)	0.268 (0.046)	0.119 (0.024)
ID0041743	O‘AHU	0.041 (0.024)	0.018 (0.02)	0.004 (0.005)	0.019 (0.016)	0 (0.007)	0.033 (0.023)	0.031 (0.023)	0 (0)	0 (0.002)	0 (0.001)	0 (0.014)	0.017 (0.024)	0 (0.008)	0 (0)	0.005 (0.006)	0 (0.003)	0 (0.014)	0 (0.011)	0.05 (0.03)	0.249 (0.029)	0.165 (0.053)	0.255 (0.042)	0.114 (0.022)
ID0041744	O‘AHU	0.022 (0.024)	0.006 (0.014)	0.015 (0.007)	0.008 (0.013)	0.014 (0.014)	0.095 (0.032)	0.021 (0.02)	0 (0)	0 (0.004)	0 (0.004)	0.026 (0.019)	0 (0.005)	0.043 (0.028)	0 (0.001)	0 (0.002)	0 (0.003)	0.025 (0.027)	0 (0.004)	0.068 (0.033)	0.231 (0.028)	0.065 (0.048)	0.232 (0.04)	0.129 (0.023)
ID0041745	O‘AHU	0.023 (0.023)	0.005 (0.012)	0.007 (0.006)	0.01 (0.014)	0.008 (0.014)	0.083 (0.025)	0 (0)	0 (0.005)	0 (0)	0 (0.007)	0.003 (0.012)	0 (0.013)	0 (0.014)	0 (0.002)	0.004 (0.007)	0 (0.005)	0.102 (0.031)	0 (0.007)	0 (0.022)	0.24 (0.03)	0.145 (0.049)	0.25 (0.037)	0.12 (0.025)
ID0041747	O‘AHU	0.051 (0.025)	0.022 (0.018)	0.017 (0.006)	0 (0.009)	0 (0.007)	0.074 (0.026)	0 (0.002)	0 (0)	0 (0.002)	0 (0.015)	0.031 (0.02)	0 (0.011)	0 (0)	0 (0)	0 (0.003)	0 (0.003)	0.048 (0.025)	0 (0.005)	0 (0.016)	0.31 (0.029)	0.052 (0.036)	0.267 (0.035)	0.128 (0.024)
ID0041749	O‘AHU	0.054 (0.022)	0.038 (0.024)	0.003 (0.005)	0.032 (0.022)	0.022 (0.015)	0 (0.015)	0 (0.004)	0 (0.02)	0.008 (0.016)	0.03 (0.027)	0.046 (0.021)	0.035 (0.027)	0.006 (0.019)	0 (0.004)	0 (0.005)	0 (0.002)	0.051 (0.033)	0 (0.005)	0 (0.011)	0.265 (0.036)	0.073 (0.054)	0.266 (0.039)	0.071 (0.023)
ID0041756	MAUI	0.045 (0.031)	0 (0.006)	0.01 (0.006)	0.008 (0.011)	0.026 (0.016)	0.06 (0.023)	0 (0)	0 (0.007)	0 (0.014)	0 (0.004)	0.01 (0.014)	0.059 (0.027)	0.008 (0.02)	0 (0.004)	0.011 (0.008)	0 (0.001)	0.02 (0.023)	0 (0.004)	0 (0.009)	0.132 (0.025)	0.22 (0.062)	0.288 (0.039)	0.103 (0.025)
ID0041760	MAUI	0.031 (0.023)	0 (0)	0.007 (0.006)	0.032 (0.019)	0.019 (0.015)	0.035 (0.027)	0 (0)	0 (0.016)	0.048 (0.025)	0 (0.017)	0.026 (0.019)	0 (0.008)	0 (0.007)	0 (0.003)	0.003 (0.007)	0.002 (0.007)	0.003 (0.019)	0 (0)	0.029 (0.021)	0.16 (0.029)	0.275 (0.062)	0.198 (0.041)	0.132 (0.021)
ID0041762	MAUI	0.022 (0.023)	0 (0.008)	0.009 (0.006)	0.004 (0.012)	0.008 (0.012)	0.047 (0.026)	0 (0.002)	0 (0.007)	0.019 (0.019)	0 (0.006)	0 (0.005)	0.047 (0.025)	0 (0.004)	0 (0)	0.017 (0.01)	0 (0.003)	0.057 (0.029)	0 (0)	0 (0.009)	0.119 (0.025)	0.324 (0.055)	0.216 (0.035)	0.112 (0.022)
ID0041767	O‘AHU	0.037 (0.023)	0 (0.008)	0.013 (0.007)	0.018 (0.014)	0.01 (0.013)	0.026 (0.023)	0.011 (0.016)	0 (0.025)	0 (0)	0 (0.015)	0.028 (0.019)	0 (0.003)	0 (0.002)	0.013 (0.017)	0 (0.002)	0 (0.002)	0.007 (0.025)	0 (0.009)	0.036 (0.025)	0.28 (0.027)	0.123 (0.052)	0.262 (0.037)	0.136 (0.027)
ID0041770	MAUI	0.021 (0.023)	0 (0.002)	0.016 (0.007)	0 (0.011)	0.026 (0.013)	0.012 (0.018)	0 (0)	0 (0.006)	0.032 (0.022)	0 (0.005)	0.001 (0.01)	0.028 (0.024)	0 (0.004)	0 (0)	0 (0.004)	0 (0.005)	0.028 (0.025)	0 (0.002)	0.025 (0.024)	0.167 (0.027)	0.259 (0.05)	0.279 (0.038)	0.105 (0.021)
ID0041773	MAUI	0.044 (0.026)	0 (0.008)	0.003 (0.005)	0.02 (0.017)	0.021 (0.014)	0.035 (0.026)	0 (0.001)	0 (0)	0.027 (0.023)	0 (0.001)	0 (0.008)	0.006 (0.02)	0 (0.007)	0 (0)	0.001 (0.005)	0.002 (0.007)	0.037 (0.025)	0 (0.004)	0.02 (0.025)	0.161 (0.026)	0.301 (0.057)	0.208 (0.042)	0.115 (0.021)
ID0041774	MAUI	0.105 (0.026)	0.001 (0.011)	0.002 (0.005)	0.036 (0.016)	0.023 (0.014)	0.013 (0.02)	0 (0)	0 (0.007)	0.056 (0.029)	0 (0)	0 (0.011)	0.001 (0.013)	0 (0.019)	0 (0)	0 (0.004)	0 (0.003)	0.013 (0.017)	0 (0)	0.031 (0.024)	0.094 (0.026)	0.444 (0.058)	0.146 (0.04)	0.035 (0.021)
ID0041776	MAUI	0.119 (0.028)	0 (0.008)	0.007 (0.005)	0.001 (0.012)	0.007 (0.013)	0.109 (0.034)	0 (0.003)	0 (0.007)	0.021 (0.021)	0 (0.008)	0 (0.004)	0 (0.013)	0 (0)	0 (0)	0.004 (0.006)	0.001 (0.005)	0.027 (0.023)	0 (0.005)	0.014 (0.022)	0.146 (0.026)	0.222 (0.063)	0.217 (0.039)	0.107 (0.024)
ID0041796	O‘AHU	0.032 (0.022)	0.041 (0.022)	0.013 (0.007)	0.025 (0.017)	0.004 (0.01)	0.048 (0.027)	0 (0.002)	0.008 (0.032)	0 (0.001)	0 (0.006)	0.012 (0.013)	0.033 (0.027)	0 (0.012)	0 (0.006)	0.002 (0.006)	0 (0.002)	0.032 (0.025)	0 (0.005)	0 (0.018)	0.308 (0.03)	0.042 (0.046)	0.286 (0.044)	0.116 (0.024)
ID0041799	O‘AHU	0.06 (0.029)	0.034 (0.02)	0.002 (0.005)	0.032 (0.015)	0.006 (0.013)	0.018 (0.023)	0 (0.005)	0 (0)	0 (0.008)	0 (0.007)	0 (0.012)	0.031 (0.029)	0 (0.01)	0 (0)	0.013 (0.008)	0 (0.005)	0.016 (0.02)	0 (0.008)	0 (0.003)	0.278 (0.027)	0.096 (0.046)	0.283 (0.038)	0.132 (0.021)
ID0041802	O‘AHU	0.023 (0.023)	0.003 (0.014)	0.011 (0.006)	0.024 (0.018)	0.015 (0.013)	0.028 (0.025)	0 (0.012)	0 (0.013)	0 (0)	0 (0.004)	0 (0.007)	0.017 (0.022)	0 (0.019)	0 (0.005)	0 (0.003)	0 (0.001)	0.022 (0.02)	0 (0.003)	0 (0.012)	0.368 (0.03)	0.131 (0.053)	0.246 (0.035)	0.111 (0.028)

ID0041803	MAUI	0.012 (0.021)	0 (0.008)	0.004 (0.006)	0.034 (0.02)	0.006 (0.012)	0.031 (0.025)	0 (0.001)	0 (0.006)	0.006 (0.016)	0 (0.004)	0.017 (0.019)	0 (0.007)	0 (0.021)	0 (0)	0.012 (0.007)	0.001 (0.006)	0.043 (0.026)	0 (0.004)	0 (0.013)	0.161 (0.027)	0.236 (0.055)	0.319 (0.034)	0.119 (0.023)
ID0041804	MAUI	0 (0.013)	0 (0.004)	0.009 (0.006)	0.019 (0.017)	0.025 (0.018)	0.027 (0.023)	0 (0)	0 (0.008)	0 (0.012)	0 (0.004)	0.011 (0.017)	0 (0.012)	0 (0.005)	0 (0)	0.004 (0.006)	0 (0.001)	0.056 (0.029)	0 (0.003)	0 (0.009)	0.149 (0.03)	0.304 (0.055)	0.291 (0.041)	0.106 (0.028)
ID0041805	MAUI	0.029 (0.025)	0 (0.002)	0.011 (0.008)	0.057 (0.021)	0.017 (0.015)	0.002 (0.016)	0 (0)	0 (0)	0.054 (0.03)	0.02 (0.025)	0 (0.009)	0 (0.015)	0 (0.012)	0 (0)	0 (0.005)	0 (0.001)	0 (0.011)	0 (0)	0.015 (0.017)	0.18 (0.028)	0.216 (0.068)	0.286 (0.044)	0.11 (0.027)
ID0041806	MAUI	0.038 (0.024)	0 (0.009)	0.007 (0.005)	0.012 (0.01)	0 (0.009)	0.019 (0.02)	0 (0)	0 (0.018)	0.033 (0.024)	0 (0.015)	0 (0.009)	0.01 (0.018)	0 (0.007)	0 (0)	0.021 (0.008)	0 (0)	0.015 (0.019)	0 (0)	0.006 (0.017)	0.161 (0.023)	0.281 (0.059)	0.264 (0.042)	0.133 (0.023)
ID0041807	MAUI	0.026 (0.024)	0 (0.01)	0.01 (0.007)	0.021 (0.018)	0.017 (0.016)	0.02 (0.021)	0 (0.016)	0 (0.005)	0 (0.017)	0 (0.003)	0.022 (0.019)	0 (0.015)	0 (0.003)	0 (0)	0.017 (0.009)	0 (0.004)	0.03 (0.022)	0 (0.002)	0 (0.016)	0.14 (0.028)	0.241 (0.056)	0.328 (0.042)	0.127 (0.025)
ID0041808	MAUI	0.003 (0.019)	0 (0.002)	0.008 (0.007)	0.027 (0.019)	0.016 (0.018)	0.009 (0.015)	0 (0.006)	0 (0.009)	0.002 (0.014)	0 (0.01)	0.04 (0.022)	0 (0.005)	0 (0.004)	0 (0)	0.012 (0.008)	0 (0)	0.041 (0.027)	0 (0.002)	0 (0.016)	0.153 (0.029)	0.283 (0.05)	0.299 (0.038)	0.107 (0.028)
ID0041810	MAUI	0.007 (0.019)	0 (0.006)	0.014 (0.008)	0.024 (0.018)	0.011 (0.015)	0 (0.013)	0 (0.003)	0 (0.007)	0 (0.012)	0 (0.003)	0.028 (0.021)	0 (0.011)	0.002 (0.017)	0 (0)	0.007 (0.007)	0 (0.001)	0.06 (0.029)	0 (0.005)	0 (0.015)	0.12 (0.031)	0.286 (0.055)	0.31 (0.043)	0.132 (0.028)
ID0041814	MAUI	0.023 (0.028)	0 (0.007)	0.012 (0.008)	0.026 (0.019)	0.01 (0.012)	0.037 (0.024)	0 (0.002)	0 (0.008)	0.03 (0.02)	0 (0.003)	0 (0.011)	0.018 (0.022)	0 (0.017)	0 (0)	0.004 (0.007)	0 (0)	0.052 (0.031)	0 (0)	0.018 (0.022)	0.158 (0.032)	0.096 (0.056)	0.348 (0.04)	0.169 (0.028)
ID0041815	MAUI	0.021 (0.024)	0 (0.004)	0.011 (0.007)	0 (0.003)	0.024 (0.015)	0.019 (0.024)	0 (0)	0 (0.001)	0.023 (0.018)	0 (0.01)	0.015 (0.017)	0.026 (0.021)	0 (0.011)	0 (0)	0.019 (0.008)	0 (0.001)	0.038 (0.029)	0 (0.001)	0.012 (0.024)	0.108 (0.029)	0.274 (0.066)	0.296 (0.04)	0.112 (0.028)
ID0041816	MAUI	0 (0.016)	0 (0.006)	0.01 (0.007)	0.025 (0.018)	0.004 (0.012)	0.052 (0.028)	0 (0.003)	0 (0.005)	0.018 (0.021)	0 (0.01)	0.027 (0.02)	0.028 (0.024)	0 (0.012)	0 (0)	0 (0.004)	0 (0.002)	0.009 (0.018)	0 (0)	0.014 (0.016)	0.166 (0.026)	0.222 (0.049)	0.301 (0.039)	0.125 (0.021)
ID0041817	MAUI	0.034 (0.024)	0 (0.002)	0.012 (0.008)	0.035 (0.02)	0.025 (0.016)	0 (0.014)	0 (0.005)	0 (0)	0.016 (0.023)	0 (0.018)	0.008 (0.015)	0 (0.011)	0 (0.003)	0 (0.003)	0.005 (0.006)	0 (0.002)	0.022 (0.023)	0 (0)	0 (0.01)	0.186 (0.029)	0.298 (0.064)	0.281 (0.045)	0.078 (0.027)
ID0041818	MAUI	0.021 (0.024)	0 (0.002)	0.011 (0.008)	0.03 (0.019)	0.027 (0.015)	0.003 (0.013)	0 (0.009)	0 (0.003)	0.012 (0.017)	0 (0.007)	0.028 (0.021)	0.014 (0.019)	0 (0.001)	0 (0)	0.009 (0.008)	0 (0.002)	0 (0.013)	0 (0)	0.028 (0.022)	0.162 (0.03)	0.216 (0.05)	0.307 (0.041)	0.133 (0.024)
ID0041819	MAUI	0.047 (0.03)	0 (0.009)	0.003 (0.005)	0.006 (0.012)	0.005 (0.011)	0 (0.012)	0 (0.001)	0 (0.008)	0.016 (0.024)	0 (0.013)	0.028 (0.019)	0 (0.007)	0 (0.003)	0 (0)	0.017 (0.009)	0 (0)	0.052 (0.028)	0 (0.006)	0 (0.017)	0.149 (0.029)	0.287 (0.063)	0.27 (0.044)	0.118 (0.026)
ID0041820	MAUI	0.033 (0.024)	0 (0.005)	0.019 (0.008)	0.019 (0.015)	0.023 (0.015)	0.034 (0.026)	0 (0)	0 (0.004)	0.013 (0.018)	0.003 (0.021)	0.031 (0.021)	0 (0.005)	0 (0.006)	0 (0)	0 (0.005)	0 (0)	0.042 (0.027)	0 (0.001)	0.016 (0.023)	0.136 (0.027)	0.263 (0.055)	0.244 (0.043)	0.123 (0.027)
ID0041822	O‘AHU	0.045 (0.027)	0.029 (0.019)	0.016 (0.007)	0 (0.007)	0.003 (0.01)	0.083 (0.03)	0 (0)	0 (0.012)	0 (0.002)	0 (0.005)	0.023 (0.018)	0 (0.014)	0 (0.004)	0 (0.003)	0.006 (0.008)	0 (0)	0.042 (0.024)	0 (0.007)	0 (0.012)	0.288 (0.027)	0.089 (0.055)	0.235 (0.047)	0.141 (0.023)
ID0041824	MAUI	0 (0.014)	0 (0)	0.003 (0.006)	0.047 (0.022)	0.033 (0.016)	0.017 (0.021)	0 (0)	0 (0)	0.042 (0.021)	0 (0.009)	0.018 (0.017)	0.023 (0.024)	0 (0.003)	0 (0)	0.013 (0.008)	0 (0.002)	0.026 (0.024)	0 (0.004)	0 (0.007)	0.162 (0.03)	0.262 (0.065)	0.273 (0.042)	0.08 (0.027)
ID0041825	MAUI	0.052 (0.027)	0 (0.002)	0.007 (0.007)	0.021 (0.018)	0.018 (0.015)	0.013 (0.017)	0 (0.005)	0.023 (0.033)	0.022 (0.021)	0 (0.019)	0.048 (0.026)	0 (0.006)	0 (0)	0 (0)	0.008 (0.007)	0 (0)	0.047 (0.032)	0 (0.001)	0.024 (0.026)	0.164 (0.032)	0.171 (0.055)	0.29 (0.039)	0.091 (0.026)
ID0041826	MAUI	0.039 (0.027)	0 (0)	0.005 (0.006)	0.028 (0.019)	0.032 (0.015)	0.006 (0.016)	0 (0.005)	0.021 (0.031)	0.03 (0.02)	0 (0.007)	0.041 (0.023)	0.006 (0.018)	0 (0.01)	0 (0)	0.01 (0.008)	0 (0)	0.045 (0.029)	0 (0)	0.016 (0.022)	0.143 (0.031)	0.159 (0.059)	0.309 (0.041)	0.111 (0.028)
ID0041829	MAUI	0.07 (0.03)	0.019 (0.016)	0.012 (0.007)	0.038 (0.02)	0.008 (0.013)	0.026 (0.019)	0 (0.005)	0 (0.001)	0.026 (0.022)	0 (0.012)	0.002 (0.011)	0.01 (0.015)	0 (0.004)	0 (0)	0.004 (0.005)	0 (0.005)	0.029 (0.022)	0 (0.004)	0 (0.015)	0.15 (0.022)	0.16 (0.054)	0.321 (0.038)	0.125 (0.023)
ID0041830	MAUI	0.026 (0.023)	0 (0.005)	0.011 (0.007)	0.012 (0.014)	0.032 (0.016)	0.01 (0.013)	0 (0.008)	0 (0)	0 (0.01)	0 (0)	0.035 (0.021)	0 (0.011)	0 (0.003)	0 (0)	0.015 (0.009)	0 (0)	0.043 (0.023)	0 (0.003)	0 (0.007)	0.158 (0.032)	0.274 (0.05)	0.281 (0.039)	0.102 (0.025)

ID0041836	MAUI	0 (0.012)	0 (0.007)	0.002 (0.005)	0.033 (0.021)	0.03 (0.017)	0.05 (0.028)	0 (0.003)	0 (0.002)	0.022 (0.024)	0 (0.005)	0.028 (0.021)	0.009 (0.017)	0.021 (0.03)	0 (0.001)	0.006 (0.006)	0 (0.003)	0.027 (0.026)	0 (0.005)	0 (0.007)	0.176 (0.03)	0.2 (0.06)	0.295 (0.043)	0.1 (0.025)
ID0041837	MAUI	0 (0.013)	0 (0.002)	0.011 (0.008)	0.039 (0.021)	0.019 (0.016)	0.039 (0.026)	0 (0)	0 (0)	0.022 (0.019)	0 (0)	0.025 (0.02)	0 (0.011)	0 (0.001)	0 (0)	0.014 (0.009)	0 (0.001)	0.016 (0.021)	0 (0.003)	0 (0.014)	0.166 (0.028)	0.279 (0.063)	0.287 (0.041)	0.084 (0.027)
ID0041838	MAUI	0.037 (0.028)	0 (0.011)	0.013 (0.007)	0.004 (0.013)	0.002 (0.013)	0.018 (0.023)	0 (0.012)	0 (0.02)	0.003 (0.014)	0 (0.007)	0.04 (0.02)	0.003 (0.014)	0 (0.005)	0 (0)	0.015 (0.008)	0 (0.002)	0.048 (0.029)	0 (0.004)	0 (0.015)	0.159 (0.033)	0.252 (0.057)	0.289 (0.042)	0.117 (0.024)
ID0041839	O‘AHU	0.037 (0.024)	0.007 (0.015)	0.005 (0.006)	0.015 (0.015)	0 (0.007)	0.031 (0.02)	0 (0)	0 (0.012)	0 (0.002)	0 (0.001)	0.015 (0.017)	0 (0.014)	0 (0.003)	0 (0.006)	0 (0.006)	0.009 (0.008)	0.036 (0.029)	0 (0.003)	0.014 (0.026)	0.273 (0.029)	0.162 (0.052)	0.302 (0.043)	0.096 (0.024)
ID0041840	MAUI	0 (0.016)	0 (0.001)	0.012 (0.008)	0.025 (0.019)	0.021 (0.019)	0.045 (0.029)	0 (0.013)	0 (0.007)	0.023 (0.024)	0 (0.004)	0.01 (0.016)	0.026 (0.022)	0 (0.007)	0 (0)	0.002 (0.005)	0 (0.001)	0 (0.015)	0 (0.001)	0.021 (0.022)	0.172 (0.031)	0.279 (0.058)	0.231 (0.043)	0.134 (0.032)
ID0041841	MAUI	0.019 (0.023)	0 (0.007)	0.007 (0.007)	0.047 (0.022)	0.021 (0.017)	0.06 (0.028)	0 (0.015)	0 (0.01)	0 (0.011)	0 (0.003)	0.019 (0.018)	0.044 (0.027)	0 (0.002)	0 (0)	0.006 (0.007)	0 (0.003)	0.022 (0.025)	0 (0)	0 (0.007)	0.116 (0.033)	0.292 (0.055)	0.237 (0.046)	0.109 (0.024)
ID0041842	MAUI	0.022 (0.02)	0 (0.006)	0.006 (0.007)	0.05 (0.02)	0.028 (0.016)	0.019 (0.019)	0 (0.006)	0 (0)	0 (0.01)	0 (0.002)	0.03 (0.019)	0.013 (0.02)	0 (0.005)	0 (0)	0.019 (0.009)	0 (0.001)	0.027 (0.024)	0 (0)	0 (0.015)	0.141 (0.03)	0.284 (0.051)	0.258 (0.036)	0.104 (0.028)
ID0041843	MAUI	0.042 (0.029)	0 (0.005)	0.011 (0.007)	0.017 (0.016)	0.007 (0.013)	0 (0.014)	0 (0.011)	0 (0.006)	0 (0.012)	0 (0.01)	0.027 (0.017)	0.001 (0.013)	0 (0.001)	0 (0)	0.003 (0.006)	0 (0.001)	0.091 (0.033)	0 (0)	0.015 (0.023)	0.092 (0.033)	0.277 (0.057)	0.27 (0.04)	0.146 (0.032)
ID0041844	MAUI	0.018 (0.022)	0 (0.006)	0.01 (0.007)	0.021 (0.017)	0.028 (0.017)	0.048 (0.027)	0 (0.005)	0 (0)	0 (0.002)	0 (0.007)	0.011 (0.015)	0 (0.013)	0 (0.018)	0 (0)	0.013 (0.008)	0 (0.004)	0.054 (0.023)	0 (0.003)	0 (0.011)	0.121 (0.025)	0.226 (0.06)	0.307 (0.043)	0.145 (0.027)
ID0041845	MAUI	0.023 (0.02)	0 (0.008)	0.007 (0.007)	0.04 (0.018)	0.023 (0.017)	0.008 (0.016)	0 (0.001)	0 (0)	0.012 (0.016)	0 (0.02)	0.026 (0.02)	0.004 (0.018)	0 (0.007)	0 (0)	0.002 (0.005)	0 (0.002)	0.046 (0.025)	0 (0.001)	0 (0.003)	0.154 (0.028)	0.208 (0.049)	0.316 (0.039)	0.13 (0.026)
ID0044124	MAUI	0.02 (0.022)	0 (0.006)	0.014 (0.007)	0.019 (0.016)	0.007 (0.013)	0.03 (0.026)	0 (0.011)	0 (0.022)	0.011 (0.017)	0 (0.007)	0.046 (0.021)	0 (0.013)	0 (0.008)	0 (0.002)	0.007 (0.007)	0 (0.002)	0.03 (0.022)	0 (0.004)	0.002 (0.017)	0.16 (0.03)	0.244 (0.059)	0.299 (0.049)	0.112 (0.027)
ID0044126	MAUI	0 (0.013)	0 (0.001)	0.016 (0.007)	0.029 (0.019)	0.006 (0.014)	0.03 (0.026)	0 (0.014)	0 (0.006)	0.032 (0.023)	0 (0.006)	0.039 (0.02)	0.026 (0.023)	0 (0.007)	0 (0)	0.004 (0.006)	0 (0.001)	0 (0.012)	0 (0.002)	0 (0.013)	0.168 (0.029)	0.269 (0.062)	0.269 (0.044)	0.111 (0.027)
ID0044128	MAUI	0 (0.017)	0.005 (0.016)	0.001 (0.005)	0.027 (0.018)	0.025 (0.018)	0 (0.006)	0 (0.008)	0 (0.003)	0.011 (0.022)	0 (0.006)	0.057 (0.026)	0 (0.015)	0 (0.019)	0 (0.002)	0.004 (0.006)	0 (0.003)	0.054 (0.035)	0 (0.003)	0.03 (0.028)	0.143 (0.033)	0.277 (0.058)	0.262 (0.043)	0.105 (0.027)
ID0044130	MAUI	0.022 (0.023)	0 (0.006)	0.003 (0.006)	0.046 (0.02)	0.013 (0.013)	0.035 (0.024)	0 (0.013)	0 (0.019)	0.013 (0.021)	0 (0.005)	0.04 (0.023)	0 (0.014)	0 (0.009)	0 (0)	0.008 (0.007)	0 (0.001)	0.068 (0.034)	0 (0)	0.004 (0.019)	0.119 (0.032)	0.238 (0.059)	0.282 (0.042)	0.11 (0.024)
ID0044132	MAUI	0 (0.015)	0 (0.004)	0.007 (0.007)	0.041 (0.02)	0.016 (0.015)	0.046 (0.025)	0 (0)	0 (0)	0.013 (0.018)	0 (0.005)	0.005 (0.014)	0 (0.015)	0 (0.007)	0 (0)	0.001 (0.005)	0 (0.005)	0.022 (0.021)	0 (0)	0.017 (0.019)	0.173 (0.026)	0.259 (0.058)	0.281 (0.046)	0.118 (0.023)
ID0044134	MAUI	0.009 (0.018)	0 (0.007)	0.012 (0.007)	0.039 (0.02)	0.017 (0.016)	0.02 (0.02)	0 (0.013)	0 (0.035)	0.034 (0.026)	0 (0.008)	0.058 (0.026)	0.022 (0.023)	0 (0.017)	0 (0)	0 (0.003)	0 (0.001)	0 (0.014)	0 (0)	0.028 (0.022)	0.148 (0.031)	0.234 (0.057)	0.281 (0.044)	0.096 (0.026)
ID0044136	MAUI	0.042 (0.025)	0 (0.006)	0.008 (0.008)	0.043 (0.023)	0.033 (0.018)	0 (0.011)	0 (0.006)	0 (0)	0 (0.01)	0 (0.009)	0.038 (0.023)	0 (0.008)	0 (0.007)	0 (0)	0.014 (0.009)	0 (0.001)	0.064 (0.031)	0 (0.003)	0 (0.023)	0.134 (0.026)	0.245 (0.061)	0.256 (0.042)	0.122 (0.031)
ID0044138	MAUI	0.014 (0.022)	0 (0.004)	0.001 (0.006)	0.064 (0.019)	0.008 (0.013)	0.01 (0.016)	0 (0.009)	0 (0.025)	0.05 (0.033)	0 (0.015)	0.028 (0.02)	0 (0.017)	0 (0.004)	0 (0)	0 (0.006)	0 (0)	0.03 (0.024)	0 (0.001)	0.008 (0.016)	0.187 (0.031)	0.196 (0.071)	0.267 (0.043)	0.136 (0.029)
ID0044142	O‘AHU	0.05 (0.027)	0 (0.01)	0.007 (0.007)	0.029 (0.018)	0.006 (0.012)	0.012 (0.021)	0 (0.003)	0 (0.002)	0 (0.003)	0 (0.011)	0.048 (0.023)	0.007 (0.024)	0 (0.001)	0 (0)	0.001 (0.006)	0 (0)	0.034 (0.026)	0 (0.002)	0 (0.006)	0.27 (0.029)	0.175 (0.046)	0.22 (0.037)	0.141 (0.024)
ID0044144	O‘AHU	0.083 (0.025)	0 (0.009)	0.008 (0.006)	0.03 (0.016)	0.02 (0.016)	0.018 (0.019)	0.003 (0.017)	0.063 (0.042)	0 (0.008)	0.001 (0.016)	0.063 (0.021)	0 (0.009)	0 (0.01)	0 (0.005)	0 (0.003)	0 (0.005)	0.014 (0.021)	0 (0.003)	0.015 (0.018)	0.263 (0.024)	0.039 (0.04)	0.27 (0.035)	0.111 (0.022)

ID0044146	O‘AHU	0.035 (0.021)	0 (0.003)	0.02 (0.007)	0.006 (0.012)	0.027 (0.015)	0.046 (0.029)	0 (0.001)	0 (0.019)	0 (0.002)	0 (0.004)	0.046 (0.023)	0 (0.017)	0 (0.018)	0.003 (0.012)	0 (0.003)	0 (0)	0.018 (0.025)	0 (0.002)	0.048 (0.032)	0.317 (0.026)	0.09 (0.051)	0.229 (0.04)	0.115 (0.024)
ID0044148	O‘AHU	0 (0.008)	0.026 (0.018)	0.012 (0.007)	0.001 (0.008)	0 (0.008)	0.028 (0.025)	0 (0.006)	0 (0.022)	0 (0.006)	0 (0.005)	0.009 (0.014)	0.039 (0.029)	0.009 (0.021)	0 (0)	0 (0.003)	0 (0.003)	0.044 (0.029)	0 (0.001)	0 (0.013)	0.336 (0.037)	0.114 (0.051)	0.234 (0.043)	0.15 (0.02)
ID0044152	O‘AHU	0.016 (0.021)	0.023 (0.02)	0.012 (0.008)	0.035 (0.02)	0.006 (0.011)	0.011 (0.018)	0 (0.008)	0.006 (0.03)	0 (0.003)	0.012 (0.021)	0 (0.007)	0.048 (0.028)	0 (0.001)	0 (0.001)	0.001 (0.005)	0 (0)	0 (0.013)	0 (0.003)	0 (0)	0.365 (0.03)	0.12 (0.045)	0.242 (0.04)	0.102 (0.026)
ID0044154	MAUI	0 (0.011)	0 (0.001)	0.013 (0.008)	0.031 (0.019)	0.041 (0.018)	0.021 (0.021)	0 (0.004)	0 (0)	0.02 (0.018)	0 (0.014)	0.038 (0.021)	0 (0.013)	0 (0.016)	0 (0)	0.01 (0.008)	0 (0.001)	0.055 (0.032)	0 (0.001)	0 (0.012)	0.114 (0.029)	0.303 (0.055)	0.254 (0.041)	0.1 (0.03)
ID0044156	MAUI	0.036 (0.027)	0 (0.002)	0.017 (0.008)	0.035 (0.02)	0.025 (0.015)	0.022 (0.02)	0 (0.01)	0 (0.019)	0.037 (0.025)	0.016 (0.022)	0.044 (0.024)	0 (0.007)	0 (0.018)	0 (0)	0.018 (0.009)	0 (0)	0.017 (0.028)	0 (0)	0.02 (0.023)	0.14 (0.034)	0.179 (0.06)	0.272 (0.051)	0.121 (0.029)
ID0044159	MAUI	0.009 (0.02)	0 (0)	0.018 (0.007)	0.021 (0.016)	0.009 (0.014)	0.049 (0.029)	0 (0.004)	0 (0)	0.023 (0.02)	0 (0.001)	0.037 (0.022)	0 (0.009)	0 (0.001)	0 (0)	0.011 (0.008)	0 (0.002)	0.033 (0.025)	0 (0.001)	0 (0)	0.164 (0.03)	0.293 (0.063)	0.248 (0.043)	0.083 (0.025)
ID0044160	MAUI	0.05 (0.028)	0 (0.008)	0.01 (0.008)	0.038 (0.02)	0.023 (0.017)	0 (0.013)	0 (0)	0 (0.002)	0.031 (0.022)	0.037 (0.029)	0.012 (0.017)	0 (0.016)	0 (0.007)	0 (0)	0.004 (0.007)	0 (0)	0.055 (0.03)	0 (0)	0 (0.011)	0.147 (0.028)	0.171 (0.064)	0.28 (0.048)	0.14 (0.028)
ID0044162	MAUI	0.032 (0.026)	0 (0)	0.017 (0.008)	0.027 (0.02)	0.032 (0.016)	0.03 (0.022)	0 (0.005)	0 (0.015)	0.055 (0.023)	0 (0.01)	0.032 (0.021)	0 (0.017)	0 (0.01)	0 (0)	0.007 (0.007)	0 (0)	0.034 (0.034)	0 (0)	0.056 (0.032)	0.091 (0.027)	0.228 (0.061)	0.227 (0.043)	0.134 (0.028)
ID0044164	MAUI	0.017 (0.022)	0 (0.002)	0.005 (0.006)	0.021 (0.015)	0.035 (0.017)	0 (0.015)	0 (0.003)	0 (0)	0.013 (0.017)	0 (0.003)	0.019 (0.019)	0 (0.015)	0 (0.003)	0 (0)	0.007 (0.007)	0 (0.003)	0 (0.016)	0 (0.002)	0.042 (0.028)	0.142 (0.028)	0.267 (0.056)	0.31 (0.042)	0.122 (0.027)
ID0044166	MAUI	0.024 (0.022)	0 (0.003)	0.012 (0.007)	0.02 (0.017)	0.003 (0.012)	0 (0.007)	0 (0.004)	0 (0)	0.034 (0.026)	0 (0.009)	0.055 (0.022)	0 (0.018)	0 (0.006)	0 (0.001)	0.007 (0.007)	0 (0)	0.07 (0.029)	0 (0.001)	0 (0.013)	0.104 (0.031)	0.269 (0.058)	0.307 (0.043)	0.095 (0.023)
ID0044168	MAUI	0 (0.018)	0 (0.003)	0.011 (0.008)	0.021 (0.017)	0.033 (0.018)	0.032 (0.024)	0 (0.002)	0 (0)	0.004 (0.014)	0 (0.005)	0.034 (0.021)	0 (0.012)	0 (0.005)	0 (0.001)	0.005 (0.006)	0 (0.001)	0.018 (0.024)	0 (0.008)	0.039 (0.029)	0.169 (0.026)	0.265 (0.048)	0.294 (0.037)	0.076 (0.026)
ID0044170	MAUI	0.01 (0.02)	0 (0.005)	0.016 (0.009)	0.031 (0.021)	0.032 (0.018)	0.007 (0.016)	0 (0.005)	0 (0.007)	0.042 (0.027)	0 (0.01)	0.036 (0.021)	0 (0.011)	0 (0)	0 (0)	0 (0.004)	0 (0.001)	0.001 (0.021)	0 (0)	0.038 (0.028)	0.19 (0.03)	0.202 (0.064)	0.297 (0.044)	0.098 (0.029)
ID0044172	MAUI	0.03 (0.027)	0 (0.003)	0.015 (0.007)	0 (0.009)	0.047 (0.018)	0.022 (0.022)	0 (0.003)	0 (0.006)	0.017 (0.02)	0 (0.003)	0.034 (0.022)	0.014 (0.019)	0 (0.007)	0 (0)	0.018 (0.009)	0 (0.004)	0 (0.016)	0 (0.006)	0.038 (0.027)	0.141 (0.026)	0.261 (0.063)	0.305 (0.04)	0.059 (0.027)
ID0044175	MAUI	0.009 (0.017)	0 (0)	0.008 (0.007)	0.036 (0.019)	0.038 (0.017)	0 (0)	0 (0.001)	0 (0)	0.019 (0.019)	0.014 (0.021)	0.017 (0.019)	0 (0.004)	0 (0)	0 (0)	0.012 (0.008)	0 (0.001)	0.01 (0.027)	0 (0)	0.048 (0.03)	0.143 (0.031)	0.219 (0.056)	0.336 (0.046)	0.09 (0.025)
ID0044177	MAUI	0.017 (0.023)	0 (0.003)	0.012 (0.007)	0.036 (0.021)	0.018 (0.016)	0.046 (0.026)	0 (0.014)	0 (0.005)	0.006 (0.017)	0 (0.005)	0.035 (0.021)	0 (0.011)	0 (0.006)	0 (0)	0.012 (0.008)	0 (0.001)	0.046 (0.027)	0 (0)	0 (0.013)	0.126 (0.031)	0.249 (0.057)	0.297 (0.043)	0.101 (0.024)
ID0044179	MAUI	0.017 (0.022)	0 (0.001)	0.016 (0.008)	0.02 (0.017)	0.032 (0.017)	0 (0.012)	0 (0.005)	0 (0.004)	0.005 (0.016)	0 (0.014)	0.036 (0.023)	0.001 (0.013)	0 (0.013)	0 (0.001)	0.004 (0.007)	0 (0.001)	0.032 (0.024)	0 (0.004)	0.006 (0.014)	0.15 (0.031)	0.319 (0.061)	0.264 (0.04)	0.099 (0.028)
ID0044181	MAUI	0.024 (0.026)	0 (0.002)	0 (0.005)	0.037 (0.018)	0.004 (0.013)	0.031 (0.026)	0 (0.002)	0 (0.005)	0.007 (0.017)	0 (0.011)	0.013 (0.016)	0 (0.011)	0 (0.003)	0 (0)	0.014 (0.008)	0 (0.002)	0.031 (0.026)	0 (0)	0.01 (0.021)	0.14 (0.028)	0.263 (0.062)	0.292 (0.041)	0.136 (0.027)
ID0044183	MAUI	0.009 (0.021)	0 (0.002)	0.009 (0.008)	0.044 (0.021)	0.023 (0.015)	0.02 (0.018)	0 (0.007)	0 (0)	0.041 (0.026)	0 (0.015)	0.035 (0.021)	0.033 (0.024)	0 (0.003)	0 (0)	0.004 (0.006)	0 (0.001)	0.019 (0.022)	0 (0.003)	0 (0.012)	0.133 (0.027)	0.295 (0.061)	0.258 (0.044)	0.078 (0.026)
ID0044185	MAUI	0.014 (0.021)	0 (0.002)	0.008 (0.007)	0.018 (0.015)	0.052 (0.015)	0 (0.004)	0 (0.004)	0 (0.011)	0 (0.012)	0 (0.008)	0.045 (0.027)	0 (0.005)	0 (0.013)	0 (0.006)	0.01 (0.009)	0 (0.004)	0.062 (0.029)	0 (0)	0.005 (0.022)	0.132 (0.029)	0.317 (0.057)	0.273 (0.043)	0.064 (0.03)
ID0044187	O‘AHU	0 (0.014)	0 (0.011)	0.014 (0.007)	0.032 (0.02)	0.027 (0.015)	0.025 (0.021)	0 (0)	0 (0.018)	0 (0.007)	0 (0.003)	0.055 (0.018)	0 (0.016)	0 (0.005)	0 (0)	0 (0.001)	0 (0)	0.015 (0.016)	0 (0.002)	0 (0.007)	0.326 (0.03)	0.174 (0.042)	0.234 (0.038)	0.097 (0.024)

ID0044189	MAUI	0.034 (0.022)	0.004 (0.013)	0.003 (0.005)	0.049 (0.022)	0 (0.009)	0.008 (0.013)	0 (0.007)	0.002 (0.031)	0.006 (0.017)	0 (0.013)	0.042 (0.023)	0.011 (0.022)	0 (0.005)	0 (0)	0.006 (0.007)	0 (0)	0.026 (0.023)	0 (0)	0 (0.011)	0.146 (0.028)	0.235 (0.053)	0.317 (0.035)	0.111 (0.025)
ID0044191	MAUI	0.018 (0.024)	0 (0.006)	0.011 (0.008)	0.027 (0.019)	0.018 (0.015)	0.022 (0.02)	0.004 (0.018)	0 (0.027)	0.026 (0.023)	0 (0.004)	0.048 (0.024)	0 (0.009)	0 (0.005)	0 (0)	0.009 (0.007)	0 (0.004)	0.004 (0.021)	0 (0)	0.036 (0.029)	0.165 (0.03)	0.216 (0.06)	0.319 (0.038)	0.077 (0.026)
ID0044193	MAUI	0.042 (0.029)	0 (0.007)	0 (0.004)	0.001 (0.011)	0.012 (0.014)	0.003 (0.017)	0 (0)	0 (0.017)	0.01 (0.021)	0 (0.007)	0 (0.009)	0.016 (0.02)	0 (0.004)	0 (0.001)	0.025 (0.008)	0 (0)	0.01 (0.019)	0 (0.003)	0 (0.008)	0.172 (0.027)	0.273 (0.064)	0.295 (0.046)	0.141 (0.027)
ID0044195	MAUI	0.034 (0.024)	0.001 (0.011)	0.011 (0.007)	0.016 (0.015)	0.025 (0.016)	0.043 (0.025)	0 (0.001)	0 (0)	0 (0.008)	0 (0.009)	0.036 (0.021)	0 (0.003)	0 (0.002)	0 (0)	0.01 (0.008)	0 (0)	0.076 (0.029)	0 (0)	0 (0.018)	0.102 (0.029)	0.226 (0.057)	0.292 (0.042)	0.127 (0.029)
ID0044197	MAUI	0.052 (0.028)	0 (0.003)	0.008 (0.007)	0.025 (0.016)	0.035 (0.017)	0.014 (0.015)	0 (0.01)	0 (0.007)	0.054 (0.028)	0 (0.013)	0.006 (0.013)	0 (0.006)	0 (0.015)	0 (0)	0.012 (0.008)	0 (0.005)	0.037 (0.023)	0 (0)	0 (0.013)	0.172 (0.029)	0.251 (0.059)	0.252 (0.043)	0.082 (0.03)
ID0044199	MAUI	0.034 (0.027)	0 (0.006)	0.003 (0.006)	0.053 (0.022)	0.023 (0.015)	0.047 (0.026)	0 (0.011)	0 (0.012)	0.052 (0.027)	0 (0.016)	0.027 (0.022)	0.033 (0.025)	0 (0.025)	0 (0)	0.015 (0.008)	0 (0)	0 (0.013)	0 (0)	0 (0.012)	0.166 (0.031)	0.119 (0.057)	0.283 (0.04)	0.146 (0.026)
ID0044201	MAUI	0 (0.011)	0 (0.001)	0.012 (0.008)	0.016 (0.016)	0.027 (0.016)	0 (0.013)	0 (0.002)	0 (0.011)	0.011 (0.018)	0 (0.004)	0.012 (0.014)	0.019 (0.021)	0 (0.005)	0 (0)	0.013 (0.009)	0 (0.001)	0 (0.022)	0 (0.004)	0.052 (0.029)	0.15 (0.029)	0.343 (0.06)	0.223 (0.042)	0.122 (0.03)
ID0044203	MAUI	0.053 (0.025)	0 (0.009)	0.01 (0.006)	0.026 (0.017)	0.018 (0.016)	0.026 (0.023)	0 (0.003)	0 (0.003)	0.038 (0.02)	0 (0.011)	0.018 (0.019)	0.008 (0.02)	0 (0)	0 (0)	0 (0.002)	0 (0.004)	0 (0.011)	0 (0.005)	0.047 (0.029)	0.162 (0.026)	0.21 (0.057)	0.254 (0.034)	0.13 (0.024)
ID0044205	MAUI	0.003 (0.018)	0 (0.001)	0.012 (0.008)	0.037 (0.019)	0.013 (0.017)	0 (0.008)	0 (0.002)	0 (0.013)	0.009 (0.019)	0 (0.013)	0.05 (0.022)	0 (0.004)	0 (0.003)	0 (0.001)	0.012 (0.008)	0 (0.002)	0.002 (0.025)	0 (0)	0.077 (0.03)	0.149 (0.026)	0.259 (0.061)	0.259 (0.043)	0.118 (0.029)
ID0044215	O‘AHU	0.035 (0.021)	0.035 (0.02)	0.014 (0.007)	0.015 (0.014)	0.003 (0.01)	0.032 (0.02)	0.026 (0.021)	0 (0.024)	0 (0)	0 (0.013)	0.032 (0.019)	0 (0.011)	0 (0.012)	0 (0.002)	0 (0.005)	0 (0)	0.011 (0.019)	0 (0.002)	0.024 (0.023)	0.321 (0.033)	0.085 (0.051)	0.247 (0.045)	0.121 (0.023)
ID0044217	O‘AHU	0.053 (0.024)	0.018 (0.015)	0.019 (0.007)	0 (0.01)	0.026 (0.014)	0.049 (0.028)	0 (0.007)	0.087 (0.046)	0 (0)	0 (0.004)	0.062 (0.024)	0 (0.006)	0 (0.003)	0 (0.005)	0 (0.001)	0.001 (0.005)	0.011 (0.015)	0 (0)	0.002 (0.019)	0.357 (0.029)	0 (0.027)	0.256 (0.035)	0.059 (0.022)
ID0044219	O‘AHU	0.053 (0.023)	0 (0.008)	0.017 (0.008)	0.024 (0.019)	0.007 (0.012)	0 (0.004)	0.007 (0.012)	0.014 (0.032)	0 (0)	0 (0.007)	0.067 (0.023)	0 (0.015)	0 (0.009)	0 (0.008)	0.006 (0.007)	0 (0.004)	0.045 (0.025)	0 (0.004)	0 (0.007)	0.295 (0.029)	0.103 (0.046)	0.244 (0.037)	0.118 (0.023)
ID0044221	O‘AHU	0 (0.012)	0.008 (0.017)	0.02 (0.007)	0 (0.004)	0.004 (0.012)	0.085 (0.034)	0 (0.007)	0.01 (0.035)	0 (0.001)	0 (0.014)	0.006 (0.016)	0.021 (0.023)	0 (0.012)	0.003 (0.014)	0.006 (0.007)	0 (0.004)	0 (0.011)	0.008 (0.013)	0 (0.009)	0.353 (0.027)	0.131 (0.052)	0.254 (0.042)	0.09 (0.021)
ID0044223	O‘AHU	0 (0.015)	0.024 (0.018)	0.021 (0.006)	0 (0.008)	0.019 (0.015)	0.079 (0.026)	0 (0.005)	0 (0.001)	0 (0)	0 (0)	0.011 (0.015)	0.081 (0.029)	0 (0.002)	0.008 (0.017)	0 (0.002)	0 (0.003)	0 (0.013)	0 (0.007)	0 (0.013)	0.302 (0.03)	0.055 (0.043)	0.262 (0.037)	0.138 (0.025)
ID0044227	O‘AHU	0.057 (0.026)	0.03 (0.019)	0.009 (0.006)	0.021 (0.017)	0 (0.01)	0.01 (0.018)	0.012 (0.02)	0 (0.007)	0 (0)	0 (0)	0.029 (0.019)	0.021 (0.021)	0 (0.005)	0 (0)	0.007 (0.008)	0 (0.006)	0.05 (0.028)	0 (0.002)	0 (0.011)	0.299 (0.031)	0.046 (0.045)	0.273 (0.038)	0.136 (0.022)
ID0044233	O‘AHU	0.03 (0.024)	0.023 (0.017)	0.022 (0.007)	0 (0.009)	0 (0.008)	0.054 (0.029)	0.017 (0.019)	0 (0.009)	0 (0.001)	0 (0.002)	0 (0.008)	0 (0.01)	0.047 (0.029)	0 (0.003)	0 (0.003)	0.001 (0.007)	0.021 (0.027)	0 (0.002)	0.05 (0.031)	0.256 (0.027)	0.065 (0.049)	0.298 (0.038)	0.116 (0.025)
ID0044235	HAWAI‘I	0.052 (0.023)	0 (0.009)	0.009 (0.006)	0.046 (0.018)	0 (0.006)	0.047 (0.025)	0.029 (0.016)	0 (0.018)	0 (0)	0 (0.006)	0.032 (0.019)	0 (0.014)	0 (0.006)	0.002 (0.014)	0 (0.004)	0.005 (0.008)	0.061 (0.023)	0 (0)	0 (0.01)	0.2 (0.031)	0.066 (0.037)	0.342 (0.037)	0.109 (0.021)
ID0044237	HAWAI‘I	0.037 (0.024)	0.006 (0.013)	0.01 (0.006)	0.015 (0.015)	0 (0.009)	0 (0.01)	0.025 (0.022)	0 (0.004)	0 (0)	0.05 (0.03)	0.041 (0.02)	0 (0)	0 (0)	0 (0.006)	0 (0.005)	0.016 (0.011)	0.057 (0.026)	0 (0.005)	0 (0.014)	0.254 (0.027)	0.093 (0.043)	0.297 (0.038)	0.099 (0.023)
ID0044239	O‘AHU	0.033 (0.023)	0.049 (0.02)	0.013 (0.005)	0 (0.008)	0.015 (0.013)	0.019 (0.021)	0 (0.002)	0 (0.009)	0 (0)	0 (0.016)	0.01 (0.015)	0 (0.004)	0 (0.017)	0 (0.005)	0.006 (0.007)	0 (0.002)	0.038 (0.022)	0.001 (0.008)	0 (0.013)	0.341 (0.027)	0.045 (0.041)	0.286 (0.042)	0.146 (0.025)
ID0044241	O‘AHU	0 (0.014)	0.042 (0.021)	0.006 (0.005)	0.021 (0.015)	0.004 (0.011)	0.022 (0.019)	0 (0)	0 (0.023)	0 (0)	0 (0.001)	0.022 (0.017)	0 (0.016)	0.061 (0.029)	0 (0.004)	0.012 (0.009)	0 (0.002)	0.013 (0.016)	0 (0.002)	0 (0.012)	0.325 (0.025)	0.037 (0.044)	0.277 (0.037)	0.158 (0.021)

ID0044243	OʻAHU	0.016 (0.02)	0.018 (0.017)	0.014 (0.007)	0.02 (0.014)	0 (0.007)	0.004 (0.014)	0 (0)	0 (0.029)	0 (0)	0 (0)	0.014 (0.016)	0.034 (0.022)	0 (0.004)	0 (0.006)	0.011 (0.009)	0 (0.001)	0 (0.013)	0 (0.005)	0 (0.014)	0.348 (0.025)	0.119 (0.043)	0.294 (0.036)	0.107 (0.02)
ID0044244	OʻAHU	0 (0.013)	0.023 (0.018)	0.016 (0.007)	0 (0.006)	0 (0.005)	0.046 (0.024)	0 (0.005)	0.017 (0.033)	0 (0)	0 (0.001)	0.011 (0.015)	0.004 (0.017)	0 (0.005)	0.012 (0.013)	0 (0.004)	0.002 (0.006)	0.009 (0.014)	0.013 (0.012)	0 (0.007)	0.353 (0.028)	0.089 (0.045)	0.27 (0.035)	0.137 (0.022)
ID0050785	MAUI	0.017 (0.025)	0 (0.005)	0.004 (0.005)	0.024 (0.019)	0.017 (0.013)	0.041 (0.027)	0 (0.005)	0 (0.003)	0.068 (0.027)	0 (0)	0 (0.003)	0 (0.01)	0 (0.003)	0 (0)	0.002 (0.005)	0 (0.004)	0.045 (0.027)	0 (0.003)	0.018 (0.024)	0.149 (0.025)	0.311 (0.063)	0.185 (0.037)	0.119 (0.019)
ID0050786	MAUI	0.026 (0.025)	0 (0.009)	0.011 (0.007)	0.02 (0.017)	0.017 (0.014)	0.009 (0.016)	0 (0.002)	0 (0.006)	0.076 (0.026)	0 (0.002)	0 (0.009)	0 (0.004)	0 (0.008)	0 (0)	0 (0.005)	0 (0.003)	0.035 (0.026)	0 (0.002)	0.024 (0.025)	0.15 (0.024)	0.282 (0.057)	0.247 (0.035)	0.103 (0.023)
ID0050787	MAUI	0.049 (0.028)	0.021 (0.018)	0.007 (0.007)	0.029 (0.019)	0.009 (0.013)	0.047 (0.027)	0 (0.003)	0 (0.013)	0.04 (0.023)	0 (0.003)	0 (0)	0.012 (0.019)	0 (0.01)	0 (0)	0.011 (0.008)	0 (0)	0.055 (0.032)	0 (0)	0 (0.019)	0.139 (0.028)	0.241 (0.06)	0.246 (0.036)	0.094 (0.023)
ID0050789	OʻAHU	0.039 (0.023)	0.005 (0.011)	0.004 (0.005)	0.042 (0.018)	0.017 (0.013)	0.061 (0.025)	0 (0.014)	0 (0.004)	0 (0.004)	0 (0)	0 (0.011)	0.007 (0.013)	0 (0.001)	0 (0)	0.004 (0.005)	0 (0.001)	0.013 (0.019)	0 (0)	0 (0.014)	0.347 (0.029)	0.13 (0.051)	0.201 (0.037)	0.13 (0.026)
ID0050790	OʻAHU	0.016 (0.025)	0.018 (0.017)	0.003 (0.006)	0.049 (0.019)	0.016 (0.012)	0 (0.013)	0 (0.004)	0 (0.002)	0 (0.01)	0 (0.005)	0.02 (0.019)	0 (0.02)	0 (0.002)	0 (0)	0 (0.003)	0 (0.004)	0.016 (0.022)	0 (0.004)	0.069 (0.034)	0.248 (0.029)	0.206 (0.059)	0.187 (0.046)	0.151 (0.025)
ID0050797	OʻAHU	0.008 (0.018)	0.015 (0.018)	0.016 (0.007)	0.029 (0.019)	0.01 (0.012)	0.046 (0.026)	0 (0.001)	0 (0.014)	0 (0)	0 (0.017)	0.011 (0.014)	0 (0.017)	0 (0.007)	0.007 (0.014)	0 (0)	0 (0.001)	0.092 (0.036)	0 (0)	0.032 (0.03)	0.255 (0.033)	0.17 (0.044)	0.24 (0.04)	0.07 (0.022)
ID0050798	OʻAHU	0.056 (0.025)	0.03 (0.018)	0.014 (0.007)	0.01 (0.015)	0.005 (0.012)	0 (0.013)	0 (0.004)	0.013 (0.032)	0 (0.002)	0 (0)	0 (0.004)	0.039 (0.031)	0 (0.006)	0 (0.005)	0.011 (0.009)	0 (0)	0.076 (0.032)	0 (0)	0 (0.022)	0.251 (0.03)	0.039 (0.037)	0.314 (0.042)	0.14 (0.026)
ID0050800	OʻAHU	0.046 (0.024)	0 (0.001)	0.01 (0.006)	0.02 (0.015)	0 (0.008)	0.036 (0.023)	0 (0.009)	0 (0.017)	0 (0.001)	0 (0.005)	0.026 (0.018)	0.055 (0.027)	0 (0.006)	0 (0.004)	0.001 (0.005)	0 (0.003)	0.011 (0.023)	0 (0.003)	0.003 (0.017)	0.337 (0.028)	0.054 (0.039)	0.255 (0.035)	0.146 (0.021)
ID0050801	OʻAHU	0.037 (0.02)	0 (0.01)	0.014 (0.007)	0.04 (0.017)	0.039 (0.015)	0.063 (0.029)	0 (0.002)	0 (0.012)	0 (0)	0 (0.001)	0.002 (0.013)	0 (0.005)	0 (0.003)	0 (0.004)	0 (0.002)	0 (0.003)	0.057 (0.029)	0 (0.004)	0 (0.022)	0.319 (0.032)	0.098 (0.047)	0.211 (0.042)	0.12 (0.025)
ID0050828	OʻAHU	0.053 (0.025)	0.041 (0.022)	0.003 (0.006)	0.051 (0.019)	0.014 (0.012)	0.009 (0.021)	0 (0.005)	0 (0)	0 (0)	0 (0.012)	0 (0.011)	0.027 (0.026)	0 (0.016)	0 (0.001)	0 (0.005)	0 (0.003)	0.044 (0.027)	0 (0.007)	0 (0.002)	0.273 (0.032)	0.137 (0.056)	0.235 (0.044)	0.114 (0.023)
ID0050837	OʻAHU	0.028 (0.024)	0.017 (0.017)	0.003 (0.005)	0.02 (0.015)	0.002 (0.01)	0.111 (0.025)	0 (0.002)	0 (0.007)	0 (0)	0 (0.01)	0.013 (0.014)	0 (0.016)	0 (0.008)	0 (0.001)	0.005 (0.007)	0 (0.002)	0.034 (0.026)	0 (0.002)	0.034 (0.027)	0.25 (0.024)	0.096 (0.05)	0.248 (0.038)	0.138 (0.022)
ID0050856	OʻAHU	0.057 (0.024)	0.021 (0.017)	0.007 (0.007)	0.026 (0.019)	0 (0.004)	0.085 (0.027)	0.012 (0.018)	0 (0.004)	0 (0.002)	0 (0.003)	0.033 (0.022)	0.037 (0.027)	0 (0.004)	0 (0.003)	0.001 (0.004)	0 (0)	0.024 (0.025)	0 (0)	0.017 (0.026)	0.264 (0.031)	0.031 (0.043)	0.24 (0.038)	0.145 (0.023)
ID0050860	OʻAHU	0.073 (0.028)	0.041 (0.021)	0.024 (0.008)	0.037 (0.018)	0.017 (0.013)	0.003 (0.014)	0 (0.01)	0 (0)	0 (0.003)	0 (0)	0 (0.01)	0 (0.011)	0 (0.003)	0 (0.001)	0 (0.003)	0.003 (0.008)	0.09 (0.03)	0 (0.004)	0 (0.005)	0.237 (0.03)	0.102 (0.047)	0.29 (0.039)	0.085 (0.021)
ID0050862	OʻAHU	0.01 (0.019)	0.042 (0.018)	0.004 (0.006)	0.04 (0.018)	0 (0.003)	0 (0.009)	0.004 (0.017)	0 (0)	0 (0)	0 (0)	0.013 (0.016)	0.034 (0.023)	0 (0.006)	0 (0.008)	0 (0.006)	0 (0.007)	0.056 (0.027)	0 (0.001)	0 (0.003)	0.28 (0.031)	0.128 (0.048)	0.286 (0.036)	0.101 (0.019)
ID0050868	OʻAHU	0.042 (0.025)	0.006 (0.016)	0 (0.004)	0.012 (0.014)	0 (0.006)	0.046 (0.028)	0.033 (0.021)	0 (0.01)	0 (0)	0 (0.016)	0.022 (0.02)	0.004 (0.021)	0 (0.011)	0 (0)	0.003 (0.005)	0 (0.001)	0.033 (0.028)	0 (0.003)	0.035 (0.03)	0.257 (0.029)	0.107 (0.053)	0.284 (0.044)	0.116 (0.024)
ID0050870	OʻAHU	0.008 (0.02)	0.031 (0.018)	0.011 (0.006)	0.022 (0.016)	0 (0.004)	0.085 (0.028)	0 (0.01)	0 (0.009)	0 (0)	0 (0.005)	0.002 (0.013)	0 (0.019)	0 (0.017)	0 (0.002)	0 (0.002)	0 (0)	0.075 (0.028)	0 (0.005)	0 (0.009)	0.228 (0.031)	0.155 (0.057)	0.237 (0.036)	0.147 (0.023)
ID0050873	OʻAHU	0.02 (0.023)	0.008 (0.013)	0.013 (0.006)	0 (0.007)	0 (0.005)	0.067 (0.032)	0 (0.008)	0 (0)	0 (0)	0 (0)	0.012 (0.017)	0 (0.002)	0 (0)	0 (0)	0.003 (0.006)	0 (0.004)	0.066 (0.028)	0 (0.004)	0 (0.004)	0.266 (0.034)	0.188 (0.043)	0.218 (0.04)	0.138 (0.023)
ID0050875	OʻAHU	0.043 (0.027)	0.031 (0.019)	0.001 (0.004)	0.005 (0.01)	0.006 (0.013)	0.105 (0.028)	0.006 (0.016)	0 (0.006)	0 (0.011)	0 (0.003)	0.005 (0.011)	0.006 (0.019)	0 (0.009)	0 (0.001)	0 (0.004)	0 (0.004)	0.042 (0.025)	0 (0.001)	0.002 (0.018)	0.279 (0.028)	0.149 (0.048)	0.189 (0.037)	0.131 (0.028)

ID0050891	O‘AHU	0.004 (0.016)	0 (0.008)	0.011 (0.007)	0.023 (0.017)	0 (0.006)	0.037 (0.024)	0 (0.001)	0 (0)	0 (0.003)	0 (0)	0.024 (0.017)	0 (0.012)	0 (0.013)	0 (0.002)	0 (0.003)	0 (0.001)	0.014 (0.021)	0 (0.004)	0.016 (0.02)	0.298 (0.025)	0.157 (0.044)	0.259 (0.038)	0.156 (0.022)
ID0050910	O‘AHU	0.053 (0.025)	0.017 (0.018)	0.001 (0.004)	0.014 (0.015)	0 (0.007)	0.037 (0.028)	0 (0.006)	0 (0.003)	0.006 (0.013)	0 (0.009)	0 (0.009)	0.007 (0.019)	0 (0.012)	0 (0)	0.002 (0.006)	0 (0.004)	0.078 (0.028)	0 (0.007)	0 (0.015)	0.271 (0.03)	0.083 (0.05)	0.301 (0.038)	0.13 (0.026)
ID0050911	O‘AHU	0.022 (0.023)	0.016 (0.018)	0.011 (0.007)	0.045 (0.018)	0.013 (0.012)	0.029 (0.023)	0 (0.011)	0 (0.008)	0 (0.005)	0 (0)	0.003 (0.014)	0.024 (0.023)	0 (0.013)	0 (0.003)	0 (0.003)	0 (0.002)	0.033 (0.024)	0 (0.01)	0 (0.007)	0.249 (0.037)	0.225 (0.055)	0.226 (0.038)	0.104 (0.023)
ID0050917	O‘AHU	0.079 (0.024)	0.035 (0.018)	0.012 (0.006)	0 (0.012)	0 (0.007)	0.11 (0.034)	0.043 (0.026)	0.013 (0.038)	0 (0)	0 (0.004)	0 (0.009)	0.025 (0.027)	0 (0.011)	0 (0.007)	0 (0.002)	0 (0.002)	0.068 (0.026)	0 (0.002)	0 (0.011)	0.251 (0.025)	0.037 (0.047)	0.229 (0.047)	0.099 (0.024)
ID0050919	O‘AHU	0 (0.009)	0.023 (0.019)	0.017 (0.007)	0.004 (0.012)	0.017 (0.012)	0 (0.006)	0 (0)	0.002 (0.02)	0 (0.01)	0 (0.001)	0.032 (0.02)	0 (0.014)	0 (0.022)	0 (0.003)	0 (0.006)	0 (0.001)	0.078 (0.032)	0 (0.009)	0 (0.007)	0.32 (0.035)	0.171 (0.053)	0.257 (0.043)	0.079 (0.021)
ID0050920	O‘AHU	0.029 (0.025)	0.056 (0.024)	0.018 (0.008)	0.021 (0.018)	0.032 (0.019)	0.042 (0.027)	0.053 (0.026)	0.027 (0.044)	0 (0.003)	0 (0.02)	0.01 (0.015)	0.024 (0.027)	0 (0.008)	0.018 (0.021)	0 (0.002)	0 (0)	0.021 (0.026)	0 (0.002)	0.014 (0.021)	0.252 (0.033)	0.002 (0.033)	0.265 (0.04)	0.116 (0.033)
ID0050921	O‘AHU	0 (0.019)	0.038 (0.022)	0.011 (0.006)	0.008 (0.012)	0.019 (0.013)	0.054 (0.03)	0 (0.004)	0.09 (0.062)	0 (0.007)	0 (0.007)	0 (0.004)	0.047 (0.028)	0 (0.018)	0.019 (0.025)	0.013 (0.009)	0 (0.001)	0.046 (0.029)	0 (0.004)	0 (0.009)	0.228 (0.029)	0.167 (0.064)	0.139 (0.05)	0.123 (0.023)
ID0050922	O‘AHU	0.033 (0.026)	0.073 (0.023)	0.021 (0.006)	0 (0.009)	0.029 (0.016)	0.069 (0.03)	0 (0.013)	0.036 (0.041)	0 (0.003)	0 (0.021)	0 (0.004)	0 (0.016)	0.032 (0.027)	0 (0.007)	0.016 (0.007)	0 (0.001)	0.032 (0.023)	0 (0.01)	0 (0.006)	0.257 (0.03)	0.077 (0.057)	0.243 (0.042)	0.082 (0.025)
ID0050923	O‘AHU	0 (0.017)	0.02 (0.017)	0.018 (0.006)	0.013 (0.013)	0.017 (0.014)	0.053 (0.025)	0 (0.001)	0 (0.003)	0 (0.008)	0 (0)	0.029 (0.021)	0 (0.013)	0.002 (0.022)	0 (0)	0 (0.003)	0 (0.002)	0.033 (0.021)	0 (0.003)	0.009 (0.019)	0.277 (0.028)	0.104 (0.049)	0.307 (0.036)	0.118 (0.025)
ID0050924	O‘AHU	0.039 (0.022)	0.014 (0.014)	0.028 (0.008)	0.02 (0.017)	0.009 (0.011)	0.013 (0.019)	0 (0)	0 (0)	0 (0)	0 (0)	0.037 (0.023)	0 (0.005)	0 (0.01)	0 (0.001)	0.001 (0.006)	0 (0.005)	0.019 (0.018)	0 (0.004)	0 (0.012)	0.322 (0.028)	0.086 (0.044)	0.309 (0.034)	0.102 (0.024)
ID0050927	HAWAI‘I	0.027 (0.019)	0 (0.005)	0.011 (0.007)	0.024 (0.018)	0.01 (0.011)	0.015 (0.019)	0.054 (0.025)	0.01 (0.026)	0 (0)	0 (0.006)	0.01 (0.014)	0 (0.007)	0 (0.013)	0 (0.005)	0 (0.003)	0 (0.004)	0.063 (0.03)	0 (0.004)	0.016 (0.026)	0.22 (0.026)	0.121 (0.048)	0.321 (0.036)	0.099 (0.022)
ID0050928	HAWAI‘I	0.016 (0.023)	0 (0.008)	0.007 (0.007)	0.024 (0.017)	0.006 (0.012)	0.039 (0.029)	0.04 (0.025)	0 (0.016)	0 (0.001)	0 (0.012)	0.018 (0.018)	0 (0.001)	0 (0.016)	0.001 (0.013)	0 (0.003)	0.003 (0.009)	0.014 (0.027)	0 (0.001)	0.073 (0.033)	0.205 (0.029)	0.142 (0.048)	0.313 (0.039)	0.097 (0.024)
ID0050929	O‘AHU	0 (0.016)	0.023 (0.019)	0.004 (0.007)	0.029 (0.017)	0 (0.006)	0.04 (0.026)	0 (0.011)	0 (0.011)	0 (0.014)	0 (0.004)	0 (0.004)	0 (0.02)	0 (0.012)	0 (0.002)	0 (0.006)	0 (0.003)	0.094 (0.039)	0 (0.007)	0.014 (0.024)	0.304 (0.037)	0.145 (0.061)	0.255 (0.044)	0.092 (0.023)
ID0050930	HAWAI‘I	0.036 (0.024)	0.001 (0.015)	0.016 (0.007)	0.014 (0.017)	0.004 (0.011)	0 (0.013)	0.042 (0.026)	0 (0.023)	0 (0)	0 (0.007)	0.024 (0.019)	0 (0)	0 (0.016)	0 (0.006)	0 (0.004)	0 (0.006)	0 (0.016)	0 (0.002)	0.091 (0.033)	0.206 (0.031)	0.097 (0.053)	0.354 (0.036)	0.116 (0.024)
ID0062734	HAWAI‘I	0.037 (0.024)	0.009 (0.017)	0 (0.004)	0.044 (0.019)	0 (0.01)	0.035 (0.03)	0.016 (0.017)	0 (0.008)	0 (0.003)	0 (0.014)	0.019 (0.017)	0.039 (0.025)	0 (0.003)	0 (0.005)	0.019 (0.008)	0 (0.001)	0 (0.011)	0 (0)	0 (0.013)	0.266 (0.031)	0.08 (0.049)	0.276 (0.039)	0.159 (0.024)
ID0062740	HAWAI‘I	0.048 (0.023)	0.019 (0.018)	0.009 (0.007)	0.039 (0.017)	0.011 (0.014)	0.006 (0.018)	0.01 (0.014)	0.029 (0.04)	0 (0)	0.028 (0.022)	0.023 (0.017)	0.006 (0.02)	0 (0.008)	0 (0.002)	0.008 (0.007)	0.007 (0.007)	0.023 (0.019)	0 (0.003)	0 (0.016)	0.263 (0.027)	0.074 (0.042)	0.267 (0.031)	0.129 (0.023)
ID0062741	O‘AHU	0.057 (0.025)	0.03 (0.018)	0.015 (0.007)	0.009 (0.014)	0.005 (0.012)	0 (0.013)	0 (0.004)	0.011 (0.031)	0 (0.002)	0 (0)	0 (0.004)	0.039 (0.031)	0 (0.006)	0 (0.005)	0.011 (0.009)	0 (0)	0.077 (0.032)	0 (0)	0 (0.022)	0.251 (0.03)	0.039 (0.037)	0.315 (0.042)	0.14 (0.026)
ID0062743	O‘AHU	0.038 (0.023)	0 (0.01)	0.023 (0.007)	0.011 (0.012)	0 (0.006)	0.006 (0.018)	0 (0)	0 (0)	0 (0)	0 (0.001)	0.015 (0.018)	0 (0.011)	0 (0)	0 (0)	0.008 (0.008)	0 (0.007)	0.045 (0.028)	0 (0)	0 (0.01)	0.269 (0.032)	0.152 (0.046)	0.314 (0.035)	0.118 (0.024)
ID0062744	O‘AHU	0.009 (0.02)	0 (0.01)	0.014 (0.006)	0 (0.007)	0.003 (0.012)	0.028 (0.025)	0 (0)	0 (0.009)	0 (0)	0 (0.001)	0.042 (0.026)	0.061 (0.031)	0 (0)	0 (0)	0 (0.005)	0 (0)	0.012 (0.02)	0 (0.002)	0 (0.002)	0.302 (0.033)	0.159 (0.049)	0.247 (0.04)	0.123 (0.027)
ID0062761	O‘AHU	0.021 (0.019)	0.079 (0.022)	0.017 (0.006)	0 (0.007)	0 (0.011)	0.076 (0.034)	0.042 (0.026)	0.012 (0.045)	0.002 (0.014)	0 (0.003)	0 (0.007)	0 (0.016)	0 (0.012)	0 (0.003)	0 (0)	0 (0)	0.053 (0.025)	0 (0.004)	0 (0.016)	0.25 (0.031)	0.047 (0.042)	0.288 (0.038)	0.111 (0.026)

ID0062797	OʻAHU	0.04 (0.029)	0.059 (0.019)	0.014 (0.006)	0.022 (0.015)	0 (0.008)	0.057 (0.022)	0 (0.006)	0 (0.011)	0.009 (0.015)	0 (0.009)	0 (0.008)	0 (0.008)	0 (0.003)	0 (0)	0 (0.001)	0.001 (0.006)	0.104 (0.028)	0 (0.008)	0 (0.015)	0.259 (0.031)	0.128 (0.05)	0.212 (0.033)	0.095 (0.021)
ID0062815	OʻAHU	0.019 (0.019)	0.054 (0.02)	0.019 (0.006)	0.007 (0.012)	0.014 (0.013)	0.087 (0.033)	0 (0.009)	0 (0.027)	0 (0.003)	0.013 (0.024)	0 (0.012)	0.011 (0.023)	0 (0.003)	0 (0.008)	0 (0.002)	0 (0)	0 (0.012)	0 (0.006)	0.03 (0.025)	0.279 (0.028)	0.168 (0.055)	0.181 (0.047)	0.117 (0.023)
ID0062827	OʻAHU	0 (0.015)	0.033 (0.022)	0.017 (0.007)	0 (0.009)	0 (0.007)	0.115 (0.032)	0.012 (0.018)	0 (0)	0 (0.002)	0 (0.006)	0 (0.009)	0 (0.011)	0 (0.021)	0 (0)	0.001 (0.005)	0 (0)	0 (0.021)	0 (0.006)	0.076 (0.033)	0.272 (0.03)	0.05 (0.047)	0.269 (0.042)	0.155 (0.024)
ID0062829	OʻAHU	0.064 (0.026)	0 (0.003)	0.01 (0.007)	0.016 (0.016)	0.006 (0.01)	0.111 (0.032)	0 (0.001)	0 (0)	0.004 (0.013)	0 (0)	0 (0.01)	0 (0.015)	0 (0.008)	0 (0)	0.006 (0.007)	0.013 (0.011)	0.029 (0.026)	0 (0)	0 (0.009)	0.315 (0.03)	0.105 (0.041)	0.224 (0.036)	0.097 (0.021)
ID0062840	OʻAHU	0.046 (0.027)	0.023 (0.019)	0.008 (0.006)	0 (0.009)	0 (0.01)	0.099 (0.027)	0 (0.009)	0 (0.001)	0 (0.001)	0 (0.003)	0 (0.005)	0.038 (0.027)	0 (0.007)	0 (0.001)	0.008 (0.008)	0 (0.003)	0.038 (0.024)	0 (0.004)	0 (0.01)	0.228 (0.03)	0.153 (0.05)	0.243 (0.042)	0.116 (0.023)
ID0062846	OʻAHU	0.019 (0.019)	0 (0.011)	0.013 (0.005)	0.014 (0.013)	0 (0.006)	0.074 (0.026)	0.034 (0.023)	0 (0.025)	0 (0)	0 (0.003)	0.019 (0.015)	0 (0.01)	0 (0.014)	0.029 (0.019)	0 (0.002)	0 (0.001)	0.004 (0.019)	0 (0.003)	0.06 (0.029)	0.263 (0.027)	0.075 (0.048)	0.268 (0.032)	0.127 (0.024)
ID0062852	OʻAHU	0.056 (0.026)	0.068 (0.02)	0.019 (0.006)	0 (0.009)	0.043 (0.016)	0.071 (0.033)	0.019 (0.02)	0.026 (0.036)	0 (0.004)	0 (0.018)	0 (0.011)	0 (0.021)	0 (0.014)	0.008 (0.014)	0 (0.003)	0 (0.001)	0.004 (0.023)	0 (0.001)	0.065 (0.029)	0.288 (0.028)	0.077 (0.048)	0.178 (0.04)	0.077 (0.026)
ID0062855	OʻAHU	0 (0.007)	0.004 (0.016)	0.004 (0.006)	0.034 (0.018)	0 (0.006)	0.025 (0.024)	0 (0.014)	0 (0.024)	0 (0.008)	0 (0.01)	0 (0.003)	0.035 (0.028)	0.024 (0.026)	0.042 (0.024)	0.003 (0.006)	0.011 (0.01)	0 (0.016)	0 (0.009)	0.02 (0.023)	0.337 (0.028)	0.085 (0.049)	0.198 (0.038)	0.178 (0.022)
ID0062859	OʻAHU	0.065 (0.023)	0.002 (0.012)	0.014 (0.006)	0.013 (0.014)	0.024 (0.016)	0 (0.003)	0.052 (0.023)	0 (0.022)	0 (0)	0 (0.01)	0.002 (0.013)	0 (0)	0 (0.006)	0.003 (0.016)	0 (0.005)	0 (0.003)	0.037 (0.026)	0 (0.007)	0 (0.011)	0.348 (0.031)	0.05 (0.044)	0.322 (0.038)	0.068 (0.027)
ID0062864	OʻAHU	0.103 (0.022)	0.055 (0.021)	0.013 (0.006)	0 (0.008)	0.009 (0.013)	0.018 (0.019)	0 (0.01)	0 (0)	0 (0.005)	0 (0.015)	0 (0.006)	0.003 (0.013)	0 (0.014)	0 (0.003)	0.016 (0.007)	0 (0.002)	0.041 (0.024)	0.001 (0.011)	0 (0.008)	0.314 (0.028)	0.032 (0.037)	0.302 (0.039)	0.092 (0.026)
ID0062873	OʻAHU	0 (0.01)	0.048 (0.02)	0.011 (0.007)	0.018 (0.015)	0.018 (0.014)	0.083 (0.032)	0.028 (0.023)	0 (0.012)	0 (0)	0 (0)	0.012 (0.014)	0 (0.013)	0 (0.003)	0 (0)	0 (0.003)	0 (0.008)	0.084 (0.031)	0 (0.005)	0.017 (0.026)	0.233 (0.026)	0.084 (0.045)	0.202 (0.034)	0.162 (0.024)
ID0062888	OʻAHU	0 (0.011)	0.007 (0.013)	0.009 (0.008)	0.052 (0.021)	0 (0.009)	0.026 (0.021)	0 (0)	0 (0.018)	0 (0)	0 (0.005)	0.023 (0.02)	0 (0.002)	0 (0.011)	0 (0)	0.007 (0.008)	0 (0.002)	0.054 (0.027)	0 (0)	0 (0.007)	0.24 (0.031)	0.167 (0.048)	0.275 (0.04)	0.14 (0.026)
ID0062913	HAWAIʻI	0.06 (0.022)	0.023 (0.018)	0.002 (0.005)	0.048 (0.018)	0.009 (0.012)	0.04 (0.024)	0 (0)	0 (0.009)	0 (0.004)	0 (0.001)	0.015 (0.013)	0 (0.008)	0 (0.014)	0 (0)	0.014 (0.007)	0.009 (0.008)	0.009 (0.014)	0.004 (0.01)	0 (0.006)	0.162 (0.02)	0.227 (0.043)	0.279 (0.038)	0.099 (0.022)
ID0062925	OʻAHU	0.015 (0.026)	0.011 (0.015)	0.011 (0.007)	0.013 (0.013)	0.025 (0.016)	0.125 (0.03)	0 (0.007)	0 (0.004)	0 (0.003)	0 (0)	0.016 (0.019)	0.034 (0.03)	0 (0)	0 (0.001)	0 (0.003)	0 (0.003)	0.028 (0.028)	0 (0.001)	0 (0.008)	0.288 (0.033)	0.089 (0.052)	0.258 (0.041)	0.086 (0.022)
ID0062927	HAWAIʻI	0.099 (0.033)	0.085 (0.027)	0 (0.002)	0 (0.008)	0 (0.001)	0.06 (0.034)	0 (0.012)	0 (0.018)	0 (0.007)	0 (0.008)	0.025 (0.02)	0.009 (0.018)	0 (0.018)	0 (0.001)	0 (0.003)	0 (0.005)	0 (0.012)	0 (0.003)	0.078 (0.029)	0.135 (0.021)	0.108 (0.063)	0.311 (0.048)	0.09 (0.024)
ID0062929	HAWAIʻI	0.048 (0.03)	0.01 (0.019)	0 (0.003)	0.06 (0.021)	0 (0.012)	0.042 (0.028)	0 (0.003)	0 (0.014)	0 (0)	0.029 (0.029)	0.002 (0.015)	0.001 (0.015)	0 (0.002)	0 (0.007)	0.018 (0.009)	0 (0.002)	0 (0.012)	0 (0)	0 (0.016)	0.313 (0.03)	0.138 (0.058)	0.216 (0.047)	0.123 (0.026)
ID0062932	OʻAHU	0.044 (0.022)	0.08 (0.022)	0.016 (0.007)	0.011 (0.013)	0.032 (0.018)	0.035 (0.025)	0.069 (0.031)	0.018 (0.035)	0 (0.011)	0 (0.015)	0 (0.01)	0.045 (0.033)	0 (0.007)	0 (0.007)	0.001 (0.005)	0 (0.002)	0.008 (0.023)	0 (0)	0.025 (0.025)	0.245 (0.031)	0.065 (0.049)	0.243 (0.043)	0.064 (0.026)
ID0062942	OʻAHU	0.07 (0.027)	0.011 (0.013)	0.001 (0.005)	0.044 (0.02)	0.013 (0.013)	0.031 (0.02)	0 (0.006)	0 (0.001)	0 (0.005)	0 (0)	0.03 (0.021)	0 (0.012)	0 (0.001)	0 (0)	0 (0.004)	0.003 (0.008)	0.058 (0.03)	0 (0.002)	0 (0.006)	0.223 (0.031)	0.179 (0.051)	0.234 (0.04)	0.104 (0.026)
ID0062945	OʻAHU	0.064 (0.029)	0.032 (0.019)	0.014 (0.007)	0.014 (0.012)	0 (0.002)	0.074 (0.029)	0 (0.007)	0 (0.003)	0 (0)	0 (0)	0 (0.005)	0.066 (0.029)	0.056 (0.036)	0 (0.008)	0.018 (0.008)	0 (0)	0 (0.016)	0 (0.001)	0 (0.003)	0.232 (0.028)	0 (0.021)	0.28 (0.034)	0.151 (0.023)
ID0062956	OʻAHU	0.081 (0.028)	0.029 (0.02)	0.005 (0.006)	0.03 (0.019)	0.014 (0.013)	0.05 (0.029)	0.033 (0.022)	0 (0.014)	0 (0.005)	0.011 (0.023)	0.001 (0.012)	0.025 (0.026)	0 (0.004)	0 (0.004)	0 (0.005)	0 (0.004)	0.034 (0.027)	0 (0.004)	0 (0.013)	0.266 (0.03)	0.064 (0.048)	0.222 (0.044)	0.132 (0.026)

ID0062970	OʻAHU	0.049 (0.028)	0.021 (0.018)	0.007 (0.006)	0.028 (0.019)	0.008 (0.013)	0.047 (0.027)	0 (0.003)	0 (0.013)	0.041 (0.023)	0 (0.003)	0 (0)	0.011 (0.019)	0 (0.011)	0 (0)	0.01 (0.008)	0 (0)	0.057 (0.031)	0 (0)	0 (0.019)	0.14 (0.028)	0.239 (0.06)	0.248 (0.036)	0.095 (0.022)
ID0062976	OʻAHU	0.051 (0.023)	0.009 (0.013)	0.008 (0.006)	0.043 (0.019)	0.006 (0.012)	0.048 (0.03)	0.018 (0.02)	0 (0.023)	0 (0)	0.014 (0.02)	0.021 (0.019)	0.025 (0.024)	0.011 (0.017)	0 (0.002)	0.019 (0.009)	0 (0)	0.012 (0.02)	0 (0.001)	0 (0.013)	0.258 (0.03)	0 (0.033)	0.309 (0.036)	0.147 (0.023)
ID0062977	OʻAHU	0.005 (0.015)	0.044 (0.018)	0.01 (0.006)	0.002 (0.009)	0 (0.006)	0.073 (0.028)	0.05 (0.024)	0 (0.012)	0 (0.001)	0 (0)	0.015 (0.017)	0.014 (0.022)	0 (0.004)	0.01 (0.015)	0 (0.004)	0 (0.004)	0 (0.01)	0 (0.007)	0.054 (0.025)	0.303 (0.031)	0.031 (0.034)	0.259 (0.035)	0.131 (0.023)
ID0062983	OʻAHU	0.036 (0.025)	0 (0.01)	0.013 (0.008)	0.038 (0.018)	0.018 (0.013)	0.028 (0.021)	0 (0.003)	0 (0.006)	0 (0)	0 (0)	0.011 (0.017)	0.015 (0.023)	0 (0)	0 (0.003)	0 (0.005)	0 (0.001)	0 (0.013)	0.005 (0.009)	0 (0.007)	0.353 (0.033)	0.065 (0.048)	0.336 (0.034)	0.083 (0.022)
ID0062987	OʻAHU	0.024 (0.022)	0.024 (0.016)	0.021 (0.007)	0.029 (0.017)	0 (0.007)	0.045 (0.026)	0 (0.012)	0 (0.007)	0 (0)	0 (0.001)	0.036 (0.022)	0.036 (0.026)	0 (0.003)	0.022 (0.018)	0.01 (0.008)	0 (0.004)	0.034 (0.024)	0 (0.003)	0.005 (0.021)	0.269 (0.028)	0 (0.029)	0.331 (0.033)	0.112 (0.024)
ID0062993	OʻAHU	0.059 (0.029)	0.008 (0.016)	0.004 (0.006)	0.045 (0.02)	0 (0.006)	0.075 (0.029)	0 (0.008)	0 (0)	0 (0.008)	0 (0)	0.009 (0.017)	0 (0)	0 (0.012)	0 (0)	0 (0.002)	0 (0.003)	0.053 (0.024)	0 (0)	0 (0)	0.246 (0.029)	0.117 (0.044)	0.26 (0.046)	0.123 (0.023)
ID0062998	OʻAHU	0.032 (0.025)	0.032 (0.018)	0.01 (0.007)	0.049 (0.017)	0.014 (0.013)	0.058 (0.025)	0 (0.006)	0.005 (0.031)	0 (0.001)	0 (0.006)	0.03 (0.019)	0 (0.004)	0 (0.003)	0 (0.001)	0 (0.002)	0 (0)	0.069 (0.026)	0 (0)	0 (0.002)	0.246 (0.031)	0.069 (0.043)	0.247 (0.036)	0.138 (0.024)
ID0063005	OʻAHU	0.034 (0.025)	0.007 (0.014)	0.012 (0.007)	0.016 (0.014)	0.026 (0.017)	0.033 (0.024)	0 (0.011)	0 (0.011)	0 (0.008)	0.004 (0.016)	0.023 (0.02)	0 (0.009)	0 (0.02)	0 (0.009)	0 (0.003)	0 (0.002)	0.05 (0.025)	0.004 (0.011)	0 (0.018)	0.294 (0.035)	0.189 (0.055)	0.232 (0.043)	0.076 (0.025)
ID0070760	HAWAIʻI	0 (0.006)	0 (0.01)	0.007 (0.007)	0.043 (0.019)	0.011 (0.012)	0 (0.009)	0 (0.005)	0 (0.008)	0 (0.004)	0.001 (0.013)	0.025 (0.019)	0 (0.013)	0.025 (0.027)	0 (0.004)	0.01 (0.008)	0.002 (0.009)	0.042 (0.021)	0 (0.01)	0 (0.015)	0.198 (0.027)	0.164 (0.042)	0.341 (0.037)	0.132 (0.023)
ID0070762	HAWAIʻI	0 (0.004)	0.001 (0.015)	0.018 (0.008)	0.014 (0.016)	0 (0.009)	0.044 (0.031)	0 (0.005)	0 (0.008)	0 (0.004)	0.017 (0.021)	0.019 (0.019)	0.005 (0.017)	0.042 (0.031)	0 (0.001)	0.008 (0.007)	0 (0.003)	0.009 (0.012)	0 (0.011)	0.017 (0.024)	0.224 (0.025)	0.125 (0.057)	0.314 (0.039)	0.142 (0.021)
ID0070763	HAWAIʻI	0 (0.014)	0 (0.004)	0.014 (0.008)	0.026 (0.018)	0.011 (0.014)	0.055 (0.035)	0 (0)	0 (0)	0 (0.001)	0.004 (0.019)	0.002 (0.014)	0 (0)	0 (0.002)	0 (0.005)	0.003 (0.005)	0 (0.007)	0 (0.021)	0 (0)	0.066 (0.035)	0.231 (0.031)	0.146 (0.058)	0.303 (0.043)	0.139 (0.026)
ID0070765	HAWAIʻI	0 (0.002)	0.002 (0.014)	0.002 (0.006)	0.048 (0.021)	0.001 (0.009)	0.046 (0.031)	0 (0)	0 (0.009)	0 (0.009)	0 (0.003)	0.012 (0.017)	0 (0.012)	0 (0.01)	0 (0.01)	0.004 (0.006)	0.008 (0.009)	0.058 (0.031)	0 (0)	0 (0.021)	0.24 (0.035)	0.197 (0.062)	0.282 (0.041)	0.098 (0.023)
ID0070766	HAWAIʻI	0 (0.011)	0.03 (0.019)	0.012 (0.007)	0.024 (0.018)	0.016 (0.015)	0.027 (0.023)	0 (0)	0 (0.004)	0 (0.008)	0 (0.012)	0.007 (0.015)	0 (0.011)	0.057 (0.033)	0 (0.003)	0.004 (0.006)	0 (0.008)	0.03 (0.021)	0 (0.008)	0 (0.016)	0.211 (0.031)	0.13 (0.052)	0.318 (0.037)	0.134 (0.027)
ID0070772	OʻAHU	0 (0.004)	0 (0.01)	0.016 (0.008)	0.015 (0.017)	0 (0.004)	0.046 (0.023)	0 (0.003)	0 (0.021)	0 (0)	0 (0.002)	0.041 (0.023)	0 (0.004)	0 (0.014)	0 (0.012)	0.003 (0.006)	0 (0.004)	0.033 (0.024)	0 (0.005)	0.009 (0.018)	0.306 (0.029)	0.145 (0.047)	0.258 (0.038)	0.127 (0.02)
ID0070773	OʻAHU	0.006 (0.016)	0.005 (0.011)	0.013 (0.007)	0.044 (0.021)	0 (0.003)	0.042 (0.026)	0.025 (0.021)	0 (0.005)	0 (0)	0 (0)	0.044 (0.022)	0 (0.019)	0 (0.001)	0 (0.004)	0.002 (0.005)	0 (0.001)	0.023 (0.024)	0 (0.003)	0.029 (0.025)	0.313 (0.027)	0.033 (0.038)	0.294 (0.033)	0.128 (0.022)
ID0070776	OʻAHU	0.036 (0.021)	0 (0.008)	0.025 (0.008)	0.004 (0.011)	0.024 (0.016)	0.05 (0.025)	0.015 (0.019)	0.011 (0.028)	0 (0)	0 (0.007)	0.034 (0.019)	0 (0.014)	0 (0.012)	0.003 (0.011)	0.007 (0.006)	0 (0.003)	0.031 (0.022)	0 (0)	0.006 (0.02)	0.313 (0.027)	0.019 (0.029)	0.323 (0.037)	0.097 (0.026)
ID0070777	OʻAHU	0.031 (0.026)	0.06 (0.019)	0.012 (0.006)	0.001 (0.008)	0.008 (0.014)	0.074 (0.027)	0 (0.009)	0 (0)	0 (0.009)	0 (0.003)	0.024 (0.018)	0.08 (0.034)	0 (0.009)	0 (0.001)	0.002 (0.006)	0 (0.006)	0.022 (0.023)	0 (0.006)	0 (0.016)	0.233 (0.033)	0.075 (0.04)	0.26 (0.037)	0.118 (0.029)
ID0070778	OʻAHU	0.05 (0.024)	0.023 (0.017)	0.014 (0.006)	0.027 (0.017)	0.001 (0.009)	0.035 (0.021)	0 (0.005)	0.017 (0.029)	0 (0)	0 (0.005)	0.001 (0.013)	0.007 (0.016)	0 (0.001)	0 (0.005)	0 (0.004)	0 (0.001)	0.017 (0.021)	0 (0.004)	0 (0.01)	0.338 (0.027)	0.049 (0.045)	0.301 (0.037)	0.121 (0.025)
ID0070782	OʻAHU	0.031 (0.023)	0.013 (0.016)	0.011 (0.007)	0.024 (0.018)	0.005 (0.013)	0.034 (0.024)	0 (0.013)	0 (0.006)	0 (0.007)	0 (0)	0.026 (0.02)	0 (0.008)	0 (0.007)	0 (0.002)	0.007 (0.007)	0.008 (0.009)	0.012 (0.024)	0 (0.007)	0.035 (0.028)	0.287 (0.031)	0.127 (0.047)	0.275 (0.035)	0.104 (0.022)
ID0070787	OʻAHU	0.043 (0.027)	0.006 (0.015)	0.003 (0.006)	0.024 (0.018)	0.016 (0.015)	0.064 (0.031)	0 (0.004)	0 (0.005)	0 (0.005)	0 (0.001)	0.048 (0.023)	0 (0.004)	0 (0.001)	0 (0.002)	0 (0.001)	0 (0)	0.053 (0.036)	0 (0)	0.07 (0.033)	0.197 (0.034)	0.124 (0.049)	0.226 (0.041)	0.125 (0.024)

ID0070799	OʻAHU	0.059 (0.024)	0.032 (0.019)	0.016 (0.007)	0.005 (0.012)	0.017 (0.013)	0.102 (0.027)	0 (0.011)	0 (0.009)	0 (0)	0 (0)	0.011 (0.015)	0.022 (0.023)	0 (0.006)	0 (0.001)	0 (0.003)	0 (0.002)	0.043 (0.032)	0 (0.002)	0.025 (0.026)	0.232 (0.029)	0.173 (0.05)	0.201 (0.036)	0.061 (0.028)
ID0070860	HAWAIʻI	0.004 (0.022)	0.029 (0.019)	0.003 (0.005)	0.023 (0.015)	0 (0.007)	0.068 (0.031)	0 (0.005)	0 (0.029)	0 (0.001)	0.011 (0.018)	0.005 (0.012)	0 (0)	0 (0.001)	0 (0.007)	0.015 (0.009)	0 (0.004)	0.088 (0.029)	0 (0.004)	0 (0.016)	0.163 (0.031)	0.164 (0.062)	0.348 (0.049)	0.077 (0.025)
ID0070861	HAWAIʻI	0.02 (0.022)	0.025 (0.021)	0.007 (0.006)	0.023 (0.016)	0 (0.005)	0.039 (0.023)	0 (0.003)	0 (0.026)	0 (0)	0.012 (0.022)	0.008 (0.014)	0 (0.01)	0 (0.012)	0 (0.007)	0.033 (0.007)	0 (0)	0.027 (0.021)	0 (0.005)	0 (0.009)	0.164 (0.028)	0.269 (0.046)	0.268 (0.041)	0.105 (0.02)
NKL894	HAWAIʻI	0.042 (0.024)	0.02 (0.021)	0.001 (0.004)	0.038 (0.019)	0.006 (0.011)	0.007 (0.018)	0 (0.009)	0.031 (0.034)	0 (0)	0.056 (0.031)	0.032 (0.019)	0 (0.009)	0 (0)	0 (0.004)	0.004 (0.007)	0 (0.004)	0.065 (0.024)	0 (0.003)	0 (0.01)	0.14 (0.029)	0.1 (0.052)	0.315 (0.048)	0.143 (0.026)
NKL895	HAWAIʻI	0.038 (0.023)	0.021 (0.018)	0.006 (0.006)	0.01 (0.01)	0.014 (0.014)	0.02 (0.019)	0.007 (0.019)	0.005 (0.032)	0 (0)	0.031 (0.026)	0 (0.01)	0 (0.014)	0 (0)	0 (0.001)	0.002 (0.007)	0.004 (0.009)	0.088 (0.027)	0 (0.001)	0 (0.01)	0.17 (0.031)	0.115 (0.052)	0.365 (0.047)	0.106 (0.025)
NKL896	HAWAIʻI	0 (0.016)	0.015 (0.016)	0.017 (0.006)	0.009 (0.012)	0.013 (0.012)	0.025 (0.023)	0 (0)	0 (0.003)	0 (0.008)	0 (0.013)	0.012 (0.015)	0.013 (0.018)	0 (0.015)	0 (0.001)	0.008 (0.008)	0 (0.003)	0.078 (0.032)	0 (0.004)	0 (0.013)	0.192 (0.026)	0.163 (0.05)	0.309 (0.037)	0.145 (0.026)
NKL897	HAWAIʻI	0.074 (0.029)	0.028 (0.02)	0.006 (0.005)	0.01 (0.011)	0.007 (0.015)	0.073 (0.031)	0 (0.012)	0.017 (0.037)	0 (0.002)	0.039 (0.029)	0 (0.004)	0 (0.004)	0 (0.013)	0 (0)	0.006 (0.008)	0 (0.005)	0.032 (0.026)	0 (0)	0 (0.01)	0.188 (0.03)	0.228 (0.059)	0.183 (0.042)	0.108 (0.025)
NKL898	HAWAIʻI	0.05 (0.025)	0.02 (0.016)	0.013 (0.007)	0.015 (0.014)	0.022 (0.013)	0.064 (0.03)	0 (0.006)	0.038 (0.035)	0 (0)	0 (0.005)	0.008 (0.014)	0.023 (0.025)	0 (0.006)	0 (0.004)	0.017 (0.008)	0 (0.003)	0.017 (0.021)	0 (0.001)	0.029 (0.026)	0.155 (0.027)	0.134 (0.052)	0.316 (0.039)	0.078 (0.025)
NKL899	HAWAIʻI	0.07 (0.025)	0 (0.012)	0.004 (0.005)	0.019 (0.015)	0 (0.008)	0.06 (0.029)	0 (0.008)	0 (0.028)	0 (0.01)	0 (0.009)	0 (0.008)	0 (0.008)	0 (0.009)	0 (0.002)	0 (0.003)	0 (0)	0 (0.01)	0 (0.001)	0.101 (0.027)	0.197 (0.026)	0.172 (0.064)	0.249 (0.04)	0.128 (0.026)
NKL900	HAWAIʻI	0.039 (0.021)	0.029 (0.017)	0.004 (0.005)	0.04 (0.023)	0 (0.01)	0.057 (0.028)	0.015 (0.018)	0 (0.003)	0 (0.003)	0 (0.004)	0.006 (0.015)	0.035 (0.026)	0 (0.008)	0 (0)	0.007 (0.008)	0 (0.006)	0.034 (0.024)	0 (0.005)	0 (0.011)	0.202 (0.027)	0.032 (0.043)	0.366 (0.04)	0.132 (0.027)
NKL901	HAWAIʻI	0.114 (0.029)	0.017 (0.019)	0.006 (0.006)	0.028 (0.016)	0.006 (0.01)	0.027 (0.022)	0.045 (0.027)	0 (0.007)	0 (0)	0.002 (0.024)	0.042 (0.023)	0 (0.014)	0 (0.002)	0 (0.001)	0.016 (0.008)	0 (0)	0 (0.005)	0 (0)	0.045 (0.023)	0.186 (0.029)	0.121 (0.051)	0.278 (0.036)	0.067 (0.025)
NKL902	HAWAIʻI	0.012 (0.017)	0 (0.008)	0.011 (0.007)	0.051 (0.018)	0.013 (0.014)	0.043 (0.027)	0 (0.001)	0.003 (0.037)	0 (0)	0 (0.002)	0.058 (0.022)	0 (0.006)	0 (0.006)	0 (0.005)	0.007 (0.006)	0.001 (0.006)	0.006 (0.017)	0 (0.004)	0.005 (0.017)	0.204 (0.027)	0.102 (0.042)	0.384 (0.037)	0.099 (0.022)
NKL903	HAWAIʻI	0.056 (0.023)	0.053 (0.022)	0.004 (0.006)	0.029 (0.017)	0.006 (0.012)	0.07 (0.036)	0 (0.007)	0 (0.018)	0 (0.009)	0 (0)	0 (0.006)	0.023 (0.029)	0.01 (0.023)	0 (0.008)	0.016 (0.009)	0 (0.004)	0.031 (0.03)	0 (0)	0.02 (0.024)	0.182 (0.034)	0.115 (0.055)	0.295 (0.042)	0.091 (0.021)
NKL904	HAWAIʻI	0.064 (0.029)	0.04 (0.021)	0 (0.002)	0.017 (0.014)	0 (0.007)	0.047 (0.027)	0 (0.007)	0.034 (0.038)	0 (0.003)	0.033 (0.029)	0.034 (0.019)	0 (0.002)	0 (0.011)	0 (0.008)	0 (0.001)	0 (0.003)	0.072 (0.033)	0 (0.005)	0.021 (0.026)	0.12 (0.032)	0.283 (0.053)	0.192 (0.042)	0.044 (0.022)
NKL905	HAWAIʻI	0 (0.006)	0.019 (0.017)	0.012 (0.007)	0.038 (0.019)	0.039 (0.016)	0 (0.014)	0 (0.003)	0 (0)	0 (0.005)	0 (0)	0.025 (0.018)	0 (0.008)	0 (0.008)	0 (0)	0.01 (0.007)	0 (0.003)	0 (0.013)	0 (0.001)	0.08 (0.024)	0.153 (0.024)	0.17 (0.047)	0.307 (0.042)	0.147 (0.025)
NKL906	HAWAIʻI	0.015 (0.018)	0 (0.009)	0.007 (0.006)	0.042 (0.021)	0 (0.005)	0.032 (0.025)	0 (0.012)	0 (0.008)	0 (0.002)	0 (0)	0 (0.008)	0 (0.015)	0 (0.001)	0 (0)	0.007 (0.007)	0 (0.002)	0 (0.018)	0 (0)	0.107 (0.03)	0.128 (0.024)	0.114 (0.052)	0.392 (0.038)	0.156 (0.023)
NKL907	HAWAIʻI	0.095 (0.029)	0 (0.011)	0.006 (0.006)	0.045 (0.017)	0.028 (0.013)	0.011 (0.02)	0 (0.01)	0.077 (0.051)	0 (0)	0.025 (0.022)	0.015 (0.017)	0 (0.012)	0 (0.007)	0 (0.003)	0 (0.005)	0 (0.005)	0.026 (0.023)	0 (0.006)	0.018 (0.023)	0.149 (0.028)	0.185 (0.058)	0.238 (0.037)	0.084 (0.022)
NKL908	HAWAIʻI	0.097 (0.027)	0.027 (0.022)	0.002 (0.005)	0.031 (0.017)	0 (0.008)	0.025 (0.023)	0.054 (0.03)	0 (0.016)	0 (0)	0.021 (0.027)	0.05 (0.022)	0 (0.006)	0.001 (0.021)	0 (0)	0 (0.003)	0.006 (0.008)	0.009 (0.026)	0 (0)	0.087 (0.036)	0.142 (0.027)	0.116 (0.058)	0.215 (0.037)	0.116 (0.025)
NKL909	HAWAIʻI	0.125 (0.031)	0.06 (0.022)	0 (0.003)	0.034 (0.015)	0 (0.005)	0.054 (0.028)	0.025 (0.019)	0 (0.023)	0 (0.01)	0 (0.005)	0 (0.005)	0.065 (0.029)	0 (0.015)	0 (0.002)	0.003 (0.006)	0 (0.004)	0 (0.019)	0 (0.011)	0.042 (0.024)	0.127 (0.027)	0.15 (0.056)	0.233 (0.036)	0.081 (0.021)
NKL910	HAWAIʻI	0.135 (0.025)	0.056 (0.022)	0 (0.003)	0.026 (0.013)	0.021 (0.013)	0.046 (0.029)	0.013 (0.024)	0.054 (0.039)	0 (0.008)	0 (0.017)	0.003 (0.011)	0.024 (0.021)	0 (0.007)	0 (0.002)	0 (0.003)	0.008 (0.009)	0.027 (0.021)	0 (0.006)	0 (0.004)	0.179 (0.026)	0.152 (0.062)	0.217 (0.043)	0.039 (0.021)

NKL911	HAWAI‘I	0.09 (0.029)	0.031 (0.02)	0.019 (0.006)	0 (0.005)	0.029 (0.015)	0 (0.013)	0 (0)	0 (0.008)	0 (0.015)	0 (0)	0.013 (0.014)	0 (0.007)	0.018 (0.026)	0.003 (0.012)	0.008 (0.008)	0 (0)	0 (0.003)	0 (0)	0.047 (0.031)	0.238 (0.03)	0.114 (0.058)	0.284 (0.04)	0.107 (0.027)
NKL912	HAWAI‘I	0.07 (0.022)	0.045 (0.023)	0.011 (0.007)	0.036 (0.019)	0.004 (0.012)	0.021 (0.024)	0 (0)	0 (0.02)	0 (0)	0.025 (0.026)	0.003 (0.012)	0.007 (0.018)	0 (0.014)	0 (0)	0 (0.004)	0 (0.002)	0.063 (0.03)	0.01 (0.014)	0.002 (0.021)	0.157 (0.027)	0.076 (0.054)	0.338 (0.046)	0.132 (0.024)
NKL913	HAWAI‘I	0.019 (0.017)	0.032 (0.02)	0.009 (0.007)	0.026 (0.015)	0.008 (0.014)	0.056 (0.029)	0 (0)	0 (0.011)	0 (0)	0 (0.002)	0.011 (0.014)	0.026 (0.022)	0.01 (0.023)	0 (0.006)	0.007 (0.006)	0 (0.004)	0.043 (0.027)	0 (0.001)	0.01 (0.021)	0.163 (0.026)	0.009 (0.032)	0.394 (0.033)	0.176 (0.023)
NKL914	HAWAI‘I	0.024 (0.021)	0.012 (0.016)	0.018 (0.006)	0 (0.006)	0 (0.006)	0.058 (0.029)	0 (0.006)	0 (0.017)	0 (0)	0 (0.013)	0.038 (0.021)	0.013 (0.025)	0 (0.005)	0 (0.006)	0.005 (0.006)	0 (0.004)	0.064 (0.038)	0 (0.01)	0.043 (0.035)	0.163 (0.026)	0.03 (0.039)	0.375 (0.033)	0.156 (0.022)
NKL915	HAWAI‘I	0 (0.014)	0.02 (0.02)	0.009 (0.006)	0.027 (0.016)	0 (0.004)	0.062 (0.032)	0 (0.003)	0.031 (0.039)	0 (0.001)	0 (0.012)	0.025 (0.019)	0 (0.006)	0 (0.007)	0 (0.012)	0.014 (0.009)	0.004 (0.007)	0.038 (0.023)	0.009 (0.012)	0 (0.019)	0.183 (0.029)	0.103 (0.049)	0.349 (0.04)	0.125 (0.025)
NKL916	HAWAI‘I	0.009 (0.017)	0.026 (0.022)	0.015 (0.006)	0.004 (0.01)	0 (0.009)	0.056 (0.031)	0 (0)	0 (0.026)	0 (0.01)	0.03 (0.024)	0 (0.009)	0.002 (0.017)	0 (0.004)	0 (0.004)	0.007 (0.007)	0.007 (0.009)	0.033 (0.021)	0.006 (0.011)	0 (0.012)	0.226 (0.026)	0.113 (0.056)	0.344 (0.032)	0.122 (0.02)
NKL917	HAWAI‘I	0.096 (0.027)	0.017 (0.017)	0.004 (0.006)	0.025 (0.014)	0.009 (0.013)	0.047 (0.028)	0 (0.004)	0 (0.009)	0 (0.002)	0 (0.006)	0.009 (0.014)	0 (0.001)	0 (0.015)	0 (0.006)	0 (0.003)	0 (0.002)	0.036 (0.02)	0 (0.004)	0 (0.013)	0.191 (0.021)	0.145 (0.055)	0.308 (0.037)	0.113 (0.026)
NKL918	HAWAI‘I	0.13 (0.027)	0.054 (0.025)	0.001 (0.004)	0.049 (0.02)	0 (0.007)	0.025 (0.025)	0 (0.005)	0 (0.01)	0 (0.001)	0 (0.002)	0 (0.005)	0 (0.006)	0 (0.012)	0 (0.002)	0.008 (0.006)	0.02 (0.01)	0.002 (0.017)	0 (0.004)	0 (0.014)	0.154 (0.025)	0.198 (0.055)	0.242 (0.037)	0.116 (0.027)
NKL919	HAWAI‘I	0.124 (0.03)	0.023 (0.018)	0 (0.002)	0.025 (0.017)	0.016 (0.015)	0.056 (0.036)	0.022 (0.026)	0.06 (0.052)	0 (0.004)	0 (0.004)	0 (0.007)	0 (0.004)	0 (0.011)	0.004 (0.018)	0.009 (0.008)	0 (0.001)	0.031 (0.029)	0 (0.001)	0.069 (0.037)	0.184 (0.027)	0.077 (0.065)	0.253 (0.038)	0.046 (0.023)
NKL920	HAWAI‘I	0.038 (0.024)	0.051 (0.023)	0.019 (0.007)	0.004 (0.012)	0.026 (0.016)	0.051 (0.024)	0 (0.008)	0 (0.032)	0 (0.007)	0 (0.004)	0.037 (0.02)	0.038 (0.027)	0 (0.001)	0 (0)	0.011 (0.008)	0 (0.005)	0.053 (0.03)	0 (0.006)	0 (0.005)	0.137 (0.034)	0.141 (0.058)	0.283 (0.039)	0.112 (0.023)
NKL921	HAWAI‘I	0.151 (0.029)	0.062 (0.02)	0.005 (0.005)	0.025 (0.017)	0 (0.004)	0.043 (0.024)	0 (0.008)	0.043 (0.043)	0 (0.004)	0.01 (0.022)	0 (0.009)	0 (0.003)	0 (0)	0 (0.001)	0.001 (0.003)	0 (0.003)	0 (0.016)	0 (0.001)	0.04 (0.023)	0.179 (0.022)	0.14 (0.059)	0.224 (0.037)	0.076 (0.023)
NKL922	HAWAI‘I	0.006 (0.018)	0.031 (0.019)	0.01 (0.006)	0.014 (0.012)	0.009 (0.012)	0.067 (0.033)	0 (0.003)	0 (0)	0.01 (0.018)	0 (0.011)	0 (0.009)	0 (0.015)	0 (0.014)	0 (0)	0.001 (0.005)	0.006 (0.007)	0.069 (0.028)	0 (0.002)	0 (0.012)	0.177 (0.028)	0.213 (0.058)	0.274 (0.04)	0.113 (0.022)
NKL923	HAWAI‘I	0.115 (0.025)	0 (0.008)	0.011 (0.005)	0 (0.005)	0.042 (0.013)	0 (0.001)	0.017 (0.02)	0 (0.008)	0.034 (0.026)	0 (0.011)	0 (0.009)	0 (0.018)	0 (0.002)	0 (0.001)	0.005 (0.006)	0 (0.005)	0 (0.014)	0 (0)	0.044 (0.023)	0.206 (0.023)	0.198 (0.048)	0.241 (0.04)	0.088 (0.021)
NKL924	HAWAI‘I	0.095 (0.021)	0 (0.009)	0.012 (0.007)	0.012 (0.012)	0.014 (0.013)	0 (0.001)	0.021 (0.021)	0 (0.012)	0.019 (0.023)	0.019 (0.021)	0.017 (0.018)	0 (0.014)	0 (0.015)	0 (0)	0.005 (0.006)	0 (0.004)	0 (0.014)	0 (0)	0.035 (0.022)	0.176 (0.025)	0.272 (0.052)	0.235 (0.038)	0.069 (0.022)

Table S2. Asian Reference Cluster Delineation. We closely examined reference clusters from Smyser et al. 2020 and Ai et al. 2013 that were comprised of Meishan and related breeds and Asian wild boar populations to help clarify ancestral signals from Asia (*n* = 626). We combined genetically similar reference groups into reference clusters based on their spatial origin and an unsupervised ADMIXTURE clustering test. We identified *K* = 5 as the highest subdivision of Asian breeds feasible to delineate potentially meaningful reference groups. In the absence of a true Polynesian pig to include in our reference set, we categorized the samples from southeast Asia (i.e., southeast China and Thailand) as the most likely reference of Polynesian pigs (Allen et al. 2001, Horsburgh et al. 2022) and refer to this group as the "Southeast Asian" cluster (K_{20}). The four remaining subclusters represent samples from across eastern China ("Jinhua" (K_{16}) and "Eastern China" (K_{18}) clusters), western China ("Western China" (K_{19}) cluster), and "Other Asian clusters" (K_{17}; with representative breeds sampled across western, central, and northern China, South Korea, and Russia).

Reference Cluster Name	Sample ID	Population Name	Population Abbreviation	Origin	Latitude	Longitude	Reference Paper
Jinhua	16-8-CNJH01U11	Jinhua	CNJH	Jinhua	29.1	119.7	Yang et al. 2017
Jinhua	16-CNJH-CNJH_10_J	Jinhua	CNJH	Jinhua	29.1	119.7	Yang et al. 2017
Jinhua	16-CNJH-CNJH_11_J	Jinhua	CNJH	Jinhua	29.1	119.7	Yang et al. 2017
Jinhua	16-CNJH-CNJH_12_J	Jinhua	CNJH	Jinhua	29.1	119.7	Yang et al. 2017
Jinhua	16-CNJH-CNJH_14_W	Jinhua	CNJH	Jinhua	29.1	119.7	Yang et al. 2017
Jinhua	16-CNJH-CNJH_15_W	Jinhua	CNJH	Jinhua	29.1	119.7	Yang et al. 2017
Jinhua	16-CNJH-CNJH_16_W	Jinhua	CNJH	Jinhua	29.1	119.7	Yang et al. 2017
Jinhua	16-CNJH-CNJH_18_W	Jinhua	CNJH	Jinhua	29.1	119.7	Yang et al. 2017
Jinhua	16-CNJH-CNJH_19_W	Jinhua	CNJH	Jinhua	29.1	119.7	Yang et al. 2017
Jinhua	16-CNJH-CNJH_20_W	Jinhua	CNJH	Jinhua	29.1	119.7	Yang et al. 2017
Jinhua	16-CNJH-CNJH_21_W	Jinhua	CNJH	Jinhua	29.1	119.7	Yang et al. 2017
Jinhua	16-CNJH-CNJH_22_W	Jinhua	CNJH	Jinhua	29.1	119.7	Yang et al. 2017
Jinhua	16-CNJH-CNJH_23_W	Jinhua	CNJH	Jinhua	29.1	119.7	Yang et al. 2017
Jinhua	16-CNJH-CNJH_25_W	Jinhua	CNJH	Jinhua	29.1	119.7	Yang et al. 2017
Jinhua	16-CNJH-CNJH_26_W	Jinhua	CNJH	Jinhua	29.1	119.7	Yang et al. 2017
Jinhua	16-CNJH-CNJH_27_W	Jinhua	CNJH	Jinhua	29.1	119.7	Yang et al. 2017
Jinhua	16-CNJH-CNJH_28_W	Jinhua	CNJH	Jinhua	29.1	119.7	Yang et al. 2017
Jinhua	16-CNJH-CNJH_29_W	Jinhua	CNJH	Jinhua	29.1	119.7	Yang et al. 2017
Jinhua	16-CNJH-CNJH_30_W	Jinhua	CNJH	Jinhua	29.1	119.7	Yang et al. 2017

Jinhua	16-CNJH-CNJH_8_J	Jinhua	CNJH	Jinhua	29.1	119.7	Yang et al. 2017
Jinhua	16-CNJH-CNJH_9_J	Jinhua	CNJH	Jinhua	29.1	119.7	Yang et al. 2017
Jinhua	JH-JH0116	Jinhua	JH	Zhejiang			Ai et al 2013
Jinhua	JH-JH0122	Jinhua	JH	Zhejiang			Ai et al 2013
Jinhua	JH-JH0124	Jinhua	JH	Zhejiang			Ai et al 2013
Jinhua	JH-JH0128	Jinhua	JH	Zhejiang			Ai et al 2013
Jinhua	JH-JH0138	Jinhua	JH	Zhejiang			Ai et al 2013
Jinhua	JH-JH0144	Jinhua	JH	Zhejiang			Ai et al 2013
Jinhua	JH-JH0164	Jinhua	JH	Zhejiang			Ai et al 2013
Jinhua	JH-JH0209	Jinhua	JH	Zhejiang			Ai et al 2013
Other Asian	16-CNDQ-CNDQ_1_J	Diqing Zang	CNDQ	Yunnan	27.8	99.7	Yang et al. 2017
Other Asian	16-CNDQ-CNDQ_10_J	Diqing Zang	CNDQ	Yunnan	27.8	99.7	Yang et al. 2017
Other Asian	16-CNDQ-CNDQ_11_J	Diqing Zang	CNDQ	Yunnan	27.8	99.7	Yang et al. 2017
Other Asian	16-CNDQ-CNDQ_12_J	Diqing Zang	CNDQ	Yunnan	27.8	99.7	Yang et al. 2017
Other Asian	16-CNDQ-CNDQ_13_J	Diqing Zang	CNDQ	Yunnan	27.8	99.7	Yang et al. 2017
Other Asian	16-CNDQ-CNDQ_14_J	Diqing Zang	CNDQ	Yunnan	27.8	99.7	Yang et al. 2017
Other Asian	16-CNDQ-CNDQ_15_J	Diqing Zang	CNDQ	Yunnan	27.8	99.7	Yang et al. 2017
Other Asian	16-CNDQ-CNDQ_16_J	Diqing Zang	CNDQ	Yunnan	27.8	99.7	Yang et al. 2017
Other Asian	16-CNDQ-CNDQ_17_J	Diqing Zang	CNDQ	Yunnan	27.8	99.7	Yang et al. 2017
Other Asian	16-CNDQ-CNDQ_18_J	Diqing Zang	CNDQ	Yunnan	27.8	99.7	Yang et al. 2017
Other Asian	16-CNDQ-CNDQ_19_J	Diqing Zang	CNDQ	Yunnan	27.8	99.7	Yang et al. 2017
Other Asian	16-CNDQ-CNDQ_2_J	Diqing Zang	CNDQ	Yunnan	27.8	99.7	Yang et al. 2017
Other Asian	16-CNDQ-CNDQ_3_J	Diqing Zang	CNDQ	Yunnan	27.8	99.7	Yang et al. 2017
Other Asian	16-CNDQ-CNDQ_4_J	Diqing Zang	CNDQ	Yunnan	27.8	99.7	Yang et al. 2017
Other Asian	16-CNDQ-CNDQ_7_J	Diqing Zang	CNDQ	Yunnan	27.8	99.7	Yang et al. 2017
Other Asian	16-CNDQ-CNDQ_8_J	Diqing Zang	CNDQ	Yunnan	27.8	99.7	Yang et al. 2017
Other Asian	16-CNDQ-CNDQ_9_J	Diqing Zang	CNDQ	Yunnan	27.8	99.7	Yang et al. 2017
Other Asian	16-CNGS-CNGS_1_J	Gansu Zang	CNGS	Gansu	35	103.1	Yang et al. 2017
Other Asian	16-CNGS-CNGS_10_J	Gansu Zang	CNGS	Gansu	35	103.1	Yang et al. 2017
Other Asian	16-CNGS-CNGS_11_J	Gansu Zang	CNGS	Gansu	35	103.1	Yang et al. 2017

Other Asian	16-CNGS-CNGS_12_J	Gansu Zang	CNGS	Gansu	35	103.1	Yang et al. 2017
Other Asian	16-CNGS-CNGS_13_J	Gansu Zang	CNGS	Gansu	35	103.1	Yang et al. 2017
Other Asian	16-CNGS-CNGS_14_J	Gansu Zang	CNGS	Gansu	35	103.1	Yang et al. 2017
Other Asian	16-CNGS-CNGS_15_J	Gansu Zang	CNGS	Gansu	35	103.1	Yang et al. 2017
Other Asian	16-CNGS-CNGS_16_J	Gansu Zang	CNGS	Gansu	35	103.1	Yang et al. 2017
Other Asian	16-CNGS-CNGS_17_J	Gansu Zang	CNGS	Gansu	35	103.1	Yang et al. 2017
Other Asian	16-CNGS-CNGS_18_J	Gansu Zang	CNGS	Gansu	35	103.1	Yang et al. 2017
Other Asian	16-CNGS-CNGS_19_J	Gansu Zang	CNGS	Gansu	35	103.1	Yang et al. 2017
Other Asian	16-CNGS-CNGS_20_J	Gansu Zang	CNGS	Gansu	35	103.1	Yang et al. 2017
Other Asian	16-CNGS-CNGS_21_J	Gansu Zang	CNGS	Gansu	35	103.1	Yang et al. 2017
Other Asian	16-CNGS-CNGS_5_J	Gansu Zang	CNGS	Gansu	35	103.1	Yang et al. 2017
Other Asian	16-CNGS-CNGS_6_J	Gansu Zang	CNGS	Gansu	35	103.1	Yang et al. 2017
Other Asian	16-CNGS-CNGS_7_J	Gansu Zang	CNGS	Gansu	35	103.1	Yang et al. 2017
Other Asian	16-CNGS-CNGS_8_J	Gansu Zang	CNGS	Gansu	35	103.1	Yang et al. 2017
Other Asian	16-CNGS-CNGS_9_J	Gansu Zang	CNGS	Gansu	35	103.1	Yang et al. 2017
Other Asian	16-CNGX-CNGX_1_J	Ganxiliangtouwu	CNGX	Jiangxi	28.2	114.9	Yang et al. 2017
Other Asian	16-CNGX-CNGX_10_J	Ganxiliangtouwu	CNGX	Jiangxi	28.2	114.9	Yang et al. 2017
Other Asian	16-CNGX-CNGX_11_J	Ganxiliangtouwu	CNGX	Jiangxi	28.2	114.9	Yang et al. 2017
Other Asian	16-CNGX-CNGX_12_J	Ganxiliangtouwu	CNGX	Jiangxi	28.2	114.9	Yang et al. 2017
Other Asian	16-CNGX-CNGX_13_J	Ganxiliangtouwu	CNGX	Jiangxi	28.2	114.9	Yang et al. 2017
Other Asian	16-CNGX-CNGX_2_J	Ganxiliangtouwu	CNGX	Jiangxi	28.2	114.9	Yang et al. 2017
Other Asian	16-CNGX-CNGX_3_J	Ganxiliangtouwu	CNGX	Jiangxi	28.2	114.9	Yang et al. 2017
Other Asian	16-CNGX-CNGX_4_J	Ganxiliangtouwu	CNGX	Jiangxi	28.2	114.9	Yang et al. 2017
Other Asian	16-CNGX-CNGX_5_J	Ganxiliangtouwu	CNGX	Jiangxi	28.2	114.9	Yang et al. 2017
Other Asian	16-CNGX-CNGX_6_J	Ganxiliangtouwu	CNGX	Jiangxi	28.2	114.9	Yang et al. 2017
Other Asian	16-CNGX-CNGX_7_J	Ganxiliangtouwu	CNGX	Jiangxi	28.2	114.9	Yang et al. 2017
Other Asian	16-CNGX-CNGX_8_J	Ganxiliangtouwu	CNGX	Jiangxi	28.2	114.9	Yang et al. 2017
Other Asian	16-CNGX-CNGX_9_J	Ganxiliangtouwu	CNGX	Jiangxi	28.2	114.9	Yang et al. 2017
Other Asian	16-CNXZ-CNXZ_1_J	Gongbujiangda Zang	CNXZ	Tibet	30	93.2	Yang et al. 2017
Other Asian	16-CNXZ-CNXZ_12_J	Gongbujiangda Zang	CNXZ	Tibet	30	93.2	Yang et al. 2017

Other Asian	16-CNXZ-CNXZ_13_J	Gongbujiangda Zang	CNXZ	Tibet	30	93.2	Yang et al. 2017
Other Asian	16-CNXZ-CNXZ_14_J	Gongbujiangda Zang	CNXZ	Tibet	30	93.2	Yang et al. 2017
Other Asian	16-CNXZ-CNXZ_17_J	Gongbujiangda Zang	CNXZ	Tibet	30	93.2	Yang et al. 2017
Other Asian	16-CNXZ-CNXZ_18_J	Gongbujiangda Zang	CNXZ	Tibet	30	93.2	Yang et al. 2017
Other Asian	16-CNXZ-CNXZ_2_J	Gongbujiangda Zang	CNXZ	Tibet	30	93.2	Yang et al. 2017
Other Asian	16-CNXZ-CNXZ_21_J	Gongbujiangda Zang	CNXZ	Tibet	30	93.2	Yang et al. 2017
Other Asian	16-CNXZ-CNXZ_22_J	Gongbujiangda Zang	CNXZ	Tibet	30	93.2	Yang et al. 2017
Other Asian	16-CNXZ-CNXZ_23_J	Gongbujiangda Zang	CNXZ	Tibet	30	93.2	Yang et al. 2017
Other Asian	16-CNXZ-CNXZ_24_J	Gongbujiangda Zang	CNXZ	Tibet	30	93.2	Yang et al. 2017
Other Asian	16-CNXZ-CNXZ_25_J	Gongbujiangda Zang	CNXZ	Tibet	30	93.2	Yang et al. 2017
Other Asian	16-CNXZ-CNXZ_28_J	Gongbujiangda Zang	CNXZ	Tibet	30	93.2	Yang et al. 2017
Other Asian	16-CNXZ-CNXZ_29_J	Gongbujiangda Zang	CNXZ	Tibet	30	93.2	Yang et al. 2017
Other Asian	16-CNXZ-CNXZ_3_J	Gongbujiangda Zang	CNXZ	Tibet	30	93.2	Yang et al. 2017
Other Asian	16-CNXZ-CNXZ_4_J	Gongbujiangda Zang	CNXZ	Tibet	30	93.2	Yang et al. 2017
Other Asian	16-CNXZ-CNXZ_6_J	Gongbujiangda Zang	CNXZ	Tibet	30	93.2	Yang et al. 2017
Other Asian	16-CNXZ-CNXZ_7_J	Gongbujiangda Zang	CNXZ	Tibet	30	93.2	Yang et al. 2017
Other Asian	16-CNXZ-CNXZ_8_J	Gongbujiangda Zang	CNXZ	Tibet	30	93.2	Yang et al. 2017
Other Asian	16-CNLP-CNLP_10_W	Leping Spotted	CNLP	Jiangxi	29	117.2	Yang et al. 2017
Other Asian	16-CNLP-CNLP_11_W	Leping Spotted	CNLP	Jiangxi	29	117.2	Yang et al. 2017
Other Asian	16-CNLP-CNLP_12_W	Leping Spotted	CNLP	Jiangxi	29	117.2	Yang et al. 2017
Other Asian	16-CNLP-CNLP_13_W	Leping Spotted	CNLP	Jiangxi	29	117.2	Yang et al. 2017
Other Asian	16-CNLP-CNLP_14_W	Leping Spotted	CNLP	Jiangxi	29	117.2	Yang et al. 2017
Other Asian	16-CNLP-CNLP_15_W	Leping Spotted	CNLP	Jiangxi	29	117.2	Yang et al. 2017
Other Asian	16-CNLP-CNLP_16_W	Leping Spotted	CNLP	Jiangxi	29	117.2	Yang et al. 2017
Other Asian	16-CNLP-CNLP_17_W	Leping Spotted	CNLP	Jiangxi	29	117.2	Yang et al. 2017
Other Asian	16-CNLP-CNLP_18_W	Leping Spotted	CNLP	Jiangxi	29	117.2	Yang et al. 2017
Other Asian	16-CNLP-CNLP_19_W	Leping Spotted	CNLP	Jiangxi	29	117.2	Yang et al. 2017
Other Asian	16-CNLP-CNLP_2_W	Leping Spotted	CNLP	Jiangxi	29	117.2	Yang et al. 2017
Other Asian	16-CNLP-CNLP_20_W	Leping Spotted	CNLP	Jiangxi	29	117.2	Yang et al. 2017
Other Asian	16-CNLP-CNLP_3_W	Leping Spotted	CNLP	Jiangxi	29	117.2	Yang et al. 2017

Other Asian	16-CNLP-CNLP_4_W	Leping Spotted	CNLP	Jiangxi	29	117.2	Yang et al. 2017
Other Asian	16-CNLP-CNLP_5_W	Leping Spotted	CNLP	Jiangxi	29	117.2	Yang et al. 2017
Other Asian	16-CNLP-CNLP_6_W	Leping Spotted	CNLP	Jiangxi	29	117.2	Yang et al. 2017
Other Asian	16-CNLP-CNLP_7_W	Leping Spotted	CNLP	Jiangxi	29	117.2	Yang et al. 2017
Other Asian	16-CNLP-CNLP_8_W	Leping Spotted	CNLP	Jiangxi	29	117.2	Yang et al. 2017
Other Asian	16-CNLP-CNLP_9_W	Leping Spotted	CNLP	Jiangxi	29	117.2	Yang et al. 2017
Other Asian	16-CNLZ-CNLZ_1_J	Litang Zang	CNLZ	Sichuan	30	100.3	Yang et al. 2017
Other Asian	16-CNLZ-CNLZ_10_J	Litang Zang	CNLZ	Sichuan	30	100.3	Yang et al. 2017
Other Asian	16-CNLZ-CNLZ_11_J	Litang Zang	CNLZ	Sichuan	30	100.3	Yang et al. 2017
Other Asian	16-CNLZ-CNLZ_12_J	Litang Zang	CNLZ	Sichuan	30	100.3	Yang et al. 2017
Other Asian	16-CNLZ-CNLZ_13_J	Litang Zang	CNLZ	Sichuan	30	100.3	Yang et al. 2017
Other Asian	16-CNLZ-CNLZ_14_J	Litang Zang	CNLZ	Sichuan	30	100.3	Yang et al. 2017
Other Asian	16-CNLZ-CNLZ_15_J	Litang Zang	CNLZ	Sichuan	30	100.3	Yang et al. 2017
Other Asian	16-CNLZ-CNLZ_16_J	Litang Zang	CNLZ	Sichuan	30	100.3	Yang et al. 2017
Other Asian	16-CNLZ-CNLZ_2_J	Litang Zang	CNLZ	Sichuan	30	100.3	Yang et al. 2017
Other Asian	16-CNLZ-CNLZ_3_J	Litang Zang	CNLZ	Sichuan	30	100.3	Yang et al. 2017
Other Asian	16-CNLZ-CNLZ_5_J	Litang Zang	CNLZ	Sichuan	30	100.3	Yang et al. 2017
Other Asian	16-CNLZ-CNLZ_6_J	Litang Zang	CNLZ	Sichuan	30	100.3	Yang et al. 2017
Other Asian	16-CNLZ-CNLZ_7_J	Litang Zang	CNLZ	Sichuan	30	100.3	Yang et al. 2017
Other Asian	16-CNLZ-CNLZ_8_J	Litang Zang	CNLZ	Sichuan	30	100.3	Yang et al. 2017
Other Asian	16-CNLZ-CNLZ_9_J	Litang Zang	CNLZ	Sichuan	30	100.3	Yang et al. 2017
Other Asian	16-CNML-CNML_10_J	Milin Zang	CNML	Nyingchi	29.2	94.2	Yang et al. 2017
Other Asian	16-CNML-CNML_11_J	Milin Zang	CNML	Nyingchi	29.2	94.2	Yang et al. 2017
Other Asian	16-CNML-CNML_14_J	Milin Zang	CNML	Nyingchi	29.2	94.2	Yang et al. 2017
Other Asian	16-CNML-CNML_15_J	Milin Zang	CNML	Nyingchi	29.2	94.2	Yang et al. 2017
Other Asian	16-CNML-CNML_16_J	Milin Zang	CNML	Nyingchi	29.2	94.2	Yang et al. 2017
Other Asian	16-CNML-CNML_2_J	Milin Zang	CNML	Nyingchi	29.2	94.2	Yang et al. 2017
Other Asian	16-CNML-CNML_5_J	Milin Zang	CNML	Nyingchi	29.2	94.2	Yang et al. 2017
Other Asian	16-CNML-CNML_6_J	Milin Zang	CNML	Nyingchi	29.2	94.2	Yang et al. 2017
Other Asian	16-CNML-CNML_7_J	Milin Zang	CNML	Nyingchi	29.2	94.2	Yang et al. 2017

Other Asian	16-CNML-CNML_8_J	Milin Zang	CNML	Nyingchi	29.2	94.2	Yang et al. 2017
Other Asian	16-CNML-CNML_9_J	Milin Zang	CNML	Nyingchi	29.2	94.2	Yang et al. 2017
Other Asian	16-CNMG-CNMG_10_J	Mingguangxiaoer	CNMG	Yunnan	25	98.5	Yang et al. 2017
Other Asian	16-CNMG-CNMG_11_J	Mingguangxiaoer	CNMG	Yunnan	25	98.5	Yang et al. 2017
Other Asian	16-CNMG-CNMG_12_J	Mingguangxiaoer	CNMG	Yunnan	25	98.5	Yang et al. 2017
Other Asian	16-CNMG-CNMG_13_J	Mingguangxiaoer	CNMG	Yunnan	25	98.5	Yang et al. 2017
Other Asian	16-CNMG-CNMG_14_J	Mingguangxiaoer	CNMG	Yunnan	25	98.5	Yang et al. 2017
Other Asian	16-CNMG-CNMG_15_J	Mingguangxiaoer	CNMG	Yunnan	25	98.5	Yang et al. 2017
Other Asian	16-CNMG-CNMG_5_J	Mingguangxiaoer	CNMG	Yunnan	25	98.5	Yang et al. 2017
Other Asian	16-CNMG-CNMG_6_J	Mingguangxiaoer	CNMG	Yunnan	25	98.5	Yang et al. 2017
Other Asian	16-CNMG-CNMG_7_J	Mingguangxiaoer	CNMG	Yunnan	25	98.5	Yang et al. 2017
Other Asian	16-CNMG-CNMG_8_J	Mingguangxiaoer	CNMG	Yunnan	25	98.5	Yang et al. 2017
Other Asian	16-CNMG-CNMG_9_J	Mingguangxiaoer	CNMG	Yunnan	25	98.5	Yang et al. 2017
Other Asian	16-CNNJ-CNNJ_1_J	Neijiang	CNNJ	Sichuan	29.8	104.9	Yang et al. 2017
Other Asian	16-CNNJ-CNNJ_10_J	Neijiang	CNNJ	Sichuan	29.8	104.9	Yang et al. 2017
Other Asian	16-CNNJ-CNNJ_11_J	Neijiang	CNNJ	Sichuan	29.8	104.9	Yang et al. 2017
Other Asian	16-CNNJ-CNNJ_12_J	Neijiang	CNNJ	Sichuan	29.8	104.9	Yang et al. 2017
Other Asian	16-CNNJ-CNNJ_14_J	Neijiang	CNNJ	Sichuan	29.8	104.9	Yang et al. 2017
Other Asian	16-CNNJ-CNNJ_15_J	Neijiang	CNNJ	Sichuan	29.8	104.9	Yang et al. 2017
Other Asian	16-CNNJ-CNNJ_16_J	Neijiang	CNNJ	Sichuan	29.8	104.9	Yang et al. 2017
Other Asian	16-CNNJ-CNNJ_2_J	Neijiang	CNNJ	Sichuan	29.8	104.9	Yang et al. 2017
Other Asian	16-CNNJ-CNNJ_3_J	Neijiang	CNNJ	Sichuan	29.8	104.9	Yang et al. 2017
Other Asian	16-CNNJ-CNNJ_4_J	Neijiang	CNNJ	Sichuan	29.8	104.9	Yang et al. 2017
Other Asian	16-CNNJ-CNNJ_5_J	Neijiang	CNNJ	Sichuan	29.8	104.9	Yang et al. 2017
Other Asian	16-CNNJ-CNNJ_6_J	Neijiang	CNNJ	Sichuan	29.8	104.9	Yang et al. 2017
Other Asian	16-CNNJ-CNNJ_7_J	Neijiang	CNNJ	Sichuan	29.8	104.9	Yang et al. 2017
Other Asian	16-CNNJ-CNNJ_8_J	Neijiang	CNNJ	Sichuan	29.8	104.9	Yang et al. 2017
Other Asian	16-CNNJ-CNNJ_9_J	Neijiang	CNNJ	Sichuan	29.8	104.9	Yang et al. 2017
Other Asian	16-CNRC-CNRC_19_W	Rongchang	CNRC	Chongqin	29.6	106.2	Yang et al. 2017
Other Asian	16-CNRC-CNRC_20_W	Rongchang	CNRC	Chongqin	29.6	106.2	Yang et al. 2017

Other Asian	16-CNRC-CNRC_21_W	Rongchang	CNRC	Chongqin	29.6	106.2	Yang et al. 2017
Other Asian	16-CNRC-CNRC_22_W	Rongchang	CNRC	Chongqin	29.6	106.2	Yang et al. 2017
Other Asian	16-CNRC-CNRC_23_W	Rongchang	CNRC	Chongqin	29.6	106.2	Yang et al. 2017
Other Asian	16-CNRC-CNRC_24_W	Rongchang	CNRC	Chongqin	29.6	106.2	Yang et al. 2017
Other Asian	16-CNRC-CNRC_25_W	Rongchang	CNRC	Chongqin	29.6	106.2	Yang et al. 2017
Other Asian	16-CNRC-CNRC_26_W	Rongchang	CNRC	Chongqin	29.6	106.2	Yang et al. 2017
Other Asian	16-CNRC-CNRC_29_W	Rongchang	CNRC	Chongqin	29.6	106.2	Yang et al. 2017
Other Asian	16-CNRC-CNRC_30_W	Rongchang	CNRC	Chongqin	29.6	106.2	Yang et al. 2017
Other Asian	16-CNRC-CNRC_32_W	Rongchang	CNRC	Chongqin	29.6	106.2	Yang et al. 2017
Other Asian	16-CNRC-CNRC_33_W	Rongchang	CNRC	Chongqin	29.6	106.2	Yang et al. 2017
Other Asian	16-CNRC-CNRC_34_W	Rongchang	CNRC	Chongqin	29.6	106.2	Yang et al. 2017
Other Asian	16-CNRC-CNRC_35_W	Rongchang	CNRC	Chongqin	29.6	106.2	Yang et al. 2017
Other Asian	16-CNRC-CNRC_36_W	Rongchang	CNRC	Chongqin	29.6	106.2	Yang et al. 2017
Other Asian	RC-RC0003	Rongchang	RC	Chongqin			Ai et al 2013
Other Asian	RC-RC0004	Rongchang	RC	Chongqin			Ai et al 2013
Other Asian	RC-RC0005	Rongchang	RC	Chongqin			Ai et al 2013
Other Asian	RC-RC0006	Rongchang	RC	Chongqin			Ai et al 2013
Other Asian	RC-RC0007	Rongchang	RC	Chongqin			Ai et al 2013
Other Asian	RC-RC0008	Rongchang	RC	Chongqin			Ai et al 2013
Other Asian	RC-RC0009	Rongchang	RC	Chongqin			Ai et al 2013
Other Asian	RC-RC0010	Rongchang	RC	Chongqin			Ai et al 2013
Other Asian	RC-RC0011	Rongchang	RC	Chongqin			Ai et al 2013
Other Asian	RC-RC0012	Rongchang	RC	Chongqin			Ai et al 2013
Other Asian	RC-RC0013	Rongchang	RC	Chongqin			Ai et al 2013
Other Asian	RC-RC0014	Rongchang	RC	Chongqin			Ai et al 2013
Other Asian	RC-RC0015	Rongchang	RC	Chongqin			Ai et al 2013
Other Asian	RC-RC0016	Rongchang	RC	Chongqin			Ai et al 2013
Other Asian	RC-RC0017	Rongchang	RC	Chongqin			Ai et al 2013
Other Asian	RC-J00050	Rongchang	RC	Chongqin			Ai et al 2013
Other Asian	RC-J00051	Rongchang	RC	Chongqin			Ai et al 2013

Other Asian	RC-J00052	Rongchang	RC	Chongqin			Ai et al 2013
Other Asian	16-CNSZ-CNSZ_1_J	Shaziling	CNSZ	Hunan	27.9	112.9	Yang et al. 2017
Other Asian	16-CNSZ-CNSZ_10_J	Shaziling	CNSZ	Hunan	27.9	112.9	Yang et al. 2017
Other Asian	16-CNSZ-CNSZ_11_J	Shaziling	CNSZ	Hunan	27.9	112.9	Yang et al. 2017
Other Asian	16-CNSZ-CNSZ_2_J	Shaziling	CNSZ	Hunan	27.9	112.9	Yang et al. 2017
Other Asian	16-CNSZ-CNSZ_3_J	Shaziling	CNSZ	Hunan	27.9	112.9	Yang et al. 2017
Other Asian	16-CNSZ-CNSZ_4_J	Shaziling	CNSZ	Hunan	27.9	112.9	Yang et al. 2017
Other Asian	16-CNSZ-CNSZ_5_J	Shaziling	CNSZ	Hunan	27.9	112.9	Yang et al. 2017
Other Asian	16-CNSZ-CNSZ_6_J	Shaziling	CNSZ	Hunan	27.9	112.9	Yang et al. 2017
Other Asian	16-CNSZ-CNSZ_7_J	Shaziling	CNSZ	Hunan	27.9	112.9	Yang et al. 2017
Other Asian	16-CNSZ-CNSZ_8_J	Shaziling	CNSZ	Hunan	27.9	112.9	Yang et al. 2017
Other Asian	16-CNSZ-CNSZ_9_J	Shaziling	CNSZ	Hunan	27.9	112.9	Yang et al. 2017
Other Asian	ZZG-ZZG17	Tibetan	ZZG	Gansu			Ai et al. 2013
Other Asian	ZZG-ZZG48	Tibetan	ZZG	Gansu			Ai et al. 2013
Other Asian	ZZG-ZZG25	Tibetan	ZZG	Gansu			Ai et al. 2013
Other Asian	ZZT-B07	Tibetan	ZZT	Tibet			Ai et al. 2013
Other Asian	ZZT-B14	Tibetan	ZZT	Tibet			Ai et al. 2013
Other Asian	ZZT-B15	Tibetan	ZZT	Tibet			Ai et al. 2013
Other Asian	ZZT-B16	Tibetan	ZZT	Tibet			Ai et al. 2013
Other Asian	ZZT-B20	Tibetan	ZZT	Tibet			Ai et al. 2013
Other Asian	ZZT-B21	Tibetan	ZZT	Tibet			Ai et al. 2013
Other Asian	ZZT-B26	Tibetan	ZZT	Tibet			Ai et al. 2013
Other Asian	ZZT-B28	Tibetan	ZZT	Tibet			Ai et al. 2013
Other Asian	ZZT-B35	Tibetan	ZZT	Tibet			Ai et al. 2013
Other Asian	ZZT-B36	Tibetan	ZZT	Tibet			Ai et al. 2013
Other Asian	16-CNTC-CNTC_1_J	Tongcheng	CNTC	Hubei	29.2	113.8	Yang et al. 2017
Other Asian	16-CNTC-CNTC_10_J	Tongcheng	CNTC	Hubei	29.2	113.8	Yang et al. 2017
Other Asian	16-CNTC-CNTC_11_J	Tongcheng	CNTC	Hubei	29.2	113.8	Yang et al. 2017
Other Asian	16-CNTC-CNTC_13_J	Tongcheng	CNTC	Hubei	29.2	113.8	Yang et al. 2017
Other Asian	16-CNTC-CNTC_14_J	Tongcheng	CNTC	Hubei	29.2	113.8	Yang et al. 2017

Other Asian	16-CNTC-CNTC_16_J	Tongcheng	CNTC	Hubei	29.2	113.8	Yang et al. 2017
Other Asian	16-CNTC-CNTC_2_J	Tongcheng	CNTC	Hubei	29.2	113.8	Yang et al. 2017
Other Asian	16-CNTC-CNTC_3_J	Tongcheng	CNTC	Hubei	29.2	113.8	Yang et al. 2017
Other Asian	16-CNTC-CNTC_4_J	Tongcheng	CNTC	Hubei	29.2	113.8	Yang et al. 2017
Other Asian	16-CNTC-CNTC_5_J	Tongcheng	CNTC	Hubei	29.2	113.8	Yang et al. 2017
Other Asian	16-CNTC-CNTC_6_J	Tongcheng	CNTC	Hubei	29.2	113.8	Yang et al. 2017
Other Asian	16-CNTC-CNTC_7_J	Tongcheng	CNTC	Hubei	29.2	113.8	Yang et al. 2017
Other Asian	16-CNTC-CNTC_8_J	Tongcheng	CNTC	Hubei	29.2	113.8	Yang et al. 2017
Other Asian	16-CNTC-CNTC_9_J	Tongcheng	CNTC	Hubei	29.2	113.8	Yang et al. 2017
Other Asian	16-KPWB-KPWB_1_W	WB_Korea	KPWB	South Korea	36.6	127.9	Yang et al. 2017
Other Asian	16-KPWB-KPWB_2_W	WB_Korea	KPWB	South Korea	36.6	127.9	Yang et al. 2017
Other Asian	16-KPWB-KPWB_3_W	WB_Korea	KPWB	South Korea	36.6	127.9	Yang et al. 2017
Other Asian	16-KPWB-KPWB_4_W	WB_Korea	KPWB	South Korea	36.6	127.9	Yang et al. 2017
Other Asian	16-KPWB-KPWB_5_W	WB_Korea	KPWB	South Korea	36.6	127.9	Yang et al. 2017
Other Asian	16-CNWB2-CNWB2_1_W	WB northern China and eastern Russia	CNWB2		NA	NA	Yang et al. 2017
Other Asian	16-CNWB2-CNWB2_2_W	WB northern China and eastern Russia	CNWB2		NA	NA	Yang et al. 2017
Other Asian	16-CNWB2-CNWB2_3_W	WB northern China and eastern Russia	CNWB2		NA	NA	Yang et al. 2017
Other Asian	16-CNWB2-CNWB2_4_W	WB northern China and eastern Russia	CNWB2		NA	NA	Yang et al. 2017
Other Asian	16-CNWB2-CNWB2_5_W	WB northern China and eastern Russia	CNWB2		NA	NA	Yang et al. 2017
Other Asian	16-RUWB1-RUWB1_1_W	WB northern China and eastern Russia	RUWB1	Russia	62.3	99	Yang et al. 2017
Other Asian	16-RUWB1-RUWB1_10_W	WB northern China and eastern Russia	RUWB1	Russia	62.3	99	Yang et al. 2017
Other Asian	16-RUWB1-RUWB1_11_W	WB northern China and eastern Russia	RUWB1	Russia	62.3	99	Yang et al. 2017

Other Asian	16-RUWB1-RUWB1_12_W	WB northern China and eastern Russia	RUWB1	Russia	62.3	99	Yang et al. 2017
Other Asian	16-RUWB1-RUWB1_13_W	WB northern China and eastern Russia	RUWB1	Russia	62.3	99	Yang et al. 2017
Other Asian	16-RUWB1-RUWB1_2_W	WB northern China and eastern Russia	RUWB1	Russia	62.3	99	Yang et al. 2017
Other Asian	16-RUWB1-RUWB1_3_W	WB_northern China and eastern Russia	RUWB1	Russia	62.3	99	Yang et al. 2017
Other Asian	16-RUWB1-RUWB1_4_W	WB_northern China and eastern Russia	RUWB1	Russia	62.3	99	Yang et al. 2017
Other Asian	16-RUWB1-RUWB1_5_W	WB_northern China and eastern Russia	RUWB1	Russia	62.3	99	Yang et al. 2017
Other Asian	16-RUWB1-RUWB1_6_W	WB_northern China and eastern Russia	RUWB1	Russia	62.3	99	Yang et al. 2017
Other Asian	16-RUWB1-RUWB1_7_W	WB_northern China and eastern Russia	RUWB1	Russia	62.3	99	Yang et al. 2017
Other Asian	16-RUWB1-RUWB1_8_W	WB_northern China and eastern Russia	RUWB1	Russia	62.3	99	Yang et al. 2017
Other Asian	16-RUWB1-RUWB1_9_W	WB_northern China and eastern Russia	RUWB1	Russia	62.3	99	Yang et al. 2017
Other Asian	16-RUWB2-RUWB2_1_J	WB_northern China and eastern Russia	RUWB2	Russia	47.1	136.6	Yang et al. 2017
Other Asian	16-RUWB2-RUWB2_10_J	WB_northern China and eastern Russia	RUWB2	Russia	47.1	136.6	Yang et al. 2017
Other Asian	16-RUWB2-RUWB2_11_J	WB_northern China and eastern Russia	RUWB2	Russia	47.1	136.6	Yang et al. 2017
Other Asian	16-RUWB2-RUWB2_2_J	WB_northern China and eastern Russia	RUWB2	Russia	47.1	136.6	Yang et al. 2017
Other Asian	16-RUWB2-RUWB2_3_J	WB_northern China and eastern Russia	RUWB2	Russia	47.1	136.6	Yang et al. 2017
Other Asian	16-RUWB2-RUWB2_4_J	WB_northern China and eastern Russia	RUWB2	Russia	47.1	136.6	Yang et al. 2017
Other Asian	16-RUWB2-RUWB2_5_J	WB_northern China and eastern Russia	RUWB2	Russia	47.1	136.6	Yang et al. 2017
Other Asian	16-RUWB2-RUWB2_6_J	WB_northern China and eastern Russia	RUWB2	Russia	47.1	136.6	Yang et al. 2017

Other Asian	16-RUWB2-RUWB2_7_J	WB_northern China and eastern Russia	RUWB2	Russia	47.1	136.6	Yang et al. 2017
Other Asian	16-RUWB2-RUWB2_8_J	WB_northern China and eastern Russia	RUWB2	Russia	47.1	136.6	Yang et al. 2017
Other Asian	16-RUWB2-RUWB2_9_J	WB_northern China and eastern Russia	RUWB2	Russia	47.1	136.6	Yang et al. 2017
Other Asian	16-CNWB1-CNWB1_1_W	WB_southern China	CNWB1		NA	NA	Yang et al. 2017
Other Asian	16-CNWB1-CNWB1_2_W	WB_southern China	CNWB1		NA	NA	Yang et al. 2017
Other Asian	16-CNWB1-CNWB1_3_W	WB_southern China	CNWB1		NA	NA	Yang et al. 2017
Other Asian	16-CNWB1-CNWB1_4_W	WB_southern China	CNWB1		NA	NA	Yang et al. 2017
Other Asian	16-CNWB1-CNWB1_5_W	WB_southern China	CNWB1		NA	NA	Yang et al. 2017
Other Asian	16-CNWB3-CNWB3_1_J	WB_southern China	CNWB3	Jiangxi	28.7	115.9	Yang et al. 2017
Other Asian	16-CNWB3-CNWB3_10_J	WB_southern China	CNWB3	Jiangxi	28.7	115.9	Yang et al. 2017
Other Asian	16-CNWB3-CNWB3_11_J	WB_southern China	CNWB3	Jiangxi	28.7	115.9	Yang et al. 2017
Other Asian	16-CNWB3-CNWB3_12_J	WB_southern China	CNWB3	Jiangxi	28.7	115.9	Yang et al. 2017
Other Asian	16-CNWB3-CNWB3_13_J	WB_southern China	CNWB3	Jiangxi	28.7	115.9	Yang et al. 2017
Other Asian	16-CNWB3-CNWB3_14_J	WB_southern China	CNWB3	Jiangxi	28.7	115.9	Yang et al. 2017
Other Asian	16-CNWB3-CNWB3_15_J	WB_southern China	CNWB3	Jiangxi	28.7	115.9	Yang et al. 2017
Other Asian	16-CNWB3-CNWB3_2_J	WB_southern China	CNWB3	Jiangxi	28.7	115.9	Yang et al. 2017
Other Asian	16-CNWB3-CNWB3_3_J	WB_southern China	CNWB3	Jiangxi	28.7	115.9	Yang et al. 2017
Other Asian	16-CNWB3-CNWB3_4_J	WB_southern China	CNWB3	Jiangxi	28.7	115.9	Yang et al. 2017
Other Asian	16-CNWB3-CNWB3_5_J	WB_southern China	CNWB3	Jiangxi	28.7	115.9	Yang et al. 2017
Other Asian	16-CNWB3-CNWB3_6_J	WB_southern China	CNWB3	Jiangxi	28.7	115.9	Yang et al. 2017
Other Asian	16-CNWB3-CNWB3_7_J	WB_southern China	CNWB3	Jiangxi	28.7	115.9	Yang et al. 2017
Other Asian	16-CNWB3-CNWB3_8_J	WB_southern China	CNWB3	Jiangxi	28.7	115.9	Yang et al. 2017
Other Asian	16-CNWB3-CNWB3_9_J	WB_southern China	CNWB3	Jiangxi	28.7	115.9	Yang et al. 2017
Other Asian	16-CNWB4-CNWB4_1_J	WB_southern China	CNWB4	Jiangxi	25.8	114.6	Yang et al. 2017
Other Asian	16-CNWB4-CNWB4_2_J	WB_southern China	CNWB4	Jiangxi	25.8	114.6	Yang et al. 2017
Other Asian	16-CNWB4-CNWB4_3_J	WB_southern China	CNWB4	Jiangxi	25.8	114.6	Yang et al. 2017
Other Asian	16-CNWB4-CNWB4_4_J	WB_southern China	CNWB4	Jiangxi	25.8	114.6	Yang et al. 2017
Other Asian	TC-TC0041		TC	Hubei			Ai et al. 2013

Other Asian	TC-TC0047		TC	Hubei			Ai et al. 2013
Eastern China	16-CNEH-CNEH_1_J	Erhualian	CNEH	Jiangsu	31.7	119.9	Yang et al. 2017
Eastern China	16-CNEH-CNEH_10_J	Erhualian	CNEH	Jiangsu	31.7	119.9	Yang et al. 2017
Eastern China	16-CNEH-CNEH_11_J	Erhualian	CNEH	Jiangsu	31.7	119.9	Yang et al. 2017
Eastern China	16-CNEH-CNEH_12_J	Erhualian	CNEH	Jiangsu	31.7	119.9	Yang et al. 2017
Eastern China	16-CNEH-CNEH_13_J	Erhualian	CNEH	Jiangsu	31.7	119.9	Yang et al. 2017
Eastern China	16-CNEH-CNEH_14_J	Erhualian	CNEH	Jiangsu	31.7	119.9	Yang et al. 2017
Eastern China	16-CNEH-CNEH_15_J	Erhualian	CNEH	Jiangsu	31.7	119.9	Yang et al. 2017
Eastern China	16-CNEH-CNEH_16_J	Erhualian	CNEH	Jiangsu	31.7	119.9	Yang et al. 2017
Eastern China	16-CNEH-CNEH_2_J	Erhualian	CNEH	Jiangsu	31.7	119.9	Yang et al. 2017
Eastern China	16-CNEH-CNEH_3_J	Erhualian	CNEH	Jiangsu	31.7	119.9	Yang et al. 2017
Eastern China	16-CNEH-CNEH_4_J	Erhualian	CNEH	Jiangsu	31.7	119.9	Yang et al. 2017
Eastern China	16-CNEH-CNEH_5_J	Erhualian	CNEH	Jiangsu	31.7	119.9	Yang et al. 2017
Eastern China	16-CNEH-CNEH_6_J	Erhualian	CNEH	Jiangsu	31.7	119.9	Yang et al. 2017
Eastern China	16-CNEH-CNEH_7_J	Erhualian	CNEH	Jiangsu	31.7	119.9	Yang et al. 2017
Eastern China	16-CNEH-CNEH_8_J	Erhualian	CNEH	Jiangsu	31.7	119.9	Yang et al. 2017
Eastern China	16-CNEH-CNEH_9_J	Erhualian	CNEH	Jiangsu	31.7	119.9	Yang et al. 2017
Eastern China	EHL-ER0013	Erhualian	EHL	Jiangsu			Ai et al. 2013
Eastern China	EHL-ER_CS0000	Erhualian	EHL	Jiangsu			Ai et al. 2013
Eastern China	EHL-ER_CS0234	Erhualian	EHL	Jiangsu			Ai et al. 2013
Eastern China	EHL-ER_CS0806	Erhualian	EHL	Jiangsu			Ai et al. 2013
Eastern China	EHL-ER_CS3000	Erhualian	EHL	Jiangsu			Ai et al. 2013
Eastern China	EHL-ER_CS3188	Erhualian	EHL	Jiangsu			Ai et al. 2013
Eastern China	EHL-ER_CS3508	Erhualian	EHL	Jiangsu			Ai et al. 2013
Eastern China	EHL-ER_CS3544	Erhualian	EHL	Jiangsu			Ai et al. 2013
Eastern China	EHL-ER_CS3566	Erhualian	EHL	Jiangsu			Ai et al. 2013
Eastern China	EHL-ER_JX0001	Erhualian	EHL	Jiangsu			Ai et al. 2013
Eastern China	EHL-ER_JX0002	Erhualian	EHL	Jiangsu			Ai et al. 2013
Eastern China	EHL-ER_JX0003	Erhualian	EHL	Jiangsu			Ai et al. 2013
Eastern China	EHL-ER_JX0004	Erhualian	EHL	Jiangsu			Ai et al. 2013

Eastern China	EHL-ER_JX0005	Erhualian	EHL	Jiangsu			Ai et al. 2013
Eastern China	EHL-ER_JX0006	Erhualian	EHL	Jiangsu			Ai et al. 2013
Eastern China	EHL-ER_JX0007	Erhualian	EHL	Jiangsu			Ai et al. 2013
Eastern China	16-CNJQ-CNJQ_1_W	Jiangquhai	CNJQ	Jiangsu	32.5	119.9	Yang et al. 2017
Eastern China	16-CNJQ-CNJQ_10_W	Jiangquhai	CNJQ	Jiangsu	32.5	119.9	Yang et al. 2017
Eastern China	16-CNJQ-CNJQ_2_W	Jiangquhai	CNJQ	Jiangsu	32.5	119.9	Yang et al. 2017
Eastern China	16-CNJQ-CNJQ_3_W	Jiangquhai	CNJQ	Jiangsu	32.5	119.9	Yang et al. 2017
Eastern China	16-CNJQ-CNJQ_4_W	Jiangquhai	CNJQ	Jiangsu	32.5	119.9	Yang et al. 2017
Eastern China	16-CNJQ-CNJQ_6_W	Jiangquhai	CNJQ	Jiangsu	32.5	119.9	Yang et al. 2017
Eastern China	16-CNJQ-CNJQ_7_W	Jiangquhai	CNJQ	Jiangsu	32.5	119.9	Yang et al. 2017
Eastern China	16-CNJQ-CNJQ_8_W	Jiangquhai	CNJQ	Jiangsu	32.5	119.9	Yang et al. 2017
Eastern China	16-CNJQ-CNJQ_9_W	Jiangquhai	CNJQ	Jiangsu	32.5	119.9	Yang et al. 2017
Eastern China	16-CNMS-CNMS_10_W	Meishan	CNMS	Jiangsu	31.5	121.1	Yang et al. 2017
Eastern China	16-CNMS-CNMS_11_W	Meishan	CNMS	Jiangsu	31.5	121.1	Yang et al. 2017
Eastern China	16-CNMS-CNMS_12_W	Meishan	CNMS	Jiangsu	31.5	121.1	Yang et al. 2017
Eastern China	16-CNMS-CNMS_13_W	Meishan	CNMS	Jiangsu	31.5	121.1	Yang et al. 2017
Eastern China	16-CNMS-CNMS_15_W	Meishan	CNMS	Jiangsu	31.5	121.1	Yang et al. 2017
Eastern China	16-CNMS-CNMS_16_W	Meishan	CNMS	Jiangsu	31.5	121.1	Yang et al. 2017
Eastern China	16-CNMS-CNMS_17_W	Meishan	CNMS	Jiangsu	31.5	121.1	Yang et al. 2017
Eastern China	16-CNMS-CNMS_18_W	Meishan	CNMS	Jiangsu	31.5	121.1	Yang et al. 2017
Eastern China	16-CNMS-CNMS_19_W	Meishan	CNMS	Jiangsu	31.5	121.1	Yang et al. 2017
Eastern China	16-CNMS-CNMS_20_W	Meishan	CNMS	Jiangsu	31.5	121.1	Yang et al. 2017
Eastern China	16-CNMS-CNMS_21_W	Meishan	CNMS	Jiangsu	31.5	121.1	Yang et al. 2017
Eastern China	16-CNMS-CNMS_25_W	Meishan	CNMS	Jiangsu	31.5	121.1	Yang et al. 2017
Eastern China	16-CNMS-CNMS_31_W	Meishan	CNMS	Jiangsu	31.5	121.1	Yang et al. 2017
Eastern China	16-CNMS-CNMS_33_W	Meishan	CNMS	Jiangsu	31.5	121.1	Yang et al. 2017
Eastern China	16-CNMS-CNMS_34_W	Meishan	CNMS	Jiangsu	31.5	121.1	Yang et al. 2017
Eastern China	16-CNMS-CNMS_37_W	Meishan	CNMS	Jiangsu	31.5	121.1	Yang et al. 2017
Eastern China	16-CNMS-CNMS_6_W	Meishan	CNMS	Jiangsu	31.5	121.1	Yang et al. 2017
Eastern China	16-CNMS-CNMS_7_W	Meishan	CNMS	Jiangsu	31.5	121.1	Yang et al. 2017

Eastern China	16-CNMS-CNMS_8_W	Meishan	CNMS	Jiangsu	31.5	121.1	Yang et al. 2017
Eastern China	16-CNMS-CNMS_9_W	Meishan	CNMS	Jiangsu	31.5	121.1	Yang et al. 2017
Eastern China	16-6-CNMS02F01	Meishan	CNMS02				Burgos-Paz et al. 2013
Eastern China	16-6-CNMS02F02	Meishan	CNMS02				Burgos-Paz et al. 2013
Eastern China	16-6-CNMS02F03	Meishan	CNMS02				Burgos-Paz et al. 2013
Eastern China	16-6-CNMS02F04	Meishan	CNMS02				Burgos-Paz et al. 2013
Eastern China	16-6-CNMS02F05	Meishan	CNMS02				Burgos-Paz et al. 2013
Eastern China	16-6-CNMS20M01	Meishan	CNMS20				Burgos-Paz et al. 2013
Eastern China	16-6-CNMS20M05	Meishan	CNMS20				Burgos-Paz et al. 2013
Eastern China	16-6-CNMS20M07	Meishan	CNMS20				Burgos-Paz et al. 2013
Eastern China	16-6-CNMS20M08	Meishan	CNMS20				Burgos-Paz et al. 2013
Eastern China	16-6-CNMS20M09	Meishan	CNMS20				Burgos-Paz et al. 2013
Eastern China	16-6-CNMS20U13	Meishan	CNMS20				Burgos-Paz et al. 2013
Eastern China	16-6-CNMS21M01	Meishan	CNMS21				Burgos-Paz et al. 2013
Eastern China	16-6-CNMS21M02	Meishan	CNMS21				Burgos-Paz et al. 2013
Eastern China	16-6-CNMS21M03	Meishan	CNMS21				Burgos-Paz et al. 2013
Eastern China	16-6-CNMS21M04	Meishan	CNMS21				Burgos-Paz et al. 2013
Eastern China	16-6-CNMS21M05	Meishan	CNMS21				Burgos-Paz et al. 2013
Eastern China	16-6-CNMS21M08	Meishan	CNMS21				Burgos-Paz et al. 2013

Eastern China	16-2176	Meishan/Fengjing	21				Smyser et al. 2020
Eastern China	16-2178	Meishan/Fengjing	21				Smyser et al. 2020
Eastern China	16-2180	Meishan/Fengjing	21				Smyser et al. 2020
Eastern China	16-2182	Meishan/Fengjing	21				Smyser et al. 2020
Eastern China	16-2183	Meishan/Fengjing	21				Smyser et al. 2020
Eastern China	16-2184	Meishan/Fengjing	21				Smyser et al. 2020
Eastern China	16-2185	Meishan/Fengjing	21				Smyser et al. 2020
Eastern China	16-2186	Meishan/Fengjing	21				Smyser et al. 2020
Eastern China	16-2187	Meishan/Fengjing	21				Smyser et al. 2020
Eastern China	16-2188	Meishan/Fengjing	21				Smyser et al. 2020
Eastern China	16-2189	Meishan/Fengjing	21				Smyser et al. 2020
Eastern China	16-2190	Meishan/Fengjing	21				Smyser et al. 2020
Eastern China	16-2191	Meishan/Fengjing	21				Smyser et al. 2020
Eastern China	16-2193	Meishan/Fengjing	21				Smyser et al. 2020
Eastern China	16-2194	Meishan/Fengjing	21				Smyser et al. 2020
Eastern China	16-2195	Meishan/Fengjing	21				Smyser et al. 2020
Eastern China	16-2196	Meishan/Fengjing	21				Smyser et al. 2020
Eastern China	16-2197	Meishan/Fengjing	21				Smyser et al. 2020
Eastern China	16-2198	Meishan/Fengjing	21				Smyser et al. 2020
Eastern China	16-2152	Meishan/Fengjing	21				Smyser et al. 2020
Eastern China	16-2154	Meishan/Fengjing	21				Smyser et al. 2020
Eastern China	16-2155	Meishan/Fengjing	21				Smyser et al. 2020
Eastern China	16-2156	Meishan/Fengjing	21				Smyser et al. 2020
Eastern China	16-2157	Meishan/Fengjing	21				Smyser et al. 2020
Eastern China	16-2159	Meishan/Fengjing	21				Smyser et al. 2020
Eastern China	16-2160	Meishan/Fengjing	21				Smyser et al. 2020
Eastern China	16-2161	Meishan/Fengjing	21				Smyser et al. 2020
Eastern China	16-2162	Meishan/Fengjing	21				Smyser et al. 2020
Eastern China	16-2163	Meishan/Fengjing	21				Smyser et al. 2020
Eastern China	16-2164	Meishan/Fengjing	21				Smyser et al. 2020

Eastern China	16-2165	Meishan/Fengjing	21				Smyser et al. 2020
Eastern China	16-2166	Meishan/Fengjing	21				Smyser et al. 2020
Eastern China	16-2168	Meishan/Fengjing	21				Smyser et al. 2020
Eastern China	16-2169	Meishan/Fengjing	21				Smyser et al. 2020
Eastern China	16-2170	Meishan/Fengjing	21				Smyser et al. 2020
Eastern China	16-2172	Meishan/Fengjing	21				Smyser et al. 2020
Eastern China	16-2173	Meishan/Fengjing	21				Smyser et al. 2020
Eastern China	16-2174	Meishan/Fengjing	21				Smyser et al. 2020
Eastern China	16-2175	Meishan/Fengjing	21				Smyser et al. 2020
Eastern China	16-CNWN-CNWN_10_W	Wannan Spotted	CNWN	Anhui	29.9	117.9	Yang et al. 2017
Eastern China	16-CNWN-CNWN_11_W	Wannan Spotted	CNWN	Anhui	29.9	117.9	Yang et al. 2017
Eastern China	16-CNWN-CNWN_12_W	Wannan Spotted	CNWN	Anhui	29.9	117.9	Yang et al. 2017
Eastern China	16-CNWN-CNWN_14_W	Wannan Spotted	CNWN	Anhui	29.9	117.9	Yang et al. 2017
Eastern China	16-CNWN-CNWN_15_W	Wannan Spotted	CNWN	Anhui	29.9	117.9	Yang et al. 2017
Eastern China	16-CNWN-CNWN_16_W	Wannan Spotted	CNWN	Anhui	29.9	117.9	Yang et al. 2017
Eastern China	16-CNWN-CNWN_17_W	Wannan Spotted	CNWN	Anhui	29.9	117.9	Yang et al. 2017
Eastern China	16-CNWN-CNWN_18_W	Wannan Spotted	CNWN	Anhui	29.9	117.9	Yang et al. 2017
Eastern China	16-CNWN-CNWN_2_W	Wannan Spotted	CNWN	Anhui	29.9	117.9	Yang et al. 2017
Eastern China	16-CNWN-CNWN_3_W	Wannan Spotted	CNWN	Anhui	29.9	117.9	Yang et al. 2017
Eastern China	16-CNWN-CNWN_4_W	Wannan Spotted	CNWN	Anhui	29.9	117.9	Yang et al. 2017
Eastern China	16-CNWN-CNWN_5_W	Wannan Spotted	CNWN	Anhui	29.9	117.9	Yang et al. 2017
Eastern China	16-CNWN-CNWN_6_W	Wannan Spotted	CNWN	Anhui	29.9	117.9	Yang et al. 2017
Eastern China	16-CNWN-CNWN_7_W	Wannan Spotted	CNWN	Anhui	29.9	117.9	Yang et al. 2017
Eastern China	16-CNWN-CNWN_8_W	Wannan Spotted	CNWN	Anhui	29.9	117.9	Yang et al. 2017
Eastern China	16-CNWN-CNWN_9_W	Wannan Spotted	CNWN	Anhui	29.9	117.9	Yang et al. 2017
Western China	16-CNBM-CNBM_1_J	Bamei	CNBM	Gansu	35.7	107.6	Yang et al. 2017
Western China	16-CNBM-CNBM_10_J	Bamei	CNBM	Gansu	35.7	107.6	Yang et al. 2017
Western China	16-CNBM-CNBM_11_J	Bamei	CNBM	Gansu	35.7	107.6	Yang et al. 2017
Western China	16-CNBM-CNBM_12_J	Bamei	CNBM	Gansu	35.7	107.6	Yang et al. 2017
Western China	16-CNBM-CNBM_13_J	Bamei	CNBM	Gansu	35.7	107.6	Yang et al. 2017

Western China	16-CNBM-CNBM_14_J	Bamei	CNBM	Gansu	35.7	107.6	Yang et al. 2017
Western China	16-CNBM-CNBM_16_J	Bamei	CNBM	Gansu	35.7	107.6	Yang et al. 2017
Western China	16-CNBM-CNBM_2_J	Bamei	CNBM	Gansu	35.7	107.6	Yang et al. 2017
Western China	16-CNBM-CNBM_3_J	Bamei	CNBM	Gansu	35.7	107.6	Yang et al. 2017
Western China	16-CNBM-CNBM_4_J	Bamei	CNBM	Gansu	35.7	107.6	Yang et al. 2017
Western China	16-CNBM-CNBM_6_J	Bamei	CNBM	Gansu	35.7	107.6	Yang et al. 2017
Western China	16-CNBM-CNBM_8_J	Bamei	CNBM	Gansu	35.7	107.6	Yang et al. 2017
Western China	16-CNBM-CNBM_9_J	Bamei	CNBM	Gansu	35.7	107.6	Yang et al. 2017
Western China	16-CNGU-CNGU_10_W	Guanling	CNGU	Guizhou	25.9	105.6	Yang et al. 2017
Western China	16-CNGU-CNGU_11_W	Guanling	CNGU	Guizhou	25.9	105.6	Yang et al. 2017
Western China	16-CNGU-CNGU_12_W	Guanling	CNGU	Guizhou	25.9	105.6	Yang et al. 2017
Western China	16-CNGU-CNGU_13_W	Guanling	CNGU	Guizhou	25.9	105.6	Yang et al. 2017
Western China	16-CNGU-CNGU_14_W	Guanling	CNGU	Guizhou	25.9	105.6	Yang et al. 2017
Western China	16-CNGU-CNGU_15_W	Guanling	CNGU	Guizhou	25.9	105.6	Yang et al. 2017
Western China	16-CNGU-CNGU_17_W	Guanling	CNGU	Guizhou	25.9	105.6	Yang et al. 2017
Western China	16-CNGU-CNGU_3_W	Guanling	CNGU	Guizhou	25.9	105.6	Yang et al. 2017
Western China	16-CNGU-CNGU_4_W	Guanling	CNGU	Guizhou	25.9	105.6	Yang et al. 2017
Western China	16-CNGU-CNGU_5_W	Guanling	CNGU	Guizhou	25.9	105.6	Yang et al. 2017
Western China	16-CNGU-CNGU_7_W	Guanling	CNGU	Guizhou	25.9	105.6	Yang et al. 2017
Western China	16-CNGU-CNGU_9_W	Guanling	CNGU	Guizhou	25.9	105.6	Yang et al. 2017
Western China	16-CNHT-CNHT_10_J	Hetaodaer	CNHT	Inner Mongolia	40.8	107.4	Yang et al. 2017
Western China	16-CNHT-CNHT_11_J	Hetaodaer	CNHT	Inner Mongolia	40.8	107.4	Yang et al. 2017
Western China	16-CNHT-CNHT_12_J	Hetaodaer	CNHT	Inner Mongolia	40.8	107.4	Yang et al. 2017
Western China	16-CNHT-CNHT_14_J	Hetaodaer	CNHT	Inner Mongolia	40.8	107.4	Yang et al. 2017
Western China	16-CNHT-CNHT_15_J	Hetaodaer	CNHT	Inner Mongolia	40.8	107.4	Yang et al. 2017
Western China	16-CNHT-CNHT_16_J	Hetaodaer	CNHT	Inner Mongolia	40.8	107.4	Yang et al. 2017

Western China	16-CNHT-CNHT_2_J	Hetaodaer	CNHT	Inner Mongolia	40.8	107.4	Yang et al. 2017
Western China	16-CNHT-CNHT_3_J	Hetaodaer	CNHT	Inner Mongolia	40.8	107.4	Yang et al. 2017
Western China	16-CNHT-CNHT_4_J	Hetaodaer	CNHT	Inner Mongolia	40.8	107.4	Yang et al. 2017
Western China	16-CNHT-CNHT_5_J	Hetaodaer	CNHT	Inner Mongolia	40.8	107.4	Yang et al. 2017
Western China	16-CNHT-CNHT_6_J	Hetaodaer	CNHT	Inner Mongolia	40.8	107.4	Yang et al. 2017
Western China	16-CNHT-CNHT_7_J	Hetaodaer	CNHT	Inner Mongolia	40.8	107.4	Yang et al. 2017
Western China	16-CNHT-CNHT_9_J	Hetaodaer	CNHT	Inner Mongolia	40.8	107.4	Yang et al. 2017
Western China	16-CNLH-CNLH_1_J	Laiwuhei	CNLH	Ji Nan Shi	36.2	117.7	Yang et al. 2017
Western China	16-CNLH-CNLH_10_J	Laiwuhei	CNLH	Ji Nan Shi	36.2	117.7	Yang et al. 2017
Western China	16-CNLH-CNLH_11_J	Laiwuhei	CNLH	Ji Nan Shi	36.2	117.7	Yang et al. 2017
Western China	16-CNLH-CNLH_12_J	Laiwuhei	CNLH	Ji Nan Shi	36.2	117.7	Yang et al. 2017
Western China	16-CNLH-CNLH_13_J	Laiwuhei	CNLH	Ji Nan Shi	36.2	117.7	Yang et al. 2017
Western China	16-CNLH-CNLH_14_J	Laiwuhei	CNLH	Ji Nan Shi	36.2	117.7	Yang et al. 2017
Western China	16-CNLH-CNLH_15_J	Laiwuhei	CNLH	Ji Nan Shi	36.2	117.7	Yang et al. 2017
Western China	16-CNLH-CNLH_16_J	Laiwuhei	CNLH	Ji Nan Shi	36.2	117.7	Yang et al. 2017
Western China	16-CNLH-CNLH_17_J	Laiwuhei	CNLH	Ji Nan Shi	36.2	117.7	Yang et al. 2017
Western China	16-CNLH-CNLH_18_J	Laiwuhei	CNLH	Ji Nan Shi	36.2	117.7	Yang et al. 2017
Western China	16-CNLH-CNLH_2_J	Laiwuhei	CNLH	Ji Nan Shi	36.2	117.7	Yang et al. 2017
Western China	16-CNLH-CNLH_3_J	Laiwuhei	CNLH	Ji Nan Shi	36.2	117.7	Yang et al. 2017
Western China	16-CNLH-CNLH_4_J	Laiwuhei	CNLH	Ji Nan Shi	36.2	117.7	Yang et al. 2017
Western China	16-CNLH-CNLH_5_J	Laiwuhei	CNLH	Ji Nan Shi	36.2	117.7	Yang et al. 2017
Western China	16-CNLH-CNLH_6_J	Laiwuhei	CNLH	Ji Nan Shi	36.2	117.7	Yang et al. 2017
Western China	16-CNLH-CNLH_7_J	Laiwuhei	CNLH	Ji Nan Shi	36.2	117.7	Yang et al. 2017
Western China	16-CNLH-CNLH_8_J	Laiwuhei	CNLH	Ji Nan Shi	36.2	117.7	Yang et al. 2017
Western China	16-CNLH-CNLH_9_J	Laiwuhei	CNLH	Ji Nan Shi	36.2	117.7	Yang et al. 2017

Southeast Asian	16-CNBX-CNBX_1_J	Bamaxiang	CNBX	Guangxi	24.2	107.3	Yang et al. 2017
Southeast Asian	16-CNBX-CNBX_10_J	Bamaxiang	CNBX	Guangxi	24.2	107.3	Yang et al. 2017
Southeast Asian	16-CNBX-CNBX_11_J	Bamaxiang	CNBX	Guangxi	24.2	107.3	Yang et al. 2017
Southeast Asian	16-CNBX-CNBX_12_J	Bamaxiang	CNBX	Guangxi	24.2	107.3	Yang et al. 2017
Southeast Asian	16-CNBX-CNBX_13_J	Bamaxiang	CNBX	Guangxi	24.2	107.3	Yang et al. 2017
Southeast Asian	16-CNBX-CNBX_14_J	Bamaxiang	CNBX	Guangxi	24.2	107.3	Yang et al. 2017
Southeast Asian	16-CNBX-CNBX_15_J	Bamaxiang	CNBX	Guangxi	24.2	107.3	Yang et al. 2017
Southeast Asian	16-CNBX-CNBX_16_J	Bamaxiang	CNBX	Guangxi	24.2	107.3	Yang et al. 2017
Southeast Asian	16-CNBX-CNBX_2_J	Bamaxiang	CNBX	Guangxi	24.2	107.3	Yang et al. 2017
Southeast Asian	16-CNBX-CNBX_3_J	Bamaxiang	CNBX	Guangxi	24.2	107.3	Yang et al. 2017
Southeast Asian	16-CNBX-CNBX_4_J	Bamaxiang	CNBX	Guangxi	24.2	107.3	Yang et al. 2017
Southeast Asian	16-CNBX-CNBX_5_J	Bamaxiang	CNBX	Guangxi	24.2	107.3	Yang et al. 2017
Southeast Asian	16-CNBX-CNBX_6_J	Bamaxiang	CNBX	Guangxi	24.2	107.3	Yang et al. 2017
Southeast Asian	16-CNBX-CNBX_7_J	Bamaxiang	CNBX	Guangxi	24.2	107.3	Yang et al. 2017
Southeast Asian	16-CNBX-CNBX_8_J	Bamaxiang	CNBX	Guangxi	24.2	107.3	Yang et al. 2017
Southeast Asian	16-CNBX-CNBX_9_J	Bamaxiang	CNBX	Guangxi	24.2	107.3	Yang et al. 2017
Southeast Asian	16-CNCJ-CNCJ_1_J	Congjiangxiang	CNCJ	Guangxi	25.4	108.5	Yang et al. 2017
Southeast Asian	16-CNCJ-CNCJ_10_J	Congjiangxiang	CNCJ	Guangxi	25.4	108.5	Yang et al. 2017

Southeast Asian	16-CNCJ-CNCJ_11_J	Congjiangxiang	CNCJ	Guangxi	25.4	108.5	Yang et al. 2017
Southeast Asian	16-CNCJ-CNCJ_13_J	Congjiangxiang	CNCJ	Guangxi	25.4	108.5	Yang et al. 2017
Southeast Asian	16-CNCJ-CNCJ_14_J	Congjiangxiang	CNCJ	Guangxi	25.4	108.5	Yang et al. 2017
Southeast Asian	16-CNCJ-CNCJ_15_J	Congjiangxiang	CNCJ	Guangxi	25.4	108.5	Yang et al. 2017
Southeast Asian	16-CNCJ-CNCJ_16_J	Congjiangxiang	CNCJ	Guangxi	25.4	108.5	Yang et al. 2017
Southeast Asian	16-CNCJ-CNCJ_2_J	Congjiangxiang	CNCJ	Guangxi	25.4	108.5	Yang et al. 2017
Southeast Asian	16-CNCJ-CNCJ_3_J	Congjiangxiang	CNCJ	Guangxi	25.4	108.5	Yang et al. 2017
Southeast Asian	16-CNCJ-CNCJ_5_J	Congjiangxiang	CNCJ	Guangxi	25.4	108.5	Yang et al. 2017
Southeast Asian	16-CNCJ-CNCJ_6_J	Congjiangxiang	CNCJ	Guangxi	25.4	108.5	Yang et al. 2017
Southeast Asian	16-CNCJ-CNCJ_8_J	Congjiangxiang	CNCJ	Guangxi	25.4	108.5	Yang et al. 2017
Southeast Asian	16-CNCJ-CNCJ_9_J	Congjiangxiang	CNCJ	Guangxi	25.4	108.5	Yang et al. 2017
Southeast Asian	16-CNDN-CNDN_11_J	Diannanxiaoer	CNDN	Yunnan	21.3	101.3	Yang et al. 2017
Southeast Asian	16-CNDN-CNDN_13_J	Diannanxiaoer	CNDN	Yunnan	21.3	101.3	Yang et al. 2017
Southeast Asian	16-CNDN-CNDN_14_J	Diannanxiaoer	CNDN	Yunnan	21.3	101.3	Yang et al. 2017
Southeast Asian	16-CNDN-CNDN_15_J	Diannanxiaoer	CNDN	Yunnan	21.3	101.3	Yang et al. 2017
Southeast Asian	16-CNDN-CNDN_2_J	Diannanxiaoer	CNDN	Yunnan	21.3	101.3	Yang et al. 2017
Southeast Asian	16-CNDN-CNDN_3_J	Diannanxiaoer	CNDN	Yunnan	21.3	101.3	Yang et al. 2017
Southeast Asian	16-CNDN-CNDN_4_J	Diannanxiaoer	CNDN	Yunnan	21.3	101.3	Yang et al. 2017

Southeast Asian	16-CNDN-CNDN_5_J	Diannanxiaoer	CNDN	Yunnan	21.3	101.3	Yang et al. 2017
Southeast Asian	16-CNDN-CNDN_6_J	Diannanxiaoer	CNDN	Yunnan	21.3	101.3	Yang et al. 2017
Southeast Asian	16-CNDN-CNDN_7_J	Diannanxiaoer	CNDN	Yunnan	21.3	101.3	Yang et al. 2017
Southeast Asian	16-CNDN-CNDN_8_J	Diannanxiaoer	CNDN	Yunnan	21.3	101.3	Yang et al. 2017
Southeast Asian	16-CNDN-CNDN_9_J	Diannanxiaoer	CNDN	Yunnan	21.3	101.3	Yang et al. 2017
Southeast Asian	16-CNDS-CNDS_1_J	Dongshan	CNDS	Guangxi	25.9	111.1	Yang et al. 2017
Southeast Asian	16-CNDS-CNDS_10_J	Dongshan	CNDS	Guangxi	25.9	111.1	Yang et al. 2017
Southeast Asian	16-CNDS-CNDS_11_J	Dongshan	CNDS	Guangxi	25.9	111.1	Yang et al. 2017
Southeast Asian	16-CNDS-CNDS_12_J	Dongshan	CNDS	Guangxi	25.9	111.1	Yang et al. 2017
Southeast Asian	16-CNDS-CNDS_13_J	Dongshan	CNDS	Guangxi	25.9	111.1	Yang et al. 2017
Southeast Asian	16-CNDS-CNDS_14_J	Dongshan	CNDS	Guangxi	25.9	111.1	Yang et al. 2017
Southeast Asian	16-CNDS-CNDS_15_J	Dongshan	CNDS	Guangxi	25.9	111.1	Yang et al. 2017
Southeast Asian	16-CNDS-CNDS_2_J	Dongshan	CNDS	Guangxi	25.9	111.1	Yang et al. 2017
Southeast Asian	16-CNDS-CNDS_3_J	Dongshan	CNDS	Guangxi	25.9	111.1	Yang et al. 2017
Southeast Asian	16-CNDS-CNDS_4_J	Dongshan	CNDS	Guangxi	25.9	111.1	Yang et al. 2017
Southeast Asian	16-CNDS-CNDS_5_J	Dongshan	CNDS	Guangxi	25.9	111.1	Yang et al. 2017
Southeast Asian	16-CNDS-CNDS_6_J	Dongshan	CNDS	Guangxi	25.9	111.1	Yang et al. 2017
Southeast Asian	16-CNDS-CNDS_7_J	Dongshan	CNDS	Guangxi	25.9	111.1	Yang et al. 2017

Southeast Asian	16-CNDS-CNDS_8_J	Dongshan	CNDS	Guangxi	25.9	111.1	Yang et al. 2017
Southeast Asian	16-CNDS-CNDS_9_J	Dongshan	CNDS	Guangxi	25.9	111.1	Yang et al. 2017
Southeast Asian	16-CNDH-CNDH_1_J	Guangdongdahuabai	CNDH	Guangdong	22.5	113.4	Yang et al. 2017
Southeast Asian	16-CNDH-CNDH_10_J	Guangdongdahuabai	CNDH	Guangdong	22.5	113.4	Yang et al. 2017
Southeast Asian	16-CNDH-CNDH_11_J	Guangdongdahuabai	CNDH	Guangdong	22.5	113.4	Yang et al. 2017
Southeast Asian	16-CNDH-CNDH_12_J	Guangdongdahuabai	CNDH	Guangdong	22.5	113.4	Yang et al. 2017
Southeast Asian	16-CNDH-CNDH_13_J	Guangdongdahuabai	CNDH	Guangdong	22.5	113.4	Yang et al. 2017
Southeast Asian	16-CNDH-CNDH_14_J	Guangdongdahuabai	CNDH	Guangdong	22.5	113.4	Yang et al. 2017
Southeast Asian	16-CNDH-CNDH_15_J	Guangdongdahuabai	CNDH	Guangdong	22.5	113.4	Yang et al. 2017
Southeast Asian	16-CNDH-CNDH_16_J	Guangdongdahuabai	CNDH	Guangdong	22.5	113.4	Yang et al. 2017
Southeast Asian	16-CNDH-CNDH_2_J	Guangdongdahuabai	CNDH	Guangdong	22.5	113.4	Yang et al. 2017
Southeast Asian	16-CNDH-CNDH_4_J	Guangdongdahuabai	CNDH	Guangdong	22.5	113.4	Yang et al. 2017
Southeast Asian	16-CNDH-CNDH_5_J	Guangdongdahuabai	CNDH	Guangdong	22.5	113.4	Yang et al. 2017
Southeast Asian	16-CNDH-CNDH_6_J	Guangdongdahuabai	CNDH	Guangdong	22.5	113.4	Yang et al. 2017
Southeast Asian	16-CNDH-CNDH_7_J	Guangdongdahuabai	CNDH	Guangdong	22.5	113.4	Yang et al. 2017
Southeast Asian	16-CNDH-CNDH_8_J	Guangdongdahuabai	CNDH	Guangdong	22.5	113.4	Yang et al. 2017
Southeast Asian	16-CNDH-CNDH_9_J	Guangdongdahuabai	CNDH	Guangdong	22.5	113.4	Yang et al. 2017
Southeast Asian	16-CNDN-CNDN_10_J	Guangdongdahuabai	CNDN	Guangdong	22.5	113.4	Yang et al. 2017

Southeast Asian	16-THJT-THJT_1_W	Jhom Thong Chiang Mai	THJT	Thailand	18.4	98.7	Yang et al. 2017
Southeast Asian	16-THJT-THJT_2_W	Jhom Thong Chiang Mai	THJT	Thailand	18.4	98.7	Yang et al. 2017
Southeast Asian	16-THJT-THJT_3_W	Jhom Thong Chiang Mai	THJT	Thailand	18.4	98.7	Yang et al. 2017
Southeast Asian	16-CNLT-CNLT_1_W	Lantang	CNLT	Guangdong	23.7	114.7	Yang et al. 2017
Southeast Asian	16-CNLT-CNLT_10_W	Lantang	CNLT	Guangdong	23.7	114.7	Yang et al. 2017
Southeast Asian	16-CNLT-CNLT_11_W	Lantang	CNLT	Guangdong	23.7	114.7	Yang et al. 2017
Southeast Asian	16-CNLT-CNLT_12_W	Lantang	CNLT	Guangdong	23.7	114.7	Yang et al. 2017
Southeast Asian	16-CNLT-CNLT_13_W	Lantang	CNLT	Guangdong	23.7	114.7	Yang et al. 2017
Southeast Asian	16-CNLT-CNLT_14_W	Lantang	CNLT	Guangdong	23.7	114.7	Yang et al. 2017
Southeast Asian	16-CNLT-CNLT_16_W	Lantang	CNLT	Guangdong	23.7	114.7	Yang et al. 2017
Southeast Asian	16-CNLT-CNLT_17_W	Lantang	CNLT	Guangdong	23.7	114.7	Yang et al. 2017
Southeast Asian	16-CNLT-CNLT_18_W	Lantang	CNLT	Guangdong	23.7	114.7	Yang et al. 2017
Southeast Asian	16-CNLT-CNLT_19_W	Lantang	CNLT	Guangdong	23.7	114.7	Yang et al. 2017
Southeast Asian	16-CNLT-CNLT_2_W	Lantang	CNLT	Guangdong	23.7	114.7	Yang et al. 2017
Southeast Asian	16-CNLT-CNLT_20_W	Lantang	CNLT	Guangdong	23.7	114.7	Yang et al. 2017
Southeast Asian	16-CNLT-CNLT_3_W	Lantang	CNLT	Guangdong	23.7	114.7	Yang et al. 2017
Southeast Asian	16-CNLT-CNLT_4_W	Lantang	CNLT	Guangdong	23.7	114.7	Yang et al. 2017
Southeast Asian	16-CNLT-CNLT_5_W	Lantang	CNLT	Guangdong	23.7	114.7	Yang et al. 2017

Southeast Asian	16-CNLT-CNLT_6_W	Lantang	CNLT	Guangdong	23.7	114.7	Yang et al. 2017
Southeast Asian	16-CNLT-CNLT_7_W	Lantang	CNLT	Guangdong	23.7	114.7	Yang et al. 2017
Southeast Asian	16-CNLT-CNLT_8_W	Lantang	CNLT	Guangdong	23.7	114.7	Yang et al. 2017
Southeast Asian	16-CNLT-CNLT_9_W	Lantang	CNLT	Guangdong	23.7	114.7	Yang et al. 2017
Southeast Asian	16-CNLP-CNLP_1_W	Leping Spotted	CNLP	Jiangxi	29	117.2	Yang et al. 2017
Southeast Asian	16-CNLU-CNLU_1_J	Luchuan	CNLU	Guangxi	22.3	110.3	Yang et al. 2017
Southeast Asian	16-CNLU-CNLU_10_J	Luchuan	CNLU	Guangxi	22.3	110.3	Yang et al. 2017
Southeast Asian	16-CNLU-CNLU_11_J	Luchuan	CNLU	Guangxi	22.3	110.3	Yang et al. 2017
Southeast Asian	16-CNLU-CNLU_12_J	Luchuan	CNLU	Guangxi	22.3	110.3	Yang et al. 2017
Southeast Asian	16-CNLU-CNLU_13_J	Luchuan	CNLU	Guangxi	22.3	110.3	Yang et al. 2017
Southeast Asian	16-CNLU-CNLU_14_J	Luchuan	CNLU	Guangxi	22.3	110.3	Yang et al. 2017
Southeast Asian	16-CNLU-CNLU_15_J	Luchuan	CNLU	Guangxi	22.3	110.3	Yang et al. 2017
Southeast Asian	16-CNLU-CNLU_16_J	Luchuan	CNLU	Guangxi	22.3	110.3	Yang et al. 2017
Southeast Asian	16-CNLU-CNLU_17_J	Luchuan	CNLU	Guangxi	22.3	110.3	Yang et al. 2017
Southeast Asian	16-CNLU-CNLU_18_J	Luchuan	CNLU	Guangxi	22.3	110.3	Yang et al. 2017
Southeast Asian	16-CNLU-CNLU_2_J	Luchuan	CNLU	Guangxi	22.3	110.3	Yang et al. 2017
Southeast Asian	16-CNLU-CNLU_3_J	Luchuan	CNLU	Guangxi	22.3	110.3	Yang et al. 2017
Southeast Asian	16-CNLU-CNLU_4_J	Luchuan	CNLU	Guangxi	22.3	110.3	Yang et al. 2017

Southeast Asian	16-CNLU-CNLU_5_J	Luchuan	CNLU	Guangxi	22.3	110.3	Yang et al. 2017
Southeast Asian	16-CNLU-CNLU_6_J	Luchuan	CNLU	Guangxi	22.3	110.3	Yang et al. 2017
Southeast Asian	16-CNLU-CNLU_7_J	Luchuan	CNLU	Guangxi	22.3	110.3	Yang et al. 2017
Southeast Asian	16-CNLU-CNLU_8_J	Luchuan	CNLU	Guangxi	22.3	110.3	Yang et al. 2017
Southeast Asian	16-CNLU-CNLU_9_J	Luchuan	CNLU	Guangxi	22.3	110.3	Yang et al. 2017
Southeast Asian	16-THOK-THOK_1_W	Om Koi Chiang Mai	THOK	Thailand	17.9	98.3	Yang et al. 2017
Southeast Asian	16-THOK-THOK_2_W	Om Koi Chiang Mai	THOK	Thailand	17.9	98.3	Yang et al. 2017
Southeast Asian	16-THOK-THOK_3_W	Om Koi Chiang Mai	THOK	Thailand	17.9	98.3	Yang et al. 2017
Southeast Asian	16-THOK-THOK_4_W	Om Koi Chiang Mai	THOK	Thailand	17.9	98.3	Yang et al. 2017
Southeast Asian	16-THOK-THOK_5_W	Om Koi Chiang Mai	THOK	Thailand	17.9	98.3	Yang et al. 2017
Southeast Asian	16-THWB-THWB_2_W	WB_Thailand	THWB	Thailand	18.1	97.9	Yang et al. 2017
Southeast Asian	16-THWB-THWB_3_W	WB_Thailand	THWB	Thailand	18.1	97.9	Yang et al. 2017
Southeast Asian	16-THWB-THWB_4_W	WB_Thailand	THWB	Thailand	18.1	97.9	Yang et al. 2017
Southeast Asian	16-THWB-THWB_5_W	WB_Thailand	THWB	Thailand	18.1	97.9	Yang et al. 2017
Southeast Asian	16-CNWZ-CNWZ_10_J	Wuzhishan	CNWZ	Hainan	18.8	109.5	Yang et al. 2017
Southeast Asian	16-CNWZ-CNWZ_11_J	Wuzhishan	CNWZ	Hainan	18.8	109.5	Yang et al. 2017
Southeast Asian	16-CNWZ-CNWZ_13_J	Wuzhishan	CNWZ	Hainan	18.8	109.5	Yang et al. 2017
Southeast Asian	16-CNWZ-CNWZ_14_J	Wuzhishan	CNWZ	Hainan	18.8	109.5	Yang et al. 2017

Southeast Asian	16-CNWZ-CNWZ_15_J	Wuzhishan	CNWZ	Hainan	18.8	109.5	Yang et al. 2017
Southeast Asian	16-CNWZ-CNWZ_2_J	Wuzhishan	CNWZ	Hainan	18.8	109.5	Yang et al. 2017
Southeast Asian	16-CNWZ-CNWZ_4_J	Wuzhishan	CNWZ	Hainan	18.8	109.5	Yang et al. 2017
Southeast Asian	16-CNWZ-CNWZ_5_J	Wuzhishan	CNWZ	Hainan	18.8	109.5	Yang et al. 2017
Southeast Asian	16-CNWZ-CNWZ_8_J	Wuzhishan	CNWZ	Hainan	18.8	109.5	Yang et al. 2017
Southeast Asian	16-CNWZ-CNWZ_9_J	Wuzhishan	CNWZ	Hainan	18.8	109.5	Yang et al. 2017
Southeast Asian	16-CNXI-CNXI_1_W	Xiang	CNXI	Guizhou	25.9	108.5	Yang et al. 2017
Southeast Asian	16-CNXI-CNXI_11_W	Xiang	CNXI	Guizhou	25.9	108.5	Yang et al. 2017
Southeast Asian	16-CNXI-CNXI_12_W	Xiang	CNXI	Guizhou	25.9	108.5	Yang et al. 2017
Southeast Asian	16-CNXI-CNXI_13_W	Xiang	CNXI	Guizhou	25.9	108.5	Yang et al. 2017
Southeast Asian	16-CNXI-CNXI_2_W	Xiang	CNXI	Guizhou	25.9	108.5	Yang et al. 2017
Southeast Asian	16-CNXI-CNXI_3_W	Xiang	CNXI	Guizhou	25.9	108.5	Yang et al. 2017
Southeast Asian	16-CNXI-CNXI_4_W	Xiang	CNXI	Guizhou	25.9	108.5	Yang et al. 2017
Southeast Asian	16-CNXI-CNXI_8_W	Xiang	CNXI	Guizhou	25.9	108.5	Yang et al. 2017
